# Nurse Care Management of Opioid Use Disorder Treatment After 3 Years: A Secondary Analysis of the PROUD Cluster Randomized Clinical Trial

**DOI:** 10.1001/jamanetworkopen.2024.47447

**Published:** 2024-11-22

**Authors:** Gwen T. Lapham, Noorie Hyun, Jennifer F. Bobb, Paige D. Wartko, Abigail G. Matthews, Onchee Yu, Jennifer McCormack, Amy K. Lee, David S. Liu, Jeffrey H. Samet, Mohammad Zare-Mehrjerdi, Jordan M. Braciszewski, Mark T. Murphy, Julia H. Arnsten, Viviana Horigian, Ryan M. Caldeiro, Megan Addis, Katharine A. Bradley

**Affiliations:** 1 Kaiser Permanente Washington Health Research Institute, Seattle, Washington; 2 Department of Health Systems and Population Health, School of Public Health, University of Washington, Seattle; 3 The Emmes Company, Rockville, Maryland; 4 Mental Health and Wellness Department, Kaiser Permanente Washington; 5 National Institute on Drug Abuse Center for Clinical Trials Network, North Bethesda, Maryland; 6 Boston University Chobanian and Avedisian School of Medicine and the School of Public Health, Boston Medical Center, Boston, Massachusetts; 7 Division of General Internal Medicine, Department of Medicine, University of Washington School of Medicine, Seattle; 8 Department of Family and Community Medicine, UTHealth Houston McGovern Medical School, Houston, Texas; 9 Center for Health Policy and Health Services Research, Henry Ford Health, Detroit, Michigan; 10 MultiCare Health System, Tacoma, Washington; 11 Montefiore Medical Center, Bronx, New York; 12 Albert Einstein College of Medicine, New York, New York; 13 Department of Public Health Sciences, University of Miami Miller School of Medicine, Miami, Florida; 14 Department of Epidemiology, School of Public Health, University of Washington, Seattle

## Abstract

**Importance:**

The Primary Care Opioid Use Disorders (PROUD) treatment trial was a 2-year implementation trial that demonstrated the Massachusetts office-based addiction treatment (OBAT) model of nurse care management for opioid use disorder (OUD) increased OUD treatment in the 2 years after implementation began (8.2 more patient-years of OUD treatment per 10 000 primary care patients). The intervention was continued for a third year, permitting evaluation of 3-year outcomes.

**Objective:**

To compare OUD medication treatment in intervention and usual care clinics over 3 years of implementation.

**Design, Setting, and Participants:**

This is a preplanned secondary analysis of a cluster randomized implementation trial, conducted in 6 health systems in 5 states (2 primary care clinics per health system) with clinic randomization stratified by system (assignment notification February 28, 2018 [August 31, 2018, in 1 system]). Data were obtained from electronic health records and insurance claims. Eligible patients were those aged 16 to 90 years visiting intervention or usual care clinics from 3 years before to 2 years after randomization. Patients new to clinics during the third year after randomization could not be included because COVID-19–era transitions to virtual care precluded assignment of patients to clinics. Data analysis occurred from November 2023 to September 2024.

**Intervention:**

Clinics were randomized to intervention or care as usual. Intervention included 3 implementation components: salary for 1 full-time OBAT nurse per intervention clinic; training and ongoing technical assistance for nurses; and 3 or more primary care buprenorphine prescribers.

**Main Outcome and Measures:**

Patient-years of OUD treatment (buprenorphine or extended-release naltrexone) per 10 000 primary care patients in the 3 years postrandomization. Mixed-effect models adjusted for baseline values of the outcome and included a health system–specific random intercept to account for correlation of clinic pairs within a system.

**Results:**

Prerandomization, a total of 290 071 primary care patients were seen, including 130 618 in intervention clinics (mean [SD] age, 48.6 [17.7] years; mean [SD] female, 59.3% [4.0%]) and 159 453 in usual care clinics (mean [SD] age, 47.2 [17.5] years; mean [SD] female, 64.0% [5.3%]). Over 3 years postrandomization, intervention clinics provided 19.7 (95% CI, 11.1-28.4) more patient-years of OUD treatment per 10 000 primary care patients compared with usual care clinics.

**Conclusions:**

In this secondary analysis of the PROUD cluster randomized trial, after an added year of the intervention, OUD treatment continued to increase in intervention clinics compared with usual care. The treatment increase over 3 years exceeded that of the first 2 years, suggesting that implementation of the Massachusetts OBAT model leads to ongoing increases in OUD treatment among primary care patients in the third year of implementation.

**Trial Registration:**

ClinicalTrials.gov Identifier: NCT03407638

## Key Points

**Question:**

How does nurse care management of office-based addiction treatment (OBAT) for opioid use disorder (OUD) affect OUD medication treatment 3 years after implementation?

**Findings:**

This secondary analysis of a cluster randomized implementation trial of 290 071 primary care patients from 6 health systems found that after 3 years, intervention clinics provided 19.7 more patient-years of OUD treatment per 10 000 patients than usual care clinics.

**Meaning:**

In 3 years since the start of OBAT implementation, the increase in OUD treatment in intervention compared with usual care clinics was greater than during the first 2 years of implementation, suggesting that integration of a new model of OUD treatment into primary care takes time to overcome barriers.

## Teaser

This secondary analysis of a cluster randomized trial assesses 3-year outcomes of the Massachusetts office-based addiction treatment (OBAT) model of nurse care management for opioid use disorder.

## Introduction

Deaths from opioid use continue to increase.^1,2^ Untreated opioid use disorder (OUD) causes substantial mortality and morbidity worldwide, and medication treatment of OUD is an essential care component that saves lives.^3,4^ However, most people with OUD do not receive medication treatment.^3,4,5,6,7^ Multiple models to increase OUD treatment in primary care have been proposed, with no demonstrated increase in OUD medication treatment until recently.^8,9,10^

The Primary Care OUD (PROUD) treatment trial was a cluster randomized implementation trial of the Massachusetts model of office-based addiction treatment (OBAT) across 6 diverse health systems, conducted by the National Institute on Drug Abuse Clinical Trials Network (CTN).^8,11,12,13,14^ OBAT integrates full-time nurse care managers into primary care for a team-based approach to providing OUD medication treatment (with buprenorphine or extended-release injectable naltrexone [XR-NTX]).^11,15^ The PROUD trial tested whether implementation of this model would increase OUD treatment among primary care patients, compared with usual care, in the 2 years after implementation start. The primary implementation strategy of this nurse-led model is to fund a full-time nurse to support OUD care. Additional PROUD trial implementation strategies included training and ongoing technical assistance for the nurses from external nurse experts and requiring 3 primary care clinicians to prescribe medications for OUD.

The PROUD trial demonstrated that the Massachusetts OBAT model meaningfully increased OUD treatment in primary care, with clinics randomized to the PROUD intervention increasing OUD treatment by 8.2 patient-years per 10 000 primary care patients, compared with usual care in the 2 years after randomization.^16^ The trial also demonstrated important variation across health systems, with significant increases in OUD treatment in 2 of 6 health systems.^16^ However, hiring and onboarding nurses takes time, and in PROUD, nurses did not start seeing patients until 4 to 15 months after randomization, contributing to low numbers of patients treated in the first year postrandomization.^11,16^ Implementation barriers included clinician and staff ambivalence toward treating patients with OUD, uneven support for the nurse role, and limited knowledge about or comfort in diagnosing OUD. A potential benefit of the Massachusetts OBAT model, once implemented, is a shift in clinic culture toward a more accepting approach to OUD care that encourages patient trust, destigmatizes treatment, and supports treatment retention.^17^ Thus, benefits of the model could increase over time.

To evaluate benefits of the model over 3 years of implementation, a decision was made early in year 2 of the trial to fund an additional year of the intervention to evaluate implementation outcomes over 3 years. In this preplanned secondary analysis, we describe the results of the PROUD primary outcome—patient-years of OUD treatment per 10 000 primary care patients—as well as secondary outcomes over 3 years postrandomization.

## Methods

This preplanned secondary analysis of the PROUD trial was approved by the Advarra institutional review board. The trial protocol and statistical analysis plan are in Supplement 1. A data and safety monitoring board oversaw the trial, and we followed the Consolidated Standards of Reporting Trials Extension (CONSORT Extension) reporting guideline.^18^

### Setting

PROUD was conducted in 6 health systems in New York, Florida, Michigan, Texas, and Washington.^19^ Each system included 2 primary care clinics or groups of smaller, geographically proximal clinics; the 12 clinics were randomized (1:1) to either intervention or usual care, stratified by health system (eFigure 1 in Supplement 2).^16^ Randomization occurred on February 28, 2018, for 5 health systems (and on August 29, 2018, for 1 system), with intervention clinics able to begin implementation at that time. Although the intervention was planned for 2 years, due to slow start-up, the CTN funded a third year. This secondary analysis evaluates the PROUD intervention over 3 intervention years.

### Data Collection and Participants

As a pragmatic trial, health systems implemented the PROUD intervention for all their patients, with the study relying on existing data to identify the sample and measure outcomes. Patients were included in the trial if they were aged 16 to 90 years and visited a participating clinic anytime in the 3 years before the health system’s randomization date until 2 years after randomization (1.5 years in 1 site) (eFigure 2 in Supplement 2). Thus, PROUD was an open cohort trial, with new patients added to the cohort postrandomization and assigned to clinics based on the clinic or clinics they visited. The trial did not recruit patients; therefore, consent was not required. PROUD received waivers of informed consent and Health Insurance Portability and Accountability Act authorization from the Advarra institutional review board.

Of note, year 3 of PROUD coincided with the first year of the COVID-19 pandemic (March 2020 to February 2021), and the shift to virtual care precluded assignment of new patients to specific clinics. As a result, no new patients were added to the study cohort year 3. Thus, the sample for this 3-year evaluation was the same as the main trial. Patients who had outcomes measured in the third year postrandomization had a visit to a trial clinic in year 3 and at least 1 visit in the prior 5 years (ie, during 3 baseline years or 2 postrandomization years).

Data were extracted from electronic health records (EHRs), including claims, from each health system and securely transferred to Kaiser Permanente Washington. Data included patient demographics, diagnoses, procedure codes, outpatient prescription medication orders and health care utilization. The baseline period for outcome measurement was 2 years prerandomization, while the follow-up period for outcome measurement was 3 years postrandomization (2.5 years in 1 system) (eFigure 3 in Supplement 2).

### Implementation Intervention

Design of the PROUD implementation trial was guided by the Practical, Robust Implementation and Sustainability Model (PRISM) framework,^19,20^ with selection of outcomes guided by the Reach, Effectiveness, Adoption, Implementation Fidelity, and Maintenance framework embedded within PRISM.^21,22^ The PROUD intervention^19^ included 3 implementation strategies to support implementation of OBAT in intervention clinics: funding for a full-time nurse in each clinic, training and technical assistance for nurses from OBAT nurse educators at Boston Medical Center,^23^ and an agreement that 3 or more primary care clinicians would prescribe buprenorphine, obtaining training and waivers if necessary, for clinics randomized to the intervention. All 3 components were continued for the third year. Clinics randomized to usual care did not receive support from the study but were allowed to improve OUD care if desired, although they were asked not to use Boston Medical Center OBAT Manual during the main trial.^23^

### Measures

Baseline descriptive measures include numbers of primary care patients, clinicians, and buprenorphine prescribers, patient sociodemographics, and clinical characteristics. The primary outcome of patient-years of OUD treatment per 10 000 patients was also described at baseline.

#### Primary Outcome

The primary implementation outcome was a clinic-level measure of the number of patient-years of OUD medication treatment with buprenorphine or XR-NTX per 10 000 patients seen during follow-up.^16^ Medication treatment was defined as having a medication order or procedure code for buprenorphine formulations indicated for OUD (buccal, sublingual, implant, or subcutaneous injection) or for XR-NTX with OUD diagnosis. Days of medication treatment were measured based on orders and procedures documented in the EHR and divided by 365 days for patient-years of treatment. To account for varying clinic sizes, we divided the outcome by the number of patients seen in the clinic during follow-up and multiplied by 10 000 to calculate the number of patient-years of OUD treatment per 10 000 patients seen.

#### Secondary Outcomes

Secondary outcomes were reported at the clinic level per 10 000 patients seen. We calculated the primary outcome for 2 mutually exclusive patient groups based on timing of initiation of OUD treatment: patients who newly initiated treatment (defined as OUD treatment for those with no treatment in the prior 365 days) and patients who had ongoing treatment (defined as OUD treatment for patients with less than a 365-day gap in treatment).

Additionally, we calculated the primary outcome restricted to the time period in which the nurse was seeing patients for each health care system (eFigure 3 in Supplement 2) and included a measure for newly initiated treatment during the time when the nurse was seeing patients in each health system (ie, restricted to the time period when the nurse began treating their first patient and ended treating their last patient at the intervention clinic for each health system, which applied to both the intervention and usual care clinics [802 days for health system 1, 690 days for health system 2, 826 days for health system 3, 616 days for health system 4, 717 days for health system 5, and 941 days for health systems 6]). We evaluated a common OUD treatment quality metric (the proportion of patients with an OUD diagnosis that received OUD medication treatment)^24,25,26^ to allow comparison with other research.^27^ Lastly, we measured number of patients per 10 000 patients seen in a clinic per month who received OUD medication treatment each month from baseline through follow-up, as well as the cumulative number treated per 10 000 patients each month after randomization.

### Statistical Analyses

Baseline characteristics of patients in the 2 years prior to randomization, as well as staffing and size of clinics, were summarized for intervention and usual care clinics. Unadjusted numbers of patients treated for OUD each month (per 10 000 patients seen per month) in intervention and usual care clinics were summarized over the study period, with cumulative numbers summarized postrandomization.^16^

Primary analyses—intent-to-treat analyses with the 12 clinics as the unit of analysis—compared mean patient-years of treatment in intervention and usual care clinics using a mixed-effect model to account for correlation of outcomes between pairs of clinics in the same system, adjusted for baseline values of the outcome, as for the primary trial results (Supplement 1).^16^ The main outcome was also evaluated by health system due to site-level variation 2 years postrandomization.^16^ All tests were 2-sided and conducted at the significance level of less than .05.

Secondary outcomes were analyzed overall and by health system. We estimated the difference in postrandomization and prerandomization patient-years of OUD treatment per 10 000 patients between intervention and usual care clinics for each health system, and obtained 2-sided, 95% CIs by using the bootstrap *t* method^28^ to resample patient-level data (Supplement 1). In addition, we summarized the primary outcome (mean and SD for patient-years of OUD treatment) by trial group for each of 2 years prerandomization and 3 years postrandomization, without adjustment, and estimated the proportion of patients who newly initiated OUD treatment prerandomization and postrandomization. We summarized the prevalence of OUD treatment for patients who received an OUD diagnosis in patient-level analyses by trial group. A post hoc analysis evaluated sensitivity of the results to statistical modeling assumptions with a permutation test (Supplement 1).^29^ Analyses were conducted in R version 4.3.1 (R Project for Statistical Computing) from November 2023 to September 2024.^30^

## Results

A total of 290 071 primary care patients were included at baseline, of whom 130 618 were seen in PROUD intervention clinics and 159 453 were seen in usual care clinics(Table 1). During the baseline period, intervention and usual care clinics saw a mean (SD) 18 530 (4074) and 22 664 (8265) patients, respectively. Patient characteristics varied between intervention (mean [SD] age, 48.6 [17.7] years; mean [SD] female, 59.3% [4.0%]) and usual care clinics (mean [SD] age, 47.2 [17.5]; mean [SD] female, 64.0% [5.3%]), with usual care clinics providing more OUD treatment at baseline compared with intervention clinics (mean [SD], 10.6 [7.4] vs 6.8 [5.5] patient-years of OUD treatment per 10 000 patients). Cumulative unadjusted numbers of patients treated for OUD in intervention clinics exceeded those in usual care clinics by 13 months and continued to increase through 3 years postrandomization (eFigure 4 in Supplement 2). Results of cumulative unadjusted numbers of patients treated varied by health system (eFigure 5 in Supplement 2).

**Table 1.** Characteristics of Clinics’ Patient Populations in the Baseline Period (2 Years Prior to Randomization)

Characteristic	Clinic mean (SD), prerandomization	
	PROUD intervention	Usual care
Primary care patients in each trial group, No.^a^	130 618	159 453
Staffing and size of clinics in baseline period		
No. of clinicians who prescribe in clinic^b^	31.7 (20.0)	36.8 (18.8)
No. of primary care buprenorphine prescribers in clinic^c^	1.5 (1.0)	1.5 (2.0)
No. of patients seen in clinic^d^	18 530 (4074)	22 664 (8265)
Proportion of clinics’ patient population, %		
Age, y^e^		
16-17	2.2 (1.7)	1.9 (1.4)
18-24	8.5 (2.2)	10.2 (3.0)
25–44	30.1 (4.9)	34.3 (7.2)
45–64	39.0 (4.3)	36.7 (6.9)
65-74	12.6 (3.1)	10.4 (3.0)
≥75	7.6 (2.9)	6.4 (3.2)
Sex		
Female	59.3 (4.0)	64.0 (5.3)
Male	40.7 (4.0)	36.0 (5.3)
Race and ethnicity		
Hispanic ethnicity	26.4 (25.6)	33.4 (29.9)
Non-Hispanic ethnicity		
Asian	5.2 (3.8)	5.0 (5.0)
Black or African American	18.4 (14.4)	18.9 (13.4)
American Indian or Alaska Native	0.5 (0.6)	0.5 (0.6)
Native Hawaiian or Pacific Islander	0.4 (0.5)	0.8 (1.1)
White	40.9 (33.3)	31.1 (24.7)
Multiple race	0.4 (0.5)	0.8 (1.3)
Other race^f^	2.4 (2.4)	2.9 (2.7)
Missing race and ethnicity data	5.4 (2.8)	6.7 (2.6)
Insurance status closest to randomization		
Medicare	21.1 (7.0)	18.3 (8.6)
Medicaid	27.7 (31.7)	35.7 (33.3)
Otherwise insured (eg, commercial or private)	57.7 (25.2)	53.1 (27.7)
Uninsured	5.3 (5.9)	4.5 (5.4)
Unknown	1.0 (1.4)	1.0 (1.2)
Patients’ neighborhood^g^		
Median household income, $	61 270.0 (24 151.8)	54 467.7 (16 779.0)
Unemployed	6.1 (2.9)	7.2 (2.3)
Below federal poverty level	16.0 (11.2)	18.2 (9.7)
Any mental health diagnoses	23.3 (5.0)	22.8 (6.7)
Depression	13.8 (3.8)	13.4 (5.6)
Anxiety	15.4 (3.4)	15.2 (3.6)
Attention-deficit/hyperactivity disorder	1.2 (0.9)	1.1 (0.7)
Posttraumatic stress disorder	1.0 (0.6)	1.1 (0.7)
Schizophrenia or psychoses	0.8 (0.5)	0.8 (0.4)
Other mental health conditions	1.8 (0.5)	1.8 (0.9)
Any substance use disorder diagnoses		
Opioid	0.7 (0.5)	0.8 (0.5)
Tobacco	7.4 (4.1)	8.2 (4.4)
Alcohol	2.0 (1.0)	1.8 (0.8)
Cannabis	0.8 (0.7)	0.7 (0.4)
Stimulant	0.4 (0.3)	0.4 (0.3)
Other	0.6 (0.3)	0.5 (0.3)
Baseline value of PROUD trial outcome: patient-years of OUD treatment per 10 000 primary care patients	6.8 (5.5)	10.6 (7.4)

Abbreviations: OUD, opioid use disorder; PROUD, Primary Care OUD Treatment trial.

^a^ All patients assigned to the PROUD intervention or usual care clinics, based on an eligible primary care visit in the 3 years prerandomization.

^b^ Number and type (eg, physician, physician assistant, advanced registered nurse practitioner, or Doctor of Osteopathic Medicine) determined from encounter data in the electronic health record during the 2 years prerandomization; clinicians assigned to clinics based on number of visits in clinic prerandomization.

^c^ Primary care clinicians who can prescribe buprenorphine determined from electronic health record medication orders and procedures.

^d^ Number of patients seen in each clinic during the 2 years prerandomization.

^e^ At eligible visit closest to and prior to randomization date.

^f^ Other race was recorded in the electronic health record when a person did not identify as belonging to any of the previously listed racial groups and was not Hispanic.

^g^ Using zip code of residence at time closest to randomization date.

### OUD Treatment Postrandomization

Compared with usual care clinics, intervention clinics provided 19.7 (95% CI, 11.1 to 28.4) more patient-years of OUD treatment per 10 000 primary care patients in the 3 years postrandomization after adjustment for baseline (Table 2). These results were confirmed post hoc using a permutation test (*P* = .03). For patients who newly initiated treatment postrandomization, the mean difference in the outcome between intervention and usual care clinics was 19.2 (95% CI, 8.4 to 30.0) patient-years of OUD treatment per 10 000 primary care patients, whereas for patients with ongoing treatment, the mean difference was 0.1 (95% CI, −4.6 to 4.8) patient-years of OUD treatment per 10 000 primary care patients. For secondary outcomes, the mean difference was 21.9 (95% CI, 9.9 to 33.9) patient-years of OUD treatment per 10 000 primary care patients when restricted to when the nurse was seeing patients and 21.4 (95% CI, 4.7 to 38.0) patient-years of OUD treatment per 10 000 primary care patients for those who newly initiated treatment when the nurse was seeing patients. When patient-years of OUD treatment was described for each study year, intervention clinics demonstrated increases in OUD treatment years 2 and 3, with the greatest increase in year 3, largely among patients who newly initiated treatment postrandomization (Figure).

**Table 2.** Clinic-Level Implementation Outcomes of the PROUD Trial Assessed in the 3 Years Postrandomization^a^

Outcome	Clinic mean (SD) postrandomization		Mean difference (95% CI)^c^
	PROUD intervention (n = 171 510)^b^	Usual care (n = 205 833)^b^	
Primary outcome: patient-years of OUD treatment^d^^,^^e^	30.6 (23.1)	22.8 (18.8)	19.7 (11.1 to 28.4)
Secondary outcomes of patient-years of OUD treatment			
Timing of treatment initiation			
Ongoing treatment	7.2 (7.1)	10.7 (6.5)	0.1 (−4.6 to 4.8)
Newly initiated treatment	23.4 (16.2)	12.1 (14.2)	19.2 (8.4 to 30.0)
Restricted to when nurse was seeing patients	30.3 (26.0)	20.2 (18.1)	21.9 (9.9 to 33.9)
Newly initiated treatment postrandomization and when nurse was seeing patients^d^	24.0 (19.2)	12.1 (14.9)	21.4 (4.7 to 38.0)

Abbreviations: OUD, opioid use disorder; PROUD, Primary Care OUD Treatment trial.

^a^ Results presented per 10 000 primary care patients by dividing by the number of patients seen in the clinic postrandomization and multiplied by 10 000.

^b^ Patients in each trial group were eligible 3 years prior to randomization and up to 2 years postrandomization.

^c^ Random-effects model adjusted for the outcome measure at baseline (2 years prior to randomization).

^d^ Treatment defined as having a medication order or procedure code for buprenorphine formulations that are indicated for OUD or having a medication order or procedure code for extended-release injectable naltrexone.

^e^ The intraclass correlation within cluster is 0.72.

**Figure.**
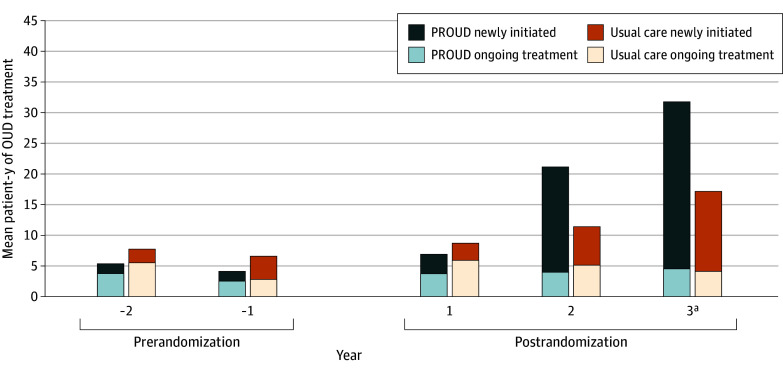
Mean Patient-Years of Opioid Use Disorder (OUD) Treatment per 10 000 Patients per Clinic by Year, Prerandomization and Postrandomization Newly initiated was defined as OUD treatment for those with no OUD treatment in the prior 365 days, and ongoing treatment was defined as OUD treatment for patients with less than a 365-day gap in OUD treatment, separately for patients in the prerandomization and postrandomization periods, unadjusted for baseline. PROUD indicates Primary Care OUD Treatment trial. ^a^No new primary care patients could be added in year 3; increases in treatment in year 3 are based on patients assigned to a primary care clinic from baseline through year 2.

Among patients with a documented OUD diagnosis, the proportion that received treatment increased more in intervention than usual care clinics (65 of 644 patients at baseline [10.1%] and 281 of 962 at follow-up [29.2%; a 19.1–percentage point increase] for intervention clinics, compared with 118 of 734 at baseline [16.1%] and 188 of 935 at follow-up [20.1%; a 4.0–percentage point increase] for usual care clinics). Thus, there was a 15.1% absolute increase in proportion of patients with documented OUD who received treatment in intervention compared with usual care clinics.

### Health System–Specific Outcomes

Outcomes varied across health systems (Table 3). For the primary outcome, the intervention yielded significant benefit in 3 health systems over the 3 years postrandomization. For the secondary outcome of treatment among patients who newly initiated treatment postrandomization, the intervention yielded significant benefit in 4 health systems, ranging between 9.4 (95% CI, 1.1-14.0) and 43.0 (95% CI, 28.0-55.8) patient-years of OUD treatment per 10 000 patients. Out of concern that health system 1, with a primary outcome mean difference of 45.6 patient-years of OUD treatment per 10 000 patients, may have influenced main results, a post hoc analysis excluded this health system. Although the adjusted mean difference of 15.9 (95% CI, 4.3-27.5) more patient-years of OUD treatment per 10 000 patients was lower than the main effect (19.7 patient-years of OUD treatment per 10 000 patients), results remained significant.

**Table 3.** Clinic-Level Implementation Outcomes of the PROUD Trial Assessed in the 3 Years Postrandomization by Health System and Clinic^a^

Outcome	Outcome value by health system and clinic											
	Health system 1		Health system 2		Health system 3		Health system 4		Health system 5		Health system 6	
	PROUD	Usual care	PROUD	Usual care	PROUD	Usual care	PROUD	Usual care	PROUD	Usual care	PROUD	Usual care
Patients, No.^b^	30 708	52 486	20 336	41 296	21 265	21 339	30 138	20 881	29 572	27 789	39 491	42 042
Primary outcome: patient-years of OUD treatment, No.^c^												
Prerandomization	13.3	3.9	5.3	11.2	8.3	20.3	1.1	13.6	0.4	0	12.4	14.3
Postrandomization	68.1	13.1	22.1	17.7	33.7	56.3	6.5	23.5	9.8	0	43.4	26
Difference (post vs pre)^d^	54.8	9.2	16.7	6.5	25.5	36.0	5.5	9.8	9.5	0	31.0	11.7
PROUD vs usual care difference (95% CI)^e^^,^^f^	45.6 (31.8 to 58.5)		10.2 (−1.4 to 20.3)		−10.5 (−28.4 to 9.0)		−4.4 (−12.4 to 4.4)		9.5 (1.2 to 14.0)		19.3 (8.5 to 30.7)	
Secondary outcomes of patient-years of OUD treatment												
Ongoing treatment^g^												
Prerandomization	9.2	1.1	4.7	9.3	4.9	8.3	0.3	10.1	0	0	8.3	8.2
Postrandomization	18.0	7.5	4.1	11.9	11.3	17.1	0	17.4	0	0	9.7	10.4
Difference (post vs pre)^d^	8.9	6.3	−0.6	2.6	6.4	8.9	−0.3	7.3	0	0	1.5	2.2
PROUD vs usual care difference (95% CI)^e^^,^^h^	2.5 (−5.5 to 10.6)		−3.2 (−8.9 to 2.5)		−2.4 (−13.6 to 8.8)		−7.6 (−15.8 to 0.7)		NA		−0.7 (−8.5 to 7.1)	
Newly initiated^e^^,^^i^												
Prerandomization	4.2	2.8	0.6	2.0	3.4	12.0	0.7	3.5	0.4	0	4.1	6.0
Postrandomization	50.1	5.7	18.0	5.8	22.4	39.2	6.5	6.1	9.8	0	33.7	15.6
Difference (post vs pre)^d^	45.9	2.9	17.3	3.9	19.0	27.1	5.8	2.6	9.4	0	29.6	9.6
PROUD vs usual care difference (95% CI)^e^^,^^f^	43.0 (28 to 55.8)		13.4 (1.5 to 21.8)		−8.1 (−25 to 8.8)		3.2 (−2.4 to 9.3)		9.4 (1.1 to 14.0)		20.0 (9.8 to 29.6)	
Restricted to when nurse was seeing patients												
Prerandomization	13.3	3.9	5.3	11.2	8.3	20.3	1.1	13.6	0.4	0	12.4	14.3
Postrandomization	73.9	13.5	16.9	12.6	32.0	53.1	6.2	16.5	8.0	0.0	44.6	25.3
Difference (post vs pre)^d^	60.6	9.6	11.6	1.3	23.7	32.8	5.2	2.9	7.6	0.0	32.2	11.1
PROUD vs usual care difference (95% CI)^e^^,^^f^	51.0 (35.9 to 65.2)		10.2 (0.7 to 18.8)		−9.1 (−26.6 to 11.3)		2.3 (−5.3 to 9.1)		7.6 (1.0 to 11.3)		21.1 (9.5 to 32.8)	
Newly initiated and restricted to when nurse was seeing patients^i^												
Prerandomization	4.2	2.8	0.6	2.0	3.4	12.0	0.7	3.5	0.4	0	4.1	6.0
Postrandomization	56.3	6.3	14.4	4.1	23.2	40.5	6.2	5.7	8.0	0	35.7	16.1
Difference (post vs pre)^d^	52.2	3.5	13.8	2.2	19.8	28.5	5.5	2.2	7.6	0.0	31.6	10.0
PROUD vs usual care difference (95% CI)^e^^,^^f^	48.7 (31.9 to 62.9)		11.6 (1.4 to 18.7)		−8.7 (−25.4 to 8.3)		3.3 (−2.8 to 9.8)		7.6 (1.0 to 11.3)		21.6 (10.9 to 31.8)	

Abbreviations: NA, not applicable; OUD, opioid use disorder; PROUD, Primary Care OUD Treatment trial.

^a^ Results presented per 10 000 primary care patients, which was calculated by dividing by the number of patients seen in the clinic postrandomization and multiplied by 10 000.

^b^ Number of eligible primary care patients assigned to the clinics in 3 years prior to randomization and up to 2 years postrandomization.

^c^ Treatment was defined as having a medication order or procedure code for buprenorphine formulations that are indicated for OUD or having a medication order or procedure code for extended-release injectable naltrexone.

^d^ Calculated by subtracting prerandomization from postrandomization patient-years of OUD treatment for each clinic.

^e^ Calculated by subtracting the usual care difference (in prerandomization and postrandomization patient-years of OUD treatment) from the intervention clinic difference for each health system.

^f^ 95% CI is based on bootstrap *t* method, described in the statistical analysis plan in Supplement 1.

^g^ Ongoing treatment was defined as OUD treatment for patients with less than a 365-day gap in OUD treatment.

^h^ 95% CI is based on a normal approximation rather than bootstrap *t* method due to rare outcome events.

^i^ Newly initiated was defined as OUD treatment for those with no OUD treatment in the past 365 days.

## Discussion

This secondary analysis of the PROUD cluster randomized clinical trial evaluated the effect of implementation of the Massachusetts OBAT model over 3 years after implementation began in the PROUD trial. Whereas main trial results showed significant increases in OUD treatment (8.2 patient-years of OUD treatment per 10 000 patients more in intervention compared with usual care clinics at 2 years follow-up^16^), the current study demonstrated increased OUD treatment by 19.7 patient-years per 10 000 primary care patients over 3 years, an increase of 11.5 patient-years of treatment over the difference seen at 2 years. After 3 years of implementation, a statistically significant benefit of the intervention on the primary outcome was observed in 3 health systems, whereas only 2 health systems had significant benefit at 2 years. Moreover, analyses of secondary outcomes among newly initiated patients showed benefit of the intervention in 4 of the 6 health systems, suggesting that more than 2 years is needed to test the success of this model in some settings.

It is noteworthy that the impact of the PROUD intervention grew considerably in the third year of implementation despite the fact that more than 70% of the increase in OUD treatment in the first 2 years of implementation was among patients new to the primary care clinics.^16^ In year 3, new patients to primary care could not be included in the trial because virtual visits due to COVID-19 were not easily assigned to specific clinics; this suggests that had this study been able to capture patients new to clinics year 3, the benefit of the intervention might have been substantially larger. The importance of new patients to the effect of the PROUD intervention in contrast with usual care is demonstrated in eFigure 4 in Supplement 2; the cumulative number of patients treated in intervention clinics increased in parallel with usual care clinics, whereas the number of patients in active treatment decreased in intervention clinics in the third year postrandomization compared with usual care.

Implementation of OUD medication treatment has proven challenging.^31,32,33^ In year 3, the PROUD intervention continued to expand treatment to more primary care patients already in the clinics and appeared to increase treatment in health systems that had not increased OUD treatment after 2 years. Removal of barriers to OBAT could improve uptake; for example, recent elimination of waiver requirements to prescribe buprenorphine^34^ may serve to increase the number of clinicians willing to treat OUD. Moreover, interventions to help clinicians optimize buprenorphine initiation practices may improve initiation and retention.^13,35,36^

### Limitations

This study has limitations. As noted, the third year postrandomization coincided with the COVID-19 pandemic, and although shifts to virtual care have not demonstrated an effect on OUD treatment,^37^ it is possible the shift increased effectiveness of the OBAT model, accounting for some observed findings. The shift made assigning patients’ virtual visits to a clinic infeasible. As a result, patients with visits for the first time in year 3 could not be reliably assigned to clinics and were excluded. Given that many patients who benefit from OBAT are new to the OBAT clinic,^11,16^ exclusion of new patients likely attenuated both intervention and usual care OUD treatment outcomes compared with results if we could have captured new patients in year 3. In addition, while COVID-19–era shifts in OUD treatment policies at local, state, and federal levels may have sustained or expanded treatment access,^38,39,40^ such policies varied by location, were not consistently adopted,^41^ and could not be accounted for here. However, such policy shifts would be expected to have a similar effect on both trial groups in each system. The study included a small number of clusters with heterogeneous effects, which can be sensitive to a modeling approach, although post hoc permutation analyses and analyses that removed 1 health system with the largest effect confirmed findings.^16^ While the main trial included qualitative implementation findings,^16^ and additional analyses are ongoing, it was beyond this study’s scope to assess changes in qualitative findings during year 3. Therefore, this study could not identify the implementation factors that contributed to observed increases in treatment in intervention clinics year 3. This study relied on EHR medication orders available from all health systems, rather than dispensed medications, and thus does not include OUD treatment with methadone and other OUD treatment not captured in orders. Health systems and clinics had to agree to participate, potentially limiting generalizability. The patient-level metric for percentage of patients with an OUD diagnosis who received medication treatment may be biased because the intervention could attract or provide diagnoses for patients with OUD who differed from those with OUD in usual care, differentially increasing patients who both received an OUD diagnosis and were treated for OUD in the intervention group.^42^

## Conclusions

In this secondary analysis of the PROUD cluster randomized trial, intervention clinics demonstrated meaningful increases in OUD treatment over 3 years, more than double the increases through the first 2 years of implementation. Three years after implementation began, 4 of 6 health systems had significant increases in primary or secondary outcomes, compared with 2 health systems after the first 2 years of implementation. Results suggest integration of a new model of OUD treatment into primary care takes time to overcome barriers and that implementation of the Massachusetts OBAT model leads to ongoing increases in OUD treatment among primary care patients in the third year of implementation.

## Notes

Supplement 1.
Trial Protocol and Statistical Analysis Plan

Supplement 2. **eFigure 1.** PROUD Study Flow Diagram **eFigure 2.** PROUD Study Timeline and Sample, With an Additional Year for the Nurse Care Manager and Outcome Measurement **eFigure 3.** Calendar Dates of When the Nurse Was Hired and When the Nurse Was Seeing Patients in Each Health System After Randomization **eFigure 4.** Number of Patients Treated for OUD in Intervention and Usual Care Clinics per 10,000 Primary Care Patients per Month: Cumulative Numbers of Patients Treated for OUD Since Randomization, and Numbers in Active OUD Treatment Each Month From Baseline Through Follow-Up (Unadjusted) **eFigure 5.** Cumulative Numbers of Patients Treated for OUD per 10,000 Patients Seen by Trial Arm for Each Health System During 3 Years of Follow-Up in Intervention and Usual Care Clinics, Unadjusted for Baseline

Supplement 3.
Data Sharing Statement
