# Supplementary material for: Nurse Care Management of Opioid Use Disorder Treatment After 3 Years: A Secondary Analysis of the PROUD Cluster Randomized Clinical Trial
Source: JAMA Netw Open. 2024 Nov 22;7(11):e2447447. doi: 10.1001/jamanetworkopen.2024.47447 (PMC11584924; doi:10.1001/jamanetworkopen.2024.47447)
Supplement: Supplement 1. — Trial Protocol and Statistical Analysis Plan [file jamanetwopen-e2447447-s001.pdf]

NIDA CTN Protocol 0074

PRimary care Opioid Use Disorders Treatment (PROUD) Trial

Lead Investigator: Katharine Bradley, MD, MPH

November 18, 2022

Version 9.0

TABLE OF CONTENTS

A. PROUD PROTOCOL MODIFICATIONS A-1
1.0 LIST OF ABBREVIATIONS 7
2.0 STUDY SYNOPSIS 10
2.1 Study Objectives 10
2.2 Study Design 10
2.3 Sample and Sample Size 11
2.4 The PROUD Intervention and Duration 11
2.5 Safety Reporting 12
2.6 Study Outcomes 12
2.7 Analyses 12
3.0 INTRODUCTION 14
3.1 Background and Significance to the Field 14
3.1.1 Epidemic of Opioid Use Disorder in US 14
3.1.2 Improved OUD Outcomes with Medication Treatment 14
3.1.3 Two Medications for OUDs Can be Prescribed in PC 15
3.1.4 The Need to Improve Access to Medication Treatment for OUD 15
3.1.5 OUD Treatment in PC Could Improve Access but Requires New Models 16
3.1.6 The MA Model is a Collaborative Care Approach to PC for OUDs 16
3.1.7 The MA Model Improves Not Only Access but Quality 17
3.1.8 Dissemination and Implementation of the MA Model to Date 17
3.1.9 Selection of the MA Model as the Intervention for the PROUD Trial 17
3.1.10 Why Study the MA Model in a Randomized Controlled Trial? 18
3.1.11 Addressing the Needs of Clinical Leaders 18
3.2 Rationale for Pragmatic Trial Comparing the MA Model to Usual PC 19
3.2.1 Can the MA Model Increase Access to High Quality OUD Treatment? 19
3.2.2 Can the MA Model of collaborative care improve patient outcomes? 19
3.2.3 Hybrid Effectiveness-Implementation Trial 19
3.3 Conceptual Model for Successful, Sustained Implementation 20
3.4 Factors that Might Modify the Impact of the MA Model 22
3.4.1 The Role of Gender 22
3.4.2 The Role of Race/Ethnicity, Age and Socioeconomic Status 22
3.4.3 The Role of Mental Health and Medical Comorbidity 23
3.5 Sustainability of the MA Model 23
3.6 Preliminary Studies – PROUD Phase 1 24
3.6.1 Phase 1 Health Care Systems (“Sites”) 24

3.6.2 Phase 1 Site Engagement 24
3.6.3 Specification of "MA Model Care" and the PROUD Intervention 25
3.6.4 Phase 1 Impact on Design Considerations 25
4.0 OBJECTIVES 28
4.1 Primary Objective 28
4.2 Secondary Objective 28
4.3 Other Secondary Objectives 28
4.3.1 PROUD Intervention vs. Other "Exemplar" Models of OUD Care 28
4.3.2 Differences in Impact of PROUD across Age, Sex and Race/Ethnicity 28
4.3.3 Evaluate Secondary Outcomes 28
4.3.4 Identify Barriers and Facilitators to PROUD Implementation 29
4.4 Planned Ancillary Studies 29
5.0 STUDY DESIGN 30
5.1 Overview of Study Design 30
5.2 Study Duration and Activities During Each Period of the Study 30
5.3 Overview of Randomization and the Intervention 31
5.4 Overview of Data Collection 31
5.4.1 Quantitative Secondary Data Obtained from Health Systems 31
5.4.2 Qualitative Data Collected 32
6.0 STUDY POPULATION 34
6.1 Primary "Visit-based" Samples 34
6.2 PROUD Trial Sample 34
6.3 Exemplar Clinic Sample 35
6.4 Non-randomized UPC Clinic Sample 35
6.5 Secondary "Enrolled" Samples (2 Health Systems Only) 36
7.0 TREATMENT CONDITIONS 37
7.1 Randomized Clinics 37
7.1.1 PROUD Intervention: Implementation of the MA Model 37
7.1.2 Usual Primary Care (UPC) 40
7.2 Non-Randomized Usual PC Clinics 40
7.3 Non-Randomized Exemplar PC Clinics 40
8.0 MEASURES 41
8.1 Primary Objective Outcome Measure: Patient Days of OUD Treatment 41
8.2 Secondary Objective Outcome Measures: Acute Care Utilization 42
8.2.1 Patients with Recognized OUDs 44
8.3 Additional Quantitative Outcome Measures 44
8.3.1 Outcomes for Comparing PROUD Intervention and Exemplar Clinics 44
8.3.2 Outcomes Reflecting Implementation, Patient Outcomes and Care Processes 44

CONFIDENTIAL

8.3.3 Implementation Fidelity Measures (PROUD Intervention Clinics Only) 44
8.4 Covariates and Measures for Describing Study Sample 44
8.4.1 Sociodemographic Measures 44
8.4.2 Measures of Medical, Mental Health and Substance Use Disorders 45
8.4.3 Comorbidity Indices 46
8.4.4 Process Measures of Possible Cross-over Between PROUD and UPC Clinics 46
8.4.5 Baseline Measures of Generalizability of PROUD Trial Clinics 46
8.4.6 Mortality 47
8.5 Qualitative Measures 47
8.5.1 Description of the 12 PROUD Trial Clinics Pre-randomization 47
8.5.2 Barriers and Facilitators to OUD Treatment in PC 47
8.5.3 Plans for Sustainment 48
9.0 DETAILS OF STUDY PROCEDURES 49
9.1 Overview of Organization and Leadership 49
9.2 Study Period 1: Start-up Prior to Kickoff 50
9.2.1 Lead Node Team (LNT) Meetings Begin 50
9.2.2 All Site Meetings Begin 50
9.2.3 Administrative Start-up 50
9.2.4 Implementation Monitoring Start-up 50
9.2.5 Data & Analytics (D&A) Team – Preliminary Analyses and SAP 50
9.2.6 Publication Team 50
9.3 Study Period 2: PROUD Intervention Implementation 51
9.3.1 Kickoff Meeting in Seattle 51
9.3.2 Site Teams 51
9.3.3 Technical Assistance Team 51
9.3.4 Implementation Monitoring Team 51
9.3.5 Data & Analytics Team 52
9.4 Study Period 3: Ongoing Technical Assistance and Data Collection 52
9.4.1 Technical Assistance Team 52
9.4.2 Implementation Monitoring Team 52
9.4.3 Data & Analytics (D&A) Team 52
9.5 Study Period 4: Analyses and Dissemination 53
10.0 DESIGN CONSIDERATIONS 54
10.1 Design Considerations: Intervention and Control 54
10.1.1 Choice of the Intervention 54
10.1.2 Usual Care Controls 54
10.2 Design Considerations Regarding Study Samples 54
10.2.1 Recruited Clinics Are Not “Usual” 54

10.2.2 Imbalance in the Size of the Two Randomized Clinics at Each Site ... 55
10.2.3 Possible Cross-over Between UPC to Intervention Clinics ... 55
10.3 Design Considerations Regarding Measures ... 55
10.3.1 Primary Outcome Measure for the Primary Objective ... 55
10.3.2 No Data on Methadone Maintenance OTP for Most Sites ... 55
10.3.3 Timing of Outcomes Measurement ... 56
10.3.4 Use of EHR Orders for Buprenorphine and Injectable Naltrexone ... 56
10.3.5 Incomplete Ascertainment of Acute Care and Other Patient Outcomes ... 56
10.3.6 Incomplete Death Data ... 56
11.0 STATISTICAL ANALYSIS ... 57
11.1 Overview of Primary and Secondary Outcomes (Endpoints) ... 57
11.2 Statistical Methods for Primary and Secondary Outcomes ... 57
11.2.1 Statistical Methods for the Primary Outcome ... 57
11.2.2 Statistical Methods: Secondary Outcome - Acute Care Utilization ... 58
11.3 Other Secondary Analyses ... 60
11.3.1 Representativeness of Randomized UPC Clinics for Usual PC in the 6 Sites ... 60
11.3.2 Secondary Modelling of Primary Outcome ... 61
11.3.3 PROUD Intervention vs. Other “Exemplar” Models of OUD Care ... 61
11.3.4 Differences in Impact of PROUD on the Primary Outcome across Age, Sex and Race/Ethnicity ... 61
11.3.5 Evaluate Secondary Outcomes ... 62
11.4 Rationale for Sample Size and Statistical Power ... 62
11.4.1 Power Simulations – Primary Objective ... 62
11.4.2 Power Simulations – Secondary Objective ... 64
11.5 Hypothesis Testing ... 66
11.6 Interim Analyses ... 66
11.7 Sensitivity Analyses ... 66
11.7.1 Sensitivity Analyses for the Primary Outcome ... 66
11.7.2 Sensitivity Analyses for Main Secondary Outcome: Acute Care Utilization ... 67
11.8 Missing Data ... 68
12.0 QUALITATIVE ANALYSIS ... 69
12.1 Formative Evaluation ... 69
12.2 Barriers and Facilitators ... 69
13.0 REGULATORY COMPLIANCE AND SAFETY ... 70
13.1 Statement of Compliance ... 70
13.2 Regulatory Files ... 70
13.3 Care Implemented by Health Delivery Systems ... 70
13.4 Risks ... 70

13.5 Mitigation of Risks of Use of Secondary Data ... 70
13.5.1 Steps Taken to Protect Secondary Data ... 70
13.5.2 Benefits ... 71
13.6 Informed Consent ... 71
13.7 Confidentiality ... 72
13.8 Documented OUD Treatment in the EHR and 42 CFR Part 2 ... 73
13.8.1 Health Information Accountability and Portability Act (HIPAA) ... 73
13.9 Investigator Assurances ... 74
13.10 Financial Disclosure ... 74
13.11 Inclusion of Women and Minorities ... 74
13.12 Records Retention and Requirements ... 74
13.13 Audits ... 74
13.14 Reporting to Sponsor ... 74
13.15 Study Documentation ... 75
13.16 Data and Safety Monitoring Board ... 75
14.0 DATA MANAGEMENT ... 76
14.1 Overview ... 76
14.2 Design and Development of Quantitative Clinical and Administrative Data from Sites ... 76
14.3 Distributed Data Model ... 76
14.4 Site Responsibilities ... 77
14.5 Data Center Responsibilities ... 77
14.6 Data Transfer ... 77
14.7 Data Training ... 77
14.8 Data Quality Assurance ... 77
15.0 PUBLICATIONS AND OTHER RIGHTS ... 79
15.1 Dissemination Plan ... 79
15.2 Data Sharing ... 80
16.0 LIST OF APPENDICES ... 81
17.0 REFERENCES ... 82

CONFIDENTIAL

A. PROUD PROTOCOL MODIFICATIONS

Modification #1 (Date: 3/20/2018)

Original protocol: The protocol noted (e.g., in the Synopsis) that the PROUD Intervention includes 3 strategies including providing "funding for at least 1.0 full time equivalent nurse care manager" and "3 primary care providers" would get DEA waivers.

Modification:

- The study will only provide the salary for a 1.0 nurse care manager (NCM).
- And the study allows for at least 3 providers to obtain DEA waivers.

Rationale: The study is not providing more than a 1.0 salary for a NCM. If a health system needs to hire more than one NCM the 1.0 salary needs to be split among these NCMs. And the study requires at least 3 DEA waivered prescribers in the intervention clinic(s) and health systems can increase the number of waivered prescribers above 3 as they see fit.

Modification #2 (Date: 3/20/2018)

Original protocol: The protocol described the sample and data collection periods as the same (5 years).

Modification:

- Patients who visited the trial clinics over 5 years are eligible.
- Data collection occurs over 6 years, including the period of sample eligibility and also data collection for 1 year prior to the earliest visit.
- Montefiore will have a shorter sample period prior to randomization.

Data will be collected on patients who make visit to a study primary care clinic during the 5 years of study eligibility, which includes the 3 years prior to and the 2 years post randomization. For eligible patients, quantitative data will be collected from 4 years prior to through 2 years post randomization.

Montefiore will have a shorter sample period than the rest of the health systems because they transitioned to Epic in May 2015 and we will not use data from their previous EHR system. Their sample period will be at least 2 years prior to randomization, with the standard 2 years after randomization.

Rationale: The additional year prior to randomization is added to allow for data collection of covariates for the 1 year period before each patient's first visit in primary care.

CONFIDENTIAL

Modification #3 (Date: 3/20/2018)

Original protocol: No exclusion criteria.

Modification: Patients who have requested through their health systems to opt out of research will be excluded from this study.

Rationale: This necessary exclusion was accidentally omitted at baseline.

Modification #4 (Date: 3/20/2018)

Original protocol: The original protocol stated that the study did not include any baseline interviews or surveys of PC staff.

Modification: We are asking to add an anonymous 1 page survey of clinic staff about their clinic to support an ancillary study of barriers and facilitators of implementation. The survey is being added to the protocol as Appendix E.

Rationale: Clinic environment is hypothesized to potentially impact the success of implementation of collaborative care in the PROUD trial. A future ancillary protocol, described in the original PROUD protocol, will assess the association between barriers and facilitators and the success of implementation of the MA model of collaborative care in PROUD clinics (A. Campbell PhD PI). After discussions with site PIs indicating that a 1 page survey might be feasible, a survey was designed in collaboration with Dr. Campbell to provide critical baseline information for that study.

- **Anonymous staff survey**: The survey will capture clinic characteristics in key domains (see Intervention-Organizational Perspectives, Recipients-Organizational Characteristics, Implementation and Sustainability domains within the PRISM Model). The survey is comprised of items reflecting three PRISM Model domains and includes: acceptability, feasibility, and appropriateness of the intervention, commitment to change, valence (or capacity to affect change), and social norms. Items are selected from several implementation and organizational change assessments.[1-4]. The measure does not capture any identifying information and completion is voluntary.
- **Data collection:** The Clinic Staff Survey will be administered to the staff at willing randomized clinics (approximately at the time of randomization or as close as possible thereafter). Any staff that have patient contact will be invited to complete the 11-item measure (e.g., physicians, nurses, medical technicians, behavioral health, front desk). Each site will be provided two $25 gift cards or equivalent gifts to give out as a raffle at each PC clinic to incentivize participation. Site PI/PM and clinic operations leaders will select the optimal setting in which to ask staff to complete the survey (e.g., staff meetings or other all staff activity). Surveys will not be reviewed by study staff until 2 years after randomization to avoid biasing qualitative data collection. At that time it will be made available to Dr. Campbell

References

1. Holt DT. (2007). Readiness for Organizational Change: The systematic development of a scale. The Journal of Applied Behavioral Science, 43(2), 232-255.
2. Shea CM, Jacobs SR, Esserman DA, Bruce K, Weiner BJ. (2014). Organizational readiness for implementing change: a psychometric assessment of a new measure. Implementation Science, 9, 7.

3. Solberg LI, Asche SE, Margolis KL, Whitebird RR. (2008). Measuring an organization's ability to manage change: The Change Process Capability Questionnaire and its use for improving depression care. American Journal of Medical Quality, 23(3), 193-200.
4. Weiner BJ, Lewis CC, Stanick C, Powell BJ, Dorsey CN, Clary AS, Boynton MH, Halko H. (2017). Psychometric assessment of three newly developed implementation outcome measures. Implementation Science, 12, 108.

Modification #5 (Date: 3/20/2018)

Original protocol: The original Table 7 of "Additional Outcomes" was not explicit about clinic-level vs. patient level measures. Several measures were also felt to be somewhat unclear in the original protocol. In addition, this Table of additional outcomes did not distinguish between secondary outcomes (piloted in Phase 1) and exploratory outcomes.

Modification: This modification is to update the Table 7 additional outcome measures per the tracked changes below. Exploratory measures are now noted with an asterisk.

Table 7. Additional Outcomes: Assessed in the 2 Years After Randomization.

**Additional Implementation Outcomes**	
**Newly recognized OUDs** (Reach)	Clinic-level number of patients with a new ICD code for OUD documented in the EHR during the period from randomization until two years after who did not have an OUD diagnosis documented in the EHR in the three years prior to randomization, reported per 10,000 primary care patients in the clinic in the 2 years post-randomization. Number of patients* with a new ICD code for OUD during follow-up who did not have an OUD diagnosis in the three years prior to randomization
**Initiation of OUD treatment** (Reach)	Clinic-level number of patients who initiate (1) buprenorphine or (2) injectable naltrexone with an OUD diagnosis as documented in the EHR during the period from randomization until two years after, reported per 10,000 primary care patients in the clinic in the 2 years post-randomization. Measure will be calculated for any initiation and separately for initiation of each type of medication. Number of patients* who initiate buprenorphine or injectable naltrexone with an OUD diagnosis (> 28 days) during follow-up: any initiation and separately for initiation of each type of medication.
**Retention in OUD treatment** (Implementation effectiveness)	Clinic-level number of patients initiating OUD treatment during the period from randomization until two years after randomization as documented in the EHR, who also receive OUD treatment on 80% of days available after initiation, reported per 10,000 primary care patients in the clinic in the 2 years post-randomization. Number of patients* initiating OUD treatment during follow-up who receive OUD treatment on 80% of days available after initiation and median days of OUD treatment after initiation for patients with at least 6 and 12 months available for follow-up. (Patients not retained in treatment per the EHR are considered no longer in treatment, an added secondary outcome).
**Contiguous days in treatment*** (Implementation effectiveness)	Days of OUD medication treatment with no gap in orders or refills exceeding 60 days[73] will be used to assess retention as well

CONFIDENTIAL

**Cross-over between clinic arms***	The number of patients with OUDs assigned to each clinic (PROUD and UPC) in the 3 years prior to randomization who are seen in the other clinic post randomization,
**Cross-over between systems*** (patients with OUD WA only)	The proportion of patients treated for OUDs in each clinic who have insurance from another health system (i.e., patients seen at Multicare who have KP insurance and patients seen in KP Washington who have outside insurance).
**Prescribing providers*** (Adoption)	Number and % of PC providers who order buprenorphine or injectable naltrexone for at least 2 patients with OUDs
**OUD treatment starts per week*** (Implementation fidelity)	Mean number of patients initiating buprenorphine or injectable naltrexone per week over the 2 years post randomization
**Time to OUD treatment*** (Implementation fidelity)	Median number of days (0 days if same day to infinity if untreated) from first visit with a new OUD ICD diagnosis (no prior diagnosis) to OUD treatment initiation during follow-up
**Urine drug monitoring*** (Implementation fidelity)	Median frequency of urine drug testing in the 1 and 3 months post initiation of OUD medication treatment. (Note: Although this aspect of the MA Model is specified locally because there is no scientific consensus on the optimal algorithm, urine monitoring early in treatment is recommended)
**Re-initiation of OUD treatment*** (Implementation fidelity)	Proportion of patients with prior EHR documentation of OUD treatment followed by at least a 3-month gap in treatment, who re-initiate buprenorphine or injectable naltrexone
**Methadone OTP***	Proportion of patients who are receiving methadone maintenance (restricted to 3 sites with OTP data)
**Buprenorphine dose***	Highest mean daily buprenorphine dose in any month of buprenorphine treatment (patient-level measure)
**Additional Patient Outcome Measures**	
**Urgent care or ED use** (Secondary patient outcome)	Patient-level number of visits to urgent care or EDs during the period from randomization until two years after, among patients with an OUD diagnosis documented in the EHR in the three years prior to randomization. ~~Number of visits to urgent care or EDs during follow-up among patients with an OUD diagnosis~~
**Inpatient Days hospitalized** (Secondary patient outcome)	Patient-level number of days hospitalized during the period from randomization until two years after, among patients with an OUD diagnosis documented in the EHR in the three years prior to randomization. ~~Number of days hospitalized during follow-up, among patients with an OUD diagnosis~~
**Opioid overdose*** (Secondary patient outcome)	Proportion of patients with an ICD code for opioid overdose during follow-up (Note this is expected to be biased due to improved ascertainment in the PROUD clinics due to NCM follow-up with patients with OUDs).
**HCV viral cure*** (Secondary patient outcome)	Number of patients with HCV who have documented viral suppression among all with diagnosed chronic active HCV.
**Additional Outcome Measures - Other Processes of Care**	
**New diagnosis or treatment of other substance use condition***	Number of patients who have a new diagnosis or treatment for another substance use condition (not diagnosed prior 2 years)

**New diagnosis or treatment of mental health conditions***	Number of patients who have a new diagnosis or treatment for a mental health condition (not diagnosed prior 2 years)
**Naloxone prescribing**	Patient-level number of prescriptions of naloxone for overdose management in the period from randomization until two years after, among patients with an OUD diagnoses in the three years prior to randomization. ~~Number of prescriptions of naloxone for OD management among patients with OUD diagnoses during follow-up~~
**HCV & HIV Screening or treatment***	Number of patients with new OUD diagnoses who have screening tests or treatment for HCV or HIV

Not included in ClinicalTrials.Gov since they are exploratory and not tested in Phase 1 therefore inadequate validation prior to the trial.* ~~ Measured in the 2 years after randomization using EHR data, reported per 10,000 PC patients in a clinic in the 3 years prior to randomization.*~~

Rationale: Clarifications were noted to be needed when measures were prepared for ClinicalTrials.gov. We are updating additional outcome measures in Table 7 to align with our published ClinicalTrials.Gov registration, as well as clarify clinic vs. patient-level measures, and other clarifications above. Finally, the modifications distinguish secondary outcomes from exploratory outcomes with an asterisk.

Modification #6 (Date: 3/20/2018)

Original protocol: The protocol was inconsistent regarding reporting of overdoses to the DSMB.

Modification: We plan to measure ODs and will report those data to the DSMB for tracking purposes.

Rationale: This modification is only to clarify the protocol regarding reporting overdoses (ODs) since this was not listed in all places.

Modification #7 (Date: 3/20/2018)

Original protocol: The protocol was inconsistent, including the estimated sample size (170,000) based on Phase 1 vs. the estimate prior to Phase 1 (120,000).

Modification: The estimated sample size based on Phase 1 is about 170,000 patients in the 12 randomized primary care clinics.

Rationale: We are revising for consistency and to update all estimates to be consistent.

Modification #8 (Date: 7/26/2019)

Original protocol: The original intervention includes 3 strategies (as noted in Section 2.4 and Modification #1):

1. The study will provide the salary for a 1.0 nurse care manager (NCM) for 2 years after randomization and technical assistance (TA) for recruiting and hiring the NCM. Once hired for the study, the NCM will receive TA from experts in Massachusetts (MA) supported by PROUD, but NCMs will be employed and supervised by the health system.
2. Experts at Boston Medical Center (BMC) who originally developed and disseminated the MA Model will: provide the intervention clinics with a MA Model Manual; train PROUD NCMs at

BMC for 2 days; and provide ongoing TA for 2 years after randomization. This is described in Appendix A.

3. At least 3 PC providers in the PROUD intervention clinic obtain DEA waivers to prescribe buprenorphine for opioid use disorders (OUDs), if not already waivered, and work closely with the NCM to offer high quality care for OUD.

Modification: This modification adds optional weekly quality improvement meetings in intervention clinics. Specifically, we would ask site lead investigators and project managers to arrange quality improvement meetings with the intervention clinics with the goal of increasing the number of patients with OUDs being treated by the clinic. The recommended elements of the quality improvement meetings include:

1. The site lead investigator(s) and project manager will work with clinic leaders to identify an interdisciplinary quality improvement team (QIT) consisting of: interdisciplinary champions from the PROUD intervention clinic (medical assistants, PCP waivered prescribers, front desk staff, etc.), the NCM, and themselves. Clinical leaders who originally supported the study may also be included.
2. The QIT is recommended to meet weekly, ideally for an hour, but at a minimum of 30 minutes to review quality improvement activities from the past week and plan quality improvement activities for the next week using the plan-do-check-adjust (PDCA) cycle approach in which small tests of change are completed by planning activities each week, reviewing data and activities at the following meeting, and iterating and/or expanding activities based on results and lessons learned. Each QIT is recommended to have a facilitator and note taker.
3. The note taker will send an email summarizing actions taken, results, and planned actions to the TA Team after each meeting (ccing the PROUD study email box). The TA team will—as time allows at their weekly TA meetings with nurses—ask nurses to share experiences with the QIT, highlighting successes and lessons learned.

Rationale: As of early July 2019, the PROUD NCMs at each of the intervention clinics are caring for 0-34 patients. Our goal has been to add 1-2 new patients a week per site and this is not currently occurring consistently. The PROUD Operations Team has decided that more structure and support is needed to successfully integrate the NCMs into primary care and increase the number of patients working with the NCMs. Holding weekly quality improvement meetings to support the NCM is an evidence-based approach that we expect will help the sites identify appropriate ways within their clinic to increase the number of new patients coming to the clinic.

Modification #9 (Date: 10/10/2019)

Original protocol: The protocol currently says that the participating health systems will complete qualitative stakeholder interviews with all their randomized intervention and usual care clinics quarterly and then debrief these interviews with the Implementation Monitoring Team who will monitor changes over time.

Modification: This modification is to request that the frequency of stakeholder interviews be changed to every 6 months (instead of quarterly).

Rationale: We are requesting to change the frequency of these stakeholder interviews and debriefs because the Implementation Monitoring Team found that conducting quarterly debriefs was not feasible due to the complexity of scheduling with the health systems.

CONFIDENTIAL

Modification #10 (Date: 10/10/2019)

Original protocol: The original power calculations for the primary outcome measures contained a typo in the simulation program and thus the reported results in prior versions of the protocol are incorrect.

Modification: The corrected power estimates reported in Table 13 have been regenerated after correcting the simulation code. The updated results are:

Table 13. Power Results for a 0.05-level One-Tailed Test, Based on 10,000 Iterations Per Cell

	Model	
**k-fold Increase in Primary Outcome (Treatment Effect)**	**No Adjustment for Baseline**	**Inclusion of Baseline as a Covariate**
1.00	5%	5%
1.06	9%	13%
1.12	15%	29%
1.18	21%	49%
1.24	30%	68%
1.30	39%	84%
1.36	50%	93%
1.42	60%	98%

Based on Table 13, there is at least 80% power to detect an 30% increase in the number of OUD-treated days per patient seen. Thus, with two clinics in each of six health care systems, the study is sufficiently powered to detect the targeted 5-fold increase in the primary outcome measure. As anticipated, there is a substantial gain in power when the baseline value is included as a covariate in the primary outcome model.

Rationale: We are revising the reported power estimates so that we are reporting results based on correct simulation code.

Modification #11 (Date: 10/10/2019)

Original protocol: The text of the original Protocol for the Objective 2 statistical analysis did not state that we would apply a small-sample correction method (Section 11.2.2), nor was such an approach applied within the power calculations. Failure to use a small-sample correction method when there are a small number of clusters can result in a type 1 error rate that is inconsistent with the nominal 0.05 level.

Additionally, the power calculations for the original protocol made an incorrect assumption for modeling the within-clinic correlation (i.e., they did not simulate clinic-level random effects), which is expected to have over-estimated power.

Modification:
We updated the Protocol for the Objective 2 primary analysis to address recent recommendations that cluster-randomized trials with a small number of clusters apply a small-sample correction method when testing for intervention effects. To address these recommendations, we are modifying the Protocol to say that "We plan to apply a small-sample degree of freedom correction approach, such as the Between-Within (BW) method for the Wald F test (Li and Redden 2015), with the final approach to be determined in the standalone statistical analysis plan (SAP)."

Additionally, we are modifying the power simulations to incorporate a small-sample degree of freedom correction method, while appropriately accounting for the within-cluster correlation. Specifically, we now present the power and type 1 error rates for both the large-sample Wald test, as well as the Wald F test with the BW correction method. We also now present power across different assumptions for the within-clinic correlation (parameterized by the standard deviation [SD] of the clinic-level random intercept). While modifying the power calculations to address these issues, at the same time we also updated the presentation of the results of power calculations to show the power across a range of effect sizes, parameterized by the relative risk of acute care utilization comparing patients in PROUD intervention clinics versus patients in usual primary care (UPC) clinics. Original calculations did not show the power across different possible effect sizes, but rather across the proportion of patients treated by the nurse care manager (NCM) in the PROUD intervention clinics, assuming a constant effect size among patients treated by the NCM. The modification below show power across a range of effect sizes and proportion of patients treated by the NCM.

Below are the revised sections 11.2.2 and 11.4.2 with track changes showing where it was modified

Section 11.2.2 Revised

Statistical Methods: Secondary Outcome - Acute Care Utilization

Detailed analytic specifications for the secondary outcomes identified in Section 8.2 will be developed during start-up and detailed in a separate SAP and finalized before final data lock. Below are brief descriptions of the proposed approaches.

We will evaluate, among individuals who have an OUD diagnosis, whether acute care utilization differs among patients from PROUD intervention clinics as compared to patients from UPC clinics (acute care utilization defined above in Measures Section 8.2). Our primary analysis will focus on patients who were identified as having an OUD prior to randomization. Because the PROUD intervention is expected to increase diagnosis of OUD, those patients diagnosed post-randomization in the PROUD intervention clinic are likely to be different than those diagnosed prior to implementation and could therefore lead to biased estimates of the treatment effect if included in the analysis. Our current plan will be to exclude those diagnosed with OUD after randomization from the primary Objective 2 analysis. Secondary Objective 2 analyses (described below) will include those individuals who were not identified until post-randomization and will incorporate analytic methods for observational data.

Primary Analysis of Objective 2

We hypothesize that, among patients who had a PC visit and were identified (pre-randomization) as having an OUD diagnosis (documented in their EHRs in the 3 years prior to randomization), individuals from a PROUD intervention clinic will have decreased acute care utilization after randomization as compared to individuals from a UPC clinic. We plan to fit a mixed-effect Poisson regression model (with log link) at the patient level to the number

of days of acute care utilization. The model will account for clustering of patients within a clinic by including clinic-specific random intercepts. Specifically, the regression model will be of the following form:

$$log[E(y_{ijk})] = \alpha + \beta * trt_{ij} + \gamma * z_{ijk} + \theta_{ij}$$

where

- y_{ijk} is the observed number of days of acute care utilization of patient k in clinic i of site j
- trt_{ij} is the treatment indicator (PROUD intervention) for clinic i in site j
- z_{ijk} is a vector of clinic and/or patient-level covariates (γ is the corresponding coefficient)
- θ_{ij} is the random effect for clinic i in site j

We plan to adjust for a parsimonious list of pre-specified, baseline covariates that are known to be strongly associated with the outcome from the literature such as age, gender, race/ethnicity at baseline,[73] comorbidity and utilization prior to randomization, to be specified in the stand-alone SAP. Because one of the HCSs randomized at a later date than the others, not all HCSs have the same amount of follow-up time. To account for this possible difference, we may include in the model an offset term for the number of days of potential follow-up time (e.g., 2 years for 5 of the HCSs and 1.5 years for the 6th HCS). We will evaluate our primary Objective 2 hypothesis by testing the null hypothesis $H_0: \beta = 0$ versus the two-sided alternative hypothesis that β is non-zero with a type 1 error rate of 0.05. We plan to apply a small-sample degree of freedom correction approach, such as the Between-Within (BW) method for the Wald F test (Li and Redden 2015), with the final approach to be determined in the standalone statistical analysis plan (SAP). A small-sample correction is necessary to obtain correct type 1 error rates in cluster-randomized trials with a small number of clusters (Kahan et al. 2016; Leyrat et al. 2017). As a sensitivity analysis, we will consider also including in the model any additional covariates found to differ between individuals with a prior OUD diagnosis in the PROUD intervention clinics as compared to the UPC clinics pre-randomization, as well as patient-level variables found to be associated with acute care utilization among patients with OUDs.

Section 11.4.2 Revised

Power Simulations – Secondary Objective

We investigated the power of the primary Objective 2 analysis via Monte Carlo simulation. ~~Among individuals in the intervention clinic with an EHR-documented OUD diagnosis pre-randomization, not all will visit the PROUD NCM and receive treatment with buprenorphine or injectable naltrexone (hereafter "treated for OUDs"). We explored how the power is affected by the proportion of patients with a prior OUD diagnosis who are treated for OUDs (p_trt). Based on the table below, and our expectation that the PROUD clinics will treat over 15% of patients with OUDs, we expect to have adequate power for our secondary objective.~~ We assumed the following sample sizes for the number of patients with a prior OUD diagnosis over a 3-year period from the Phase 1 data, reflecting the 3-year baseline period of PROUD during which patients with an OUD diagnosis will be identified:

site_id	clin_num	clin	nOUD
A	1	A1	9
A	2	A2	12
C	1	C1	63
C	2	C2	39
E	1	E1	58
E	2	E2	200
I	1	I1	100
I	2	I2	49
J	1	J1	27
J	2	J2	10
K	1	K1	388
K	2	K2	290

We generated individual-level outcome data within each of the 12 clinics as follows. First, we randomly assigned one of the two clinics within a HCS to receive the PROUD intervention and the other to the Usual Primary Care (UPC) group. ~~Then, for each patient from a PROUD intervention clinic with a prior OUD diagnosis, we identified whether that patient was treated for OUDs by the nurse (with probability p_trt). Of the patients treated for OUDs by the nurse, we assumed that the probability that they are persistently treated is 50%. Among patients who are able to be treated for OUDs and are persistently treated, w~~We then generated outcome data from a Poisson distribution with mean number of acute care days over a two-year period (time-frame of PROUD outcome ascertainment) using the following mean model,

$$\log E\left(y_{ijk}\right) = \alpha + \beta * trt_{ij} + \theta_{ij}$$

where

- y_{ijk} is the number of days of acute care utilization of patient k in clinic i of site j
- trt_{ij} is the treatment indicator (PROUD intervention versus UPC) for clinic i in site j
- $\theta_{ij} \sim N(0, \tau^2)$ is the random effect for clinic i in site j (τ is the standard deviation of the clinic-level random effect)

For the parameter α we assumed that the baseline rate of acute care utilization over a two-year period for patients assigned to a UPC clinic was equal to the average number of acute care visits among patients with a prior OUD diagnosis obtained from Phase 1 data (=4.0 visits) multiplied by the average number of days per acute care visit. The average number of days per acute care visit was based on KPWA data on the average length of stay among all patients (since length of stay data was not available from all sites at Phase 1), which was 2.04 days per visit. That is we assumed $\alpha = \log(4 * 2.04) = 2.1$. We considered a range of values for the intent-to-treat relative risk parameter $RR_{ITT} = \exp(\beta)$ governing the association between being assigned to the PROUD intervention and acute care utilization over the follow-up period. Finally, we considered three different values for the standard deviation τ of the clinic-level random effect θ_{ij}. Specifically, we estimated a value for τ using Phase 1 data of $\tau = 0.23$,

and also considered two values as a sensitivity analysis: one that was 50% smaller ($\tau = 0.12$) as well as one that was 50% larger ($\tau = 0.35$). We estimated power and type 1 error based on the standard Wald test, as well as the Wald F test that used a denominator degree of freedom based on the Between-Within (BW) small sample degree of freedom correction. For testing the coefficient β from the above model, the BW method uses as denominator degree of freedom (10 = 12 clinics - 2 fixed effect parameters being estimated). Results are based off of 1,000 simulation repetitions.

In addition to presenting power across values of RR_{ITT}, we also provide additional context in light of the fact that not all Among individuals in the intervention clinic with an EHR documented OUD diagnosis pre-randomization, not all will visit the PROUD NCM and receive sustained treatment with buprenorphine or injectable naltrexone (hereafter "treated for OUDs"), which is hypothesized to meaningfully reduce acute care utilization (36). Specifically, for different values of RR_{ITT}, we report the proportion of patients who would need to be treated for OUDs (denoted by p_{treat}), if the relative risk of acute care utilization comparing patients who are treated for OUDs versus patients who are not treated for OUDs ($RR_{treated}$) is 0.1 or 0.2. A value of 0.2 corresponds approximately to the observed RR of acute care visits comparing those without OUD to those with OUD; a value of 0.1 corresponds to the assumption that those who are treated for OUDs will have a 50% decrease in the average visit length compared to those with OUD who are not treated. These calculations assume that patients with OUD in a PROUD intervention clinic who are not treated for OUDs have the same rate of acute care utilization as patients with OUD in UPC clinics.Note that these initial power calculations were not based on simulating clinic-level random effects. In another set of simulations that did generate clinic-level random effects, the type I error rates were no longer accurate. This is because under the small number of clinics per treatment arm (intervention vs. usual care), generating outcome data in this way yielded imbalance in the mean number of acute care days in the intervention versus usual care arm. Imbalance in the rates of acute care across treatment arms is a concern; as described in our analysis plan above, we plan to adjust for baseline utilization along with other covariates that could account for differential utilization across clinics.

Results of Power Simulations

The following plot and table show the power across different values for the proportion of patients within PROUD clinics who are treated for OUDs (p_trt). We see that power was >0.90 in scenarios where more than 20% of patients with recognized OUDs are treated for OUDs (p_trt > 0.20).

The following table shows the type 1 error rates for each of the 3 values of clinic-level random effect SD (τ) using the naïve Wald test ("Wald" below), as well as the Wald F test based on the BW degree of freedom correction (BW below):

	τ	**Wald**	**BW**
Sensitivity	0.12	0.106	0.075
**Primary**	0.23	0.104	0.070
Sensitivity	0.35	0.111	0.067

Although the type 1 error rates using the BW method (0.067-0.075) are still slightly elevated over the nominal 0.05 level, they are much closer to the correct level as compared to the

standard Wald test (all > 0.1). We will continue to explore whether this can be improved further as the SAP continues to be developed.

The power (or type 1 error rates for $RR_{ITT} = 1$) across different effect sizes (parameterized by RR_{ITT}) and different values of the random-effect SD τ (0.12, 0.23, and 0.35) is given in the following figure:

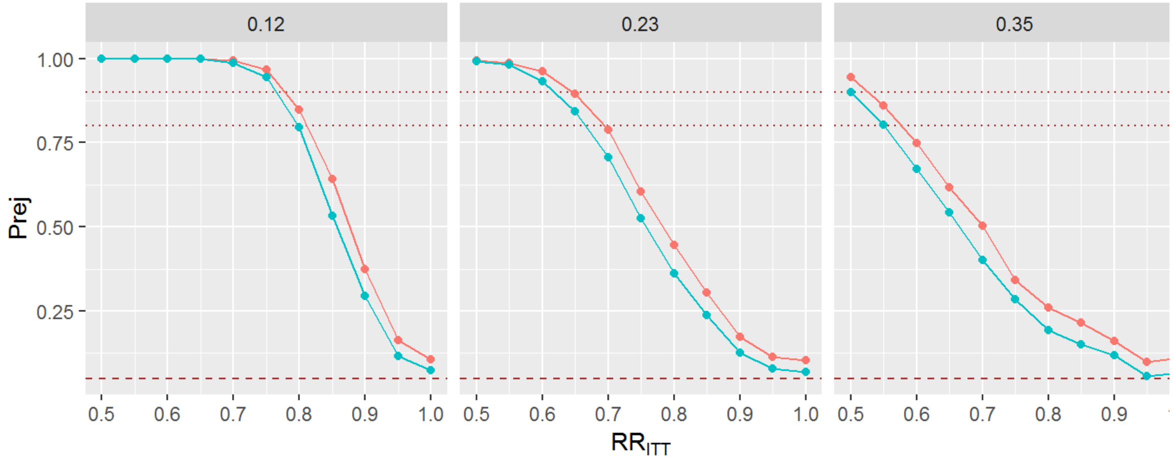

Here 'Prej' denotes the proportion of Monte Carlo iterations for which the null hypothesis was rejected; each panel corresponds to a different value of the random-effect SD (τ). Based on using the BW degree of freedom correction approach, we estimated that we have >80% power to detect values of $RR_{ITT} \leq 0.65$ when $\tau = 0.23$, corresponding to a 35% reduction in the acute care utilization rate among patients in a PROUD versus UPC clinic. Similarly, we have >80% power to detect values of $RR_{ITT} \leq 0.80$ when $\tau = 0.12$ and of $RR_{ITT} \leq 0.55$ when $\tau = 0.35$.

We next provide additional context on the corresponding proportion of patients in PROUD intervention clinics who would need to be treated for OUDs (p_{treat}) in order to detect the above values of RR_{ITT} when the underlying relative risk of acute care utilization comparing patients treated for OUDs versus those who are not treated ($RR_{treated}$) is 0.1 or 0.2.

	τ	RR_{ITT}	**Proportion of patients needing to be treated for OUD (p_{treat})**	
			RR among treated ($RR_{treated}$)=	
			0.1	0.2
Sensitivity	0.12	≤ 0.80	≥22%	≥25%
**Primary**	0.23	≤ 0.65	≥39%	≥44%
Sensitivity	0.35	≤ 0.55	≥50%	≥56%

Thus, under our primary assumption for the random-effect SD (τ) if at least 39-44% of patients with OUD in the PROUD intervention arm at baseline are treated for OUD by the NCM, then we will have over 80% power to detect at least a 35% decrease in acute care utilization ($RR_{ITT} \leq 0.65$) comparing patients with OUD in the PROUD intervention arm versus UPC, when the true RR comparing treated to untreated patients with OUD ($RR_{treated}$) is 0.1-0.2. If fewer patients are treated, then our power would be less than 80% under these same assumptions.

CONFIDENTIAL

Rationale: These updates to Objective 2 primary analysis will address recent recommendations that cluster-randomized trials with a small number of clusters apply a small-sample correction method when testing for intervention effects. The modification of the power simulations now incorporate a small-sample degree of freedom correction method, while appropriately accounting for the within-cluster correlation. Original calculations did not show the power across different possible effect sizes, but rather across the proportion of patients treated by the nurse care manager (NCM) in the PROUD intervention clinics, assuming a constant effect size among patients treated by the NCM. The modification show power across a range of effect sizes and proportion of patients treated by the NCM.

Modification #12 (Date: 10/10/2019)

Original protocol: Section 4.4 of the protocol indicates that one of three planned ancillary studies would be a study of the cost benefit of the intervention.

Modification: The planned cost benefit ancillary study was recently funded; therefore, we are modifying the main protocol to add the fully developed plans for this study. Attached as Appendix F is the PROUD Trial Economics Analysis.

Rationale: This ancillary study will leverage the existing research infrastructure and data cohort of the main PROUD Trial. It will allow us to answer questions of economic viability of the PROUD Trial, that is, from the perspective of the healthcare sector, to what extent do the downstream cost savings associated with improved patient outcomes offset the additional costs of the PROUD intervention.

Clarification #13 (Date: 10/10/2019)

Original protocol: Appendix B is a version of the Boston Medical Center OBAT Policy and Procedures Manual, last updated on May 10, 2017, and was attached as a separate document to the original protocol.

Clarification: We would like to clarify and emphasize that the Boston Medical Center OBAT Policy and Procedures Manual is a living document and is updated frequently by the Technical Assistance Team, so that the manual stays up to date with current standards of care. We would also like to clarify that, as stated in the protocol, each PROUD intervention site adapts the flexible components (such as the frequency of urine testing) to their local standards with assistance from the Technical Assistance Team. Therefore, Appendix B is an example of the OBAT manual but not the actual version of the manual being used at any particular time or site.

Rationale: It is not feasible to update the manual with the IRB each time the Technical Assistance Team updates it to stay current with current standards of care and/or each time a site makes minor adjustments. We are requesting that the manual be deemed a living document due to the multiple updates that occur over time, and others that are made by the sites.

Modification #14 (Date: 1/30/2020)

Original protocol: The original protocol does not have any chart review activity.

Modification: We are requesting to modify the protocol to add chart reviews to investigate what appear to be same day duplicate buprenorphine orders in the electronic data.

<u>Background on the need for this modification:</u> Electronic data from electronic health records (EHRs) obtained to date include a minority of patients with buprenorphine orders that appear to be duplicates. Duplicates are currently defined as medication orders with the same order date, medication name,

strength, and form. It is unknown whether these are a data artifact in need of de-duping (e.g., orders cancelled, suspended, revised, etc.) or actual unique orders (e.g., multiple unique orders written with the intent that the patient obtains some of the orders at a later date when the prior order runs out).

Methods for added chart reviews: The Data & Analytics Team will select all duplicate orders in the EHR datasets for each site (approximately 250 patients for all sites combined) and send the study IDs, order dates, medication name, days supply, quantity, and strength associated with these duplicate orders to the associated site via secure file transfer (SFT). Study Site Programmers keep a crosswalk of medical record numbers and study IDs, which they will provide to the person doing chart reviews. The Site PI, Site Project Manager, or a staff person working under their supervision will complete the chart reviews and document the chart review questions below for each duplicate identified in the EHR. The chart review questions may evolve to some extent as the research team pilots this process to ensure we are abstracting the information needed to help inform our understanding and decisions about the duplicate orders we are seeing in the EHR data. The study site will send the chart review documentation to the Data & Analytics team via SFT, ensuring they aren't including any protected health information (PHI). No information on whether patients are from the intervention clinics or usual care clinics will be provided to the sites or back to the Data & Analytics Team.

Chart Review Objectives and Questions for Abstractors:

1. Note which duplicates within a group of duplicates is a true duplicate due to a data artifact versus another explanation (e.g., multiple orders written on the same day with the intention of one or more being filled at a later date)?
2. If a true duplicate due to a data artifact, specific reason/data artifact for duplicate records:
    a. Suggestions for if and how-to de-dup this example using automated only data
3. If the duplicate is actually multiple unique orders "written" on the same day for filling at a later date, (is there evidence that these orders were sent to be filled or obtained by patients?
    a. Is there evidence that 1+ of the duplicates was for induction?
        i. Suggestions for how this could be determined using automated only data?
    b. What is the intended date for the unique orders to be filled? For example, 2 orders that appear as duplicates on 1/1/2020 but one was intended to be filled on 2/1/2020 after the first one ran out.
        i. Suggestions for how this could be determined using automated only data?
4. Abstractors will look for evidence on whether the patient has been having visits, has picked up buprenorphine, has laboratory tests indicative of buprenorphine use, and is taking buprenorphine over time to help figure out if the duplicates are actually unique orders picked up at a later date.

Rationale: Adding this chart review activity will help inform our understanding and decisions about the duplicate orders we are seeing in the secondary EHR data we are collecting and analyzing for the study. It is important that orders are correct prior to calculating our primary outcome.

Modification #15 (Date: 1/30/2020)

Original protocol: The original protocol included a 2-year intervention period.

Modification: This modification is to request to extend the original 2-year intervention period to a 3-year intervention period. We would like to extend the time the nurse care managers (NCMs) are working in the clinics implementing the PROUD intervention by 12 months with continued support by the Technical Assistance Team. There will be no changes to the main trial analyses as we will still analyze outcomes of the trial as proposed in the protocol with a 2-year intervention period. In addition, a final data extraction will be added, after the 3-year intervention period ends on 2/28/2021, to allow us to conduct secondary analyses of the primary outcome, as well as other secondary analyses.

Rationale: Recent data has revealed that the Massachusetts model of collaborative care that we are testing often takes more than 2 years to achieve steady recruitment rates of 1-2 patients a week. Therefore, NIDA decided that it was appropriate to test the PROUD intervention for 3 years instead of 2 years. Extending the intervention to 3-years will assist in seeing the full effect of the PROUD Trial intervention. In addition, limiting the trial to 2 years might lead NCMs to leave as the study ended, further undermining our ability to assess the effectiveness of the Massachusetts model.

Modification #16 (Date: 11/16/2020)
**Original protocol:** Appendix A outlines the Massachusetts (MA) Model of OUD care specific for the PROUD Trial, including the data the nurse care manager (NCM) is responsible for collecting and reporting to the Technical Assistance (TA) Team weekly. A portion of this outline is also pasted in the protocol as Table 5, and Table 8 in the protocol outlines elements in the NCM report that are sent to the TA Team weekly.
**Modification:** We would like to modify Appendix A, Table 5 and Table 8 in the protocol to align all three of these sections with the data being collected weekly by the NCMs. The NCM weekly reporting elements in these sections are slightly different than what is being collected for the trial. Below is an outline of weekly data reports being collected by the NCMs for the trial and sent to the TA Team (with the Implementation Monitoring Team (IMT) being cc'd).<br><br>• # Screened<br>• # Intakes*<br>• # New starts*<br>• # Follow-ups seen on scheduled day*<br>• # Walk-ins seen<br>• # Re-engagements seen<br>• # Naltrexone injections given<br>• # Buprenorphine injections given<br>• # Discharges and reason (screened only but never started, completed, administrative/non-adherence, incarceration, transferred to higher level of care, medical, lost to follow up, deceased, other)*<br>• # No shows<br>• # Visits rescheduled<br>• # Patients seen new to primary care clinic<br>• # New patients seen with primary care outside health system

• # Patients transferred from elsewhere already on medication*<br><br>* by oral buprenorphine, injectable buprenorphine, oral naltrexone, injectable naltrexone<br><br>Summary of differences: Appendix A, Table 5, and Table 8 indicated that we captured number of patients with unexpected positive urine drug tests and those contacted within 7 days, however those data are not collected. Appendix A and Table 5 had not mentioned that we captured information on: # no shows; most of the above bullet points are broken out by groups: oral buprenorphine, injectable buprenorphine, oral naltrexone, injectable naltrexone; number of patients transferred to NCM who were already on medication (broken out by medication); # of walk-in patients seen (broken out by medication). In addition, Appendix A and Tables 5 and 8 did not indicate that we captured data on patients being treated with injectable buprenorphine and oral naltrexone.
**Rationale:** As the trial launched and the NCMs began reporting data, the reporting elements for the NCM weekly report were adjusted slightly to ensure the most relevant data were being collected in relation to the standards of care for treating OUDs in primary care. These adjustments were made as a part of the TA Team process, supported by formative evaluation between the TA Team and the IMT.

Modification #17 (Date: 11/16/2020)
**Original protocol:** Appendix A and Table 5 in the protocol originally noted that we would provide intervention clinics performance feedback on the implementation of the MA Model with a study-supported dashboard with all sites' performance by month based on weekly data provided to the Technical Assistance (TA) Team from the nurse care managers (NCMs).
**Modification:** We are requesting to modify Appendix A and Table 5 in the protocol to update how performance monitoring is implemented for the PROUD Trial. Originally, we were planning to provide clinics feedback each month on a study-supported dashboard, with all sites' performance based on weekly data provided to the TA Team from the NCMs. Although we initially shared all-site performance data, sharing was changed at the recommendation of the TA Team. The TA team noted that sharing cross-site performance data was not beneficial to NCM morale, given each site's uniqueness and challenges with implementing the intervention. Therefore, each site now only receives performance reports on their site. Each week, a summary of the site's performance is emailed to just the site PI(s), project manager(s) and NCM. The PROUD Trial Operations Team, Implementation Monitoring Team, and TA Team, however, all get emails weekly with all sites' performance.
**Rationale:** Due to each site's unique challenges in implementing the PROUD intervention, it was decided by the TA Team, who is in weekly contact with the NCMs, that it was not good for morale to have all site data shared with all sites. Rather it would be more beneficial for sites to just see their own data weekly to assist with monitoring and supporting the program.

Modification #18 (Date: 11/16/2020)

Original protocol: Section 3.2 of the Economic Analysis (Appendix F) indicates that the project will estimate costs for implementing the PROUD intervention by primarily using a macro-costing "top down" approach based on financial information received by the PROUD study team and from the PROUD clinics. And that these data will be supplemented with information collected by the PROUD Implementation Monitoring Team (IMT).

Modification: We would like to modify Section 3.2 of the Economic Analysis to add sending aggregate data from the weekly reports that the nurse care managers (NCMs) at each intervention site submit to the Technical Assistance Team weekly (and cc the IMT) to the Economic Analysis Team at Weill Cornell Medical College.

Rationale: The Economic Analysis Team at Weill Cornell Medical College will use this aggregate data from the weekly NCM reports from each intervention site to assist in completing their Aim 1 analyses, which is to estimate the start-up and ongoing management costs of the PROUD intervention. Knowing the number of NCM visits at each site weekly and the other information collected in this report will assist them in understanding and calculating the costs of the PROUD intervention.

Modification #19 (Date: 11/16/2020)

Original protocol: Section 15.2 of the original protocol does not include plans for data sharing beyond the main analytic dataset.

Modification: We are requesting to modify section 15.2 of the protocol to update the data sharing plan in its entirety to be more inclusive of sharing beyond just the main analytic dataset. Below is the updated language which we are proposing would replace the original language in section 15.2.

Updated Data Sharing Plan (that replaces the existing plan in section 15.2 in its entirety):

In keeping with NIH policy on data sharing for promoting new research, encouraging further analyses and disseminating information to the community, CTN-0074 has established a data sharing policy that also protects the rights and privacy of individuals and follows the revised 42 CFR Part 2 regulation which prevents re-disclosure of 42 CFR Part 2 covered data. Therefore, all data shared by KP Washington will be de-identified.

Data Sharing with the NIDA Clinical Trials Network Data and Statistics Center (DSC): A de-identified dataset will be shared with the NIDA CTN DSC to allow the DSC to perform analyses supporting the primary objective. All dates that are provided will be masked, as will all indicators of site and clinic, which are essential elements for the analysis of the primary objective.

Additional de-identified datasets will be shared with the DSC to allow DSC statisticians to review, replicate, and test computer code written at KP Washington Health Research Institute (KPWHRI) to create the primary outcome measure. Again, all dates, site indicators and clinic indicators that are provided will be masked. In addition, any treatment assignments of the clinics that are provided (e.g. randomization to Intervention or Usual Care) will not represent the true treatment but rather will be scrambled and a dummy assignment for each clinic will be provided. This will permit the DSC to review code for the primary outcome measure without revealing anything about the observed treatment effect. Any other

quality checks that may be performed by the DSC to supplement those done by the Lead site at KPWHRI will follow these same principles (i.e., de-identified datasets, masked indicators of dates, site and clinic, and dummy treatment assignments).

Main analytic dataset supporting the primary objective on NIDA Data Share: The main analytic dataset supporting the primary objective will be de-identified and shared on the NIDA Data Share website. This dataset will have no data elements representing clinics or health systems. The NIDA Data Share website will explicitly indicate that data elements for site or clinic may be obtained from the Lead Investigator on a case-by-case basis, but access will be highly restricted and may require funding for programming to prepare and transfer the de-identified analytic dataset(s) and funding to establish a data transfer agreement and IRB approval. If data on sites and clinics are provided, names will be masked. De-identification includes masking all dates or zip codes prior to providing the data.

Other analytic datasets supporting main secondary objective or other secondary objectives/analyses: Investigators wishing to obtain analytic datasets for secondary analyses should contact the Lead Investigator to discuss the dataset request. Requests will be reviewed on a case-by-case basis and may require funding to support 1) programming to create the necessary de-identified analytic dataset(s) and 2) establish a data transfer agreement and IRB approval.

Rationale: Updating the data sharing plan in the protocol will allow for additional quality checks on our main outcome and data sharing beyond the main analytical dataset with interested investigators. The updated plan aligns better with the NIH policy on data sharing for promoting new research, encouraging further analyses and disseminating information to the community.

Modification #20 (Date: 11/16/2020)

Original protocol: Table 4 of the original protocol includes the secondary quantitative data variables being collected from each participating health care system for the main objectives of the study.

Modification: We are requesting to modify Table 4 of the protocol to clarify and add data variables.

1. Demographics: Modify the demographics section to add collection of the following variables "provider specialty", "type of provider" and "all providers". Currently this section already includes "each patient's primary care clinic & provider", which broadly covers these variables, so this is a clarification rather than a modification.
2. Health Care Utilization: Modify the health care utilization section to add "all procedure codes" as variables collected. Currently this section already includes "all health care utilization data", which broadly covers procedure codes, so this is a clarification rather than a modification.
3. Laboratory and Radiology: Modify the laboratory and radiology section to add "all COVID-19 related laboratory tests including inpatient and outpatient screening, and antibodies and any other tests reflecting immune response for the viral antigen" as variables collected. This is a modification to assist the study in collecting these new variables that were non-existent at the beginning of the trial but are now relevant due to the COVID-19 pandemic and may assist in interpreting findings from the 1-year extension of the intervention.

Rationale: The rational for clarifying and adding secondary quantitative data variables to Table 4 of the protocol is to ensure the data we are ascertaining for the trial are clearly documented as well as beneficial

CONFIDENTIAL

to the scientific and medical community during the rapid change of health care in 2020 due to the COVID-19 pandemic.

Modification #21 (Date: 11/16/2020)
**Original protocol:** Provider IDs are collected from the EHR of the health systems participating in PROUD for all encounters of interest. They are masked prior to secure file transfer to the Lead Node Data & Analytics Team (D&A Team) at KPWA. However, the original protocol does not indicate that we will be linking these provider IDs to nurse care managers (NCMs) that were specifically hired/assigned at each intervention clinic to implement the PROUD intervention.
**Modification:** We are requesting to modify the protocol to clarify that we will ask the research teams at each participating health system to provide the Lead D&A Team with the masked provider IDs for the NCMs specifically hired/assigned to implement the PROUD intervention. This includes all the main NCMs and back-up NCMs throughout the 3-year intervention period. Each health systems' research team already retains a crosswalk between the real provider IDs and masked provider IDs generated for the study to ensure that identifiable information, not allowed in a limited dataset, are not transferred to the Lead Node D&A Team for analyses. Using internal data sources, individual health system research teams will match their site's NCM names to the real provider IDs and then provide the Lead Node D&A Team with a list of corresponding masked provider IDs for these NCMs. The health system research teams will ensure that the real provider IDs and names of the NCMs are not included when this information is securely transferred to the Lead Node D&A Team.
**Rationale:** The rational for knowing the masked NCM provider IDs is to assist in describing the care provided by the NCMs hired/assigned for the main trial and will assist the Economic Ancillary study team in identifying the care provided by the NCMs.

Modification #22 (Date: 11/16/2020)
**Original protocol:** The original protocol indicated in section 10.2.1 that the two health systems that were not in the PROUD trial, but that were just providing exemplar clinics would also provide 4 usual primary care clinics as comparator clinics, with the purpose being to compare OUD outcomes of the exemplar and usual care clinics in the same health system.
**Modification:** We are requesting to modify section 10.2.1 of the protocol to indicate that we have decided that no usual care clinics (i.e. comparator clinics) will be included from the two health systems that are not in the trial but are only providing data on exemplar sites. Instead, we will compare the exemplar sites to the intervention sites adjusting for the baseline values of the outcomes at the same site.
**Rationale:** The rationale for this modification is that after extensive exploration, there are not appropriately similar comparator clinics in the two health systems, and the exemplar clinics drew from multiple clinics so that there would be cross-over/contamination between the exemplar clinics and any

similar clinics. This is in part because Colorado and Oregon have large rural areas and the exemplar clinics served their urban areas. Given differences in the available comparator clinics, they were deemed not comparable. For example, if we selected a comparator clinic that was close to the exemplar clinic, the measured OUD treatment in the comparator clinic could be artificially low if patients who were originally members of the selected comparator clinic transferred their care to the exemplar clinic. On the other hand, if we selected a comparator clinic that was far from the exemplar clinic, there would likely be a large number of differences in the two clinics due to geographical heterogeneity in patient characteristics and treatment provision (e.g. urban exemplar vs rural).

Modification #23 (Date: 11/16/2020)
**Original protocol:** This modification augments (and therefore supersedes) modification #15 above regarding extending the intervention period from 2-years to 3-years.
**Modification:** This modification is to request to extend the original 2-year intervention period to a 3-year intervention period. We would like to extend the time the nurse care managers (NCMs) are working in the clinics implementing the PROUD intervention by 12 months with continued support by the Technical Assistance Team. There will be no changes to the main trial analyses as we will still analyze outcomes of the trial as proposed in the protocol with a 2-year intervention period. In addition, a final data extraction will be added, after the 3-year intervention period ends on 2/28/2021, to allow us to conduct secondary analyses of the primary outcome, as well as other secondary analyses.<br><br>We are requesting to add study objectives to secondarily evaluate the impact of extending the intervention period. We have also added secondary objectives to reflect the changed circumstances after COVID hit the U.S. in March 2020.<br><br>Added Secondary Objectives:<br><br>To evaluate the primary outcome* of the PROUD trial over 3 years follow-up (*days of OUD treatment). This objective will involve replicating the main trial analyses 1 year after the end of the main PROUD trial (after 3 years post-randomization for 5 sites and 2.5 years post-randomization for 1 site).<br><br>Secondarily, if the PROUD intervention has an impact on the main outcome (days of OUD treatment over 2 years follow-up), we will evaluate whether time—before or after 3/1/2020--modified the effect of the PROUD intervention on the main outcome:<br><br>a. In the year before and after 3/1/2020 and;<br><br>b. In the 6 months before and after 3/1/2020 (before 2 NCMs left the intervention clinics).
**Rationale:** Recent data has revealed that the MA model of collaborative care that we are testing often takes more than 2 years to achieve steady recruitment rates of 1-2 patients a week. Therefore, NIDA decided that it was appropriate to test the PROUD intervention for 3 years in addition to main analyses of the trial after 2 years. Extending the intervention to 3-years will assist in seeing the full effect of the PROUD Trial intervention. Limiting the trial to 2 years might have led the NCMs to leave as the study ended, further undermining our ability to assess the effectiveness of the MA model. In addition, COVID hit the U.S. in March 2020 as the third year began. We therefore would like to take advantage of this

extension to evaluate the impact of COVID secondarily (testing whether any impact of the intervention was modified after 3/1/2020).

Modification #24 (Date: 11/16/2020)
**Original protocol:** The original protocol stated that the study did not include any interviews or surveys of primary care staff. The protocol was modified in March 2018 to include an anonymous 1-page survey at baseline of clinic staff's opinions on treating opioid addiction in primary care (modification #4 in the protocol).
**Modification:** We are requesting to modify the protocol to add a follow-up clinic staff survey to be administered in all randomized clinics (if they are able and willing) after the end of the 2-year intervention (as close to when the PROUD nurse leaves the clinic as possible). The purpose is to understand primary care clinic staff's opinions about treating opioid addiction in primary care. This 1-page survey will be anonymous and will include the same questions as the baseline survey. Due to COVID-19, and the possibility of fewer staff being physically in the clinics or possibility of clinics not holding staff meetings, we are offering an option to administer this survey online if this is preferred over in-person administration.<br><br>**The survey and supporting documents to administer the survey are being added to the protocol as Appendix G.**<br><br>New Appendix G Documents:<br>1. Instructions for Administrator<br>2. Information Sheet<br>3. Clinic Staff Survey (Follow-up)<br>4. Survey Invitation Email Template<br>5. Reminder Email Template for Survey Invitation
**Rationale:** The results of this anonymous follow-up survey will be compared to the baseline clinic survey results to assess changes in staff's opinions from the start (baseline) to the end (follow-up) on treating opioid addiction in primary care, comparing changes over time in intervention compared to control clinic responses. The follow-up survey will capture the same clinic characteristics as the baseline survey in key domains of the PRISM Model, which is the conceptual model being used by the PROUD Trial to guide efforts of implementing evidence-based care (section 3.3 of the protocol). The survey is comprised of items reflecting three PRISM Model domains and includes: acceptability, feasibility, and appropriateness of the intervention, commitment to change, valence (or capacity to affect change) and social norms. Items are selected from several implementation and organizational change assessments. The measure does not capture any identifying information and completion is voluntary.

Modification #25 (Date: 11/16/2020)
**Original protocol:** The original protocol did not include interviews with the nurse care managers (NCMs).

**Modification:** We are requesting to modify the protocol to add interviews of the NCMs near the end of the trial. We are asking that the Implementation Monitoring Team (IMT) interview the PROUD trial NCMs that were hired by the healthcare systems to implement the PROUD intervention.<br><br>These interviews with the NCMs will occur near the end of (or after) their work offering the PROUD intervention. The purpose of the interviews will be to better understand how the Office Based Addictions Treatment (OBAT) model was implemented in each healthcare system as part of the PROUD intervention during the trial, as well as capture barriers and facilitators to implementing and integrating opioid use disorder (OUD) care in primary care from the perspective of the PROUD trial NCMs.<br><br>In addition, the IMT will ask each NCM if they would be willing to be contacted by the economics study investigators, who may also ask for an interview to assess resources used to implement OBAT at each site).<br><br>**The NCM interview guide and supporting documents are being added to the protocol as Appendix H.**<br><br>New Appendix H Documents:<br>1. Invitation Email Template<br>2. Reminder Invitation Email Template<br>3. Reminder Invitation Phone Script<br>4. Reminder Email for Scheduled Interview<br>5. Information Sheet<br>6. Interview Guide<br>7. Future Contact Sheet<br>8. Thank You Letter<br>9. Request Review of Summary Notes Email Template
**Rationale:** The rationale for interviewing the PROUD trial NCMs is that collecting this qualitative information will be valuable in helping the study understand the implementation of the PROUD intervention from the perspective of the NCM within each healthcare system. Collecting and summarizing barriers and facilitators in the PROUD Trial related to barriers and facilitators of implementation in a variety of different settings all with different circumstances will be beneficial to the scientific community and healthcare systems interested in implementing this model of OUD care in primary care.

Modification #26 (Date: 04/15/2022)
**Original protocol:** As part of Aim 1 of the PROUD Trial Economic Analysis, "costs will be estimated using a macro-costing ("top down") approach, based on financial information received by the PROUD study team from the PROUD clinics as part of implementing PROUD. These data will be supplemented with information collected by the PROUD Implementation Monitoring Team (IMT) and from semi-structured interviews with Site PI's and Project Managers. Additional interviews, to aid in estimating PROUD costs, may be conducted with specific clinic staff, if necessary and approved by the Site PI." (see Appendix F).
**Modification:** As noted in the protocol, the Economic Analysis team will supplement PROUD data with information collected from "additional" interviews to aid in estimating PROUD costs. We are requesting to modify the protocol to specify that the Economics team at Weill Cornell Medicine would like to interview PROUD nurse care managers (NCMs) who implemented the PROUD intervention. The purpose

of these interviews would be to understand NCM involvement in the Office Based Addictions Treatment (OBAT) model implemented in their health system, as well as to obtain their perspective on the resources (e.g., time, supplies) required to implement and sustain the OBAT model.<br><br>As noted in Modification #25, during their interviews, the IMT asked each NCM if they would be willing to be contacted by the economics study investigators so the team could assess resources used to implement OBAT at each site. Five of the interviewed NCMs agreed to be re-contacted. Therefore, the Economics Analysis Team will only be conducting interviews with those NCMs who agreed to be interviewed. Note that none of the NCMs are currently in the same role at their health systems.<br><br>The interview guide and supporting documents for this modification have been added to the protocol as **Appendix I**. The new documents are:<br><br>1. Invitation Email Template<br>2. Reminder Invitation Email Template<br>3. Reminder Invitation Phone Script<br>4. Reminder Email for Scheduled Interview<br>5. Information Sheet for NCM interviews<br>6. NCM Interview Guide<br>7. Req Review of Summary Notes email template<br>8. Thank You Letter
**Rationale:** The rationale for the Economics Analysis team to interview the PROUD trial NCMs is, as follows:<br><br>Collecting this qualitative information will help the economics team to understand NCM involvement in the OBAT model implemented in their health system, as well as to obtain their perspective on the resources (e.g., time, supplies) required to implement and sustain the OBAT model. This work supports the overall goal of the economics analysis which is to compare the costs and benefits of the OBAT model, which is a collaborative care model for treating opioid use disorders in primary care, to treatment-as-usual.

Modification #27 (Date: 04/15/2022)
**Original protocol:**<br><br>The **primary objective** of the PROUD trial is to evaluate whether implementation of the Massachusetts Model of collaborative care for management of opioid use disorder in primary care, increases buprenorphine or naltrexone pharmacotherapy for opioid use disorder (OUD).<br><br>A **main secondary objective** of PROUD is to test the hypothesis that primary care patients with recognized OUDs prior to randomization who receive primary care in a PROUD intervention clinic will have fewer days of acute care utilization, relative to comparable patients receiving primary care in a usual primary care (UPC) clinic.<br><br>**Other secondary objectives** are included in the protocol as well.

**Modification:**<br><br>We are requesting a modification of the protocol to conduct additional secondary analyses, that are not already in the protocol, that will:<br><br>1) Describe the patterns of healthcare resource utilization, cost, and cost drivers of pregnant women with OUD.<br>2) Estimate healthcare costs by resource category for persons with co-occurring OUD and housing vulnerability.<br>3) Describe the association between the PROUD intervention and incidence of non-fatal self-harm.<br>4) Describe stimulant use and use disorders in primary care patients with OUD in the PROUD trial.<br><br>These secondary analyses are retrospective observational analyses of existing PROUD trial data.
**Rationale:**<br><br>The rationale for the Economics Analysis team to conduct additional secondary analyses is, as follows:<br><br>1) OUD is rapidly becoming a growing public health problem among pregnant women in the US and is associated with poor obstetric and postpartum outcomes. The literature has shown 1) the utilization of behavioral health services and evidence-based pharmacotherapy for OUD during pregnancy is low, and 2) pregnant women with OUD utilize less prenatal care than pregnant women with other substance use disorders. The PROUD trial provides a unique data resource to extend our understanding of the economic consequences of OUD during pregnancy and provide needed context for future treatment interventions for this population.<br><br>2) Persons with OUD who experience housing vulnerability face a higher burden of chronic illnesses and mental disorders compared to housed persons with OUD. The literature has shown there are higher healthcare costs for both the general and Veteran homeless populations, compared to housed populations. Healthcare costs of persons with OUD who are also experiencing housing vulnerability is understudied. Given the paucity of studies on health economic outcomes of persons with co-occurring OUD and housing vulnerability outside of inpatient studies, this secondary PROUD data analysis will fill an important gap in the literature.<br><br>The rational for the Main PROUD trial team to conduct additional secondary analyses is, as follows:<br><br>3) Effective treatment for OUD is expected to reduce self-harm. This analysis will evaluate whether implementation of the Massachusetts Model in the PROUD trial improved OUD enough to decrease self-harm.<br><br>4) OUD is more difficult to treat in patients with stimulant use. Little is known about comorbid stimulant use and OUD in primary care. These analyses will help to address this gap.

Modification #28 (Date: 11/18/2022)

Original protocol:

The **primary objective** of the PROUD trial is to evaluate whether implementation of the Massachusetts Model of collaborative care for management of opioid use disorder in primary care, increases buprenorphine or naltrexone pharmacotherapy for opioid use disorder (OUD).

A **main secondary objective** of PROUD is to test the hypothesis that primary care patients with recognized OUDs prior to randomization who receive primary care in a PROUD intervention clinic will have fewer days of acute care utilization, relative to comparable patients receiving primary care in a usual primary care (UPC) clinic.

Other **secondary objectives** are included in the protocol as well.

Modification:

We are requesting a modification of the protocol to conduct additional secondary analyses of existing PROUD trial data, including data of non-randomized clinics included in baseline and exemplar analyses. These analyses will include:

1. **Observational analyses of factors associated with OUD and SUD outcomes in primary care**
   Observational analyses will be conducted in different samples, including the entire trial population, patients with OUD, patients being treated for OUD, and other demographic and clinical subgroups.
   <u>Independent variables</u> considered will include but not be limited to demographic, clinical, laboratory, and utilization patient characteristics such as age, sex, race, ethnicity, insurance status, and other sociodemographic variables such as neighborhood deprivation index, mental health diagnoses, pain diagnoses, prescribed opioids, documented SUD diagnoses, medical conditions associated with SUD (e.g. HCV, HIV, etc.), urine drug screens and other laboratory tests, and specialty mental health treatment, etc. as well as system characteristics (e.g. residency clinics vs not, number of buprenorphine prescribers, and attitudes expressed in anonymous staff surveys, etc.)
   <u>Outcomes will include:</u>
   - OUD diagnosis and treatment in primary care (e.g. duration of treatment, dose of buprenorphine, tapering off buprenorphine, frequency of visits, etc.)
   - Adverse clinical outcomes (e.g. overdose, suicide, HIV/HCV diagnosis, acute health care utilization, etc.)
   - Diagnosis and treatment of other substance use and mental health disorders and substance use related outcomes (e.g. stimulant use disorder with and without OUD, etc.)
   - The quality of care for OUD, other substance use disorders, and mental health disorders in primary care (e.g. urine drug tests, counseling, measures of medication adherence, and evolving national quality measures, etc.)
   - Attitudes toward OUD treatment in primary care from anonymous surveys
2. **Additional analyses comparing PROUD and usual care clinics regarding additional secondary outcomes (e.g. outcomes listed under #1 above, attitudes on anonymous surveys etc.).**
3. **Evaluation and comparisons of different approaches to modeling substance use outcomes in primary care and in pragmatic trials.**
4. **Description of barriers and facilitators encountered in implementation of the PROUD intervention across individual sites and description of other programs addressing the opioid epidemic in primary care at the different sites.**

Rationale:

The rational for conducting these additional secondary analyses of existing PROUD trial data is to allow for important scientific questions to be answered with the data already collected that can benefit and inform the scientific and medical communities. These analyses will maximize use of the PROUD trial data for research to address the opioid crisis.

CONFIDENTIAL

1.0 LIST OF ABBREVIATIONS

AUD	Alcohol Use Disorders
BMC	Boston Medical Center
BUP	Buprenorphine
CCC	Clinical Coordinating Center
C-IRB	Central Institutional Review Board
CMS	The Centers for Medicare and Medicaid Services
CTN	Clinical Trials Network
CTP	Community Treatment Program
DHHS	Department of Health and Human Services
DM	Data Monitoring
DSC	Data and Statistics Center
DSM-5	Diagnostic and Statistical Manual – 5th Edition
DSMB	Data and Safety Monitoring Board*
DUA	Data Use Agreement
EDC	Electronic Data Capture
HER	Electronic Health Record
FDA	Food and Drug Administration
FQHC	Federally Qualified Health Centers
FWA	Federal Wide Assurance
GAD	Generalized Anxiety Disorder
GCP	Good Clinical Practice
HCPCS	Healthcare Common Procedure Coding System Codes
HCSRN	Health Care Systems Research Network
HIPAA	Health Insurance Portability and Accountability Act
HSN	Health Systems Node
ICD	International Classification of Disease
ICH	International Conference of Harmonization
IDU	Injection Drug Use or Injection Drug User
IRB	Institutional Review Board
KPCO	Kaiser Permanente Colorado
KPNW	Kaiser Permanente Northwest
KPWA	Kaiser Permanente Washington

CONFIDENTIAL

KPWHRI	Kaiser Permanente Washington Health Research Institute
LI	Lead Investigator
LN	Lead Node
LNT	Lead Node Team
MA Model	Massachusetts Model
MF	Montefiore
NCM	Clinic-based Nurse Care Manager
NDCs	National Drug Codes
NESARC	National Epidemiologic Survey on Alcohol and Related Conditions
NIDA	National Institute on Drug Abuse
NSDUH	National Survey on Drug Use and Health
NTX	Naltrexone
OBAT	Office Based Addiction Treatment
OBOT	Office Based OUD Treatment
OCD	Obsessive Compulsive Disorder
OTP	Outpatient Treatment Program
OUD	Opioid Use Disorder
PDV	Protocol Deviation
PC	Primary Care
PI	Principal Investigator
PN	Patient Navigation or Patient Navigator
PRISM	Practical, Robust Implementation and Sustainability Model
PT	Participant
PTSD	Post-traumatic Stress Disorder
QA	Quality Assurance
RA	Research Assistant
RE-AIM	Reach, Effectiveness, Adoption, Implementation Fidelity, Maintenance
RRTC	Regional Research and Training Center
SAP	Statistical Analysis Plan
SOP	Standard Operating Procedure
TA	Technical Assistance
UPC	Usual Primary Care (Control Condition)
VDW	Virtual Data Warehouse

CONFIDENTIAL

* Please note: NIDA CTN's DSMB acts as a scientific review board for all CTN studies, as well as a data monitoring board doing interim reviews of data for low risk studies. Therefore, even though this is a minimal risk study, it has been reviewed by NIDA CTN's DSMB.

2.0 STUDY SYNOPSIS

2.1 Study Objectives

Evidence-based treatment for opioid use disorders (OUDs) includes medications, and two medications for OUDs—buprenorphine and naltrexone—can be prescribed in primary care (PC). However, despite the current opioid epidemic and expert recommendations that OUDs should be treated in PC, most PC clinics do not offer treatment for OUDs. This reflects a lack of consensus among health system leaders and clinicians that OUDs *should be* treated in PC. The overall objective of the PRimary care Opioid Use Disorders (PROUD) trial is to provide information to guide health system leaders in the decision of whether or not to treat OUDs in PC, by evaluating the benefits of implementing a program that integrates high quality OUD treatment into the normal flow of PC.

Specifically, the **primary objective** of the PROUD trial is to evaluate whether implementation of the Massachusetts (MA) Model[1] of collaborative care for management of OUDs in PC (the "PROUD intervention") increases OUD treatment with buprenorphine or injectable naltrexone, documented in the electronic health records (EHRs) of PC patients, over a 2 year follow-up, as compared to usual PC (UPC).

Our primary hypothesis is that there will be a significant increase in the number of patient-days of medication treatment for OUDs documented in the EHR of PC patients in the 2 years after clinics are randomized to the PROUD intervention compared to PC clinics randomized to Usual Primary Care (UPC). This implementation objective reflects whether the PROUD intervention increases initiation of and/or retention in OUD treatment, documented in EHRs within medical settings.

The **main secondary objective** is to test our hypothesis that PC patients with OUDs in the 3 years prior to randomization who receive care in PROUD intervention clinics, compared to those who receive care in UPC clinics, will have fewer days of acute care utilization (including urgent care, emergency department [ED] and hospital care) in the 2 years after randomization. This "effectiveness" objective assesses whether implementation of the MA Model improves patient outcomes.

Other Secondary Objectives include: (1) to conduct observational analyses comparing outcomes of the PROUD intervention clinics to 10 other PC clinics with promising models of OUD care (from the same health systems and 2 other health systems from PROUD Phase 1); (2) to evaluate differences in the impact of the PROUD intervention across sex and race/ethnicity; (3) to test whether the PROUD intervention improves secondary implementation, patient, and process outcomes; and (4) to identify barriers and facilitators to implementation of the MA Model.

2.2 Study Design

The PROUD trial is a hybrid type III pragmatic, cluster-randomized, quality improvement trial. Hybrid type III trials are mixed effectiveness and implementation trials, with greater emphasis on implementation.[2] The trial will be conducted in 6 health systems across the United States. Randomization is stratified by health system. Each health system has recruited 2 PC clinics (or a cluster of smaller clinics) willing to implement collaborative care for patients with OUDs using a model developed at the Boston Medical Center (BMC) in Massachusetts and spread across federally qualified health clinics in that state (the "MA Model" hereafter). One of the two recruited PC clinics in each health system will be randomized to implement the MA Model, while the other will continue with UPC.

All outcomes will be evaluated using secondary data from electronic health records (EHRs) and/or health insurance claims during the 2 years after randomization.

2.3 Sample and Sample Size

The sample for the trial consists of patients who have visited the selected 12 PC clinics in the 6 participating health systems. Health systems were selected for the PROUD trial based on 1) leadership support for participating in the trial, 2) elements of clinic eligibility such as adequate size and having at least 3 PC providers willing to prescribe buprenorphine in each of the PC clinics, and 3) a demonstrated ability to obtain the secondary data necessary for the PROUD trial measures—specifically days of OUD treatment with buprenorphine and naltrexone and days of acute care utilization. Smaller clinics were eligible if a group of clinics near each other included adequate numbers of patients (target ~10,000 unique patients with visits in a year) and were willing to participate as a single clinic for purposes of this trial, i.e., if selected to implement the MA Model, a nurse care manager (NCM) would be shared.

PC patients 16-90 years old with at least 1 visit to the participating clinics from 3 years before to 2 years after randomization (the 5-year study period) will be included in the trial. The total sample of PC patients in the trial is anticipated to be over 170,000 patients across the 12 clinics, since over 14,000 patients were seen, on average, in each clinic in 2016. The implementation objective is addressed in the full study sample, while the secondary objective is evaluated in the subsample of patients who have EHR documentation of an OUD during the 3 years prior to randomization.

2.4 The PROUD Intervention and Duration

Intervention: Implementation of the MA Model of Collaborative Care for OUDs. The PROUD trial provides financial support to cover the NCM salary and technical assistance (TA) for the duration of the study, but the health system—not investigators—implement the MA Model program as part of quality improvement, and the health system and its clinicians provide all clinical care. One PC clinic or cluster of smaller clinics is randomized to the PROUD intervention in each health system ("*PROUD intervention clinics*" hereafter), and implements the MA Model after randomization. Specifically, the PROUD intervention includes 3 strategies used to implement the Model in Massachusetts.

(1) Clinic leadership receives funding for at least 1.0 full time equivalent NCM for 2 years after randomization and technical support for recruiting and hiring the NCM. Once hired for the study, the NCM will receive TA from experts in Massachusetts supported by PROUD, but NCMs will be employed and supervised by the health system.

(2) Experts at BMC who originally developed and disseminated the MA Model will: provide intervention clinics with a MA Model Manual; train PROUD NCMs at BMC for 2 days; and provide the ongoing TA for 2 years after randomization.

(3) Three PC providers in the PROUD intervention clinic obtain DEA waivers to prescribe buprenorphine for OUDs (if not already waivered) and work closely with the NCM to offer high quality PC care for OUD (e.g., medication treatment with buprenorphine or naltrexone with close follow-up to maximize retention in treatment).

Comparison: Usual Primary Care (UPC). Clinics randomized to UPC do not receive any resources or support from the study but are free to improve OUD care in any way they choose. UPC is the appropriate comparison to evaluate the impact of implementation of the MA Model on access to and quality of OUD care because most PC clinics do not currently offer treatment for OUDs, but that could change over the course of the trial. We will qualitatively evaluate OUD treatment at baseline via templated interviews with Site PIs and project managers, and monitor any changes in OUD treatment services over time, in UPC clinics.

2.5 Safety Reporting

This pragmatic trial evaluates a quality improvement intervention that will be implemented by the participating clinics' and health care systems' staff. Therefore, patient safety falls under the purview of the health systems' clinical systems overseen by clinical and quality leaders within the health system.

2.6 Study Outcomes

The primary outcome measure will be a clinic-level measure of patient-days of FDA-approved OUD treatment documented in the EHR in the 2 years after randomization. This measure of implementation is defined as the total number of days of buprenorphine or injectable naltrexone treatment for OUDs documented in the EHRs of PC patients of each clinic in the 2 years after randomization. To account for differing size of clinics, the outcome is reported per 10,000 patients who visited the clinic during the 2 year follow-up period.

The main secondary outcome measure, a patient-level outcome, is a composite of days of acute care utilization (urgent care visits, ED visits and days hospitalized) during 2 years of follow-up. This outcome will be evaluated in patients with an OUD diagnosis prior to randomization.

Other secondary outcomes include, but are not limited to: the prevalence of diagnosed OUDs, initiation of OUD treatment with medications; retention in OUD medication treatment for 1 year; opioid overdoses (ODs); and process measures of the quality of care for patients with OUDs (e.g., time to treatment).

2.7 Analyses

Analyses for the primary objective compare the implementation outcome in the 6 PROUD intervention clinics and the 6 UPC clinics. Specifically, we compare the total number of patient days of OUD treatment per clinic over the 2 years after randomization in PROUD intervention and UPC clinics in an intent-to-treat "per randomization" analysis. These primary analyses are at the clinic-level and as such will be analyzed using a random effects model to capture the correlation between clinics from the same health care system.

Analyses for the secondary objective compare days of acute care utilization in the 2 years after randomization in the 6 PROUD intervention clinics and 6 UPC clinics. The sample for these analyses is restricted to patients who were seen in the 3 years prior to randomization and who had one or more documented OUD diagnoses during that period. The planned analytic approach is to use a mixed-effect Poisson regression model (with log link) at the patient level to compare eligible patients seen in the MA Model or UPC clinics regarding the number of days of acute care utilization during the two years of follow-up.

CONFIDENTIAL

Figure 1. Outline of the PROUD Trial Design

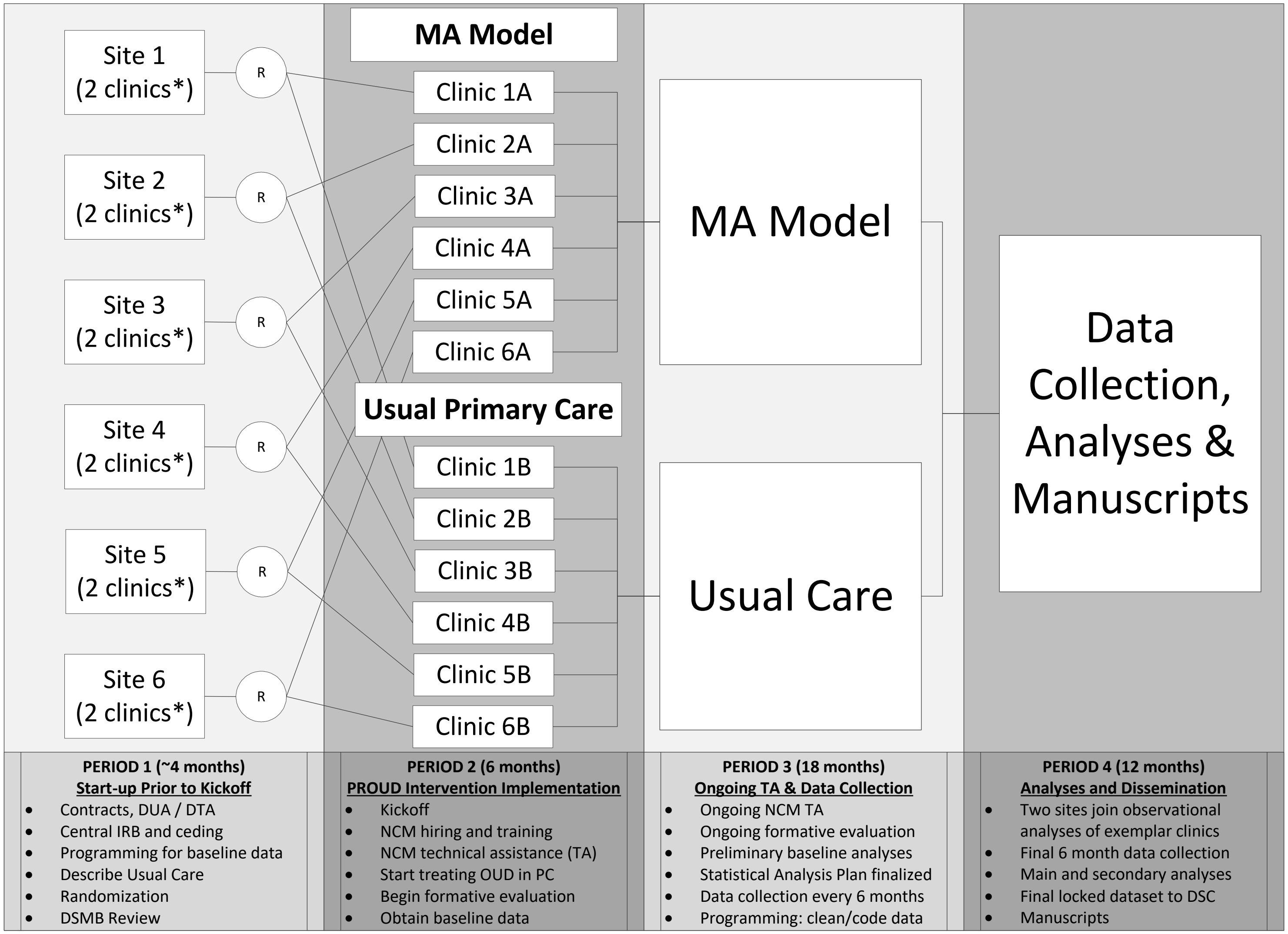

*or cluster IRB – Institutional review board; DUA – Data use agreement; DTA – Data transfer agreement; NCM – Nurse care manager; TA – Technical assistance; PC – Primary care

3.0 INTRODUCTION

3.1 Background and Significance to the Field

3.1.1 Epidemic of Opioid Use Disorder in US

Opioid use disorders (OUDs) have reached epidemic proportions; leaders across the US are calling for improved treatment.[3-5] Overall, overdose deaths in the US reached 52,404 in 2015.[6] Heroin deaths rose 23 percent in one year, to 12,989, slightly higher than the number of people killed with guns. Deaths from non-prescribed synthetic opioids, including illicit fentanyl, rose 73 percent to 9,580, while prescription opioid medications killed 17,536. By comparison, the number of people who died in car crashes was 37,757.[6]

The prevalence of OUDs—prescription opioids and/or heroin and other illicit opioids—in the general population has consistently increased over the past decade. US general population data from the National Survey on Drug Use and Health (NSDUH) indicate that 0.9% of adults age 12 and over met DSM-IV criteria for OUDs.[7] Applying DSM-5 criteria to define OUD is likely to increase prevalence estimates. Data from the most recent NESARC survey found that the prevalence of DSM-5 prescription OUDs alone (not including heroin) was 0.9%.[8,9]

The prevalence of OUDs may be elevated among PC patients. CTN study #0059 which validated a screening tool for tobacco, alcohol and drugs, found that in a sample of 2,000 general adult PC patients, 4.7% met DSM-5 criteria for OUD (heroin and/or prescription opioids).[10] In large cities (Baltimore and New York City) the prevalence of both heroin (5.6-6.3%) and prescription opioids (4.5-7.8%) misuse were considerably higher than in suburban areas (0-2.8%). Rates of OUD are also elevated in PC patients receiving prescriptions for opioids.[11,12] One study found that among patients receiving chronic opioids, the rate of OUDs was 4 times higher (3.8%) than in the general population (0.9%).[7,8] Many PC patients were prescribed chronic opioids for chronic non-cancer pain in the last 2 decades, and 3-26% become addicted depending on the sample and definition of OUD used,[12-16] with prescription opioids now accounting for the majority of OUDs.[9,14] While OUDs are often under-recognized in PC, patients on chronic opioids at increased risk for OUDs can be identified based on 5 characteristics in EHRs: (1) high morphine equivalent dose (> 90 mg morphine equivalent dose);[11,17-19] (2) documented alcohol or other substance use disorders;[18,20] (3) documented mental health disorders;[21-23] (4) concurrent sedative use,[24-27] or (5) widespread pain defined based on pain in different body regions (e.g., back pain and headaches).[28,29] Death rates among patients treated in PC and diagnosed with OUDs were 48.6 per 100 years (18% over 7 years follow-up) in one study.[30]

3.1.2 Improved OUD Outcomes with Medication Treatment

Extensive evidence has demonstrated that treatment of OUDs with medications increases abstinence, and improves health status and survival over behavioral treatments alone. Moreover, trials have demonstrated that medication management is effective even without behavioral interventions[31,32] and that there is no benefit of making patients pick up medications and have urine screening for drug use more often than weekly.[33] OUDs due to prescription opioid medications, constituting over 80% of OUDs in the US (2005-2013),[9] have poor outcomes without medication treatment.[34] Finally, medication treatment decreases total health care costs in most studies[35] compared to behavioral treatments alone.

3.1.3 Two Medications for OUDs Can be Prescribed in PC

Evidence-based medications for OUDs exist, and two can be prescribed in PC—buprenorphine, a partial agonist at the mu-opioid receptor, and naltrexone, an antagonist at the mu-opioid receptor. Patients with more severe OUDs or who need more structure can be treated in opioid treatment programs (OTPs) with methadone, a full agonist at the mu-opioid receptor, which is slightly more effective, but access is limited due to limited numbers and locations of programs and lack of coverage by Medicare. Medication treatment for OUDs, especially persistent treatment for 12 months, decreases ED visits, hospitalizations, and deaths.[36]

3.1.3.1 Buprenorphine

Buprenorphine is often considered first line[37]due to its greater availability, superior safety profile as a partial agonist compared to the full agonist methadone, and near equivalent outcomes in most samples of patients with OUDs. Buprenorphine treatment is sometimes used for symptom management while tapering patients off prescription opioids or for short term treatment (e.g., 12 weeks) instead of longer OUD treatment with buprenorphine. However, patients with OUD due to prescription opioid medications have poor outcomes after buprenorphine tapers and such short term treatment[34] irrespective of level of counseling. This suggests that long-term treatment with buprenorphine is also appropriate for patients with OUDs due to prescription opioid medications. In addition, evidence suggests that higher doses of buprenorphine improve outcomes (e.g., at least 16 mg daily dose).[38,39] However, no study to our knowledge has compared medication treatment for OUDs to usual care in comparable settings and populations. Because most people with OUDs never receive treatment, this is a critical comparison to understand the true benefits of implementing systems of care that improve access to high quality OUD treatment in PC. Moreover, it would not be ethical to conduct such a trial in patients seeking treatment for OUDs. As a result, the true impact of OUD treatment has never been evaluated in a randomized controlled trial.

3.1.3.2 Naltrexone

Early research on oral naltrexone suggested little benefit except in patients with enforced high adherence,[40,41] but injectable naltrexone appears more promising.[42] Two meta- analyses, using oral naltrexone to treat OUDs revealed significant heterogeneity,[41] and no benefit overall.[40] However, among patients who were forced to be adherent, there appeared to be a benefits in both retention in treatment and abstinence over placebo.[40,41] Other trials of samples with increased adherence due to external sanctions also suggest efficacy.[43,44] Trials of extended release injectable naltrexone, which only requires one intramuscular injection every four weeks, have shown more promise than the oral formulation.[45,46] Participants on active medication showed significantly superior retention in treatment and significantly less illicit opioid use compared to participants on placebo. A recent open-label randomized trial in the U.S. compared 380 mg extended release naltrexone every 4 weeks to treatment as usual (without medication) over 24 weeks among 308 criminal justice involved participants with DSM-IV opioid dependence.[42] Participants on active medication had fewer opioid-positive urine specimens and a longer interval to and less likelihood of opioid relapse.

3.1.4 The Need to Improve Access to Medication Treatment for OUD

Access to OUD treatment remains low. Less than 1 in 5 U.S. adults with OUDs receives medication treatment for OUDs.[9] This results from many factors including the limitation of methadone maintenance treatment to certified programs that have inadequate capacity. While buprenorphine use has increased over time in Medicaid samples,[36,47] there are substantial barriers to widespread use both inside specialty addiction treatment programs and in mental

CONFIDENTIAL

health and PC settings.[48] OUDs are often not recognized until patients seek OUD treatment, and most PC patients with OUDs are not treated with medications.[49] Even when buprenorphine is available, only 25% of patients with OUDs receive buprenorphine.[50-52] Moreover, 53% of patients with OUDs are privately insured but nevertheless have poor access to medications for OUDs.[53] The prevalence of OUD alone suggests that treatment for the majority of patients will need to be provided in PC.

3.1.5 OUD Treatment in PC Could Improve Access but Requires New Models

OUD treatment with buprenorphine or naltrexone can be provided in PC.[37] However, there are a number of barriers to PC providers doing so. These include perceptions that: 1) treating addiction is out of scope for PC; 2) PC lacks the resources, structure, and behavioral interventions required for OUD treatment (e.g., in person induction with close follow-up and urine monitoring, call backs and pill counts, and counseling); and PC providers need to focus on access and panel size so that taking on specialty care is not appropriate (i.e., “PC does not have time”).[54] Some leaders raise concerns that PC can’t offer as high quality OUD care as that provided in specialty settings.[54] Finally, pessimism and stigma pose a major, if often unspoken, barrier. PC providers or leaders are worried about attracting too many of “those” patients, and that patients with addiction will be difficult and overwhelming to PC clinics.[54]

Successful implementation of OUD treatment in PC has generally relied on a team to address the above barriers. A recent evidence review,[55] indicated that there are published peer-reviewed studies of 6 models of PC OUD treatment, and 12 models were described by key informants, many of them developed in part by collaborators on PROUD.[56-61] Many of these models have overlapping characteristics, with 4 characteristics noted as most common: 1) medications; 2) education and outreach; 3) care coordination; and 4) psychosocial services. Although many states are now testing a “Hub and Spoke model,”[55,62] the two PC models with the most support in the literature were the Yale office based OUD treatment (OBOT) model[33,55,63,64] and the Massachusetts nurse care management model,[1,47,65] which are very similar. The key difference between the models is that the MA model always uses a nurse for care coordination and also adds education and outreach—potentially key ingredients for improving access and quality of care.

3.1.6 The MA Model is a Collaborative Care Approach to PC for OUDs

The MA Model is a team-based, collaborative care approach that uses a fulltime clinic-based nurse care manager (NCM) to integrate medication treatment for OUDs into PC. The model is one of shared care between a NCM and PC providers who prescribe medications for OUDs, in which agreed upon algorithms allow the NCM to provide much of the routine care, with the provider’s role limited to diagnosis and treatment decisions, including referral to specialty addictions care when appropriate. Recently, evidence-based care for other addictions (i.e., alcohol use disorders)[66-68] has been added to the MA Model, which is now referred to as office based addiction treatment (OBAT). The role of the NCM includes telephone screening of patients, in-person intake assessments, coordinating a visit with a prescriber to diagnose and prescribe buprenorphine (or naltrexone), induction per protocol, monitoring, and once stable, placing prescriptions for refills in the EHR and monitoring and responding to urine drug tests. Most important, the NCM role allows the PC provider to treat OUD in the normal flow of PC. This allows PC clinicians to provide OUD treatment and experience the huge benefits to patients with little added work load beyond that of a typical PC patient. Providers even note that patients who are stable on buprenorphine are *easier* and more satisfying than typical PC patients, and that they have shorter visits freeing up time for more complex patients. The nurse also plays an educational role both for other staff within the clinic and in the delivery system.

3.1.7 The MA Model Improves Not Only Access but Quality

The MA Model of collaborative care for OUDs appears to increase both access and retention in treatment,[1,47,65] a strong predictor of outcomes.[69] In contrast the Hub and Spoke Model has not been shown to improve retention in treatment. Based on reports from Massachusetts and Harborview Medical Center in Seattle that implemented the MA Model in 2016, only 7-30% of patients with OUDs treated with buprenorphine were PC patients in clinics before they sought OUD treatment. Moreover, the MA Model results in high rates of long-term engagement: 51% and 67% of patients remain engaged in OUD treatment at 12 months in care: in 2 case series of 408 and 7,722 patients with OUDs, respectively.[1,47] Retention is a major predictor of health outcomes, as demonstrated in the Pennsylvania Medicaid program where only 21% of patients had persistent treatment at 1 year, but those with persistent treatment had lower rates of hospitalizations and ED visits than patients who left treatment sooner.[70]

3.1.8 Dissemination and Implementation of the MA Model to Date

Based on the success of the model at BMC, the experts at BMC developed a method of training and TA that supports implementation of the MA Model in other settings outside BMC. The MA Model thus also now includes a group of strategies for dissemination and implementation of the collaborative care approach to OUD treatment. The MA Model of care and implementation has been used predominantly in publically financed community clinics: including 22 Federally Qualified Health Centers (FQHCs) in MA[47,65] and recently in a PC clinic at a county hospital in Seattle (Harborview Medical Center). The model has been used to successfully treat large numbers of patients with OUDs in PC: at least 100-125/nurse. Moreover, the nurse can support multiple prescribers. When implemented in a public hospital and a state financed network of FQHCs it has rapidly attracted new patients with OUDs into PC, largely by virtue of word of mouth, responsiveness to patient need (without long wait times), and lower stigma compared with methadone OTPs. The majority of patients with OUDs treated in clinics using the MA Model had not previously received PC in those clinics.

The MA Model of implementing collaborative care for OUDs relies on NCM experts to train other NCMs and ensure that PC providers have adequate mentorship for treating OUDs. Specifically, the MA Model implements shared care using 3 strategies: (1) hiring a full time NCM for supporting addiction treatment in PC, (2) training the NCM at BMC and then supporting the NCM with ongoing TA with weekly performance monitoring and feedback; and (3) having PC providers prescribe buprenorphine and naltrexone supported by expert mentors.

3.1.9 Selection of the MA Model as the Intervention for the PROUD Trial

The decision to test the MA Model of implementation of OUD treatment in PC in PROUD was based on 2 factors. First it had the strongest published record of improving both access and high-quality care, as reflected in the fact that 50-67% of patients were retained in medication treatment at 1 year. Second, the experts at BMC, Labelle and colleagues, had already developed a successful strategy for widespread implementation. PROUD investigators considered testing models aside from collaborative care for treating OUDs in PC.[55] Models of treating OUDs in PC without dedicated staffing for OUD treatment were considered due to the lower upfront costs which might foster more rapid spread. However, unlike collaborative care, alternative models of OUD treatment in PC have not been extensively studied. The MA Model has a demonstrated ability to draw patients into PC for OUD treatment based on their methods of education and outreach and providing rapid access to treatment. ***The MA Model was the only model associated with documented high rates of persistent treatment at 12 months.[71] It is unknown whether alternative models of OUD treatment in PC result in as high levels of access and persistent OUD treatment.***

Spread and sustainability of other models of OUD care in PC clinics is unknown. Alternative models could have lower acceptability to PC practices if they place a greater burden on PC providers (which could dissuade them from prescribing medications for OUDs), or if they require local champions to spend more time implementing OUD treatment. In contrast, the MA Model has demonstrated its ability for spread and sustainability relying on centralized training and technical assistance of the NCM who then becomes the local champion. The model has resulted in high numbers of patients treated per clinic across MA: even though NCMs are expected to care for only 100-125 patients, in practices across MA, 227 patients are managed per a full-time NCM on average (data provided by Colleen Labelle on clinics in the state of MA). Moreover, research suggests that the model decreases Medicaid costs,[72] which could further improve its sustainability in other settings such as managed care organization, accountable care organizations and other models of value-based purchasing of health care. ***In short, the published experience on spread and sustainability of the MA Model make it unique.***

To summarize, the MA Model's collaborative care approach has a proven ability to attract and retain patients in OUD treatment and has demonstrated successful and sustained implementation. These features led the PROUD investigators to decide to test a collaborative care model of OUD care in PC over alternatives models (e.g., hub and spoke).[55,62]

3.1.10 Why Study the MA Model in a Randomized Controlled Trial?

The MA Model is not widely implemented outside MA despite the evidence supporting its effectiveness as a means to implement buprenorphine and naltrexone in PC and deliver high quality OUD care to patients. Despite high rates of opioid overdose deaths, health systems have not been willing to make the upfront investment of resources required by this model of care. This likely reflects all the barriers described above in Section 3.1.5 but also some unique barriers to this model. Because OUD is under-recognized, many systems may believe they are adequately treating OUDs by referring patients to specialty addiction treatment and might be hesitant to invest without documented need. ***The purpose of the proposed trial is to demonstrate the extent to which investing in a NCM and the MA Model can lead to identification of more patients with OUDs, expand access to high quality OUD care, and improve patient outcomes. In addition, future ancillary studies will evaluate the costs and benefits of the MA Model relative to usual PC.***

3.1.11 Addressing the Needs of Clinical Leaders

The target audience for this trial is health system leaders—of large health systems as well as PC clinic networks—who could implement the MA Model. The goal is to demonstrate the magnitude of expected improvements in access to and quality of, OUD treatment, as well as assessing any observed benefits to patients' health. At the same time ancillary studies will evaluate: costs and economic benefits (if any); organizational factors associated with successful implementation; and the impact of the intervention on survival in patients with OUDs or at increased risk for OUDs.

Most PC leaders are not seeking to improve OUD treatment in PC. In fact, based on our experience in Phase 1, many are resistant to the idea of providing OUD treatment in PC, believing that referral to specialty care is optimal for all the reasons outlined in Section 3.1.5 above. At the same time, implementing the MA Model requires a relatively large upfront investment in the salary of the NCM. Therefore, these leaders would need compelling evidence of how the MA Model improves care and outcomes over usual standard PC in order to dedicate upfront financial resources to implement this model. As a result, PROUD investigators chose to compare the MA Model to UPC. ***The primary and secondary objectives of this trial are NOT to determine which of the different models that have evolved for treating OUDs in PC is optimal. Rather the primary and secondary objectives are to determine whether continuing with usual PC***

compromises access to and quality of OUD treatment and outcomes for patients with OUDs, as compared to a promising practical approach to treating OUDs as part of PC. As a result, the optimal comparison for a pragmatic trial of the MA Model in large health systems is usual primary care (UPC) in the same health systems.

At the same time, some health systems *are* innovating, and this innovation may increase over time. As our nation recognizes and devotes increased resources to the opioid epidemic, with the accompanying innovation in OUD treatment practices across health systems, it is also important to compare the MA Model to alternative innovative models designed to provide high quality OUD treatment in PC. Such models include other team-based models for providing buprenorphine in PC as well as specialty care models that link PC to high quality specialty OUD treatment programs. Because the MA Model requires health systems to make an upfront investment in a full-time NCM, it is imperative to evaluate the benefits of the MA Model compared to other models perceived to provide high quality OUD treatment for PC patients. ***Therefore, although our main comparison for the MA Model will be PC clinics randomly assigned to "usual PC" (UPC), another objective of the PROUD trial will be to conduct observational analyses to compare outcomes of the MA Model to outcomes of other innovative models of PC for OUDs that health systems develop (referred to as "exemplar" PC clinics).***

3.2 Rationale for Pragmatic Trial Comparing the MA Model to Usual PC

3.2.1 Can the MA Model Increase Access to High Quality OUD Treatment?

Despite optimistic findings from several case series demonstrating the success of the MA Model of OBAT for improving access and retention in safety net clinics serving vulnerable populations,[1,47,73] the MA Model of implementation is unproven in other settings. Specifically, the MA Model has not been compared to the usual model of caring for OUDs in PC, which is typically referral to specialty addictions treatment, and occasionally treatment in PC with several PC providers treating a small number of patients with OUDs. Nor has the MA Model been evaluated outside safety-net clinics, or compared to other innovative approaches which integrate care for OUDs into PC[55] or create linkages between PC and addiction treatment programs, as we will be able to do using observational analyses to compare the MA Model to 10 exemplar PC clinics with innovative systems of OUD care.

3.2.2 Can the MA Model of collaborative care improve patient outcomes?

The other key unanswered questions relate to whether implementing the MA Model improves patient outcomes compared to usual PC for patients with OUDs. No randomized controlled trial has compared patient outcomes with the MA Model—or for that matter any other team-based approach to treating OUDs in PC—compared to usual PC.

3.2.3 Hybrid Effectiveness-Implementation Trial

We will therefore conduct a pragmatic hybrid effectiveness-implementation trial to address these unanswered questions.[2] Our primary objectives relate to the success of implementation, but key secondary objectives relate to patient outcomes. This type of hybrid effectiveness-implementation trial—emphasizing implementation over effectiveness, but evaluating both—is considered a Type III Hybrid.[2] Our primary comparison is to UPC in the same health care systems.

3.2.3.1 Primary Implementation Objectives

The primary objective of this trial is to evaluate the magnitude of the impact of the MA Model on access to and quality of medication treatment for OUDs, as reflected by total patient days of medication treatment for OUDs. Our hypothesis is that the MA Model will markedly increase patient-days of OUD treatment compared to UPC. Secondarily, we hypothesize that the MA Model will improve access to high quality OUD treatment in three ways: recognition of OUDs, initiation of OUD treatment, and retention in OUD treatment. First, as a result of the ready access to care by the NCM, we hypothesize that the MA Model will increase the number of patients with recognized OUDs in MA Model clinics compared to UPC clinics after randomization. This is because the model attracts new patients into PC for OUD treatment (as observed in MA and at the PC clinic at Harborview Medical Center in Seattle) and likely increases disclosure of OUD symptoms in PC when treatment is available in PC. Second, we hypothesize that the MA Model will markedly increase the number of patients who initiate treatment for OUDs, because of the NCMs' ability to screen and assess patients soon after they seek care for OUDs, resulting in decreased delay in treatment and increased engagement. Third, based on experience in Massachusetts and Seattle, we hypothesize that the MA Model will increase the proportion of patients treated for OUDs who are retained in medication treatment for OUDs at 12 months, which has been shown to improve outcomes.[70]

3.2.3.2 Secondary Effectiveness Objectives

We also hypothesize that due to increased access to high quality OUD treatment with the MA Model (for the reasons outlined immediately above), the PROUD intervention will improve OUD-related health outcomes for PC patients seen in PC in the 3 years prior to randomization who were diagnosed with OUDs during that time.[11,17-29] Specifically, we hypothesize patients in the PROUD intervention clinics will have fewer visits to urgent care clinics and EDs, as well as decreased days hospitalized compared to PC patients in the UPC clinics. Because the MA Model addresses all PC needs of patients with OUDs (e.g., diagnosis of other substance use disorders, prescribing naloxone, and screening and treatment of common comorbid conditions such as Hepatitis C (HCV), which could decrease overdoses and increase HCV viral cures), we will also measure these care processes and outcomes.

3.3 Conceptual Model for Successful, Sustained Implementation

Over the past two decades, implementation researchers have developed a number of evidence-based frameworks for guiding efforts at implementing evidence-based care. For the PROUD trial we will use the Practical, Robust Implementation and Sustainability Model (PRISM; Figure 2). The PRISM framework combines domains that impact the success of implementation as well as important domains of outcomes measurement. The upper portion of the PRISM Framework (Figure 2) summarizes the four domains that can act as barriers and facilitators to implementation: the intervention, and how it interacts with patient and organizational characteristics; the recipient of the intervention, including both patient and organizational characteristics; the external environment; and the implementation and sustainability infrastructure.[74] This aspect of the Model was built on previous literature and syntheses[75,76] including the Consolidated Framework of Implementation Research (CFIR),[77] which has been used previously to organize and understand barriers to buprenorphine implementation.[54] For example, the recently announced billing codes for collaborative care for mental health and substance use disorders[78] would be considered part of the external environment that might facilitate sustainability of the model after the trial concludes. In the PROUD trial, we will use ongoing formative evaluation to understand how the PROUD intervention overcomes barriers and facilitates implementation of collaborative care for OUDs, as well as to inform any adaptations needed to enhance implementation.

Figure 2. PRISM Framework (Feldstein, 2008)

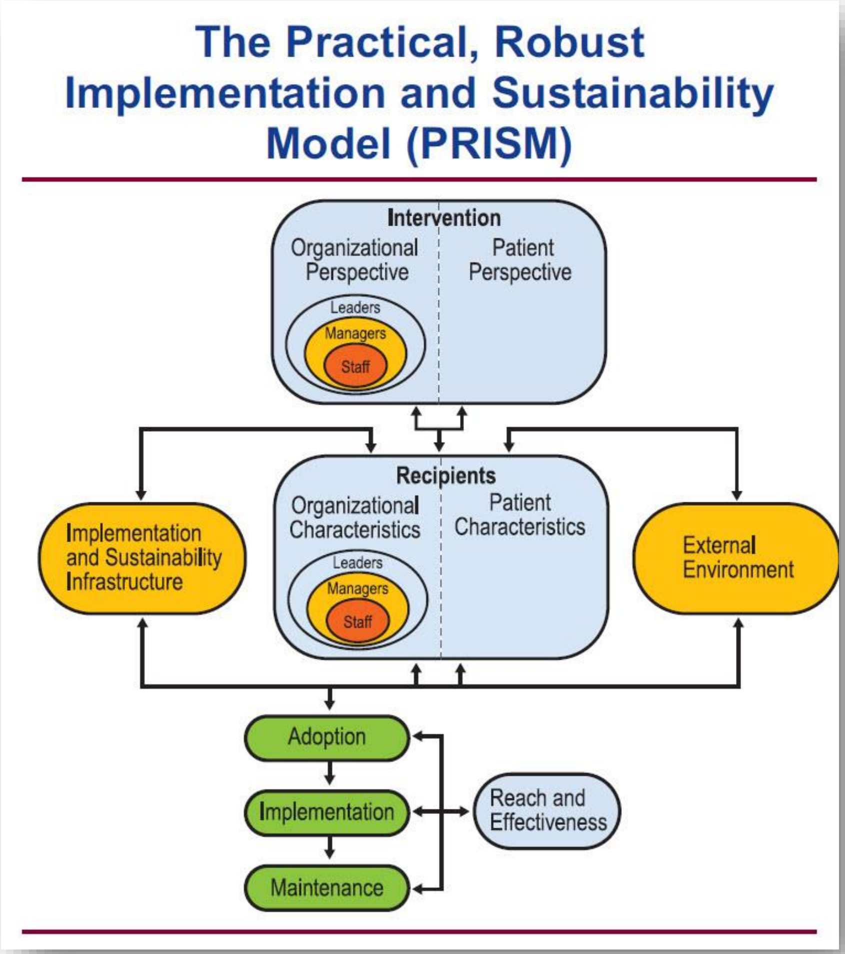

The lower portion of the PRISM (Figure 2: Reach, Implementation, Maintenance, Reach and Effectiveness) helps guide outcomes measurement using the RE-AIM Framework.[79-81] The "R" in RE-AIM stands for Reach which could indicate the number of patients who receive the desired care, such as initiation of medication treatment for OUDs (referred to as induction for buprenorphine). "E" in the RE-AIM Model stands for Implementation Effectiveness. For OUD treatment implementation effectiveness might be reflected in whether implementation resulted in high quality treatment, such as retention in treatment at 12 months. "A" in the RE-AIM Model stands for Adoption, for example by clinicians, and in OUD treatment implementation could refer to the number of new buprenorphine prescribers in PC clinics. "I" in the RE-AIM Model stands for Implementation fidelity, which could be reflected in the proportion of patients prescribed buprenorphine or naltrexone who have urines monitored or who meet MA Model targets for initiation of OUD treatment: 2 patients a week on average. "M" is for Maintenance of implementation, and reflects whether improvements were sustained after active implementation ended. Sustainability of implementation of the MA Model after the study ends would reflect Maintenance, but will not be systematically evaluated as part of the main PROUD trial, except for qualitative reports by Site PIs that the NCM is retained and supported by the health system after

CONFIDENTIAL

trial funding for the NCM ends. We have used the PRISM model to guide design of trial outcomes. Patient demographic and clinical factors that might impact implementation success are reviewed below.

3.4 Factors that Might Modify the Impact of the MA Model

3.4.1 The Role of Gender

Evaluation of the PROUD trial's results among men and women separately is important for several reasons. First, research on opioid addiction generally has focused primarily on male opioid users and their respective response to treatment.[82-84] Although rates of OUDs are higher in men than women, over a third of US adults with OUDs are women,[85] rates of heroin use among women has been increasing rapidly,[84] and there are no sex differences in terms of the percent of men and women past-year users meeting criteria for prescription opiate abuse or dependence (13.2%). The rate of prescription opioid overdose deaths in women is also increasing more rapidly than the rate in men.[86] More than 6,600 women died from prescription opioid overdose in the US in 2010 – 18 women a day. [87-89]Although research on the impact of biologic differences on outcomes is inconsistent,[90-95] there are biologic differences in neuro- and endocrine physiology related to opioids[87-89] and OUD medication treatments.[87]

Second, women might respond differently than men to the availability of OUD treatment in PC for several reasons. Women who use opioids also may have more severe psychiatric disease (particularly depression), greater chronic pain, and more children compared to their male counterparts,[82,96-98] all of which could act as barriers to treatment. Women have different help seeking patterns, and are more likely to seek care in PC.[99] Finally, and most importantly, a study of retention in the Massachusetts clinics implementing the MA Model revealed greater retention in OUD treatment among women.[73] Thus, although research shows that methadone maintenance therapy has similar treatment retention,[100,101] opioid use outcomes,[102,103] and mortality[104] for women and men, implementation of the MA Model in PC could impact men and women differently regarding initiation and retention in OUD treatment and subsequent patient outcomes.

3.4.2 The Role of Race/Ethnicity, Age and Socioeconomic Status

Race and ethnicity are also associated with the prevalence of OUDs (higher in white and Hispanic patients),[105] use of prescription opioids for non-medical use, transition to heroin, and overdose deaths (non-Hispanic Whites at highest risk).[105] Race and ethnicity are also associated with access to buprenorphine and methadone (buprenorphine access lower in Black,[106] non-white[50] increases in access over time (higher in non-Black, non-Hispanic and higher income areas),[107] and retention in treatment (higher in non-Black, non-Hispanic).[38,73,100] A study that evaluated retention in Massachusetts clinics implementing the MA Model found that among adults on buprenorphine from 2002 to 2014, Black race/ethnicity (AOR=0.53, 95% CI: [0.36, 0.78]) and Hispanic race/ethnicity (AOR=0.66, 95% CI: [0.48, 0.92]) were associated with lower odds of ≥1 year retention.[73] The study of retention in Massachusetts clinics implementing the MA Model also found that unemployment was associated with lower retention.[73] PROUD will use health insurance status as a proxy for socioeconomic status.

The study of retention in Massachusetts clinics implementing the MA Model also found that older age was associated with greater retention in OUD treatment.[73] Therefore, evaluating whether the MA Model increases access and retention in young adults is of critical importance. Many adolescents misuse prescription opioids (4.8% in 2015),[108] putting them at risk for lifetime OUD.[108] Young adults have the highest rate of prescription use. In 2015, an estimated 168,000 adolescents (age 12-17 years) and 430,000 young adults (age 18-25 years) had past year

prescription opioid use disorder.[109] In addition, an estimated 18,000 adolescents and 168,000 young adults had heroin use disorder.[109] These youth experience significant risks of morbidity and mortality, such as risk for mental health conditions, injury, Hepatitis C, HIV, and nonfatal and fatal overdoses.[110,111]

Buprenorphine is FDA approved for individuals 16 years and older. Outpatient treatment for opioid use disorder with medications such as buprenorphine is safe and effective for adolescents and young adults.[112-114] However, medications to treat opioid use disorders in primary care and outpatient settings for adolescents and young adults have remained underused,[115] and implementation of medication treatment for youth in outpatient settings has only been described in a few studies.[116,117] Because younger youth are thought by experts to possibly require additional counseling and other types of support, this study is evaluating youth 16 and older, focusing pre-planned secondary analyses on the 16-25 age group.

3.4.3 The Role of Mental Health and Medical Comorbidity

Psychiatric disorders co-occur frequently in patients with OUD with depression being the most common.[118] Previous findings are mixed in regard to the impact of psychiatric disorders on outcomes during treatment with methadone or buprenorphine with some studies finding that either illicit substance use or treatment retention was worse among patients with psychiatric symptomatology or psychiatric disorders[119] and some finding that patients with such symptoms or disorders had better outcomes.[120-122] Given prior somewhat contradictory findings, no directional hypothesis regarding the effect of co-occurring psychiatric disorders on OUD treatment outcomes in the proposed study can be generated at this time, but the study will examine the impact of psychiatric disorders via exploratory analyses.[118-122] The MA Model includes screening for mental health comorbidity (if such screening is not already in place), suggesting that the MA Model might also increase engagement in mental health care, and the study of retention in Massachusetts clinics implementing the MA Model found that a psychiatric diagnosis was associated with greater retention in OUD treatment. In contrast, Hepatitis C infection was associated with lower retention.[73]

3.5 Sustainability of the MA Model

The MA Model of OUD treatment in PC is expected to be sustainable for several reasons. First, the model is expected to be financially sustainable due to cost offset,[72] new reimbursement opportunities,[78] and the lower cost of PC providers compared to specialty addictions providers. Treating OUDs with medications results in considerable cost offset in acute care averted and in most studies show decreased total health care spending.[35,72] As a result, both insurance companies and integrated health care delivery systems, such as the Veterans Health Administration (VA) and Kaiser Permanente, are expected to reap cost savings. Third, insurers are expected to begin covering mental health care management due to overall health care cost savings. The Centers for Medicare and Medicaid Services (CMS) recently announced new payment codes for care management.[78] These codes allow reimbursement for care management, to supplement other payments for PC. The estimated annual reimbursement for coordination services from a single full time NCM is expected to be $103,440 based on estimated payments for the first month of $140, and the second month of $125 and $65 per month thereafter, and the minimum panel of a NCM in the MA Model of 100 patients with an additional 2 new patients a week on average (8/month). Harborview’s PC clinic recruited 76 patients in the first year (77% retention in treatment, 1 year after implementation), and their ability to bill for this OUD treatment has led to Harborview hiring a second NCM and Medical Assistant (MA). Second, the MA Model allows PC providers to treat OUDs in the normal flow of short appointments, making the model attractive to PC providers. These providers are considerably less expensive than addiction

specialists, and many report the great satisfaction of treating OUDs as part of PC (personal communication with lead investigator).

3.6 Preliminary Studies – PROUD Phase 1

The objective of Phase 1 of the PROUD Study (August 2016 to the Present) was to engage health systems in the trial and to demonstrate feasibility of data collection and sharing and to refine the intervention. There were 3 corresponding workgroups—data and analytics, health systems engagement, and intervention specification. The work was overseen by a Leadership Team of investigators from the Health Systems Node and Northwest Node, who met weekly for an Operations call with staff from the Center for Clinical Trials Network (CCTN) who manages NIDA's National Drug Abuse Treatment Clinical Trials Network (CTN), and the CTN's Clinical Coordinating Center (CCC) and Data and Statistics Center (DSC) both located at The Emmes Corporation (Emmes). Eleven health systems were supported to participate in Phase 1).

3.6.1 Phase 1 Health Care Systems ("Sites")

Phase 1 included 11 health systems, also referred hereafter as "sites", selected from 18 that applied indicating that they thought they could provide the required data and obtain permissions for participation in the trial (Phase 2 of PROUD). The 11 Phase 1 sites included 7 integrated health systems in the Health Care System Research Network that share a virtual data warehouse[123] (Kaiser Permanente Washington, Seattle, WA; Henry Ford Health Systems, Detroit, MI; Health Partners, Minneapolis, MN; Kaiser Permanente Northern California, Oakland, CA; Kaiser Permanente Southern California, Pasadena, CA; Kaiser Permanente Colorado, Denver, CO; and Kaiser Permanente Northwest, Portland, OR) and 4 other health systems (Multicare Health System, Tacoma, WA; Harris Health System, Houston, TX; Montefiore Medical Center, Bronx, NY; and University of Miami Health System, Miami, FL).

3.6.2 Phase 1 Site Engagement

The Phase 1 Health System Engagement Group, led by Dr. Bradley, consisted of Site PIs and Project Managers from all 11 sites. The group met every other week in November-December 2016 and weekly January-April 2017. The initial focus was obtaining DUAs and ceding IRB approval to Kaiser Permanente Washington Health Research Institute (KPWHRI). All sites were able to share a limited dataset with KPWHRI and de-identified data with Emmes. In addition, data transfer agreements (DTAs) were required between some sites and Emmes, to define permitted uses for de-identified data being shared by KPWHRI with NIDA CTN's DSC at Emmes. Ten sites ceded IRB review to KPWHRI (all except Multicare Health System).

Starting in January 2017, Site PIs worked to engage their health system leaders to assess whether the health system would be willing to participate in the trial to test the MA Model. Initially Site PIs spoke with key boundary spanners or stakeholders with whom they had connections, and finally with PC clinic and health system leaders. Five of the 11 Phase 1 sites were not able to obtain the required support from all levels of the health system (leaders, regional leaders, PC leaders, clinic leaders, and/or PC prescribers). ***This resulted in 6 of the 11 health systems being willing to participate in the trial and providing required letters of support: Montefiore (MF); University of Miami (UM); Henry Ford (HF); Harris Health (HH); Multicare (MC); and Kaiser Permanente Washington (KPWA).***

Table 1. Eligibility of Sites & Clinics for the PROUD Trial

- Regulatory and data sharing requirements
- Availability of required secondary EHR data
- Leadership support for the trial in the health system
- Leaders of 2 PC clinics support participation
- 3 willing PC prescribers in each participating PC clinic
- Adequately sized clinics with low cross-over of patients
- Desirable: geographic, demographic, site diversity

The Phase 1 Data Group, led by Drs. Boudreau and Lapham developed all operational Phase 1 data definitions for basic data on demographics, OUD prevalence, comorbidity and OUD treatment. The data group also developed methods for combining data on buprenorphine and naltrexone from dispensed and ordered medications; procedure data on naltrexone injections, and methadone maintenance from OTPs where available, for the main outcome. Finally, the data group provided data for power analyses. Only the 7 HCSRN sites and 1 other site met the original data deadline of March 15 for data, but all 11 sites provided required data by 3/19/2017.

Table 1 outlines site eligibility, which is also detailed in the site selection document. Because 6 sites were desired, and only 6 were eligible, the process focused on site eligibility. The characteristics of the 6 sites willing to participate are shown in Table 2 showing data from both clinics for 2016 except where otherwise stated.

3.6.3 Specification of "MA Model Care" and the PROUD Intervention

The Phase 1 Intervention Specification Group was led by Dr. Jeffrey Samet and it included experts in PC OUD treatment. They developed specifications for MA Model as would be implemented in the trial, including the NCM role (Appendix A) and refinement of the OBAT manual (Appendix B). Key determinations were that to maximize flexibility of the model the NCM role could be split between two individuals and that at least 75% of the patients managed by the PROUD NCM should receive PC in the PROUD intervention clinic(s).

The PROUD intervention—implementation strategies used to implement the MA Model care in the intervention clinics—were specified based on dissemination and implementation of the MA Model across MA and at Harborview: (1) the NCM would be funded full time from the start; (2) the NCM would be trained in Boston Medical Center where s/he could shadow MA Model NCMs for a day and have ongoing technical assistance (TA) from the TA team, and (3) the intervention clinics would have to have 3 buprenorphine prescribers.

3.6.4 Phase 1 Impact on Design Considerations

Based on low rates of OUD diagnosis and treatment in Phase 1 of PROUD, and findings in MA that each NCM can treat 100-125 patients and that retention is 50-67% at 1 year, we anticipate at least a 5-10 fold increase in the number of patient days of OUD treatment if each PROUD intervention clinic compared to control. Phase 1 also led to several changes in the protocol of the PROUD trial. We summarize these changes here.

Table 2. The 6 PROUD Phase 1 Sites Able to Participate in the PROUD Trial (2016)

	HF	HH	KPWA	MF	MC	UM
Total N seen in 3 years	86,262	60,463	46,214	34,763	27,031	32,687
% Medicare	25.86	3.13	21.35	0	19.57	16.31
% State subsidized	9.67	75.98	6.46	0	23.35	4.90
% Uninsured	1.21	0	6.59	0	0	0.06
% 16-25 years old	15.87	9.05	14.71	19.13	15.61	8.55
% African American	33.65	22.31	5.75	37.36	9.56	14.67
% Hispanic	3.19	55.59	6.93	74.57	4.55	49.76
% OUD	0.09	0.03	1.05	0.29	0.68	0.09
% buprenorphine	0.02	0.01	0.26	0.08	0.03	0.01
% OUD (any yr) + bup.	0.00	0	0.22	0.02	0.03	0.00
% Methadone OTP	0	0	0.10	0	0.01	0
% Urgent care	1.90	0	18.03	10.64	25.72	0
% any ED	20.07	14.73	13.60	23.65	23.54	10.01
% any hospitalization	2.90	5.99	7.26	9.20	8.12	4.85
% any acute care	21.86	18.59	29.99	31.81	43.39	13.39
% with OUD	0.04	0.02	0.67	0.22	0.57	0.06
% without OUD	21.82	18.57	29.32	31.59	42.82	13.33
N in clinic #1	34,114	11,390	14,271	15,222	9,776	9,018
N in clinic #2	16,540	17,652	15,667	8,851	9,138	11,527
% overlap: 2 clinics	0.33 – 0.46	0.11-0.27	0.13 – 0.15	0.28 – 0.43	0.01 – 0.04	0.23-0.29
% seen 2016 & in 2014-2015	64.0-67.8	60.7 – 64.7	76.3-77.1	73.9-81.4	75.0-88.2	60.3 - 63.3

3.6.4.1 Visit-based Sample

Because the 7 health plans in Phase 1 were able to provide more complete data on a defined population, we had expected that they would largely be selected for the PROUD trial. Having health plan data allows the study sample to be enrolled patients, so that even patients who have not visited PC can be included in the trial. However, most of the health plans were unable to participate in the trial; only 2 of the sites in the trial will be health plans (HF and KPWA). Therefore, a decision was made to use a visit-based sample at all sites (described in detail below).

3.6.4.2 Excluding Methadone OUD Treatment from the Outcome

Only 1 of the 6 sites able to participate in the PROUD pragmatic trial provided data on methadone maintenance in Phase 1 (KPWA). One other site indicated they could obtain data from internal OTP programs but was unable during phase 1 (MF); one other said they would obtain data from external OTP for the trial (HF). The others were health care *delivery* systems that did not have access to insurance claims or integrated delivery and health insurance plans where only a small fraction of the sample was covered by the plan (30%). A decision was made to exclude methadone from the definition of OUD treatment for the primary outcome measure. Since methadone can only be dispensed from licensed opioid treatment programs that would be outside of the health care systems studied, and, since only about 350,000 individuals are enrolled in licensed opioid treatment programs nationwide, it is very unlikely that the small number of PC patients in the study clinics in methadone maintenance programs would affect the overall PROUD outcomes.

CONFIDENTIAL

3.6.4.3 Low Prevalence Rates of OUDs in the PC Clinics at Baseline

As shown in Table 2, some sites had very low prevalence rates of OUDs at baseline (0.03%). This resulted in small numbers for analyses for our original secondary objective: to comparing acute care utilization during follow-up among patients with OUDs prior to randomization, between PROUD intervention and UPC clinics). However, power simulations for our secondary objective (below) indicate sufficient power despite the small sample with OUDs. A decision was therefore made to include patients at high risk for OUDs based on chronic use of high dose opioids for chronic pain in the presence of other risk factors.

3.6.4.4 Comparison to Other Exemplar PC Clinics

Phase 1 revealed that there were PC clinics at some sites that were already perceived as providing high quality OUD treatment—either in PC (e.g., with all PC providers prescribing or a non-RN care manager), due to systems of linkage between PC and specialty care, or due to nearby or co-located specialty addiction treatment clinics—so that PC patients were perceived to have both excellent access and retention. We therefore added an observational component that would compare PC clinics randomized to the MA Model to PC exemplar clinics thought to offer high quality OUD care. For this objective two health systems unable to participate in the trial will be included in observational analyses. We refer to these clinics from PROUD trial sites or other PROUD Phase 1 sites as “Non-randomized exemplar PC clinics”.

3.6.4.5 Adding Non-randomized Usual Primary Care (UPC) Clinics

During Phase 1 of PROUD, it became clear that the clinics that were agreeing to participate were often not “usual” in that they were willing to implement OUD treatment in PC, whereas many PC clinics were not willing to do so. Moreover, the very act of recruiting clinics appeared to increase interest in providing OUD treatment in PC. We, therefore, add other observational analyses that will compare randomized Usual PC Clinics to non-randomized PC clinics from each site in the trial (in addition to adding exemplar PC clinics for observational analyses, as described immediately above). The latter are referred to as “non-randomized UPC clinics” and allow us to evaluate generalizability of the randomized UPC clinics in the trial. Five of the 6 sites were able to commit to providing data from 4 non-randomized UPC clinics.

3.6.4.6 Ancillary Mortality Study

Mortality is an important outcome for OUDs. However, death data are obtained from the state and generally lag by 2 years. Further, while health insurance plans will often have data on the death of their enrollees, health delivery systems do not. Deaths will be documented in the EHR when known, but that process could be influenced by a NCM who reaches out to patients when they are lost to follow-up (biasing results). Therefore we will not use death from EHRs in main analyses, but a Health Systems Node (HSN) investigator with expertise in OUDs and mortality (I. Binswanger) has been invited to lead an ancillary study that will evaluate death in the sample for Objective 2 as well as conduct sensitivity analyses to determine whether deaths impacted main findings.[124-130]

4.0 OBJECTIVES

4.1 Primary Objective

The primary objective of the PROUD trial is to evaluate whether implementation of the MA Model of collaborative care for management of opioid use disorders (OUDs) in PC (the “PROUD intervention”) increases OUD treatment with buprenorphine or naltrexone over 2 years follow-up as compared to usual PC (UPC).

Our primary hypothesis is that there will be a significant increase in the number of patient-days of treatment for OUDs with buprenorphine or injectable naltrexone in the 2 years after randomization in clinics randomized to the PROUD intervention as compared to PC clinics randomized to UPC. This objective is an implementation objective, reflecting whether the PROUD intervention increases initiation of and/or retention in OUD treatment and will be expressed per 10,000 patients seen in each clinic during the intervention phase.

4.2 Secondary Objective

The main secondary objective is to determine whether the PROUD intervention—compared to UPC—decreases acute care utilization in the 2 years after randomization among PC patients with OUDs who were seen in the clinic during the 3 years prior to randomization. This objective assesses whether implementation of the MA Model improves patient outcomes.

Our secondary hypothesis is that PC patients with OUD in the 3 years prior to randomization who receive care in PC clinics randomized to the PROUD intervention, compared to those who receive care in PC clinics randomized to UPC, will have fewer days of acute care utilization (including urgent care, emergency department [ED] and hospital care) in the 2 years after randomization.

4.3 Other Secondary Objectives

4.3.1 PROUD Intervention vs. Other “Exemplar” Models of OUD Care

Several health systems in PROUD Phase 1 reported that some or all of their PC clinics had already implemented other approaches thought to provide improved access and quality of care for PC patients with OUDs. We refer to these as “exemplar clinics.” Therefore, another objective will be to compare patient days of OUD treatment and acute care utilization, as well as other secondary outcomes in those exemplar PC clinics compared to PC clinics randomized to the PROUD intervention, as well as to PC clinics randomized to UPC, in observational analyses.

4.3.2 Differences in Impact of PROUD across Age, Sex and Race/Ethnicity

An important secondary objective, per NIH Guidelines, is to understand whether the impact of the PROUD intervention differs based on sex, race, and ethnicity. In addition, understanding the impact of the PROUD intervention on patients 16-25 years old (compared to older patients) is important to address a critical gap in the literature. We hypothesize that receiving care in a clinic randomized to the PROUD intervention, compared to a UPC clinic, will increase OUD treatment in patients < 26 years old, albeit to a lesser extent than in older patients; in women more than men[73] and in patients of black or Hispanic race/ethnicity less than others.[73] Exploratory analyses will evaluate differences in outcomes between the PROUD intervention and UPC clinics across groups defined by psychiatric and medical comorbidity, including chronic pain.

4.3.3 Evaluate Secondary Outcomes

Another secondary objective is to evaluate important secondary Implementation, patient, and process outcomes, which are described under outcomes below.

4.3.4 Identify Barriers and Facilitators to PROUD Implementation

Another important secondary objective of the PROUD trial is to identify important barriers and facilitators of implementation of the MA Model in diverse health systems and develop any needed adaptations to the implementation strategy to support high quality implementation of the MA Model. To identify and address barriers to implementation of PC OUD treatment with the MA Model as they arise, we will conduct regular debriefs with Site PIs and Project Managers, as well as with the technical assistance (TA) team. This will be followed by formative evaluation and feedback of results of formative evaluation to PROUD intervention clinics.

4.4 Planned Ancillary Studies

The Lead Node is collaborating on 3 planned ancillary studies. The first is a study of the cost benefit of the intervention, organized by Dr. Bruce Schackman and the NIDA Center for Health Economics and Treatment Interventions in Substance Use Disorders HCV, and HIV (CHERISH) center (and led by Sean Murphy, PhD). The Scientific Reviewers of the Phase 1 Protocol requested that an economist be added to the protocol development team, and Dr. Murphy has joined both the data group and the protocol development group during Phase 1. In addition, we had added Kai Yeung, PhD, a health outcomes researcher who recently joined KPWHRI, to the data and analytics team. The data for cost benefit analyses are being collected during PROUD as part of secondary data collection at no additional cost. The second ancillary study is a study of the roles of organizational context in supporting (or inhibiting) success of implementation of the MA Model and team-based PC for patients with OUDs (Dr. Campbell, NY Node, lead). This study will be proposed as a just-in-time proposal to NIDA and will use data collected for the PROUD trial (formative evaluation and ongoing measurement of usual care and care in "Exemplar" clinics), as well as primary data collection at the end of the trial. Finally, death is a critically important outcome for the trial, but unbiased vital status data will not be available during the trial due to data lags and possible preferential ascertainment of death due to the nurse care manager in PROUD intervention clinics. Therefore, a later study of mortality is planned comparing opioid related mortality in the two study arms beginning at least 2 years after the active PROUD intervention ends (Ingrid Binswanger, MD, HSN lead). Dr. Binswanger has extensive experience using publically reported death data for research.[124,125,128,129,131,132]

5.0 STUDY DESIGN

5.1 Overview of Study Design

The PROUD trial is a pragmatic, Hybrid Type III, cluster-randomized, quality improvement trial. The trial will be conducted in 12 PC clinics, two from each of 6 health care systems. Each PC clinic, or a group of nearby PC clinics that function as a single clinic in the trial, are expected to have >10,000 unique patients who made visits to the PC clinic in the 3 years prior to randomization. As a result, the study will compare outcomes in ~60,000 PC patients who are exposed to the PROUD intervention to ~60,000 patients offered only UPC. Randomization is by clinic, stratified within health system, so that one clinic in each health system is randomized to the PROUD intervention, and one is randomized to UPC.

The study sample includes all patients who received PC in the 12 trial clinics during a 5 year period: from 3 years before randomization through 2 years after randomization (hereafter the “study period”). Main outcomes rely entirely on secondary EHR and administrative data from the participating clinics and health systems. The primary outcome is “patient days of OUD treatment” documented in the EHR in the 2 years after randomization, reflecting both initiation and retention in medication treatment for OUDs with buprenorphine or injectable naltrexone in PC. The main secondary outcome is the number of days of acute care utilization in the 2 years after randomization among patients with an OUD diagnosis in the 3 years prior to randomization.

The PROUD trial is considered pragmatic because it evaluates care provided by PC clinicians as part of routine care and because it compares outcomes between all patients cared for in clinics randomized to the PROUD intervention and all patients cared for in clinics randomized to UPC, and obtains all outcomes from secondary EHR or claims data.

The trial is considered a quality improvement trial because the health system leaders and clinicians, not the investigators, implement the MA Model of OUD treatment in randomly assigned PC clinics. Cluster-randomization at the level of the PC clinic is stratified within health system to maximize the balance of patient characteristics between the 2 study arms. As above, based on Curran et al.’s nomenclature,[2] PROUD is considered a Type III implementation-effectiveness trial, including predominantly elements of an implementation trial with evaluation of implementation outcomes (e.g., 4 out of 5 domains of the RE-AIM model: Reach, implementation Effectiveness, Adoption and Implementation fidelity, as above Figure 2), but also, secondarily, elements of an effectiveness trial measuring the impact of the MA Model on patient outcomes (days of acute care utilization among patients with OUD diagnoses, in the 3 years prior to randomization).

5.2 Study Duration and Activities During Each Period of the Study

The PROUD trial lasts 40 months including 4 study periods (Table 3): 1) startup prior to kickoff (~4 months); (2) PROUD intervention implementation (6 months); 3) ongoing TA and data collection (18 months); and 4) final data collection, analyses and dissemination (12 months). Each period is described below, but activities are summarized in Table 3 and in Figure 1 above.

Table 3. Overview of Periods of the PROUD Trial

**Period 1**<br>(~ 4 months)<br>Start-up Prior to Kickoff	Contracting, IRB approval, data use agreements (DUAs), data transfer agreements (DTAs) will be completed. Begin preparing code to measure OUD, OUD risk, and OUD care at baseline in all randomized and nonrandomized clinics. Prepare for ongoing data collection. Randomization (concealed) by DSC.
**Period 2**<br>(6 months)<br>PROUD Intervention Implementation	Trial kick-off meeting in Seattle: lead node, site teams, CCTN, DSC, CCC, and technical assistance (TA) team. Sites hire NCMs, training in Boston by technical assistance (TA) team, PCPs waivered and start prescribing, site visits from TA team. Baseline data collection (retrieve records for 3 years pre-randomization), and first Data Safety Monitoring Board (DSMB)* report of "baseline data" from the 3 years prior to randomization. Prior to the baseline data extraction, the SAP will be completed for the primary objective analyses and all baseline analyses pertinent to the primary objective.
**Period 3**<br>(18 months)<br>Ongoing TA & Data Collection	Ramp up of OUD treatment in PC at 6 PROUD intervention clinics with ongoing TA and ongoing formative evaluation; weekly reports from each PROUD Intervention clinic to TA team and lead node; ongoing secondary quantitative data collection every 6 months with reports to DSMB"; monitoring all aspects of performance. Planning for support for NCM after trial ends. SAP approved by NIDA CTN's DSMB* and locked during Period 3.
**Period 4**<br>(12 months)<br>Final Data Collection,<br>Analyses & Dissemination	Two additional sites with exemplar clinics join the study to provide secondary data on exemplar clinics. Final data collection (6 months after end Period 3), main and secondary analyses; and manuscript preparation.
* Please note: NIDA CTN's DSMB acts as a scientific review board and a data monitoring board doing interim data reviews in low risk studies. Therefore, even though this is a minimal risk study, it has been reviewed by NIDA CTN's DSMB.	

5.3 Overview of Randomization and the Intervention

Randomization (1:1), conducted with R software, will be stratified by health care system, resulting in one intervention and one UPC clinic per healthcare system. One or both of the 2 randomly assigned "PC clinics" in each system might be a cluster of 1-3 smaller nearby clinics that share a NCM. Although the PROUD trial randomly assigns one PC clinic in each health care system to receive the PROUD intervention, the MA Model is implemented by the health care system. The PROUD intervention consists of three strategies to support randomly selected clinics in implementing MA Model OBAT care: providing funding for an NCM for 2 years, providing training and TA from experts in Massachusetts; and requiring that 3 PC clinicians become waivered and agree to prescribe buprenorphine and naltrexone for OUDs.

5.4 Overview of Data Collection

5.4.1 Quantitative Secondary Data Obtained from Health Systems

All data for PROUD main outcomes (primary and secondary) and additional quantitative outcomes comparing PROUD Intervention and UPC clinics are derived from data from the EHRs, other health system administrative databases, and insurance claims (when available) at least every 6

months from the 6 health systems participating in the trial. Similar data will also be obtained from the two other Phase 1 health care systems (not participating in the trial) to allow observational analyses comparing access, quality and patient outcomes in the PROUD intervention clinics and in other (non-randomized) exemplar PC clinics perceived to provide access to high quality OUD care. Designated study programmers at Kaiser Permanente Washington and each of the other health systems participating in PROUD will identify the study sample using EHR and claims data as available at each system. In Table 4, we outline the data components to be collected during the study period.

Table 4. Types of Secondary Quantitative Data Obtained from Each Site

**Demographics**	Date of birth, sex, race/ethnicity, insurance type (Medicare, commercial, state subsidized/ Medicaid, uninsured, self-pay), zip code (at baseline), to characterize the study sample. Each patient's primary care clinic & provider. Dates of enrollment in health plan (for 2 sites that are health plans).
**Pharmacy dispensings, medication orders, and injections**	All dispensings (when available), orders and order refill data when available, and injections for medications related to treatment of opioid and other substance use disorders and addictive substances (e.g., buprenorphine, naltrexone), and risky medications for patients with alcohol or drug misuse (e.g., opioids, sedative hypnotics, stimulants, muscle relaxers). For each dispensing/order, the following data will be collected: drug name, days' supply (dispensings only), unit dose per pill or implant or injection, quantity dispensed/ordered, date, directions for use (orders only) and prescriber. Dates and drug name will be collected for injections.
**Laboratory and radiology**	Urine drug screens and dates, and results where available, virology (e.g. HCV, HIV related tests for secondary outcomes) and radiologic procedures to characterize samples (e.g. pregnancy for objective 1, fractures for objective 2)
**Health care utilization**	Dates of visits related to study objectives e.g., PC, integrated behavioral health, mental health clinics, hospitalizations (admit/discharge dates), urgent care, ED, certified OUD treatment programs (where available), other substance use disorders treatment (inpatient and outpatient), hospice and palliative care. These data including all health care utilization data will be obtained for later cost analyses (planned ancillary study).
**Diagnoses**	All ICD-9 and ICD-10 diagnostic codes during the study period and dates, including but not limited to: OUDs; other substance use disorders (e.g., alcohol); mental health conditions (e.g., depression, anxiety), associated medical conditions (e.g., chronic pain, HIV, HCV, etc.), other relevant comorbidities and co-morbidity scores (e.g., Charlson and Elixhauser) for use characterizing the study samples, as covariates and as outcomes
**Mortality**	Death data will be obtained from all sites, although ~ 2 year lags in data result in under ascertainment.

5.4.2 Qualitative Data Collected

Qualitative data will be used not only for the formative evaluation regarding the PROUD intervention clinics above but to characterize OUD and other addictions care in the UPC and Exemplar PC clinics, and changes over time in care in all PC clinics not randomly assigned to the PROUD intervention. Specific domains will be outlined in the standalone Statistical Analysis Plan (SAP).

5.4.2.1 Baseline Qualitative Descriptions of Existing Practices

Existing practices of both clinics that will be randomized at each site will be described at baseline, prior to randomization. The Site Principal Investigator (PI) and project manager will complete a standard study questionnaire to describe each clinic. The questionnaire will be supplemented by interviews of all Site PIs and project managers prior to randomization by members of the implementation monitoring team, with additional information gathering by the Site PI and project manager, if needed to fully characterize current practices. For example, they might be asked to call the methadone OTPs used by their health system and characterize the current wait time for starting on methadone, if they do not know that information. The implementation monitoring team will also interview the Site PI and project manager for the two sites providing exemplar clinics at baseline to further characterize exemplar clinic's care for OUDs. Changes over time in the UPC and Exemplar clinics will be assessed via Site PI and project manager debriefs as described below.

5.4.2.2 Monitoring Usual Care in UPC and Exemplar PC Clinics

The implementation monitoring team will interview the Site PI and project manager quarterly to monitor changes over time in any factors that could impact OUD care (delivery, access, or quality). Each Site PI and/or project manager will be expected to check-in with key informants at each UPC or exemplar clinic at least quarterly before these interviews.

These quarterly interviews with the Site PIs and their teams will include (but are not limited to): changes in patient populations served or patient characteristics (e.g., by having a new contract with Medicaid, the military, or a large employer); changes in clinic or department leadership, other changes in the organization, changes in the EHR, changes in the legal, policy, economic, or social environment, changes in OUD treatment practices, and other factors that might have impacted usual care for OUDs in the study clinics, including assessing where patients receive addictions treatment generally, what services are available in PC ,and where and how patients receive medication treatment for OUDs. A similar process will be carried out for the 2 sites that are not in the trial but are providing data on exemplar clinics (KPCO and KPNW) but only every 6 months.

5.4.2.3 Monitoring implementation

We will monitor implementation of the PROUD intervention for the formative evaluation (Section 12.1) as well as the description of barriers and facilitators to implementation of the MA Model (Section 12.2). Two types of data will be collected. First, the debrief above of the Site PI, project manager and lead programmer in each health system will cover any information they have on barriers and facilitators encountered as part of implementation, and also changes in the health system that might impact implementation using the domains of the PRISM model, as above. Second, qualitative data for the formative evaluation (PROUD intervention clinics) will be collected from monthly debriefs with the TA team in Boston and listening to the TA calls with NCM. The implementation monitoring team will interview the two TA nurses about interactions with each PROUD intervention clinic that month. This debrief will include review of both facilitators and barriers that each NCM and site reported and will be documented by an administrative assistant who will type detailed notes. The implementation monitoring team will summarize findings and recommendations for any changes in implementation for the study team.

CONFIDENTIAL

6.0 STUDY POPULATION

The study population includes 4 sub-samples.

1. *Main PROUD trial sample*. The main PROUD trial sample includes PC patients who visit the 2 randomized clinics in each of 6 health care systems participating in the trial—Harris Health System, TX; Henry Ford Health System, MI; Kaiser Permanente Washington, WA; Montefiore Health System, NY; Multicare Health System, WA; and University of Miami Health System, FL.
2. *Exemplar clinic sample.* The exemplar clinic sample includes patients seen in PC clinics in 4 health systems that have implemented OUD treatment using other innovative approaches: two in the trial (MF and KPWA) and two not in the trial (KPNW and KPCO). Each will be described separately below.
3. Non-randomized UPC Clinic sample. Five of the 6 PROUD trial sites (all but UM) also agreed to provide data on patients who receive PC in 4 other non-randomized PC clinics, for a total of data from 20 additional clinics. These clinics are not participating in the trial, but will be compared to the randomized UPC clinics in secondary analyses to assess the generalizability of the PROUD trial clinics. These PC clinics will be selected randomly from among each of the 5 site's PC clinics that are not in the trial.
4. "*Enrolled" samples*. At 2 health systems (HF and KPWA) that are health insurance plans, more inclusive samples of patients "enrolled" for health insurance will be used for sensitivity analyses to assess biases in the main visit-based samples and in measures.

6.1 Primary "Visit-based" Samples

The primary samples for main and secondary analyses are visit-based samples. Patients are eligible if they visited a participating PC clinic and met inclusion criteria detailed below. The standalone Statistical Analysis Plan (SAP) will define the detailed algorithm for assigning patients to a PC clinic when patients have been seen at more than one PC clinic in the trial, based on Phase 1 analyses.

6.2 PROUD Trial Sample

Patients are eligible for inclusion in the sample for analyses of the PROUD trial if they visited one of the randomized clinics at any time in the 5 year study period. Table 1 above shows key characteristics of the 6 study samples at the sites participating in the PROUD Trial.

Inclusion criteria for the trial are:

1. Age is 16 to 90 years at any time during the study; and
2. Visited a PROUD trial PC clinic in the 3 years prior to randomization or the 2 years after.

Again, details may be refined in the SAP based on ongoing Phase 1 analyses, but we currently expect patients to be assigned to a PC clinic if they make at least one visit during the 3 years prior to randomization or during the 2 years post-randomization (visit-based sample). In the rare cases that patients visit both PROUD Intervention clinics and UPC clinics (see % overlap 2016 in Table 1), they will be assigned the clinic which they visited the most pre-randomization (if they visited during that period), and if they are tied, the clinic visited nearest to and preceding the time of randomization will be considered the patient's PC clinic. If patients only visit a trial clinic post-randomization, and they visit both PROUD and UPC clinics, they will be assigned to the clinic they visited the most, and if tied they will be assigned to the clinic they visit last.

6.3 Exemplar Clinic Sample

PROUD Phase 1 health care systems, including those not in the trial, were invited to participate in an observational component of Phase 2 (see Section 7.3). Several health systems submitted descriptions of PC clinics they perceived as high performing regarding OUD treatment (see Section 3.6.4.4). These could be PC clinics that had implemented systems to increase OUD treatment, such as care managers for buprenorphine or integrated behavioral health clinicians skilled in addictions care, or they might have nearby or co-located specialty addictions clinicians or have linkage to such providers or their clinics, and/or they might have large numbers of PC providers or collocated mental health physicians, nurse practitioners or physician assistants who prescribe buprenorphine. Two health systems that are not participating in the trial are providing data on exemplar clinics. We will also ask them for data on 4 other UPC clinics from their systems for comparison.

The samples for these exemplar clinics will be defined using the identical criteria as the PROUD trial sample (above). This will allow us to compare main and secondary outcomes in PROUD intervention clinics to Exemplar PC clinics.

6.4 Non-randomized UPC Clinic Sample

Existing practice controls, like the UPC clinics used in this trial, have a number of strengths and limitations.[133] These “laissez faire” controls allow comparison to care from health care providers who are not influenced by the research team.[133] However, there are variants to existing practice controls in which they are influenced by the research team in some way. These have been divided into 3 types: constrained (in which usual care restricted in some way); enhanced (in which usual care is augmented in some way); and standardized (in which usual care is systematized in some way).[133]

We intended for PROUD to be completely laissez faire, and designed the trial so that we would have minimal influence on the UPC clinics (e.g., we are not including any baseline interviews or surveys of PC staff). However, during recruitment of clinics in PROUD Phase 1, most of the clinics considered for participation were not able to participate. Inability to participate reflected (1) lack of support for treating OUD in PC; (2) lack of support for participating in the trial, or (3) inability to identify 3 PC clinicians in the clinic who were willing to become waivered and prescribe buprenorphine and naltrexone for OUDs. Thus, we were selecting somewhat atypical sites. Further, the process of recruitment could have enhanced care in the UPC clinics,[133] motivating PC providers to become waivered or clinic leaders to initiate OUD treatment in PC if they are randomized to UPC. In addition, we constrained UPC sites in one way: they cannot be given the MA Model manual from the PROUD intervention clinic and asked to implement the MA Model. In short, the recruited clinics are not entirely typical of true usual care.

To evaluate how much the recruited clinics differed from other PC clinics in the same system, the 6 sites participating in the trial were asked to select 4 additional PC clinics (considered for the trial but not participating) for inclusion as non-randomized UPC clinics. This will allow secondary analyses comparing randomized UPC clinics to more “typical” UPC sites. Five sites were able to do so (U Miami did not have other PC clinics). The non-randomized UPC sample will include patients who receive PC in one of 4 other non-randomized UPC clinics in those 5 sites. The samples for these clinics will be defined using the identical criteria as the PROUD trial sample (above). This will allow us to conduct observational analyses in the 5 sites contributing non-randomized UPC clinics, to compare the 10 clinics in the trial to 20 non-randomized UPC clinics prior to randomization, and to compare the 5 UPC clinics in the trial to 20 non-randomized clinics after randomization. This will allow us to evaluate the generalizability of findings in the UPC Clinics both at baseline and during follow-up.

CONFIDENTIAL

6.5 Secondary "Enrolled" Samples (2 Health Systems Only)

Often pragmatic trials are conducted in populations defined based on health care insurance coverage. Such systems have a number of advantages, three of which might be particularly important in PROUD. First, insured patients can be identified pre-randomization and included in the trial irrespective of whether they have sought PC pre-randomization. Since many patients with active substance use disorders do not seek PC, this might be important for analyses of individuals with OUDs who are not engaged in care pre-randomization. Second, medications dispensed are often available in health insurance plan data (rather than just medication orders from an EHR which might not have been filled). Third, insurance claims are available for all covered services, whether or not services were received in an affiliated health care delivery system. However, only two health systems that were health insurance plans were able to participate in PROUD. In order to determine whether using a visit-based sample, OUD medication orders instead of dispensed medications, and capturing acute care utilization only within a single delivery system for 4 sites (rather than claims for all acute care received), we will conduct secondary analyses of 2 health insurance plans: KPWA and HF.

For these two participating health systems that are health insurance plans (KPWA and HF), we will have a secondary sample of patients enrolled in the health plan (enrollment-based sample). This enrollment-based sample will be used for sensitivity analyses. Patients are considered enrolled if they were enrolled in the health plan and paneled to the participating PC clinic during the 3 years before or 2 years post randomization. These integrated delivery systems and health plans (a.k.a "managed care organizations") have algorithms to assign patients to PC clinics (typically patient selects, if not, the clinic patients visit, if not the clinic nearest their home). We will rely on those assignments made by health plans using algorithms. In rare cases when patients are paneled to multiple clinics, due to changes over time, they will be assigned to the clinic they are paneled to at the time of randomization.

CONFIDENTIAL

7.0 TREATMENT CONDITIONS

7.1 Randomized Clinics

Clinics recruited to be in the trial will be randomized to one of two conditions (UPC and the PROUD intervention) described below. Randomization will be stratified by Health Care System.

7.1.1 PROUD Intervention: Implementation of the MA Model

***Overview and Duration*.** Clinics randomized to the PROUD intervention will be asked to implement collaborative care for OUDs consistent with the MA Model. The intervention is funded for 2 years. This includes 6 months start-up (hiring, training and policy development) and 18 months for induction of an estimated 1-3 patients a week (~75-225 total), as well as continued care with buprenorphine and naltrexone. The intervention consists of 3 strategies used for implementation. These 3 strategies are described immediately below. The detailed outline of each element of the MA Model that will be implemented by PROUD Intervention clinics and the Draft PROUD Manual are described below (Appendix B).

Strategy #1: Providing Funding and Guidance to Hire a NCM. First, Site PIs and health system leaders will be notified they were selected for the trial. A phone meeting will orient them towards hiring even before the in-person kickoff meeting. After kickoff, when clinics randomized to the PROUD intervention will be announced, clinic leaders and providers who have agreed to prescribe buprenorphine and naltrexone will be notified that they will receive resources and support to implement the MA Model. The clinic randomized to the PROUD intervention will be provided funding for 1.0 FTE salary for a NCM for 2 years. The clinic and health system will then recruit, hire, and onboard the NCM, supported by the project. Hiring two NCMs to share a full-time position is also an option.

The nurse care manager (NCM) is a dedicated person in the primary care clinic who works with a group of primary care doctors, nurse practitioners, and physician assistants to support management of patients on buprenorphine and injectable naltrexone for OUDs. Table 5 outlines the roles and responsibilities of the NCM (also included as part of Appendix A).

Strategy #2: Providing Training, TA, and Performance Feedback. Second, the study provides support for training and TA, including monitoring performance and feedback, throughout, provided by the TA team at Boston Medical Center (BMC; C. Labelle, lead).[1,47,73] This includes an**:** a) BMC OBAT Manual (Appendix B), b) sending PROUD NCMs to Boston Medical Center for training, and c) ongoing TA and performance monitoring and feedback to the NCM by the Boston TA team, throughout the study. The TA team is made up of 2 NCMs experienced in implementing the MA Model. Each site will be supported with weekly phone check-ins with the Site PI and project manager during the hiring period from the TA team. The TA team then provides ongoing monitoring and performance feedback to each site weekly to every other week, including two face-to-face site visits from the TA team.

Strategy #3: PC Providers Trained & Mentored. The third strategy is that PC providers obtain training and a DEA waiver to allow them to prescribe buprenorphine, and ensure mentoring from a local or PCSS mentor. Each PC prescriber selects a mentor and the TA team ensures engagement with the mentor during the site visit. Mentors are either local addictions experts in the health system or are available from a national program of voluntary mentors through Providers Clinical Support System for Medication Assisted Treatment (PCSS-MAT).[134] The PROUD NCM also supports them in caring for patients with OUD, including adapting the MA Model Manual to local needs and meeting regularly.

Table 5. NCM Training, Roles, and Responsibilities from Appendix A

Study provides

1. Salary for 1.0 FTE NCM
2. Training and ongoing technical assistance – described below
3. Policy and Procedures Manual developed by MA OBOT team

Training and ongoing technical Assistance (TA)

EARLY IMPLEMENTATION DIDACTIC SESSIONS AND OBSERVATION:

1. All NCMs travel to Boston for 2-day training (all sites can come to training simultaneously or asynchronously). The didactic portions will be available for others.
    a. Day 1: 8 hours didactic/cases/classroom learning (will be available as modules for review later)
    b. Day 2: 4-8 hours of shadowing in BMC OBAT Clinic
2. For any NCM who cannot come to Boston before they start supporting management of OBAT patients can do a 4-hour webinar "Introduction to OBAT." (This will be available as a refresher as well.) In this scenario, subsequent to some patients' enrollment, this NCM will come to Boston for the remainder of the "2-day training."
3. 1 -hour webinar (i.e., Addiction 101) available for other health care team members

ONGOING TA AND CLINIC SITE VISITS:

1. Technical assistance (TA) will occur with MA OBAT team
    - Weekly phone calls with each site (together or separately depending on scheduling) for 6 months, then every other week until end of study, and ad hoc as needed throughout the study.
2. 1st local clinic site visit by a TA team nurse trainer early after start of NCM:
    a. 1 hour all-staff training by TA team nurse
    b. Admin/leadership meeting
    c. One-on-One with NCM, walk through details
    d. Conference call with local or PCSS mentor/warm handoff of mentorship for the MD, NP, PA to the mentor
3. 2nd clinic site visit (optional– joint decision between TA team and local group):
    a. Sit in with patients
    b. Chart reviews
    c. Talk about problems, review cases

Performance/Feedback: Weekly, by end of day Friday (Monday noon at latest)

1. The NCM is responsible for collecting and reporting the following data to the TA team weekly:

- No. of nurse visits that week
- No. of patients screened in the OBAT program and induced within that week
- No. of screeners completed (total and that week)
- No. of new patients assessment/ intakes completed (total and that week, overall and stratified by already a patient in the clinic, from another primary care clinic, or from outside health system
- No. of inductions completed (total and that week)
- No. of follow-up visits (total and that week)
- No. of patients lost to follow-up (also why and what happened)
- No. of injectable Naltrexone injections (total and that week)
- % of patients with unexpected + urine drug tests (UDT) contacted within 7 days (total and that week)
- Death and how (total and that week)
- Returned to care re-engaged with care after left OBAT (total and that week)

2. Clinic Feedback:

Study will support dashboard with all sites' performance (based on above data) by month based on weekly data provided to TA and study. Phone or in person feedback will also be provided by the TA team to the PROUD RN, depending on need.

Nurse: Patient ratio

CONFIDENTIAL

Table 5. NCM Training, Roles, and Responsibilities from Appendix A

Approx. 100 active pts/1.0 FTE NCM (estimated ~2-3 new patients/week, so panel will increase in size over year 1)

Role/Duties
Screen and engage patients, conduct office visits, prepare prescriptions, conduct ongoing monitoring, interact weekly (NCM and prescriber) to review patients. NCM also leads the clinic's buprenorphine program including: maintaining the local annotation of the manual to include all local policies (e.g., policies pertaining to urine drug screens, any added baseline screen or assessment questions, exclusions from primary care OUD treatment, counseling requirements; supervision of other involved medical staff such as Medical Assistant); accounting of number of patients attributed to each physician/NP/PA so as to comply with FDA rules; as well as spreading the word on the availability of treatment for OUD within primary care.<br><br>If buprenorphine or naltrexone are not the right medications for the patient, then the NCM will facilitate referral to methadone maintenance treatment program at a federally licensed opioid treatment program (OTP) as appropriate and available. The NCM will track whether the patient kept the appointment at the OTP.<br><br>In terms of NCM being asked/expected to do non-OBAT care (e.g., flu shots, refills, see non-OBAT patients), each clinic will need to find the balance between the NCM helping out as a team member, so that others cover OBAT care when s/he is away (as appropriate per health system), but remaining dedicated to OBAT activities.
**Registry**
Each system will choose an approach to track patients with OUDs. Templates of required and recommended fields (e.g., for monitoring OBAT population and for patient care) will be provided by the study. Three options 1) use EMR if possible, 2) local database (e.g., excel, access), or 3) develop a clinic registry
**RN Eligibility**
Required:<br>• Registered nurse (RN). This can be diploma, AD, BSN, or MSN. It doesn't have to be BSN unless that is a requirement of the health system for care management work.<br>• Willingness to enroll 2-3 patients per week<br>• Willingness to provide weekly reporting<br>• Willingness for regular interactions with TA team<br>• Enthusiasm and energy/leadership ability to lead OBAT Team and for working with patients with addictions and providers and clinical leaders (e.g. addiction medicine) throughout the health system and community, as relevant.<br>• The FT RN can be split (e.g. 50:50, 40:60) and if the site has a cluster of clinics, the RN can practice at more than 1 site to make the system workable and support the prescribers if they are at multiple sites. If the position is split, the RNs will do weekly reporting jointly to the TA team (as if one site) and both will attend training in MA<br>• Preferred qualifications: prior behavioral health or addiction experience and adequate experience to function as the nurse manager of this program. (Nurses are hired as nurse managers of this program.)

7.1.2 Usual Primary Care (UPC)

As above, most individuals with OUDs never receive evidence-based medication treatment and most PC practices do not directly provide treatment for OUDs. The PROUD trial is designed to evaluate implementation and patient outcomes of the MA Model when implemented in diverse health systems. Therefore, it is appropriate to evaluate the impact of the MA Model on OUD treatment and patient outcomes, compared to UPC.

UPC clinics will not receive the PROUD Manual for OBAT, and will not have any interaction with TA. There will be no suggested or required enhancement in OUD care in the UPC clinics. This includes no requirement that any PC providers in UPC become waivered, as that is part of the intervention and not part of UPC. At the same time, they will be free to implement any quality improvement program they wish for treating OUDs, with the exception of using the MA Model manual in the UPC clinic. Care processes regarding OUD care in UPC clinics will be assessed at baseline and changes monitored over time by Site PIs and project managers via key stakeholder interviews at least every quarterly, as described above.

7.2 Non-Randomized Usual PC Clinics

Five sites participating in the trial agreed to obtain data from 4 non-randomized PC clinics for secondary observational analyses. These will be used in preliminary analyses to assess the generalizability of the selected clinics that are participating in the trial. The 4 clinics not in the trial will be randomly selected from among the PC clinics at each of the 5 sites.

7.3 Non-Randomized Exemplar PC Clinics

PC clinics that may provide excellent access (i.e., high rates of initiation) and/or quality (i.e., high rates of retention in treatment) of treatment for OUDs were identified during Phase 1 of PROUD. These will be detailed in the SAP but include a total of 10 clinics in 4 health care systems, including two sites that are in the trial:

- KPCO (4 clinics including an innovative “rapid start” program for OUD and other addiction treatment, two PC clinics with collocated addiction treatment and one with addiction treatment across the street),
- KPNW (1 clinic that consists of co-located addictions and PC with 8 buprenorphine prescribers),
- MF (3 clinics including one with a clinical pharmacist coordinator who serves 1000 patients on buprenorphine, 2 other PC clinics that share a NCM), and
- KPWA (2 clinics, one with a goal that all providers prescribe and one rural site that hired a NCM after discussions regarding the PROUD trial).

CONFIDENTIAL

8.0 MEASURES

Table 6 outlines domains of measurement and sources. Measures will be obtained predominantly from EHRs, health system administrative datasets and insurance claims (CLM) data (in the 2 sites that are insurance plans—KPWA and HF), when available. Other measures come from data collected by TA team (weekly enrollment reports) or weekly debriefs of the TA team by the Lead team. Finally, debriefs with each Site PI, project manager and programmer will provide additional data.

Table 6. Measures: Overview

	EHR	ADM	CLM	TA	DB
**Main Outcomes**					
Primary: days of buprenorphine or injectable naltrexone	X				
Secondary: acute care utilization	X		X		
**Additional Outcomes**					
Implementation outcomes	X	X			
Patient outcomes	X		X		
Processes of care	X			X	
Socio-demographics and covariates	X	X	X		
**Other Variables**					
Implementation fidelity measures	X			X	
Description of clinical practices in UPC clinics					X
Description of clinical practices in Exemplar clinics					X

ADM=administrative data; CLM=insurance claims; TA = weekly debriefs and reports from technical assistance team; DB=debriefs with site teams

8.1 Primary Objective Outcome Measure: Patient Days of OUD Treatment

The number of patient days of OUD medication treatment documented in the EHR in each clinic in the 2 years post-randomization is the primary outcome measure. "OUD medication treatment" includes medications for OUDs that are prescribed in PC and documented in EHRs—buprenorphine (with or without an OUD diagnosis) or injectable naltrexone with an OUD diagnosis. An OUD diagnosis is required for injectable naltrexone because it is often used for alcohol use disorders. We do not require an OUD diagnosis for buprenorphine because PROUD Phase 1 analyses revealed OUD diagnoses are often missing, consistent with the literature,[50] and an OUD diagnosis is likely to be documented by PROUD clinic providers based on the PROUD NCM manual, so that requiring an OUD diagnosis could bias findings toward favoring the intervention clinics. To account for varying clinic sizes, the outcome will be reported as the number of patient days of OUD treatment in the 2 years after randomization per 10,000 patients seen in the clinics during that time period.

This outcome was selected as our measure of the success of implementation because investigators believe that it will be compelling to clinical leaders, as it reflects both initiation (reach) and/or retention (implementation effectiveness). Further because it is scaled it provides excellent statistical power for a relatively small pragmatic trial, where the size of the proposed trial is limited due to the cost of supporting a NCM at each site for 2 years.

Although final definitions and algorithms used to define this outcome will be refined in the SAP, we provide a brief overview here. Two FDA-approved treatments for OUDs that can be provided in medical settings and documented in EHRs are included as OUD treatment. Buprenorphine and injectable naltrexone use will be determined from the EHR orders and procedure codes (for injections). National Drug Codes (NDCs), Healthcare Common Procedure Coding System (HCPCS) codes, and text string searches on medication name will be used to ascertain buprenorphine and injectable naltrexone use from medication orders and procedure data on injectable medications. Pharmacy dispensings are the gold standard for outpatient non-injectable medication use but only medication orders, not pharmacy dispensings, are available for 4 sites that are not health plans and do not have their own pharmacies. To make the outcome comparable across sites, we therefore use orders only for our primary analyses. We will build on the algorithms developed in Phase 1, which are summarized in Appendix C, to estimate days of OUD treatment (updated algorithm will be specified in the final version of the standalone SAP). A single order or procedure code for buprenorphine or injectable naltrexone (the latter with an OUD diagnosis) is considered OUD treatment. Although other studies have assumed single orders (without a refill) are tapers,[36] tapers will be uncommon in the intervention arm. Therefore, assuming single fills are treatment (conservatively) biases the PROUD trial to the null.

For the diagnosis of OUDs required with injectable naltrexone the exact timing and number of the documented OUD diagnoses, and whether the number and timing of documented alcohol use disorders diagnoses should be taken into account, will be developed for the SAP, defined based on preliminary Phase 1 analyses. Appendix C provides further details on the main outcome at the time of DSMB review September 2017.

Only 3 sites (KPWA, HF, and MF) are able to provide claims for outside pharmacies or OUD treatment, including outside methadone treatment for OUDs from OTPs. These data will be used for sensitivity analyses including the 3 health care systems able to obtain it. This measure is ascertained from utilization data for sites that have internal methadone OTPs (only MF) and claims data using International Classification of Disease (ICD) procedure codes for KPWA and HF. Days of methadone treatment are estimated based on the interval between claims if 3 or more claims are available or the average interval for the site's first pair of claims if only 1-2 claims, although the exact algorithm for estimating days of treatment in an OTP may be refined in the SAP based on Phase 1 analyses. Because location could bias results (if one clinic from a site was near a methadone OTP and another was not), we will evaluate the distance from each clinic to the nearest 3 OTPs as a possible covariate in secondary analyses including methadone treatment for OUDs.

8.2 Secondary Objective Outcome Measures: Acute Care Utilization

The secondary endpoint is a count measure of the number of days of acute care utilization in the 2 years after randomization. This measure includes visits to urgent care clinics or emergency departments (EDs), as well as days hospitalized. As above, acute care utilization will be determined from EHR and insurance claims data when available (Table 6). Each day with a visit to an urgent care or ED will be counted as 1 day (even if the patient stays overnight). For hospitalizations, the number of days will be the number of days from admission to discharge, inclusive. If a patient is admitted from urgent care or an ED, the ED or urgent care day is not (double) counted. Final definitions and algorithms used to define this outcome will be provided in the SAP.

CONFIDENTIAL

Table 7. Additional Outcomes: Assessed in the 2 Years After Randomization.

**Additional Implementation Outcomes**	
**Newly recognized OUDs** (Reach)	Number of patients* with a new ICD code for OUD during follow-up who did not have an OUD diagnosis in the three years prior to randomization
**Initiation of OUD treatment** (Reach)	Number of patients* who initiate buprenorphine or injectable naltrexone with an OUD diagnosis (> 28 days) during follow-up: any initiation and separately for initiation of each type of medication.
**Retention in OUD treatment** (Implementation effectiveness)	Number of patients* initiating OUD treatment during follow-up who receive OUD treatment on 80% of days available after initiation and median days of OUD treatment after initiation for patients with at least 6 and 12 months available for follow-up. (Patients not retained in treatment per the EHR are considered no longer in treatment, an added secondary outcome).
**Contiguous days in treatment** (Implementation effectiveness)	Days of OUD medication treatment with no gap in orders or refills exceeding 60 days[73] will be used to assess retention as well
**Cross-over between clinic arms**	The number of patients with OUDs assigned to each clinic (PROUD and UPC) in the 3 years prior to randomization who are seen in the other clinic post randomization,
**Cross-over between systems** (patients with OUD WA only)	The proportion of patients treated for OUDs in each clinic who have insurance from another health system (i.e., patients seen at Multicare who have KP insurance and patients seen in KP Washington who have outside insurance).
**Prescribing providers** (Adoption)	Number and % of PC providers who order buprenorphine or injectable naltrexone for at least 2 patients with OUDs
**OUD treatment starts per week** (Implementation fidelity)	Mean number of patients initiating buprenorphine or injectable naltrexone per week over the 2 years post randomization
**Time to OUD treatment** (Implementation fidelity)	Median number of days (0 days if same day to infinity if untreated) from first visit with a new OUD ICD diagnosis (no prior diagnosis) to OUD treatment initiation during follow-up
**Urine drug monitoring** (Implementation fidelity)	Median frequency of urine drug testing in the 1 and 3 months post initiation of OUD medication treatment. (Note: Although this aspect of the MA Model is specified locally because there is no scientific consensus on the optimal algorithm, urine monitoring early in treatment is recommended)
**Re-initiation of OUD treatment** (Implementation fidelity)	Proportion of patients with prior EHR documentation of OUD treatment followed by at least a 3-month gap in treatment, who re-initiate buprenorphine or injectable naltrexone
**Methadone OTP**	Proportion of patients who are receiving methadone maintenance (restricted to 3 sites with OTP data)
**Buprenorphine dose**	Highest mean daily buprenorphine dose in any month of buprenorphine treatment (patient-level measure)
**Additional Patient Outcome Measures**	
**Urgent care or ED use** (Secondary patient outcome)	Number of visits to urgent care or EDs during follow-up among patients with an OUD diagnosis
**Inpatient Days hospitalized** (Secondary patient outcome)	Number of days hospitalized during follow-up, among patients with an OUD diagnosis
**Opioid overdose** (Secondary patient outcome)	Proportion of patients with an ICD code for opioid overdose during follow-up (Note this is expected to be biased due to improved ascertainment in the PROUD clinics due to NCM follow-up with patients with OUDs).
**HCV viral cure** (Secondary patient outcome)	Number of patients with HCV who have documented viral suppression among all with diagnosed chronic active HCV.

CONFIDENTIAL

Additional Outcome Measures - Other Processes of Care	
**New diagnosis or treatment of other substance use condition**	Number of patients who have a new diagnosis or treatment for another substance use condition (not diagnosed prior 2 years)
**New diagnosis or treatment of mental health conditions**	Number of patients who have a new diagnosis or treatment for a mental health condition (not diagnosed prior 2 years)
**Naloxone prescribing**	Number of prescriptions of naloxone for OD management among patients with OUD diagnoses during follow-up
**HCV & HIV Screening or treatment**	Number of patients with new OUD diagnoses who have screening tests or treatment for HCV or HIV

** Measured in the 2 years after randomization using EHR data, reported per 10,000 PC patients in a clinic in the 3 years prior to randomization.*

8.2.1 Patients with Recognized OUDs

Our main secondary objective evaluates acute care outcomes in patients with recognized OUDs, based on EHR documentation, in the 3 years prior to randomization. Recognized OUDs will be defined as any ICD code for an OUD during the study period. Final definitions will be specified in the final SAP based on preliminary analyses.

8.3 Additional Quantitative Outcome Measures

8.3.1 Outcomes for Comparing PROUD Intervention and Exemplar Clinics

Outcome measures for comparisons of randomly selected PROUD intervention clinics to Exemplar PC clinics during the 2 years of trial follow-up will be the same as the primary outcome and select secondary trial outcome measures above.

8.3.2 Outcomes Reflecting Implementation, Patient Outcomes and Care Processes

Additional explanatory secondary outcome measures are defined in Table 7, reflecting implementation, patient outcomes and other care processes potentially impacted by the PROUD intervention. Definitions and algorithms used to define the outcomes will be provided in the SAP. In addition, to help understand the risk of "bleeding" of the intervention from PROUD clinics to other clinics, programmers at each site will monitor and report any movement of providers across primary care clinics every 6 months, in order to identify whether primary care providers move between randomized PROUD and UPC clinics, and especially if PROUD clinic primary care providers who prescribe buprenorphine, or other staff, move to randomized UPC clinics.

8.3.3 Implementation Fidelity Measures (PROUD Intervention Clinics Only)

The NCM is responsible for collecting and reporting data to the TA team and the Lead Node weekly. Each Friday each PROUD NCM will report to TA Team on their work from the prior week (Friday through Thursday) and since study start. Measures reported are shown in Table 8. These data will also be maintained by the NCM(s) in the local patient registry.

8.4 Covariates and Measures for Describing Study Sample

Measures in this Section are used to characterize the study samples and variation across clinics at baseline, and used as covariates for adjustment as appropriate. Data on measures will be obtained for the entire study period (3 years prior to randomization through 2 years following randomization).

8.4.1 Sociodemographic Measures

Sociodemographic characteristics available for this study include: age at randomization, sex as documented in the EHR (male, female, other, unknown); race/ethnicity (Asian, Black or African American (non-Hispanic), Hispanic, White (non-Hispanic), American Indian/Alaska Native, Native Hawaiian or other Pacific Islander, other, more than one, unknown); zip code; and type of insurance (uninsured, self-pay, state subsidized, private insurance, Medicare). Details of the covariates may be refined based on ongoing Phase 1 analyses and preliminary (3 year "baseline" period) trial analyses will be included in the SAP.

Table 8. Weekly NCM Reports to TA Team

Number of patients:<br>• Screened by NCM*<br>• New patient assessments*<br>• New patients to the clinic<br>• New patients to the health system<br>• Buprenorphine inductions*<br>• Started on injectable naltrexone*<br>• Follow-up visits per week<br>• Total with NCM phone or in-person visits per week<br>• Lost to follow-up (reason, etc.)<br>• Unexpected urine drug tests<br>• (%) contacted within 7 days<br>• Deaths and cause of death<br>• Re-engaged after left OUD care

* per week and to date

8.4.2 Measures of Medical, Mental Health and Substance Use Disorders

The following measures based on ICD codes (unless noted) will be used.

- <u>Measures of medical conditions</u> include: HIV, HCV (both ICD codes and laboratory tests) chronic pain, and all of the individual diagnoses included in two commonly used comorbidity indices below.[135-139] Measures of non-cancer chronic pain are broken into the following pain categories: arthritis, back pain, neck pain, chest pain, limb pain, neuro pain, headache, fibromyalgia, abdominal pain, pelvic pain, general pain, and a composite of any chronic non-cancer pain.
- <u>Mental health measures</u> include: depressive disorders (e.g., major depression, persistent depressive disorder, mood disorder NOS); anxiety disorders (Panic, GAD, OCD, other anxiety), trauma & stressor related disorders, including PTSD; serious mental illness (bipolar, schizoaffective disorder, schizophrenia, other psychotic disorders); attention deficit disorders; eating disorders, and neurocognitive disorders.

- Substance use disorders include both detailed opioid diagnoses (active, remission, overdose and dates of each) and other substance use disorders broken down by: tobacco use disorder, alcohol use disorder, cannabis use disorder, stimulant use disorder, sedative-hypnotic use disorder, other substance use disorder (non-tobacco, non-alcohol, non-cannabis, non-opioid, non-sedative hypnotic, and non-stimulant use disorder).

8.4.3 Comorbidity Indices

Patient outcomes are impacted by concurrent comorbidity. The two most commonly used comorbidity indices will be used to characterize the PROUD intervention and UPC clinics in the 3 years prior to baseline and adjust for baseline differences if necessary: the Charlson Comorbidity Index and the Elixhauser index.[135] The Charlson was developed to predict 1-year mortality among patients admitted medically to an inpatient stay, while the Elixhauser was developed to predict hospital charges, length of stay and in-hospital mortality.[136,137]

8.4.4 Process Measures of Possible Cross-over Between PROUD and UPC Clinics

In addition to process measures of cross-over in patients with OUDs (now in Table 7) we will monitor cross-over in general. Beginning 3 years prior to randomization and every 6 months after randomization the overall number and proportion of patients assigned to each clinic who visit the other randomized clinic at the same site will be assessed (as context for cross-over in patients with OUDs Table 7). In WA state, where 2 health systems are participating in PROUD (KPWA and MC), we will also assess cross-over from KPWA to MC PC clinics using KP claims data in patients in general, as context for cross-over (Table 7) in patients with OUDs.

8.4.5 Baseline Measures of Generalizability of PROUD Trial Clinics

Two randomized and 4 non-randomized clinics from the 5 PROUD trial sites able to provide non-randomized usual care clinics will be compared in the period before randomization to evaluate the degree to which the clinics in PROUD are representative of other PC clinics in each health system. Pre-randomization measures will be identical to primary and secondary outcomes above except that they are measured during the 3 years prior to randomization (Table 9). Other pre-randomization measures include: demographic characteristics (age, sex, race/ethnicity); insurance status (uninsured, state subsidized insurance, private insurance, private pay and Medicare); and the prevalence of OUDs, other substance use and mental health disorders, and chronic pain syndromes; medical comorbidity (Charlson and Elixhauser indices), and the prevalence of prescription opioids (low, medium and high dose) in the PC samples.[135-137]

Table 9. Example of Secondary Pre-randomization Measures for Comparison of 2 Randomized Clinics to 4 Non-randomized UPC Clinics in the Same Health Care Systems	
Recognition of OUD Before Randomization	Number of patients with an ICD code for OUD per 10,000 patients seen during the 3 years prior to randomization
Initiation of OUD treatment before Randomization	Number of patients who meet criteria for OUD treatment above in the 2 years prior to randomization, with no OUD treatment in the preceding year (per 10,000 patients seen)
Retention in OUD treatment before Randomization	Number of patients initiating OUD treatment in the 2 years prior to randomization who receive OUD treatment on 80% of days between initiation and randomization or 365 days whichever comes first

8.4.6 Mortality

Death data during the study will not be complete due to lags in obtaining death data from the state. Moreover, death data are expected to be potentially biased during follow-up, with increased ascertainment within the EHR in the PROUD intervention clinics due to NCM follow-up. However, we will obtain death data from the EHR and administrative data and use them for descriptive purposes, to inform the ancillary study of mortality.

8.5 Qualitative Measures

The details of methods and domains for implementation monitoring will be included in the final version of the standalone SAP. Two broad areas are included.

8.5.1 Description of the 12 PROUD Trial Clinics Pre-randomization

Prior to randomization, Site PIs will complete a questionnaire about their health systems primary care clinics and OUD care in the clinics based on their knowledge of the health system supplemented with stakeholder interviews as needed (some Site PIs are leaders and/or PC clinicians in their health systems whereas others have no clinical involvement). Measures of baseline care in the 12 PROUD trial clinics (both PROUD Intervention clinics and UPC clinics) fall into 3 domains: the PC clinics (possible recipients of the intervention), the health system infrastructure, and the external environment (Figure 2). Aspects of PC clinics assessed will be current staffing, current procedures for providing mental health care generally, OUD treatment options for patients seen in the clinic, including prescription of buprenorphine or injectable naltrexone, as well as methadone in OTPs, and how a patient would access those treatment options.

8.5.2 Barriers and Facilitators to OUD Treatment in PC

Qualitative data on barriers and facilitators to OUD treatment in PC during the trial will be obtained from debriefs regarding barriers and facilitators to OUD treatment in PC. Specifically, the Implementation Monitoring Team led by Dr. Bradley and Amy Lee, MPH—an early career investigator with implementation research and interview expertise—will debrief the TA team about barriers and facilitators for PROUD intervention clinics monthly, as well as having a member of the Implementation team listen to all TA calls with NCMs. The Implementation Monitoring Team will also debrief Site PIs and project managers about any barriers and facilitators they have heard about in the PROUD intervention or UPC clinics every 12 weeks. Qualitative data on barriers and

facilitators will be coded into domains from the upper portion of the PRISM model: the intervention; the PC clinics (recipients of the intervention); the health systems infrastructure that impacts implementation and sustainability; and the external environment.

8.5.3 Plans for Sustainment

At the time of site recruitment, PROUD Phase 1 sites were concerned about sustainment of the program after the trial. We provided sites with information about the new Medicare codes which pay for nurse care managers (NCMs),[78] which will help cover the NCMs after the trial. Nevertheless, we expect that some sites will have challenges continuing the program. Therefore, qualitative data on plans for sustainment will be obtained during Year 2 of the intervention period. This will include: the roles of leaders who decide to continue or stop the program; information that was important to them in making that decision; mechanisms for paying for the NCM after study end; and barriers to sustainment in settings in which the program was not sustained.

CONFIDENTIAL

9.0 DETAILS OF STUDY PROCEDURES

9.1 Overview of Organization and Leadership

Figure 1 outlines the 4 periods of the study: Period 1 – Start-up Prior to kickoff; Period 2 – PROUD Intervention Implementation; Period 3 – Ongoing TA and Data Collection; and Period 4 – Analysis and Dissemination. Activities during these 4 periods are outlined in this section.

The PROUD trial will be led by the Lead Node Team (LNT). The LNT will plan agendas for weekly All Site Meetings (described below). Additional PROUD Teams include: Administrative, CTN Operations, Implementation Monitoring, Intervention, Data & Analytics, and Publication Teams. These are shown below with the leadership of each, along with each team's membership and team responsibilities (Figure 3).

Figure 3. Organization of PROUD Trial

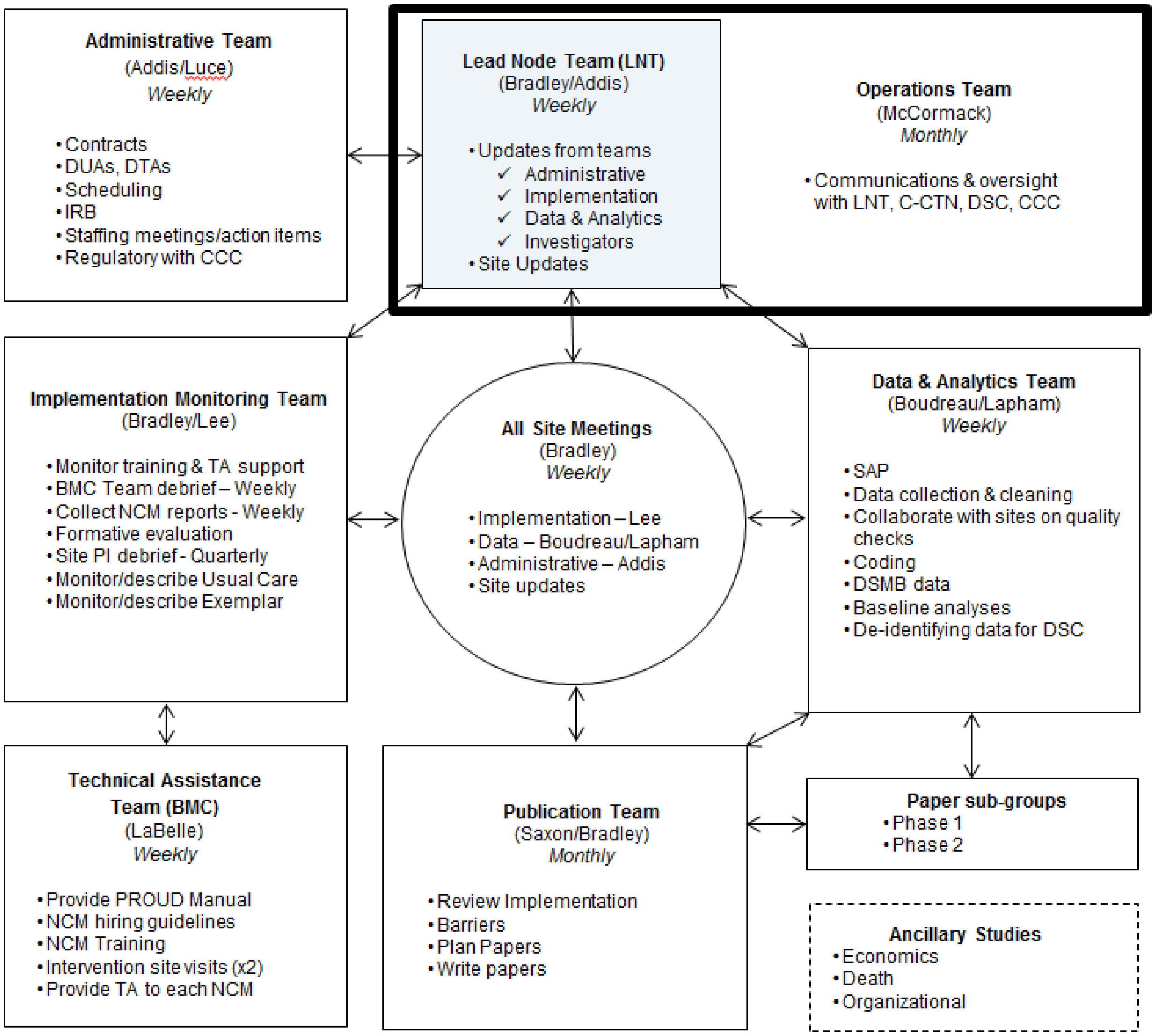

9.2 Study Period 1: Start-up Prior to Kickoff

9.2.1 Lead Node Team (LNT) Meetings Begin

Weekly meetings of the LNT will begin immediately, with weekly reports from the leaders of each PROUD Team (above). Monthly, the full CTN Operations Team (C-CTN, DSC, CCC, and Lead Node investigators) will meet with the LNT for a higher-level update. These teams have been working throughout Phase 1 of PROUD and have developed strong working relationships.

9.2.2 All Site Meetings Begin

We will have an All Site Meeting weekly that involves participation from the Site PI, site project manager and site programmer from each site, as well as representation from all other PROUD teams above. These weekly calls will begin immediately and will be led by Dr. Bradley, with agendas planned by the LNT. The major purpose of these calls will be to report each site's progress and trouble shoot barriers. Each week the agenda will include status updates for all sites covering administrative, programming and implementation issues, followed by discussion. During this "Start-up Prior to Kickoff" study period, the main focus will be on administrative issues (contracts, DUAs, ceding to the central IRB and preparing to hire an NCM) as well as data & analytics (preliminary analyses).

9.2.3 Administrative Start-up

During this phase, the LNT and CTN Operations Team will present to the DSMB for scientific review and make necessary revisions to the protocol; prepare all documents for the central institutional review board (C-IRB) and lead the sites in ceding to a C-IRB; prepare DUAs and DTAs for sharing data with the DSC and the contractor that manages NIDA's Data Share website. The DSC will be involved at all review discussions. Once the trial is approved by the reviewers on the DSMB and NIDA CTN leadership, the PROUD trial will be registered on the clinicaltrials.gov website. The subcontract with the Technical Assistance Team in Boston will also be executed.

9.2.4 Implementation Monitoring Start-up

The Implementation Monitoring Team will collect standard qualitative and descriptive baseline (pre-randomization) data on addictions and OUD care in all randomized and non-randomized clinics, and develop processes and procedures for all other qualitative and quantitative data collected during the 2-year implementation phase. The Technical Assistance Team in Boston will prepare all materials to facilitate hiring and training of the NCMs.

9.2.5 Data & Analytics (D&A) Team – Preliminary Analyses and SAP

The D&A Team will continue developing the detailed standalone SAP, including preparing code describing the sample of patients who visit all participating PC clinics (non-randomized and randomized) in the 3 years prior to randomization including all outcome measures and covariates described above. The NIDA DSC will randomize clinics participating in the trial (and conceal results) after all contracts, C-IRB and DUAs are approved. The analytics team will also prepare data for manuscripts from Phase 1 for the Phase 1 papers.

9.2.6 Publication Team

PROUD investigators will participate in meetings at least monthly to prepare Phase 1 manuscripts.

9.3 Study Period 2: PROUD Intervention Implementation

Study Period 2 lasts 6 months and includes: a kickoff meeting; beginning the intervention, baseline data collecting and SAP development. Beginning the intervention includes announcing which clinics receive a NCM, supporting PC prescribers in becoming waivered, supporting hiring of NCMs, NCM training in Boston, and supporting development of any local buprenorphine and naltrexone policies.

9.3.1 Kickoff Meeting in Seattle

We will hold a 1.5-day kickoff meeting in Seattle attended by the LNT, CTN Operations Team, Site PIs, site project managers, site programmers, the Technical Assistance Team from Boston, and physicians with experience implementing the MA Model (Drs. Samet, Tsui and Merrill). Randomization will be conducted after: central IRB approval is obtained for the trial; all sites cede; the protocol is placed in ClinicalTrials.gov, and full funding for the NCMs year 1 has been received by the Lead Node. If possible, the results of randomization will be unveiled at the Kickoff. Irrespective, the protocol will be reviewed as well as information on the hiring and training of NCMs and all data collection protocols (weekly summary of NCM activities per Table 8, required Site and Intervention Team debriefs and rationale, timing and documentation of EHR data collection, and timelines for all required deliverables). Organization of the trial will be reviewed as well as communication plans between the LNT and sites.

9.3.2 Site Teams

During Period 2, the clinics randomized to the PROUD intervention will be provided funding for the NCM. The sites will hire the NCM as soon as possible within 1-6 months (ideally someone experienced in addictions treatment or mental health care). The sites ensure the following take place: credentialing; arranging local supervision for the PROUD NCM; and developing a system for a registry in or outside the EHR. The site sends the PROUD NCM to Boston to be trained by the Technical Assistance (TA) team and begins OUD treatment in the PROUD PC clinic according to the MA Model Manual. During this time the NCM works with prescribers to define local policies (e.g., regarding urine screens demonstrating drug use). PC providers who will prescribe buprenorphine obtain DEA waivers to prescribe buprenorphine if they do not have one already and identify their mentors (locally or virtually via PCSS). Sites begin sending reports to the TA Team and Lead Node Team weekly.

9.3.3 Technical Assistance Team

As soon as a NCM is hired by a PROUD intervention clinic, s/he begins meeting weekly with the Technical Assistance Team, and receives a PROUD OBAT Manual (Appendix B) from them. NCMs are trained by the Technical Assistance Team in Boston for 2 days, scheduled ~4 months after randomization. Soon after the start of OUD treatment in each clinic, the Technical Assistance Team will conduct a site visit to orient other members of the PC clinic and ensure linkage to the mentor for the prescribing PC providers. The Technical Assistance Team supervises the PROUD NCMs weekly initially (and later every other week as needs for technical assistance decrease).

9.3.4 Implementation Monitoring Team

Weekly debriefs of the Technical Assistance Team by the Implementation Monitoring Team will begin early in this period when the NCMs are being hired. Barriers to implementation (e.g., a NCM is not screening patients or a prescriber is not prescribing) are problems solved during the debriefs, as well as during the All Site Team meetings and the LNT meetings, as necessary.

9.3.5 Data & Analytics Team

During Period 2, the data core in Seattle obtains all data for the sample, outcomes, and covariates from all sites for the 3 years preceding randomization (both the 2 randomized clinics and non-randomized clinics from 5 of the 6 sites in the trial), and conducts secondary analyses comparing clinics in the trial to other non-randomized clinics in the 3 years prior to randomization (both Exemplar and UPC).

9.4 Study Period 3: Ongoing Technical Assistance and Data Collection

9.4.1 Technical Assistance Team

All PROUD intervention clinics are expected to have implemented the MA Model by the beginning of Period 3. Sites will be supported by the Technical Assistance Team, participate in ongoing data collection over the 1.5 years of Period 3, and participate in continued weekly All Site Team meetings. During follow-up, the Technical Assistance team supervises the PROUD NCM every 1-2 weeks, and sites continue to send weekly data to the Technical Assistance Team and the Implementation Monitoring Team regarding the number of new patients who have started OUD treatment, etc., as shown in Table 8. A second Technical Assistance Team site visit will occur at a mutually agreed upon time (and is optional).

9.4.2 Implementation Monitoring Team

The Implementation Monitoring Team will debrief the Technical Assistance Team every week. As above, these debriefs will be used to problem solve barriers and adapt implementation in PROUD intervention clinics as needed (e.g., formative evaluation). Implementation Monitoring Team will also debrief Site PIs and project managers quarterly to identify barriers and facilitators from their perspective in the PROUD intervention clinic and any changes in their UPC clinic or more broadly in their health system. During this period, the Site PI and project manager at each of the 6 sites will conduct formal interviews with key informants in their health systems quarterly to monitor changes over time in the health care system's usual PC for OUD. Based on notes from those meetings, the barriers and facilitators identified will be relayed to the Implementation Monitoring Team at the next debrief, and the Implementation Monitoring Team will maintain a database of all data collected on the PROUD Intervention in the 6 PROUD health systems, coded into barriers and facilitators in an iterative process.

9.4.3 Data & Analytics (D&A) Team

Every 6 months after randomization, secondary EHR data (and claims data when available) will be obtained for the period from 3 years prior to randomization to the date of extraction (6, 12, and 18 months after randomization) and prepared for the investigators and DSMB (Table 10) which is monitoring data quality as the trial progresses. The DSMB data quality report will include the sample to date (number of patients to date who will contribute primary and secondary outcomes, demographics, % OUD, and % with any OUD treatment at each site). During this period, the D&A Team will conduct preliminary data analyses to inform the SAP, as well as descriptive analyses of the study sample pre-randomization. These analyses result in a finalized SAP, as well as manuscripts on the baseline sample and comparison of PROUD trial, exemplar, and usual care clinics at baseline (prior to randomization).

CONFIDENTIAL

Table 10. Data Reporting to NIDA CTN's DSMB Which is Monitoring Data Quality

Data on the Size and Composition of the Study Sample		
**Period of data**	**When Data are Extracted**	**Available for Presentation to NIDA CTN's DSMB Which Monitors Data Quality**
-3 to 0 years	0-3 months after randomization	6 months after randomization
0-6 months	6-9 months after randomization	12 months after randomization
7-12 months	12-15 months after randomization	18 months after randomization
13-18 months	18-21 months after randomization	24 months after randomization
19-24 months	24-27 months after randomization	30 months after randomization
0-2 years	30 months after randomization	36 months after randomization

Randomization = time "0"

9.5 Study Period 4: Analyses and Dissemination

Final data collection will occur 2.5 years after randomization to allow 6 months for the lag in claims data after the end of the 2-year intervention, to collect all data needed for the main analyses and sensitivity analyses. An analytic de-identified dataset for the primary objective will be prepared for the DSC before 32 months after randomization. The DSC will conduct the primary analyses for the primary objective. The Lead Node biostatistics team will conduct all other analyses including the secondary objectives. Manuscripts on main findings will be prepared and submitted. Main analysis and manuscript preparation will occur during the last 6 months.

Dissemination will include academic papers outlined in Table 11 as well as dissemination via webinars such as the NIH Collaboratory Grand Rounds, the Health Care System Research Network (HCSRN) Learning Health System webinars and federal partners such as CSAT and SAMSHA (we have already had a joint CSAT-CTN webinar on the design in Fall 2016).

Table 11. Planned Manuscripts

1. *Baseline Measures of Generalizability of PROUD Trial Clinics: comparison to Other Clinics in the Same Health Systems on primary and secondary outcome*
2. *Main Results: Primary Objective*
3. *Main Results: Secondary Objective*
4. Comparing PROUD Intervention and Exemplar Clinics
5. Differences in PROUD Primary and Secondary Outcomes across Age, Sex and Racial/Ethnic Groups

10.0 DESIGN CONSIDERATIONS

The PROUD study was designed to be generalizable to diverse healthcare systems and to inform health system leaders on how implementing the MA Model might increase OUD treatment and decrease acute care utilization in "real world" settings. The design has a number of strengths. First, we recruited a pair of PC clinics from diverse health systems: safety net clinics (MF and HH); an academic clinic (UM); integrated delivery systems with health care delivery and health insurance combined in a single organization (HF and KPWA); and a regional healthcare delivery system (MC). Moreover, these systems are geographically diverse and we will use secondary data to evaluate outcomes in a generalizable sample of patients aged 16 to 90 years old. Second, all 6 health care systems agreed to implement the MA Model in a randomly selected clinic, out of a pair of clinics they provided. Third, all systems have a demonstrated ability to provide data on buprenorphine and injectable naltrexone orders or dispensed medications, all diagnoses, and all health care utilization in their health care system. At the same time, the design also has limitations; we address major limitations below.

10.1 Design Considerations: Intervention and Control

10.1.1 Choice of the Intervention

Although the MA Model has an outstanding track record of engaging and retaining PC patients in OUD treatment, dissemination of the MA Model could be hampered by several factors. First, it requires a large upfront investment in a fulltime NCM, and there are often regional shortages of nurses, which might make hiring difficult in some locations, especially with the required skills and openness to treating OUDs. Ideally, we could have tested a more flexible, lower cost model as well. However, that was not feasible for budgetary reasons. ***To address this limitation we will compare the MA Model to 10 other exemplar clinics in secondary observational analyses described below in Section 11.3.3.***

10.1.2 Usual Care Controls

Use of a usual care control has a number of strengths for this study, as outlined above, but also poses a number of challenges.[140] These include diversity of UPC across sites and clinics at baseline and over time, and the challenge of defining what usual care includes at each site. We address these with robust assessment at baseline and during the study by monitoring OUD care in all clinics with qualitative and quantitative data. Qualitative data to be collected might include new leadership or behavioral health clinicians in PC, or changes in requirements for prior authorization of injectable naltrexone, and whether or not care management for mental health or substance use disorders is available in a UPC or intervention clinic. Quantitative data to be collected include the number of PC providers total, number of PC providers who prescribe buprenorphine, number of integrated behavioral health clinicians, and movement of providers between clinics (PROUD, UPC, and non-randomized UPC. At the same time we avoid any contact between the investigators and PROUD Intervention or UPC clinic staff, and the site lead investigator and project manager will only check in with 1-2 key stakeholders quarterly about changes in usual care over time, to avoid contamination of the UPC clinics and to increase generalizability.

10.2 Design Considerations Regarding Study Samples

10.2.1 Recruited Clinics Are Not "Usual"

During site engagement in Phase 1, most clinics could not participate due to leadership at some level not wanting to treat OUDs in PC (i.e., leaders at the highest level of the organization, regional leadership, addictions leadership, PC leadership or clinic leadership) or the absence of 3

clinicians willing to prescribe buprenorphine. Thus, UPCs in the trial are not typical. To address this we have asked all sites to provide data on 4 non-randomized PC clinics and five sites have said they are able to do so. We will also ask 2 other health systems providing exemplar PC clinics, to also provide 4 UPC clinics. Secondary analyses comparing the 12 PROUD trial clinics to these other usual care clinics prior to baseline, and comparing outcomes in the randomized UPC clinics and in these non-randomized UPC clinics, will inform generalizability of the results of the trial to other settings.

10.2.2 Imbalance in the Size of the Two Randomized Clinics at Each Site

Imbalance in the size and other characteristics of the 2 clinics at each site could bias the study. The NCM being hired through the PROUD intervention has a finite capacity to treat approximately 100-125 total patients based on experience in MA. Because our main outcome is reported per 10,000 patients in the clinic pre-randomization, if 2 clinics each treated 100 patients for OUD, but one saw 20,000 total patients and the other saw 10,000 total patients in the year prior to randomization, the patients per 10,000 treated per clinic would be 50 or 100, respectively. However, none of the clinics has more than a 2-fold difference in size and we hypothesize a 5-10 fold increase in days of buprenorphine treatment (reflecting the number of patients who initiate as well as improved retention of patients in treatment). Therefore, the relatively small imbalance is thought acceptable.

10.2.3 Possible Cross-over Between UPC to Intervention Clinics

Although cross-over between the two clinics was less than 0.5% at all sites in 2016 based on Phase 1 data, it is still possible that this could change when patients with OUDs want buprenorphine: they might travel to the PROUD intervention clinic to obtain it. Main analyses could be biased to the null if patients from UPC sought OUD treatment in the PROUD intervention clinic. We will therefore measure crossover at baseline and throughout the trial, will report crossover in our main manuscript, and will conduct sensitivity analyses to address crossover as described below (Section 11.7.1.3).

10.3 Design Considerations Regarding Measures

10.3.1 Primary Outcome Measure for the Primary Objective

Treatment of OUDs for at least a year has been associated with decreased acute care utilization.[36] We had therefore initially hoped to use OUD medication treatment for at least a year as our primary outcome. However, preliminary simulations by the DSC early in Phase 1 revealed that 6 sites would be inadequate for such a trial, and we did not have adequate budget to conduct an adequately sized trial for that dichotomous outcome. However, we developed a novel scaled measure of “patient-days of OUD treatment” that reflects both initiation and retention in treatment with medication treatment for OUDs. We will conduct preliminary analyses of this outcome, which is complicated to model by the fact that it is zero for most patients.

10.3.2 No Data on Methadone Maintenance OTP for Most Sites

It is possible that availability of PC treatment of OUDs could shift patients from OTPs into PC, in contrast to initiating new patients into OUD treatment. Also, the MA Model is expected to increase treatment in OTPs because some patients seeking buprenorphine will not be appropriate candidates for PC management or they will need to transfer to more structured programs. Ideally, we would therefore have OTP data from all sites. However, that was not possible: we could only obtain claims for methadone OTPs for all patients at one site (KPW) and 30% of patients at another (HF). Another site (MF) had one internal OTP program. However, even when OTP claims were available, estimating our outcome “days of OUD treatment” from the claims required a large

CONFIDENTIAL

number of assumptions. Therefore, a decision was made to use only evidence-based medication treatments provided in medical settings (buprenorphine and injectable extended-release naltrexone) in calculating days of OUD treatment. Nevertheless, to address the limitation of not having a measure of days treated with methadone from OTPs for all sites, we will conduct analyses using the available methadone maintenance data from 3 sites to evaluate whether implementation of the MA Model impacts utilization of methadone (in addition to buprenorphine and injectable naltrexone), and whether including OTP data changes our main results.

10.3.3 Timing of Outcomes Measurement

Initially, we planned to start measurement 6 months after randomization to allow sites up to 6 months to hire the NCM before outcome measurement began. However, several stakeholders pointed out that leaders of health systems would be interested in information on outcomes beginning at the time a decision was made to hire an OBAT NCM, which occurs at randomization in this trial. As a result, based on discussions with the protocol development team, a decision was made to measure outcomes starting at randomization. Process measures will include timing of NCMs and prescribers starting to care for patients with OUDs.

10.3.4 Use of EHR Orders for Buprenorphine and Injectable Naltrexone

Most sites in the PROUD trial do not provide us with data on dispensed medications but instead provide EHR prescription orders. Orders could have been written and never dispensed. However, we expect that is uncommon for buprenorphine. We will address this limitation by conducting sensitivity analyses with the outcome restricted to episodes of OUD treatment with at least a series of two orders (suggesting that the patient picked up and used the first prescription). We will also use data from the two sites that are health plans, which can therefore provide dispensed medications (KPWA and HF), to conduct sensitivity analyses to see if use of dispensed medications (compared to medication orders) changes main results at those two sites.

10.3.5 Incomplete Ascertainment of Acute Care and Other Patient Outcomes

Most participating sites are not health insurance plans (which typically have quite complete ascertainment of health utilization outcomes on their enrolled samples). Although it appears from preliminary Phase 1 analyses that we are capturing large and somewhat comparable amounts of acute care in all sites, it is possible that the two randomized clinics in a health system could differ in their ascertainment of acute care outcomes. For example, one of the clinics might be near an “internal” hospital or ED from which data are captured, while another of the clinics might be near another hospital or ED that is not part of their delivery system. This could create biased ascertainment of outcomes. To address this, we will qualitatively describe usual PC in both clinics at baseline including all nearby acute care facilities and distance to each clinic, and evaluate for differential acute care use pre-randomization to understand the extent to which this might impact secondary outcomes.

10.3.6 Incomplete Death Data

Most sites participating are not health insurance plans and do not have population based death data (i.e., from state vital status files). Relying on the EHR could bias the study towards greater ascertainment of OUD deaths in the PROUD intervention clinics due to NCM documentation. Mortality rates in patients with OUDs documented in EHRs are high (48.6 per 1000 person-years or a 10-fold increase over expected death rates (standardized mortality ratio of 10.3 (95% confidence interval [CI] 9.4–11.3)).[30] We address this below with sensitivity analyses and with an ancillary study to evaluate the intervention’s impact on death among patients with OUDs.

11.0 STATISTICAL ANALYSIS

11.1 Overview of Primary and Secondary Outcomes (Endpoints)

The planned primary outcome is a continuous measure defined as the total count of patient days of OUD treatment during the 2-year follow-up period per 10,000 patients seen in the clinic during that period. This outcome reflects the success of implementation of high quality OUD treatment in PC, including recognition of OUD (both previously diagnosed and newly diagnosed patients), as well as initiation of OUD treatment with medications and persistence of medication treatment, reflecting retention in OUD care. The study sample for calculating the number of patient days of OUD treatment is defined as all clinic patients who had a PC visit at any time in the 3 years prior to randomization or in the 2 years of follow-up after randomization. For each clinic, the primary outcome measure is operationalized as the total number of patient days covered by OUD treatment in the 2 years of follow-up divided by the number of patients in the clinic during that time and then multiplied by 10,000. Only buprenorphine and injectable naltrexone are considered OUD treatment when calculating the primary outcome measure.

Our hypothesis is that the PROUD intervention, compared to usual PC, will be associated with an increase in the number of patient days of medication treatment for OUDs during the 2 years after randomization. In addition to testing whether that is true when the MA Model is implemented in the diverse health systems in this trial, the analyses described below also estimate the magnitude of the increase.

As above, the secondary outcome is days of acute care utilization including visits to urgent care clinics, visits to EDs and days hospitalized after randomization among patients with or at increased risk for an OUD diagnosis in the 3 years before randomization. This is a patient outcome and reflects health benefits of the PROUD intervention to patients.

Our hypothesis is that the PROUD intervention, compared to usual PC, will be associated with a decrease in days of acute care utilization during 2 years of follow-up by PC patients diagnosed with OUDs in the 3 years prior to randomization.

11.2 Statistical Methods for Primary and Secondary Outcomes

11.2.1 Statistical Methods for the Primary Outcome

Since randomization and the intervention occur at the clinic-level, the unit of analysis will be clinic and not patient for the primary outcome. The mixed effects model evaluating the effect of the PROUD intervention is:

$$y_{ij} = \alpha + \beta * trt_{ij} + \gamma * z_{ij} + \theta_j + \epsilon_{ij}$$

where

- y_{ij} is the observed value of the primary outcome measure (see Section 8.1) for clinic i at site j
- trt_{ij} is the treatment indicator (PROUD intervention) for clinic i at site j
- z_{ij} is the observed value of the primary outcome measure for the two years prior to randomization (hereafter baseline) for clinic i at site j
- θ_j is the random effect for site j and is distributed $N(0, \sigma_\theta^2)$
- ϵ_{ij} is the error term for clinic i in site j and is distributed $N(0, \sigma_\epsilon^2)$

CONFIDENTIAL

The research question of interest is whether β significantly exceeded zero at the 0.05 level, thus a one-sided hypothesis test. Example SAS code for the analysis is as follows:

```
proc mixed data = primout;

  class arm;

  model scaletotdays = arm basevalue / solution;

  random intercept / subject = site;

  estimate "Treatment Effect" arm 1 -1;

run;
```

where “arm” is the treatment assignment, “scaletotdays” is the value of the primary outcome measure, “basevalue” is the value of the primary outcome measure for the two years prior to randomization and “site” indexes the different health care systems.

This model allows for clinics to be correlated within a health care system, in addition to allowing for an association of the scaled days of OUD treatment prior to randomization with the post-randomization outcome. The latter relationship is assumed to be linear. Secondary analyses of the primary outcome may relax this assumption by considering alternative relationships, such as inclusion of a quadratic term. Further, the need for a random effect of health care system can be evaluated by testing whether the variance of that term (i.e., σ_θ^2) is zero.

11.2.2 Statistical Methods: Secondary Outcome - Acute Care Utilization

Detailed analytic specifications for the secondary outcomes identified in Section 8.2 will be developed during start-up and detailed in a separate SAP. Below are brief descriptions of the proposed approaches.

We will evaluate, among individuals who have an OUD diagnosis, whether acute care utilization differs among patients from PROUD intervention clinics as compared to patients from UPC clinics (acute care utilization defined above in Measures Section 8.2). Our primary analysis will focus on patients who were identified as having an OUD prior to randomization. Because the PROUD intervention is expected to increase diagnosis of OUD, those patients diagnosed post-randomization in the PROUD intervention clinic are likely to be different than those diagnosed prior to implementation, and could therefore lead to biased estimates of the treatment effect if included in the analysis. Our current plan will be to exclude those diagnosed with OUD after randomization from the primary Objective 2 analysis. Secondary Objective 2 analyses (described below) will include those individuals who were not identified until post-randomization and will incorporate analytic methods for observational data.

CONFIDENTIAL

11.2.2.1 Primary Analysis of Objective 2

We hypothesize that, among patients who had a PC visit and were identified (pre-randomization) as having an OUD diagnosis (documented in their EHRs in the 3 years prior to randomization), individuals from a PROUD intervention clinic will have decreased acute care utilization after randomization as compared to individuals from a UPC clinic. We plan to fit a mixed-effect Poisson regression model (with log link) at the patient level to the number of days of acute care utilization. The model will account for clustering of patients within a clinic by including clinic-specific random intercepts. Specifically, the regression model will be of the following form:

$$log[E(y_{ijk})] = \alpha + \beta * trt_{ij} + \gamma * z_{ijk} + \theta_{ij}$$

where

- y_{ijk} is the observed number of days of acute care utilization of patient k in clinic i of site j
- trt_{ij} is the treatment indicator (PROUD intervention) for clinic i in site j
- z_{ijk} is a vector of clinic and/or patient-level covariates
- θ_{ij} is the random effect for clinic i in site j

We plan to adjust for a parsimonious list of pre-specified, baseline covariates that are known to be strongly associated with the outcome from the literature such as age, gender, race/ethnicity at baseline,[73] comorbidity and utilization prior to randomization, to be specified in the stand-alone SAP. We will evaluate our primary Objective 2 hypothesis by testing the null hypothesis $H_0: \beta = 0$ versus the two-sided alternative hypothesis that β is non-zero with a type 1 error rate of 0.05. As a sensitivity analysis, we will consider also including in the model any additional covariates found to differ between individuals with a prior OUD diagnosis in the PROUD intervention clinics as compared to the UPC clinics pre-randomization, as well as patient-level variables found to be associated with acute care utilization among patients with OUDs.

11.2.2.2 Secondary Analysis of Objective 2

As above, it is expected that some of the patients who initiate treatment in PROUD intervention clinics have been diagnosed with OUDs after randomization. It is also likely that patients may be newly attracted to the clinic (or to the health system entirely) specifically because of the PROUD intervention (based on the fact that at least 77% of patients treated in MA were new to the clinic after implementation).[47] The primary analysis of Objective 2 described above would miss any impact of the PROUD intervention on these patients, because they were not identified pre-randomization (because they were not previously diagnosed with OUDs or because they did not visit the clinic in the pre-randomization period). These secondary analyses are designed to capture these additional patients who may be affected by the PROUD intervention.

On the other hand, these secondary analyses must account for the fact that patients diagnosed with OUD post-randomization in the PROUD intervention clinics are likely to differ markedly from patients diagnosed with OUD post-randomization in the UPC clinics. Further, it is likely that these patients could differ in ways that may be associated with acute care utilization. To address this, analyses will adjust for potential confounding factors that are associated with both (1) acute care utilization, and (2) differences in characteristics of patients who get diagnosed with OUDs in PROUD intervention clinics (as compared to UPC clinics) after randomization.

We will first conduct preliminary analyses to compare the characteristics of patients diagnosed with OUDs in PROUD intervention clinics and UPC clinics. Because individuals who were first diagnosed with OUD prior to randomization are expected to be comparable across PROUD

intervention and UPC clinics (due to the randomization), these comparisons will focus on patients who entered our analytic sample because they were first diagnosed with OUDs in the 2 years post-randomization. Preliminary analyses will seek to understand differences in demographic characteristics, comorbidity, and utilization patterns between patients not included in primary analyses of Objective 2, but diagnosed with OUDs in PROUD intervention clinics and UPC clinics after randomization, in order to identify covariates for these secondary analyses of Objective 2.

We plan to fit a similar mixed-effect Poisson regression model as in the primary Objective 2 analysis but that includes additional covariates that allow the treatment effect comparing the PROUD intervention clinics to UPC clinics to differ among patients who were newly identified as having an OUD diagnosis in the post-implementation period. Specifically, the model will be of the following form:

$$log\left[E\left(y_{ijk}\right)\right] = \alpha_0 + \alpha_1 \mathrm{period}_{ijk} + (\beta_0 + \beta_1 * \mathrm{period}_{ijk}) * trt_{ij} + \gamma * z_{ijk} + \theta_{ij}$$

where *period* is an indicator for the period when the patient had their first documented OUD (in the post-randomization study phase versus pre-randomization), and the other terms are defined as in the primary analysis. We will adjust for the same covariates (in *z*) as in our primary Objective 2 analysis described above, as well as any covariate found to differ between individuals diagnosed with OUDs after randomization in the PROUD intervention clinics as compared to the UPC clinics, as identified in our preliminary analyses described above.

We will evaluate our secondary Objective 2 hypothesis by testing the composite null hypothesis $H_0: \beta_0 = \beta_1 = 0$ versus the alternative hypothesis that at least one of β_0 or β_1 is non-zero by conducting a likelihood ratio test. Additionally, we will estimate the treatment effect separately among people identified in the pre-implementation period (β_0) and people who weren't diagnosed until the post-implementation period ($\beta_0 + \beta_1$). The coefficient β_1 is the difference in the treatment effect comparing patients identified post-randomization to those identified in the pre-randomization period. This could either reflect a true difference in the treatment effect, or, more likely, it could reflect unmeasured confounders (not included in *z*) that differ between patients newly diagnosed post-randomization versus those with prior diagnoses. We do not have a specific hypothesis regarding β_1 because new patients may be attracted to the clinic to receive the PROUD intervention, as seen in Labelle,[1,47] and these patients may be sicker (or healthier) than patients identified pre-randomization, which could increase (or decrease) acute care utilization during the period after randomization. We are not powered to test for β_1 rather, this is an exploratory analysis that will generate hypotheses for testing in future studies.

11.3 Other Secondary Analyses

11.3.1 Representativeness of Randomized UPC Clinics for Usual PC in the 6 Sites

As above, the process of recruitment of sites for the PROUD trial suggested that we did not recruit typical PC clinics. We therefore plan to conduct analyses to compare the recruited (randomized) clinics from each site, to randomly selected PC clinics from the same health system, in the five health systems able to provide data from 4 other PC clinics. These patient level analyses compare patients seen in the randomized PC clinics (2 per site; total clinics =10) to those seen in the other PC clinics that were not included in the trial (4 per site, total clinics=20) in the 3 years prior to randomization. Characteristics to be compared will include the demographics and clinical characteristics of patients visiting the clinics (e.g., mental health diagnoses, substance use disorders diagnoses, medical diagnoses such as HCV[73] and HIV, and pain diagnoses, as well as our measure of increased risk for OUDs).

Additionally, we will evaluate whether, for each of the baseline measures of OUD care described above, there is a difference in the measure among randomized clinics compared to 4 non-randomized UPC clinics from the same site, adjusted for differences in patient demographics across the clinics. Specifically, we will fit a mixed effect Poisson regression model of the clinic-level outcome data (e.g., number of patients with an OUD diagnosis during the year prior to randomization). As in our primary outcome model, we will adjust for correlation of outcomes from the same site by including a site-specific random intercept. Specifically, we will consider models of the form

$$log\left(E(\,y_j)\right) = log(n) + \alpha + \beta * randomized + \gamma z + \theta_j,$$

where y_j denotes the clinic-level outcome (e.g., number of patients with an ICD for OUD during the year prior to randomization), $log(n)$ denotes an offset for the number of patients who visited the clinic in the year prior to randomization (for the recognition and initiation measures; for the retention measure it corresponds to the number of patients who initiated OUD treatment), *randomized* is an indicator for whether the clinic was included in the trial, *z* is a vector of demographic covariates being adjusted for (e.g., proportion male, comorbidity rates), and θ_j is a random intercept for site *j*. The coefficient β corresponds to the adjusted relative rate of the outcome (e.g., recognition of OUD) comparing *randomized* versus non- *randomized* clinics across all sites.

11.3.2 Secondary Modelling of Primary Outcome

Secondary analyses of the primary outcome measure with adjustment for additional covariates that are potential confounders will be provided in the standalone Statistical Analysis Plan (SAP). The exact approach to identifying which covariates will be included will be described in the SAP, including the process for selecting the appropriate analytic method.

11.3.3 PROUD Intervention vs. Other "Exemplar" Models of OUD Care

We will compare outcomes in ten exemplar clinics in 4 health systems to outcomes in the PROUD intervention clinics. We will compare implementation outcomes and patient outcomes (identical outcomes as for primary and secondary objectives) in the 2 years after randomization in those clinics, to the PROUD intervention clinics. Analyses for this secondary observational objective will follow the same general modeling approach as described above for the primary and secondary main outcomes (e.g., implementation and patient outcomes). To account for possible differences between the exemplar clinics that were not included in the trial as compared to the PROUD intervention clinics included in the trial, we will adjust for any covariate found to differ between these two sets of clinics (PROUD Intervention vs Exemplar) in the 3 years prior to randomization in the regression models.

11.3.4 Differences in Impact of PROUD on the Primary Outcome across Age, Sex and Race/Ethnicity

Given the NIH requirement to perform subgroup analyses of the primary outcome on the basis of sex, race and ethnicity, and the importance of understanding how the MA Model performs in individuals < 26 years, we plan to conduct analyses of subgroups based on: age (< 26 vs older); sex; race and ethnicity (categories depend on final race/ethnicity data available). An interaction term between demographic subgroup and treatment assignment can be used to evaluate whether the demographic factor moderates the treatment effect. Any such comparisons will likely be underpowered and must be interpreted with caution. Our hypothesis is that the PROUD intervention will result in differences in the main outcomes across sex, age, race and ethnicity,[73] so that we hypothesize that coefficients for interaction terms will not be equal to zero.

11.3.5 Evaluate Secondary Outcomes

Secondary outcome measures outlined in Section 8.3.2 are analyzed using methods similar to those used for the primary and secondary outcomes.

11.4 Rationale for Sample Size and Statistical Power

Given that Phase 1 of CTN-0074 found that only six health care systems (HCS) were eligible for Phase 2, the power calculations focus on 6 HCS, with each HCS contributing two clinics. One clinic from each health care system will be randomized to implement the PROUD intervention, while the other will continue with usual primary care (UPC). Simulations were conducted to calculate the power associated with various values of the treatment effect, which is parameterized as the mean of the primary outcome measure in the intervention clinics divided by the mean in the UPC clinics. Of ultimate interest in the calculations presented here is whether, with the sites selected in Phase 1, there will be sufficient power (>80%) to detect at least a 5-fold increase in the number of patient days of OUD treatment (per 10,000 participants) associated with implementation of the PROUD intervention as compared to UPC. To accomplish this, we considered various values of the treatment effect and calculated the corresponding statistical power via simulation.

11.4.1 Power Simulations – Primary Objective

11.4.1.1 Data-generation and Analysis Model

In our power simulation, there were 6 HCS and 2 clinics within each HCS (one clinic assigned to the intervention and one to UPC), making 12 clinics in all. The outcome variable for each clinic is *Treated Days per Patient Seen,* which with actual data, we would calculate by dividing the total number of treated days at the clinic during 2 years of follow-up by the total number of unique patients seen by the clinic during that same time period. In addition, we would use Phase 1 data to calculate an approximation of the magnitude of the association between the number of OUD-treated days per patient seen pre- and post-randomization. The analytic model previously specified would require four years of data for this estimation, however only three years of data are available from Phase 1. Thus, data was generated in a recursive fashion as follows:

Step 1: Estimate via regression the relationship between the number of OUD-treated days per patient seen in the last two years of Phase 1 data and the same measure for the first two years of the Phase 1 data. This corresponds to regression of the outcome for FYs 2015-2016 on FYs 2014-2015 (see Section 11.4.1.2). Since the Phase 1 data does not capture four years of data we cannot directly model the relationship between non-overlapping years. For this model, a random effect capturing the correlation of clinics from the same health care system was not included (see Section 11.4.1.2).

Step 2: Using this estimated regression model, predict the outcome measure for the next two- year period (FYs 2016-2017), and repeat to generate the outcome measure for the post-randomization period which roughly will correspond to FYs 2018-2019.

Step 3: For half of the clinics, when generating the data for the post-randomization period a covariate for treatment assignment is also included.

The details of this simulation approach will be included in the first version of the standalone SAP.

11.4.1.2 Parameters Used to Generate the Simulated Data

Table 12 provides the number of OUD-treated days during a two-year period per patient seen in the clinic. These values were used to estimate the parameters for Steps 1 and 2 in the algorithm summarized in Section 11.4.1.1.

Table 12. Number of Days Treated for OUDs (with buprenorphine or injectable naltrexone) per Patient Seen at 2 Phase 1 Clinics in Each of 6 Health Care Systems (HCS)

	Fiscal Years 2014-2015		Fiscal Years 2015-2016	
HCS	Clinic 1	Clinic 2	Clinic 1	Clinic 2
1	0.031	0.008	0.085	0.019
2	0.339	0.215	0.354	0.173
3	0.001	0.022	0.001	0.076
4	0.015	0.089	0.021	0.431
5	0.007	0.002	0.004	0.002
6	1.385	0.715	1.361	0.703

From Table 12 we fit a random effects model with a fixed intercept for the number of OUD-treated days per patient seen in FYs 2015-2016 as a function FYs 2014-2015 where the random effect captures the correlation between clinics arising from the same health care system. The estimated intercept was 0.05, the coefficient for FYs 2014-2015 was 0.94 and the variance of the random effect was not significantly different from zero. Thus, the predictive model used to generate the simulated data did not include a random effect for health care system (see Section 11.4.1.1).

11.4.1.3 Results of Simulations

It was also of interest to assess the potential increase in power associated with inclusion of the baseline value of the primary outcome measure in the regression model. Table 13 presents power results for the 0.05-level one-tailed test, based on 10,000 iterations per table cell for two models: one without adjustment for baseline, and one with baseline included as a covariate.

Table 13. Power Results for a 0.05-level One-Tailed Test, Based on 10,000 Iterations Per Cell

	Model	
**k-fold Increase in Primary Outcome (Treatment Effect)**	**No Adjustment for Baseline**	**Inclusion of Baseline as a Covariate**
1.00	5%	5%
1.03	8%	14%
1.06	13%	28%
1.09	18%	46%
1.12	24%	65%
1.18	39%	92%
1.24	54%	99%

Based on Table 13, there is at least 80% power to detect an 18% increase in the number of OUD-treated days per patient seen. Thus, with two clinics in each of six health care systems, the study is sufficiently powered to detect the targeted 5-fold increase in the primary outcome measure. As anticipated, there is a substantial gain in power when the baseline value is included as a covariate in the primary outcome model.

11.4.2 Power Simulations – Secondary Objective

We investigated the power of the primary Objective 2 analysis via simulation. Among individuals in the intervention clinic with an EHR documented OUD diagnosis pre-randomization, not all will visit the PROUD NCM and receive treatment with buprenorphine or injectable naltrexone (hereafter "treated for OUDs"). We explored how the power is affected by the proportion of patients with a prior OUD diagnosis who are treated for OUDs (p_trt). Based on the table below, and our expectation that the PROUD clinics will treat over 15% of patients with OUDs, we expect to have adequate power for our secondary objective.

We assumed the following sample sizes for the number of patients with a prior OUD diagnosis over a 3-year period from the Phase 1 data, reflecting the 3-year baseline period of PROUD during which patients with an OUD diagnosis will be identified:

site_id	clin_num	clin	nOUD
A	1	A1	9
A	2	A2	12
C	1	C1	63
C	2	C2	39
E	1	E1	58
E	2	E2	200
I	1	I1	100
I	2	I2	49
J	2	J2	10
K	1	K1	388
K	2	K2	290

We generated individual-level outcome data within each of the 12 clinics as follows. First, we randomly assigned one of the two clinics within a HCS to receive the PROUD intervention. Then, for each patient from a PROUD intervention clinic with a prior OUD diagnosis, we identified whether that patient was treated for OUDs by the nurse (with probability p_trt). Of the patients treated for OUDs by the nurse, we assumed that the probability that they are persistently treated is 50%. Among patients who are able to be treated for OUDs and are persistently treated, we generated outcome data from a Poisson distribution with mean number of acute care days over a two-year period (time-frame of PROUD outcome ascertainment) equal to that of individuals without a prior OUD diagnosis based on Phase 1 data (=1.7 days). Among the remaining patients (including those who are not treated for OUDs or who are not persistently treated), we generated outcome data from a Poisson distribution with the corresponding mean number of acute care days equal to that among individuals with a prior OUD diagnosis based on the Phase 1 data (=8.2

days). To each simulated dataset, we fit the Poisson mixed-effect model described in the analysis section.

Note that these initial power calculations were not based on simulating clinic-level random effects. In another set of simulations that did generate clinic-level random effects, the type I error rates were no longer accurate. This is because under the small number of clinics per treatment arm (intervention vs. usual care), generating outcome data in this way yielded imbalance in the mean number of acute care days in the intervention versus usual care arm. Imbalance in the rates of acute care across treatment arms is a concern; as described in our analysis plan above, we plan to adjust for baseline utilization along with other covariates that could account for differential utilization across clinics.

Results

The following plot and table show the power across different values for the proportion of patients within PROUD clinics who are treated for OUDs (p_trt). We see that power was >0.90 in scenarios where more than 20% of patients with recognized OUDs are treated for OUDs (p_trt > 0.20).

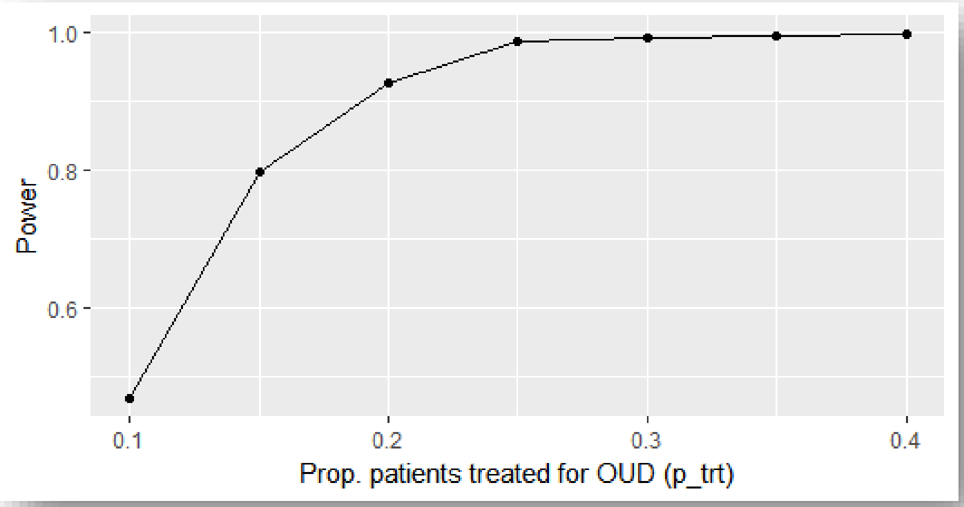

p_trt	Power	Type 1 error
0.10	0.468	0.056
0.15	0.798	0.046
0.20	0.926	0.046
0.25	0.988	0.030
0.30	0.992	0.064
0.35	0.996	0.020
0.40	0.998	0.036

11.5 Hypothesis Testing

Hypothesis testing for the primary outcome measure will be assessed using a one-sided significance level of 5%. While there are a large number of secondary outcomes, multiple comparisons will not be adjusted for since they are not of primary interest. However, care will be made to interpret any results with caution and report the number of comparisons made to provide context.

11.6 Interim Analyses

Due to the nature of this study, there are no formal interim analyses performed. There will be no re-evaluation of the sample size (i.e., number of health systems or clinics) and no interim assessment of efficacy or futility.

Data on the sample size and the number of patients in each clinic, along with the primary outcomes and other key secondary outcomes (e.g., overdoses [ODs], deaths) will be reported to the Data and Safety Monitoring Board (DSMB) as requested, to allow them to monitor data quality. Of note, after randomization, ODs and deaths are expected to reflect potentially biased ascertainment due to improved documentation in the PROUD clinics (Table 7).

11.7 Sensitivity Analyses

Due to the pragmatic nature of this trial and the use of secondary data collection, extensive sensitivity analyses will be undertaken. The following sensitivity analyses are planned to address as much as possible the limitations identified in Section 10.0. We provide an overview here, but these and others added during preliminary analyses will be specified in detail in the SAP.

11.7.1 Sensitivity Analyses for the Primary Outcome

11.7.1.1 No Data on Methadone Maintenance OTP Included in Main Outcome

We will conduct analyses using the available methadone maintenance data from 3 sites to evaluate whether inclusion of these data meaningfully change the main outcome. First, these data will also be used to assess the relationship between buprenorphine, injectable naltrexone and methadone treatment patterns, within and across the two treatment arms at the 3 sites. Subsequently, a modified primary outcome measure will be constructed as in Section 11.1 above that also includes days of methadone treatment in OTPs (as estimated above), and main analyses will be replicated with the modified (+methadone) outcome.

11.7.1.2 Imbalance in the Size of UPC to PROUD Intervention clinics or Other Factors

Every effort was made to balance the size of selected clinics (~10,000 visits from unique patients per year), however there were few options for clinics able to participate in most health systems, resulting in imbalance shown in Table #1 above. This limitation is difficult to handle. However, we will attempt to perform secondary analyses of the primary outcome measure that better address any imbalance. With respect to imbalance in clinic size, additional analyses may adjust for the number of patients seen post-randomization in addition to scaling by the same factor (per 10,000 patients) pre-randomization. Other approaches will be considered as needed.

11.7.1.3 Possible Crossover of Patients from UPC to PROUD Intervention Clinics

As described in Section 10.2.3, it is possible patients could cross-over from UPC to PROUD intervention clinics in order to obtain OUD treatment in primary care. In order to place findings of this pragmatic trial in context, secondary analyses will evaluate cross-over during the pre- and post-randomized periods. Cross-over will be defined as a patient assigned to a clinic in one arm of the trial (PROUD or UPC) pre-randomization being seen in a clinic in the other arm post-randomization. Patients will be assigned to a primary care clinic based on the algorithm stipulated in the standalone SAP related to where they have the most visits (per Section 6.1). We will evaluate cross-over in both directions, each year of the study, in the total sample as well as in primary care patients with an OUD diagnosis to understand trends over time in cross-over. However, the main analyses of interest will be the proportion of UPC patients with OUD diagnosed prior to randomization who are treated for OUDs in a PROUD clinic post-randomization.

In the event that there is substantial evidence of crossover of patients from a UPC clinic to a PROUD clinic, an additional analysis will be performed. The main analysis of the primary outcome measure "assigns" patients to the clinic as described above (and potentially refined in the standalone SAP). The sensitivity analysis to address the impact of crossover will "assign" patients to the clinic they were assigned to in the year after their first visit to a primary care clinic (pre- or post-randomization).

11.7.1.4 Use of Orders Instead of Medications Dispensed for OUD Treatment

When evaluating patient days of OUD treatment, only medication orders were available at most sites. It is possible that orders could have been written and never dispensed. However, we expect that this is uncommon. We will address this limitation by conducting sensitivity analyses with the outcome restricted to episodes of treatment with at least one refill, with the last refill omitted in case it as not picked up and taken by the patient.

11.7.2 Sensitivity Analyses for Main Secondary Outcome: Acute Care Utilization

11.7.2.1 Incomplete Ascertainment of Acute Care

It is possible that clinics within a health care system could differ in their ascertainment of acute care outcomes if, for example, one of the clinics was near a hospital or ED for which care was not captured by the health system. This is particularly true since most of the health care systems in this protocol are solely delivery systems (rather than integrated insurance plans and delivery systems). If differential acute care utilization is observed in preliminary data of the 3 years prior to randomization, we will consider approaches to addressing it in the final SAP.

CONFIDENTIAL

11.7.2.2 Possible Cross-over of Patients: UPC to PROUD Intervention Clinics

Sensitivity analyses abut cross-over for the secondary objective will be performed in an analogous manner to cross-over analyses described in Section 11.7.1.3 for the primary outcome measure.

11.8 Missing Data

Given that the primary and secondary outcomes for the main study rely on the EHR data, if there is no evidence in the EHR of a particular event, such as provision of buprenorphine or a visit to the ED, we will assume that the event did not occur. To assess whether this assumption is accurate we will conduct sensitivity analyses in the 2 systems that are health insurance plans, using enrolled samples and claims data. These same assumptions apply to the only covariate in the primary analyses of the primary objective (baseline value of the primary outcome in the 2 years prior to randomization).

For secondary analyses of the primary objective, covariates are defined as those for which there was imbalance between the PROUD and UPC clinics. There may be some missingness in some of these variables (e.g., missing race/ethnicity); the approach for handling missing covariate information for secondary analyses of the primary outcome and for the analysis of secondary outcomes will be detailed in the final version of the standalone SAP.

12.0 QUALITATIVE ANALYSIS

12.1 Formative Evaluation

We will use implementation-focused formative evaluation[141] to guide adaptation of the implementation to the diverse health systems in the trial if necessary. Barriers and facilitators will be summarized by the implementation monitoring team weekly for the LNT based on weekly TA Team conversations with the NCMs at PROUD intervention clinics. The LNT will review findings and, if significant barriers are encountered, will present results to the CTN Operations Team, to discuss. We will use an iterative approach to evaluating barriers and facilitators and—if needed—adapting the intervention (e.g., extra calls between the TA team and health system leadership to support hiring, or extra calls between the TA team and a physician who is not prescribing buprenorphine, if a site encounters such barriers). This approach has been used successfully in Dr. Bradley's stepped-wedge trial implementing alcohol-related care in 22 PC clinics using practice coaches in the Sustained Patient-centered Alcohol-related Care (SPARC) Project.[142]

12.2 Barriers and Facilitators

To address our objective to identify barriers and facilitators to implementation of the MA Model in PC in diverse health care systems (Section 4.6 above), we will use a rapid assessment process,[143] used in prior studies,[144] to develop a conceptual map of barriers and facilitators using the PRISM domains (Figure 2) as a foundation: intervention, recipients—including organizational leaders, managers, and staff, as well as patients, implementation infrastructure, and external environment.

CONFIDENTIAL

13.0 REGULATORY COMPLIANCE AND SAFETY

13.1 Statement of Compliance

This study will be conducted in compliance with the appropriate protocol and all applicable regulatory requirements. Prior to study initiation, the protocol and other supporting documents must be reviewed and approved by an Institutional Review Board (IRB). Any amendments to the protocol or other study materials must be approved by the IRB before they are implemented.

13.2 Regulatory Files

The regulatory files will contain all required regulatory documents, study-specific documents, and all important communications. Regulatory files will be checked for regulatory compliance by the Clinical Coordinating Center and Lead Node, as applicable, prior to study initiation, throughout the study, as well as at the study closure.

13.3 Care Implemented by Health Delivery Systems

The investigators, including site investigators, of this study have no contact with patients as part of conducting the study. Any change in the care processes or delivery of care to patients is implemented by the health care system and its clinicians. The clinics in the PROUD trial are implementing an improved care process and all care provided is consistent with routine standard practice in each health system that offers patients access to OUD treatment as part of routine PC. This sort of improvement in care processes is constantly occurring as part of quality improvement in health systems. As such, no patient consent is necessary for the sites to implement the MA Model into care in the 6 randomly-selected clinics. All data to evaluate outcomes are secondary data from the EHR and administrative and claims databases, and waiver of informed consent will be requested from the IRB. As above, site lead investigators and/or project managers will complete a questionnaire (e.g., how many clinicians practice in each clinic, what are the ways patients in a clinic can obtain buprenorphine), and conducti targeted stakeholder interviews to describe their health systems at baseline and monitor changes in the health system over time. The NCM also sends weekly aggregate reports to the TA team weekly that are cc'd to the lead node. These activities describing the health system and changes over time and monitoring implementation are summarized in Appendix D and considered "not human subjects research".

13.4 Risks

This study relies on secondary, electronic data from EHRs and administrative data from 8 health systems (6 in the trial and 2 providing data on exemplar clinics). The risk of using secondary data is <u>breach of confidentiality.</u> These data on OUD and OUD treatment are sensitive because, if disclosed, they could have negative consequences to patients or damage their financial standing, employability, insurability or reputation.

13.5 Mitigation of Risks of Use of Secondary Data

13.5.1 Steps Taken to Protect Secondary Data

The following steps are taken:

<u>IRB review.</u> All research activities will be reviewed and approved by a federally recognized central IRB for the protection of human subjects before any activities can take place.

<u>Staff training.</u> KPWHRI has strict procedures in place to assure the confidentiality of information on human subjects. All KPWHRI staff must annually sign a confidentiality agreement. All KPWHRI investigators and key personnel responsible for both the design and content of research are

required to receive training in the protection of human subjects. New employees receive training on data handling procedures, confidentiality and security.

Secure storage. Identifiable or protected health information (either hard copy or in computerized databases) is stored in secured areas with restricted access at the sites and at KPWHRI.

Data management to ensure privacy. The research procedures are designed to protect the safety, rights, and welfare of human subjects as well as the privacy and confidentiality of their protected health information as described below. Only approved project investigators, project managers and data analysts, including the designated programmers, will have access to the data. Data will be maintained on a KPWHRI secure-access drive in permission-restricted folders only accessible to the project investigators and data analysts.

Use of limited datasets. Programmers will create analytic datasets by removing all personal health information (PHI) except dates and zip codes from original datasets. The programmers at the sites will retain both the original datasets and a “crosswalk” to the study ID on secure servers at their sites. Only date and zip codes are provided to KPWHRI.

Limited access to data servers**.** Only approved project investigators, programmers and data analysts, will have access to the analytic datasets.

Secure transfer**.** Data are transferred to a secure transfer restricted-access folder, only accessible to the designated programmers from the sites. We will ensure the secure transmission of the original datasets by limiting the responsibility of pulling and/or downloading data to the designated programmers at each site. They will download data to Secure File Transfer (SFT).

The above measures protect these secondary data from disclosure. As a result, the waiver of consent and HIPAA Waiver to use of these secondary data will not adversely affect the rights and welfare of the subjects. Patient access to any benefit or service will not be affected by this study. As above, extensive safeguards are in place to protect the confidentiality of research subjects.

13.5.2 Benefits

The above potential risks must be weighed against the potential benefits of the proposed research. In 2015 over 52,000 US adults died of overdoses related to opioid use. But access to effective OUD medication treatment for OUDs remains limited. The PROUD trial is testing the impact of the most promising primary care model for providing high-quality OUD treatment in the PC setting. Data from MA suggest that it will result in a 5-10 fold increase in the number of patients offered OUD treatment if each PROUD intervention clinic treats 100 patients with OUDs.

13.6 Informed Consent

A waiver of informed consent will be requested from the IRB. Waivers of informed consent are used routinely for obtaining secondary health care data for minimal risk studies. Under 45 CFR 46.116(d), a waiver is permissible if the following four conditions are met:

1. The research involves no more than minimal risk to the subjects;
2. The waiver or altercation will not adversely affect the rights and welfare of the subjects;
3. The research could not practicably be carried out without the waiver or altercation;
4. Whenever appropriate the participants will be provided with additional pertinent information after participation.

The PROUD trial uses secondary observational data to evaluate outcomes that result from one of the 2 PC clinics at each site receiving the PROUD intervention (funding and technical

assistance for the MA Model) and the other continuing usual primary care. Use of these observational data include no more than minimal risk of harm to subjects, with the risk chiefly being loss of confidentiality of data. The risk of using these data is no greater than the risk of routine uses of health care data for quality improvement by the health systems.

Subjects' access to health care or health care benefits will not be affected by this study. We will follow measures described in the confidentiality Section below to ensure that all identifiers are removed from data and that limited datasets are accessible only to authorized parties.

The primary objective of this study—an implementation trial—could not be achieved if only a consenting sub-sample of patients were included. Moreover, to identify these subjects and conduct outreach to obtain signed authorization would greatly increase the risk of breach of confidentiality. Additionally, we estimate our subject enrollment numbers will exceed 120,000 across all participating health systems. The study could not therefore not be practically carried out if consent were required.

We will provide results of the study to the clinical leaders of each site in the trial. They will decide what to communicate to their patients. Study investigators will never contact patients, even after the trial, because we will not have access to their identities.

13.7 Confidentiality

All research activities will be reviewed and approved by an IRB to ensure subjects are adequately protected against risk. The research will be conducted under a waiver of written informed consent. In addition, the research will be carried out under a HIPAA waiver of authorization. Consistent with these applicable laws we will obtain the minimal data required for each analysis. Subject confidentiality will be strictly maintained through standard confidentiality procedures at each participating health system. All electronic files will be stored on secure health systems servers, accessible only to study staff. Subject confidentiality will be strictly maintained through standard procedures at each of the collaborating health systems. All data files will be coded with a study-created ID. Each participating health system's study programmer will assign study IDs to individuals; and will maintain a file for linking study IDs to medical record IDs. No files used for analysis will contain identifiers beyond dates and zip codes (e.g., limited datasets). Identifying information will be used only for the purpose of extracting relevant information from different data sources over time and merging into datasets at each site, replacing identifiers with a study ID before transfer of data to the lead node. Data will be provided to individuals on the study team as needed to conduct the PROUD analyses.

Confidentiality of specialty addiction treatment is covered under 42 CFR Part 2, which specifies conditions for sharing of identified data from covered entities. Recently, on January 18, 2017 (effective March 21, 2017), 42 CFR Part 2 regulations were changed and will likely be open to diverse interpretation again. However, several important changes have been made: 1) identifiers have been broadened to include HIPAA identifiers (which include date and zip code); 2) data can be shared by a lawful data owner (e.g., health plan that has insurance claims) as well as program directors. It may be desirable therefore that any health system providing data from 42CFR Part 2 covered entities provides a letter of assurance that their disclosure of dates and zip codes is permitted because the researcher receiving the data has complied with the necessary applicable requirements (e.g., HIPAA and IRB regulations).[145] 42 CFR Part 2 prohibits re-disclosure of data.

A Certificate of Confidentiality (CoC) is not appropriate for this study for the following reasons. Often CoCs are obtained for addictions research trials to prevent anyone from being able to subpoena data indicating a person has a substance use disorders. Often, just the fact of participating in the trial is evidence of a substance use disorder. In PROUD, secondary data are

CONFIDENTIAL

obtained for all PC patients so inclusion in the study is not an indication of an OUD. Moreover, no meaningful identifiers are shared. We do not expect a CoC would add any protection as all data are already documented in the patient's EHR, which can be subpoenaed. Finally, NIH will not provide a CoC to cover EHR data.

13.8 Documented OUD Treatment in the EHR and 42 CFR Part 2

Providing OUD treatment as part of PC in the MA Model has not fallen under 42 CFR previously. However, the regulation was recently revised (January 18, 2017, effective March 21, 2017) and interpretation of 42 CFR Part 2 regarding EHR documentation of OUD medication treatment data in the EHR in the MA Model could therefore evolve. However, we do not expect that to happen because the revised regulation did not change who or what was covered by the regulation and the MA Model has never fallen under the regulation in its spread across MA and no into WA. That is likely because general medical providers can fall under 42CFR if they meet one of two criteria. First, a provider can be covered by 42CFR if it "holds it out as providing and provides substance use disorder diagnosis, treatment, or referral for treatment" (https://www.gpo.gov/fdsys/pkg/FR-2017-01-18/pdf/2017-00719.pdf). Second, a provider can be covered by 42CFR if "its primary function is the provision of substance use disorder diagnosis, treatment or referral for treatment *and* is identified as such special medical personnel or other staff by the general medical facility." Nurses don't fall under these criteria because it is out of scope for nurses to diagnosis, treat, or refer. PC providers practicing under the MA Model do not fall under these definitions because they provide OUD treatment as part of routine PC and do not hold themselves out as providing substance use disorders treatment.[145] Nevertheless, if a site's legal counsel says that OUD medication treatment in PC falls under 42 CFR Part 2, the clinicians in that health system would have to develop a process to address that issue. Each site will be responsible for clarifying with their own legal counsel whether their OUD care in PC falls under 42CFR.

13.8.1 Health Information Accountability and Portability Act (HIPAA)

As stated above, we will request from the IRB a HIPAA waiver of authorization, which may be granted if the following conditions are met:

- Use or disclosure involves no more than minimal risk to the privacy of individuals because of the presence of at least the following elements:
  - An adequate plan to protect health information identifiers from improper use or disclosure;
  - An adequate plan to destroy identifiers at the earliest opportunity absent a health or research justification or legal requirement to retain them, and
  - Adequate written assurances that the PHI will not be used or disclosed to a third party except as required by law, for authorized oversight of the research study, or for other research uses and disclosures permitted by the Privacy Rule;
- Research could not practicably be conducted without the waiver or alteration; and,
- Research could not practicably be conducted without access to and use of PHI.

This study will be conducted in compliance with required HIPAA regulations. Identifiers will be destroyed 5 years following the end of the project. We estimate this will be December 2025.

We certify that the PHI used in this study (dates and zip codes) will not be disclosed to any person or entity except as required by law, for authorized oversight of the research study, or for other research for which the use or disclosure of protected health information would be permitted by an Institutional Review Board in accordance with relevant state and federal laws.

Additionally, we certify that we are obtaining the minimum information necessary in order to achieve the goals of the research.

13.9 Investigator Assurances

Each study site must file (or have previously filed) a Federal Wide Assurance (FWA) with the DHHS Office for Human Research Protection setting forth the commitment of the organization to establish appropriate policies and procedures for the protection of human research subjects, with documentation sent to NIDA or its designee. Research covered by these regulations cannot proceed in any manner prior to NIDA receipt of certification that the research has been reviewed and approved by an IRB covered under the assurance (45 CFR 46.103(b) and (f)). Prior to initiating the study, the principal investigator (PI) for each study site will sign a protocol signature page, providing assurances that the study will be performed according to the standards stipulated therein. The study Site PI must sign a protocol signature page for each corresponding, IRB-approved version of the protocol.

13.10 Financial Disclosure

All investigators will comply with the requirements of 42 CFR Part 50, Subpart F to ensure that the design, conduct, and reporting of the research will not be biased by any conflicting financial interest. Everyone with decision-making responsibilities regarding the protocol, including the site Principal Investigators, will have an up-to-date signed financial disclosure form on file with the sponsor.

13.11 Inclusion of Women and Minorities

The proposed study will include women and minorities as represented in the primary care clinics selected for the trial.

13.12 Records Retention and Requirements

Research records are to be maintained by the lead investigator in a secure location for a minimum of 3 years after the study is completed and closed. This includes all IRB records and other regulatory records per NIDA CTN policies that apply. These records are also to be maintained in compliance with IRB, State and Federal requirements, whichever is longest. The sponsor investigator must be notified in writing and acknowledgment must be received by the lead investigator prior to the destruction or relocation of research records.

13.13 Audits

The Sponsor has an obligation to ensure that this trial is conducted according to good research practice guidelines and may perform quality assurance audits for protocol compliance. The Lead Investigator, the National Institute on Drug Abuse Clinical Trials Network (NIDA CTN, the study sponsor); NIDA’s contracted agents, monitors or auditors; and other agencies such as the Department of Health and Human Services (DHHS), the Office for Human Research Protection (OHRP) and the IRB may inspect research records for verification of data, compliance with federal guidelines on human participant research, and to assess participant safety.

13.14 Reporting to Sponsor

The site principal investigator agrees to submit accurate, complete and timely reports to the Sponsor, as required. These include, but are not limited to, reports of any changes that significantly affect the conduct or outcome of the trial or increase risk to study participants. At the completion of the trial, the Lead Investigator will provide a final report to the Sponsor.

CONFIDENTIAL

13.15 Study Documentation

Study documentation for this trial includes sponsor-investigator correspondence, signed protocol and amendments, and IRB correspondence stored at each site. In addition, a cross walk between the medical record number and study ID are stored at each site (secured as above).

13.16 Data and Safety Monitoring Board

The DSMB will meet as often as the DSMB and CCTN deem appropriate, with meetings focused on data quality given that this study includes no patient contact. Reports to the Data and Safety Monitoring Board (DSMB) will be provided after randomization at a frequency specified by the DSMB and CCTN. While the data in Table 10 (above) cannot be provided more often than every 6 months, reports from the NCM to the TA and Implementation Monitoring Teams (Table 8) will be generated weekly and can be made available to the DSMB at their request. Process measures will be provided to the Trial Operations Team (Lead Node, CCTN, CCC, DSC) monthly. As above, due to the nature of this study, there are no formal interim analyses performed. There will be no re-evaluation of the detectable effect size, and no interim assessment of efficacy or futility. While limited safety data (i.e., overdoses) could be reported to the DSMB they are expected to reflect potentially biased ascertainment due to improved documentation in the PROUD intervention sites. Further, all care is provided by the site not the study, and it would not be appropriate to intervene at the patient or provider level. Information on mortality generally takes approximately two years to become available and thus may not be reported to the DSMB unless explicitly requested, and it is recognized that the list may not be complete.

CONFIDENTIAL

14.0 DATA MANAGEMENT

14.1 Overview

The project includes 3 types of data:(1) secondary quantitative data on clinical care and outcomes from sites' EHRs and administrative data, (2) qualitative data on the experience of implementing the MA Model at each site, and (3) summary NCM performance data from nurse care management weekly data reports to the TA team (e.g., total number screened, # of new patients enrolled, # of patients lost to follow-up, # re-engaged, etc.).

14.2 Design and Development of Quantitative Clinical and Administrative Data from Sites

This project will utilize a distributed data model that is standardized to the Health Care Systems Research Network (HCSRN) Virtual Data Warehouse (VDW) model.[123] Required data will be ascertained from electronic medical records (and claims data when available). Kaiser Permanente Washington (KPWA) will serve as the data center and be responsible for developing the study data dictionary and specifications for analytic datasets, development and validation of the study database, harmonization of data across sites, specifying procedures for compiling, querying, and transmitting data to KPWA, and ensuring data integrity.

14.3 Distributed Data Model

HCSRN Sites. The HCSRN VDW study sites have electronic medical and administrative data available on members dating back to the 1970's, which are updated at least quarterly. Currently, standard VDW data tables include but are not limited to the following:

- Demographics (birthdate, sex, self-reported race, self-reported Hispanic ethnicity)
- Census (income, educational attainment, race, ethnicity and characteristics of census block)
- Encounters (all inpatient, outpatient, radiology/imaging, telephone, and virtual encounters with date, rendering provider, facility, department and/or specialty) with linked tables providing additional data on:
  - Diagnoses (all recorded diagnoses with priority order, allowing ICD9 and ICD10 format)
  - Procedures (all recorded codes with rendering provider, allowing CPT and ICD format)
  - Providers (provider sex, age, specialty, race/ethnicity, years in practice)
- Pharmacy (dispensing date, prescribing provider, NDC/RxNorm code, quantity dispensed, days supply)
- Lab Results (test type, ordering department, order date-time, draw date-time, result date-time, result)
- Enrollment (start and end dates for coverage, insurance plan type and coverage characteristics)
- Mortality (date and cause of death, data source, confidence of match with state vital statistics records)
- Social history (drug, alcohol, sex, and tobacco history)

The electronic data sources are updated regularly and standardized into a single VDW format across HCSRN sites. The VDW follows a federated or distributed architecture paradigm, meaning that all identifiable data are held by member health systems. Each system translates data from local sources into local databases following a common data model (i.e., common file structures, variable definitions, specifications and formats).[146,147]

When sharing individual data is necessary, as is the case for the current study, each HCSRN site will execute a common data extraction program developed by KPWA programmers and share de-identified data containing the minimum information necessary to address the research question. The VDW is virtual in that it allows each HCSRN site to keep its own datasets locally, but run programs from any site against their local data and share those data as needed with KPWA. This retains local control, security, and confidentiality of access. Each site supports the VDW locally, thus we will be able to follow our subjects using electronic data through the end of follow-up and beyond at these sites. Maintaining, updating, and validating the consistency and quality of the VDW is an ongoing activity within HCSRN sites. Maintaining valid, quality data is a critical ongoing process as health care mandates and electronic medical record capabilities continually change.

Non-HCSRN Sites. Sites without a VDW will work with KPWA to build data tables from their EHRs that map to the VDW's standard data tables. These data tables will contain the minimum information necessary to address the research question and will be transferred to the KPW for programmers to run queries, similar to HCSRN sites. In order to ensure accurate extraction of data and translation of the data elements, each non-HCSRN site will create a mapping document that cross-references data values in their respective systems with the data values in the VDW.

14.4 Site Responsibilities

Sites will be responsible for building and/or maintaining the required data elements in VDW standard format. They will work with KPWA to build analytic datasets from VDW data tables, quality check data, modify and execute SAS programs on data tables and EHR (and claims data if available) as needed, and transferring data via a secure web portal set up by KPWA.

14.5 Data Center Responsibilities

KPWA data center management of administrative data will consist of various components that include: 1) develop and comply with a data management plan; 2) establish the data elements for extraction from the various sites; 3) develop and maintain an analytic dataset data dictionary for all study data; 4) develop programs for quality checks and standard reports; 5) program, document, and extract minimally necessary data to complete the study; 6) merge and harmonize extracted data across study sites; 7) quality checks, cleaning, and creation of linkable analytic datasets; 8) execute data use agreements; 9) transfer of limited analytic datasets as necessary and agreed upon via a secure web portal; and 10) share de-identified datasets with the DSC.

14.6 Data Transfer

KPWA will set up a Secure File Transfer (SFT) site for transferring data and data schema between the sites and KPWA. The SFT site secures Protected Health Information (PHI) via encryption. Site data managers will upload requested data tables to the SFT site, where KPWA programmers will download these files to secure KPWA network drives.

14.7 Data Training

Only trained programmers/analysts and biostatisticians will have access to the study site's computerized systems, perform data management, transfer data, create analytic datasets, and perform analyses of data.

14.8 Data Quality Assurance

Two types (levels) of standardized data queries will be run locally to validate key data characteristics. The first level addresses conformity to the data structure and primary keys. This level must be "passed" (i.e., run without errors) in order to load data into the tables. The second level of data checks focuses on formatting, completeness of data, outliers, logic checks, and

accuracy. Once data are uploaded to KPWA, an analyst will run a series of data queries to assess data quality and standardization. Results of these queries will be shared with the sites and used to guide remediation if necessary. Data summaries will be made available during the course of the study.

CONFIDENTIAL

15.0 PUBLICATIONS AND OTHER RIGHTS

Per NIH Policy, the results of the proposed study are to be made available to the research community and the public at large. The planning, preparation, and submission of publications will follow the policies of the Publications Committee of the CTN.

15.1 Dissemination Plan

Two main papers will result from this trial: the main paper on the primary objective and a secondary paper on the secondary objective. In addition, several papers are planned with Phase 1 data (main results on variation in the prevalence of OUDs and OUD treatment across health systems, results in youth and young adults; and the prevalence of OUDs and OUD treatment in patients with recognized mental health disorders). Other planned papers are outlined in Table 15.

Table 15. Planned manuscripts
• Baseline Measures of Generalizability of PROUD Trial Clinics: comparison to Other Clinics in the Same Health Systems on primary and secondary outcome
• Main Results: Primary Objective
• Main Results Secondary Objective
• Comparing PROUD Intervention and Exemplar Clinics
• Differences in PROUD Primary and Secondary Outcomes across Age, Sex and Race/Ethnicity Groups (separate papers)

To ensure that we are prepared for early dissemination, we have outlined key stakeholders, plans for engagement of these stakeholders in early dissemination planning, plans for post study activities.

List of key stakeholders for whom the findings will likely be relevant:

- Patients with OUDs and their families who can act as advocates for implementation of optimal models of care.
- Federal agencies addressing the opioid epidemic (SAMHSA and CSAT, HRSA, and the CDC) who make decisions on funding NCMs (we have already had a joint CSAT-CTN webinar on the design in Fall 2016).
- State, County and city, health leaders for departments of health, Medicaid, public employee benefits, and regional, county and city departments of health;
- Health insurance leaders who purchase OUD treatment;
- Leaders of health care systems that provide primary care;
- Researchers and quality improvement experts regarding addiction and primary care (e.g., NIH Collaboratory leaders; leaders of the Health Care System Research Network (HCSRN) and other Learning Health Systems experts; Society of General Internal Medicine), and
- Primary care clinic and mental health leaders and providers (e.g., American Board of Internal Medicine; American Board of Family Medicine, and American Psychiatric Association).

- National nursing leaders and groups, including groups that Ms. Labelle is already working with.

Recruitment and engagement of stakeholder group for dissemination. At the kickoff we will ask participants about key individuals from groups above who should be considered for this group. During Years 1 and 2 we will invite stakeholders and then convene 3 virtual meetings to develop a rapid dissemination plan, addressing core issues of interest to stakeholders (access and retention, prevention of adverse events such as acute care, and cost implications [from the ancillary study]). By working with this stakeholder group in advance we will increase target audience acceptance of the findings and increase the likelihood of possible implementation of study results as appropriate.

15.2 Data Sharing

The revised 42 CFR Part 2 regulation prevents re-disclosure of 42 CFR Part 2 covered data. Therefore, data cannot be shared by KP Washington unless it is de-identified. As a result, all data shared with the DSC will be de-identified unless it comes from each site separately to the DSC. Specifically, we will de-identify the main analytic dataset masking all dates. If the NIDA DSC is involved in conducting quality checks on the raw data (with dates or zip codes), to supplement those done in Seattle and reviewed in weekly meetings with the DSC, we will mask dates. Details regarding final quality checks are still under discussion and will be specified in the standalone SAP.

The main analytic dataset for the primary aim will be de-identified and shared on the NIDA Data Share website. This dataset will have no data elements representing clinics or health systems. The NIDA Data Share website should explicitly indicate that data elements for site or clinic (which would be masked) may be obtained from the Lead Investigator on a case-by-case basis, but access will be highly restricted and may require an additional DUA between the original sites and the requesting researcher.

CONFIDENTIAL

16.0 LIST OF APPENDICES

Documents provided separately:

APPENDIX A: MA Model OUD Care as Implemented for PROUD

APPENDIX B: Boston Medical Center OBAT Policy and Procedures Manual

APPENDIX C: Original PROUD Algorithms for Days of OUDs Treatment

APPENDIX D: Outline of Protocol Elements Anticipated to be “Not Human Subjects Research”

APPENDIX E: PROUD Clinical Staff Survey (Baseline)

APPENDIX F: PROUD Trial Economics Analysis

APPENDIX G: PROUD Clinical Staff Survey (Follow-up)

APPENDIX H: Nurse Care Manager Interview

APPENDIX I: Economic Analysis Nurse Care Manager Interviews

17.0 REFERENCES

1. Alford DP, LaBelle CT, Kretsch N, et al. Collaborative care of opioid-addicted patients in primary care using buprenorphine: five-year experience. *Arch Intern Med.* 2011;171(5):425-431.

2. Curran GM, Bauer M, Mittman B, Pyne JM, Stetler C. Effectiveness-implementation hybrid designs: combining elements of clinical effectiveness and implementation research to enhance public health impact. *Med Care.* 2012;50(3):217-226.

3. *Epidemic: responding to America's prescription drug abuse crisis.* Washington, D.C: Office of National Drug Control Policy Executive, Office of the President of the United States;2011.

4. Compton WM, Jones CM, Baldwin GT. Relationship between Nonmedical Prescription-Opioid Use and Heroin Use. *N Engl J Med.* 2016;374(2):154-163.

5. Hudson KL, Collins FS. The 21st Century Cures Act - A View from the NIH. *N Engl J Med.* 2017;376(2):111-113.

6. Stobbe M. A Grim Tally Soars: More than 50,000 Overdose Deaths in US. *STAT.* December 9, 2016;Health.

7. Jones CM, Campopiano M, Baldwin G, McCance-Katz E. National and State Treatment Need and Capacity for Opioid Agonist Medication-Assisted Treatment. *Am J Public Health.* 2015;105(8):e55-63.

8. Saha TD, Kerridge BT, Goldstein RB, et al. Nonmedical Prescription Opioid Use and DSM-5 Nonmedical Prescription Opioid Use Disorder in the United States. *J Clin Psychiatry.* 2016;77(6):772-780.

9. Wu LT, Zhu H, Swartz MS. Treatment utilization among persons with opioid use disorder in the United States. *Drug Alcohol Depend.* 2016;169:117-127.

10. McNeely J, Wu LT, Subramaniam G, et al. Performance of the Tobacco, Alcohol, Prescription Medication, and Other Substance Use (TAPS) Tool for Substance Use Screening in Primary Care Patients. *Ann Intern Med.* 2016;165(10):690-699.

11. Edlund MJ, Martin BC, Russo JE, DeVries A, Braden JB, Sullivan MD. The role of opioid prescription in incident opioid abuse and dependence among individuals with chronic noncancer pain: the role of opioid prescription. *Clin J Pain.* 2014;30(7):557-564.

12. Fleming MF, Balousek SL, Klessig CL, Mundt MP, Brown DD. Substance use disorders in a primary care sample receiving daily opioid therapy. *J Pain.* 2007;8(7):573-582.

13. Chou R, Turner JA, Devine EB, et al. The effectiveness and risks of long-term opioid therapy for chronic pain: a systematic review for a National Institutes of Health Pathways to Prevention Workshop. *Ann Intern Med.* 2015;162(4):276-286.

14. Degenhardt L, Bruno R, Lintzeris N, et al. Agreement between definitions of pharmaceutical opioid use disorders and dependence in people taking opioids for chronic non-cancer pain (POINT): a cohort study. *Lancet Psychiatry.* 2015;2(4):314-322.

15. Banta-Green CJ, Merrill JO, Doyle SR, Boudreau DM, Calsyn DA. Opioid use behaviors, mental health and pain--development of a typology of chronic pain patients. *Drug Alcohol Depend.* 2009;104(1-2):34-42.

16. Boscarino JA, Rukstalis M, Hoffman SN, et al. Risk factors for drug dependence among out-patients on opioid therapy in a large US health-care system. *Addiction.* 2010;105(10):1776-1782.

17. Dunn KM, Saunders KW, Rutter CM, et al. Opioid prescriptions for chronic pain and overdose: a cohort study. *Ann Intern Med.* 2010;152(2):85-92.

18. Bohnert AS, Valenstein M, Bair MJ, et al. Association between opioid prescribing patterns and opioid overdose-related deaths. *JAMA.* 2011;305(13):1315-1321.

19. Zedler B, Xie L, Wang L, et al. Risk factors for serious prescription opioid-related toxicity or overdose among Veterans Health Administration patients. *Pain Med.* 2014;15(11):1911-1929.

20. Chang YP, Compton P. Management of chronic pain with chronic opioid therapy in patients with substance use disorders. *Addict Sci Clin Pract.* 2013;8:21.

21. Edlund MJ, Forman-Hoffman VL, Winder CR, et al. Opioid abuse and depression in adolescents: Results from the National Survey on Drug Use and Health. *Drug Alcohol Depend.* 2015;152:131-138.

22. Grattan A, Sullivan MD, Saunders KW, Campbell CI, Von Korff MR. Depression and prescription opioid misuse among chronic opioid therapy recipients with no history of substance abuse. *Ann Fam Med.* 2012;10(4):304-311.

23. Seal KH, Shi Y, Cohen G, et al. Association of mental health disorders with prescription opioids and high-risk opioid use in US veterans of Iraq and Afghanistan. *JAMA.* 2012;307(9):940-947.

24. Dilokthornsakul P, Moore G, Campbell JD, et al. Risk Factors of Prescription Opioid Overdose Among Colorado Medicaid Beneficiaries. *J Pain.* 2016;17(4):436-443.

25. Gomes T, Mamdani MM, Dhalla IA, Paterson JM, Juurlink DN. Opioid dose and drug-related mortality in patients with nonmalignant pain. *Arch Intern Med.* 2011;171(7):686-691.

26. Dasgupta N, Funk MJ, Proescholdbell S, Hirsch A, Ribisl KM, Marshall S. Cohort Study of the Impact of High-Dose Opioid Analgesics on Overdose Mortality. *Pain Med.* 2016;17(1):85-98.

27. Jones CM, McAninch JK. Emergency Department Visits and Overdose Deaths From Combined Use of Opioids and Benzodiazepines. *Am J Prev Med.* 2015;49(4):493-501.

28. Fitzcharles MA, Ste-Marie PA, Gamsa A, Ware MA, Shir Y. Opioid use, misuse, and abuse in patients labeled as fibromyalgia. *Am J Med.* 2011;124(10):955-960.

29. Manchikanti L, Cash KA, Damron KS, Manchukonda R, Pampati V, McManus CD. Controlled substance abuse and illicit drug use in chronic pain patients: An evaluation of multiple variables. *Pain Physician.* 2006;9(3):215-225.

30. Hser YI, Mooney LJ, Saxon AJ, et al. High Mortality Among Patients With Opioid Use Disorder in a Large Healthcare System. *J Addict Med.* 2017.

31. Ling W, Hillhouse M, Ang A, Jenkins J, Fahey J. Comparison of behavioral treatment conditions in buprenorphine maintenance. *Addiction.* 2013;108(10):1788-1798.

32. Amato L, Minozzi S, Davoli M, Vecchi S. Psychosocial combined with agonist maintenance treatments versus agonist maintenance treatments alone for treatment of opioid dependence. *Cochrane Database Syst Rev.* 2011(10):CD004147.

33. Fiellin DA, Pantalon MV, Chawarski MC, et al. Counseling plus buprenorphine-naloxone maintenance therapy for opioid dependence. *N Engl J Med.* 2006;355(4):365-374.

34. Weiss RD, Potter JS, Fiellin DA, et al. Adjunctive counseling during brief and extended buprenorphine-naloxone treatment for prescription opioid dependence: a 2-phase randomized controlled trial. *Arch Gen Psychiatry.* 2011;68(12):1238-1246.

35. Murphy SM, Polsky D. Economic Evaluations of Opioid Use Disorder Interventions. *Pharmacoeconomics.* 2016;34(9):863-887.

36. Lo-Ciganic WH, Gellad WF, Gordon AJ, et al. Associations between trajectories of buprenorphine treatment and emergency department and inpatient utilization. *Addiction.* 2015.

37. Dunlap B, Cifu AS. Clinical Management of Opioid Use Disorder. *JAMA.* 2016;316(3):338-339.

38. Hser YI, Saxon AJ, Huang D, et al. Treatment retention among patients randomized to buprenorphine/naloxone compared to methadone in a multi-site trial. *Addiction.* 2014;109(1):79-87.

39. Fareed A, Vayalapalli S, Casarella J, Drexler K. Effect of buprenorphine dose on treatment outcome. *J Addict Dis.* 2012;31(1):8-18.

40. Minozzi S, Amato L, Vecchi S, Davoli M, Kirchmayer U, Verster A. Oral naltrexone maintenance treatment for opioid dependence. *Cochrane Database Syst Rev.* 2006(1):CD001333.

41. Johansson BA, Berglund M, Lindgren A. Efficacy of maintenance treatment with naltrexone for opioid dependence: a meta-analytical review. *Addiction.* 2006;101(4):491-503.

42. Lee JD, Friedmann PD, Kinlock TW, et al. Extended-Release Naltrexone to Prevent Opioid Relapse in Criminal Justice Offenders. *N Engl J Med.* 2016;374(13):1232-1242.

43. Cornish JW, Metzger D, Woody GE, et al. Naltrexone pharmacotherapy for opioid dependent federal probationers. *J Subst Abuse Treat.* 1997;14(6):529-534.

44. Krupitsky EM, Zvartau EE, Masalov DV, et al. Naltrexone for heroin dependence treatment in St. Petersburg, Russia. *J Subst Abuse Treat.* 2004;26(4):285-294.

45. Krupitsky E, Nunes EV, Ling W, Illeperuma A, Gastfriend DR, Silverman BL. Injectable extended-release naltrexone for opioid dependence: a double-blind, placebo-controlled, multicentre randomised trial. *Lancet.* 2011;377(9776):1506-1513.

46. Comer SD, Sullivan MA, Yu E, et al. Injectable, sustained-release naltrexone for the treatment of opioid dependence: a randomized, placebo-controlled trial. *Arch Gen Psychiatry.* 2006;63(2):210-218.

47. LaBelle CT, Han SC, Bergeron A, Samet JH. Office-Based Opioid Treatment with Buprenorphine (OBOT-B): Statewide Implementation of the Massachusetts Collaborative Care Model in Community Health Centers. *J Subst Abuse Treat.* 2016;60:6-13.

48. Rieckmann T, Daley M, Fuller BE, Thomas CP, McCarty D. Client and counselor attitudes toward the use of medications for treatment of opioid dependence. *J Subst Abuse Treat.* 2007;32(2):207-215.

49. Becker WC, Fiellin DA, Merrill JO, et al. Opioid use disorder in the United States: insurance status and treatment access. *Drug Alcohol Depend.* 2008;94(1-3):207-213.

CONFIDENTIAL

50. Gordon AJ, Lo-Ciganic WH, Cochran G, et al. Patterns and Quality of Buprenorphine Opioid Agonist Treatment in a Large Medicaid Program. *J Addict Med.* 2015;9(6):470-477.

51. O'Brien CP. A 50-year-old woman addicted to heroin: review of treatment of heroin addiction. *JAMA.* 2008;300(3):314-321.

52. American Methadone Treatment Association. *Methadone Maintenance Program and Patient Census in the US.* New York, NY: American Methadone Treatment Association;1998.

53. National Survey on Drug Use and Health (NSDUH). Results from the 2012 National Survey on Drug Use and Health: Summary of National Findings and Detailed Tables. 2014; http://www.samhsa.gov/data/NSDUH/2012SummNatFindDetTables/Index.aspx Accessed April 11, 2014.

54. Green CA, McCarty D, Mertens J, et al. A qualitative study of the adoption of buprenorphine for opioid addiction treatment. *J Subst Abuse Treat.* 2014;46(3):390-401.

55. Korthuis PT, McCarty D, Weimer M, et al. Primary Care-Based Models for the Treatment of Opioid Use Disorder: A Scoping Review. *Ann Intern Med.* 2017;166(4):268-278.

56. McNeely J, Drucker E, Hartel D, Tuchman E. Office-based methadone prescribing: acceptance by inner-city practitioners in New York. *J Urban Health.* 2000;77(1):96-102.

57. McLellan AT, Starrels JL, Tai B, et al. Can Substance Use Disorders be Managed Using the Chronic Care Model? Review and Recommendations from a NIDA Consensus Group. *Public Health Rev.* 2014;35(2).

58. Lee JD, McDonald R, Grossman E, et al. Opioid treatment at release from jail using extended-release naltrexone: a pilot proof-of-concept randomized effectiveness trial. *Addiction.* 2015;110(6):1008-1014.

59. Han B, Polydorou S, Ferris R, Blaum CS, Ross S, McNeely J. Demographic Trends of Adults in New York City Opioid Treatment Programs-An Aging Population. *Subst Use Misuse.* 2015;50(13):1660-1667.

60. Gordon AJ, Bertholet N, McNeely J, Starrels JL, Tetrault JM, Walley AY. 2013 Update in addiction medicine for the generalist. *Addict Sci Clin Pract.* 2013;8:18.

61. Roose RJ, Kunins HV, Sohler NL, Elam RT, Cunningham CO. Nurse practitioner and physician assistant interest in prescribing buprenorphine. *J Subst Abuse Treat.* 2008;34(4):456-459.

62. Brooklyn JR, Sigmon SC. Vermont Hub-and-Spoke Model of Care For Opioid Use Disorder: Development, Implementation, and Impact. *J Addict Med.* 2017.

63. Fiellin DA, Moore BA, Sullivan LE, et al. Long-term treatment with buprenorphine/naloxone in primary care: results at 2-5 years. *Am J Addict.* 2008;17(2):116-120.

64. Fiellin DA, Pantalon MV, Pakes JP, O'Connor PG, Chawarski M, Schottenfeld RS. Treatment of heroin dependence with buprenorphine in primary care. *Am J Drug Alcohol Abuse.* 2002;28(2):231-241.

65. Clark RE, Baxter JD. Responses of state Medicaid programs to buprenorphine diversion: doing more harm than good? *JAMA Intern Med.* 2013;173(17):1571-1572.

66. Jonas DE, Amick HR, Feltner C, et al. Pharmacotherapy for adults with alcohol use disorders in outpatient settings: a systematic review and meta-analysis. *JAMA.* 2014;311(18):1889-1900.

CONFIDENTIAL

67. Anton RF, O'Malley SS, Ciraulo DA, et al. Combined pharmacotherapies and behavioral interventions for alcohol dependence: the COMBINE study: a randomized controlled trial. *JAMA.* 2006;295.

68. Oslin DW, Lynch KG, Maisto SA, et al. A randomized clinical trial of alcohol care management delivered in Department of Veterans Affairs primary care clinics versus specialty addiction treatment. *J Gen Intern Med.* 2014;29(1):162-168.

69. Lo-Ciganic W, Zgibor JC, Ruppert K, Arena VC, Stone RA. Identifying type 1 and type 2 diabetic cases using administrative data: a tree-structured model. *J Diabetes Sci Technol.* 2011;5(3):486-493.

70. Lo-Ciganic WH, Gellad WF, Gordon AJ, et al. Association between trajectories of buprenorphine treatment and emergency department and in-patient utilization. *Addiction.* 2016;111(5):892-902.

71. Agency for Health Research and Quality. Draft Technical Brief: Medication-Assisted Treatment Models of Care for Opioid Use Disorder in Primary Care Settings. In: Agency for Healthcare Research and Quality US Department of Health and Human Services 5600 Fishers Lane Rockville M, www.ahrq.gov, eds2016.

72. Clark RE, Samnaliev M, Baxter JD, Leung GY. The evidence doesn't justify steps by state Medicaid programs to restrict opioid addiction treatment with buprenorphine. *Health Aff (Millwood).* 2011;30(8):1425-1433.

73. Weinstein ZM, Kim HW, Cheng DM, et al. Long-term retention in Office Based Opioid Treatment with buprenorphine. *J Subst Abuse Treat.* 2017;74:65-70.

74. Feldstein AC, Glasgow RE. A practical, robust implementation and sustainability model (PRISM) for integrating research findings into practice. *Jt Comm J Qual Patient Saf.* 2008;34(4):228-243.

75. Rogers EM. *Diffusion of Innovations.* New York, NY: Free Press; 1995.

76. Greenhalgh T, Robert G, Macfarlane F, Bate P, Kyriakidou O. Diffusion of innovations in service organizations: systematic review and recommendations. *Milbank Q.* 2004;82(4):581-629.

77. Damschroder LJ, Aron DC, Keith RE, Kirsh SR, Alexander JA, Lowery JC. Fostering implementation of health services research findings into practice: a consolidated framework for advancing implementation science. *Implement Sci.* 2009;4:50.

78. Press MJ, Howe R, Schoenbaum M, et al. Medicare Payment for Behavioral Health Integration. *N Engl J Med.* 2017;376(5):405-407.

79. Gaglio B, Shoup JA, Glasgow RE. The RE-AIM framework: a systematic review of use over time. *Am J Public Health.* 2013;103(6):e38-46.

80. Glasgow RE, Dickinson P, Fisher L, et al. Use of RE-AIM to develop a multi-media facilitation tool for the patient-centered medical home. *Implement Sci.* 2011;6:118.

81. Glasgow RE, Vogt TM, Boles SM. Evaluating the public health impact of health promotion interventions: the RE-AIM framework. *Am J Public Health.* 1999;89(9):1322-1327.

82. Bawor M, Dennis BB, Varenbut M, et al. Sex differences in substance use, health, and social functioning among opioid users receiving methadone treatment: a multicenter cohort study. *Biol Sex Differ.* 2015;6:21.

CONFIDENTIAL

83. Cicero TJ, Ellis MS, Surratt HL, Kurtz SP. The changing face of heroin use in the United States: a retrospective analysis of the past 50 years. *JAMA Psychiatry.* 2014;71(7):821-826.

84. Jones JD, Roux P, Stancliff S, Matthews W, Comer SD. Brief overdose education can significantly increase accurate recognition of opioid overdose among heroin users. *Int J Drug Policy.* 2014;25(1):166-170.

85. Fischer G, Eder H. [Interdisciplinary therapeutic approaches with substance abusers taking into consideration gender differences]. *Wien Med Wochenschr.* 1999;149(11):331-336.

86. Centers for Disease Control and Prevention. Today's Heroin Epidemic. *Vital Signs*2015.

87. Zubieta JK, Dannals RF, Frost JJ. Gender and age influences on human brain mu-opioid receptor binding measured by PET. *Am J Psychiatry.* 1999;156(6):842-848.

88. Gear RW, Miaskowski C, Gordon NC, Paul SM, Heller PH, Levine JD. Kappa-opioids produce significantly greater analgesia in women than in men. *Nat Med.* 1996;2(11):1248-1250.

89. Kornstein SG, Schatzberg AF, Thase ME, et al. Gender differences in treatment response to sertraline versus imipramine in chronic depression. *Am J Psychiatry.* 2000;157(9):1445-1452.

90. Jones HE, Fitzgerald H, Johnson RE. Males and females differ in response to opioid agonist medications. *Am J Addict.* 2005;14(3):223-233.

91. Schottenfeld RS, Pakes JR, Kosten TR. Prognostic factors in Buprenorphine- versus methadone-maintained patients. *J Nerv Ment Dis.* 1998;186(1):35-43.

92. Johnson RE, Eissenberg T, Stitzer ML, Strain EC, Liebson IA, Bigelow GE. A placebo controlled clinical trial of buprenorphine as a treatment for opioid dependence. *Drug Alcohol Depend.* 1995;40(1):17-25.

93. Ling W, Hillhouse M, Domier C, et al. Buprenorphine tapering schedule and illicit opioid use. *Addiction.* 2009;104(2):256-265.

94. McPherson S, Barbosa-Leiker C, Burns GL, Howell D, Roll J. Missing data in substance abuse treatment research: current methods and modern approaches. *Exp Clin Psychopharmacol.* 2012;20(3):243-250.

95. McPherson S, Barbosa-Leiker C, Mamey MR, McDonell M, Enders CK, Roll J. A 'missing not at random' (MNAR) and 'missing at random' (MAR) growth model comparison with a buprenorphine/naloxone clinical trial. *Addiction.* 2015;110(1):51-58.

96. Cicero TJ, Lynskey M, Todorov A, Inciardi JA, Surratt HL. Co-morbid pain and psychopathology in males and females admitted to treatment for opioid analgesic abuse. *Pain.* 2008;139(1):127-135.

97. Kelly SM, Schwartz RP, O'Grady KE, et al. Gender Differences Among In- and Out-of-Treatment Opioid-Addicted Individuals. *Am J Drug Alcohol Abuse.* 2009;35(1):38-42.

98. Green TC, Grimes Serrano JM, Licari A, Budman SH, Butler SF. Women who abuse prescription opioids: findings from the Addiction Severity Index-Multimedia Version Connect prescription opioid database. *Drug Alcohol Depend.* 2009;103(1-2):65-73.

99. Rustoen T, Wahl AK, Hanestad BR, Lerdal A, Paul S, Miaskowski C. Gender differences in chronic pain--findings from a population-based study of Norwegian adults. *Pain Manag Nurs.* 2004;5(3):105-117.

100. Hser YI, Anglin MD, Liu Y. A survival analysis of gender and ethnic differences in responsiveness to methadone maintenance treatment. *Int J Addict.* 1990;25(11A):1295-1315.

101. Magura S, Nwakeze PC, Demsky SY. Pre- and in-treatment predictors of retention in methadone treatment using survival analysis. *Addiction.* 1998;93(1):51-60.

102. Peles E, Adelson M. Gender differences and pregnant women in a methadone maintenance treatment (MMT) clinic. *J Addict Dis.* 2006;25(2):39-45.

103. Strain EC, Bigelow GE, Liebson IA, Stitzer ML. Moderate- vs high-dose methadone in the treatment of opioid dependence: a randomized trial. *JAMA.* 1999;281(11):1000-1005.

104. Jimenez-Trevino L, Saiz PA, Garcia-Portilla MP, et al. A 25-year follow-up of patients admitted to methadone treatment for the first time: mortality and gender differences. *Addict Behav.* 2011;36(12):1184-1190.

105. Martins SS, Santaella-Tenorio J, Marshall BD, Maldonado A, Cerda M. Racial/ethnic differences in trends in heroin use and heroin-related risk behaviors among nonmedical prescription opioid users. *Drug Alcohol Depend.* 2015;151:278-283.

106. Manhapra A, Quinones L, Rosenheck R. Characteristics of veterans receiving buprenorphine vs. methadone for opioid use disorder nationally in the Veterans Health Administration. *Drug Alcohol Depend.* 2016;160:82-89.

107. Hansen H, Siegel C, Wanderling J, DiRocco D. Buprenorphine and methadone treatment for opioid dependence by income, ethnicity and race of neighborhoods in New York City. *Drug Alcohol Depend.* 2016;164:14-21.

108. McCabe SE, West BT, Veliz P, McCabe VV, Stoddard SA, Boyd CJ. Trends in Medical and Nonmedical Use of Prescription Opioids Among US Adolescents: 1976-2015. *Pediatrics.* 2017;139(4).

109. Substance Abuse and Mental Health Services Administration Center for Behavioral Health Statistics and Quality. *Behavioral health trends in the United States: Results from the 2014 National Survey on Drug Use and Health.* Rockville, MD: Substance Abuse and Mental Health Services Administration;2015.

110. Subramaniam GA, Stitzer MA. Clinical characteristics of treatment-seeking prescription opioid vs. heroin-using adolescents with opioid use disorder. *Drug Alcohol Depend.* 2009;101(1-2):13-19.

111. Subramaniam GA, Fishman MJ, Woody G. Treatment of opioid-dependent adolescents and young adults with buprenorphine. *Curr Psychiatry Rep.* 2009;11(5):360-363.

112. Marsch LA, Moore SK, Borodovsky JT, et al. A randomized controlled trial of buprenorphine taper duration among opioid-dependent adolescents and young adults. *Addiction.* 2016;111(8):1406-1415.

113. Marsch LA, Bickel WK, Badger GJ, et al. Comparison of pharmacological treatments for opioid-dependent adolescents: a randomized controlled trial. *Arch Gen Psychiatry.* 2005;62(10):1157-1164.

114. Woody GE, Poole SA, Subramaniam G, et al. Extended vs short-term buprenorphine-naloxone for treatment of opioid-addicted youth: a randomized trial. *JAMA.* 2008;300(17):2003-2011.

CONFIDENTIAL

115. Committee On Substance USE, Prevention. Medication-Assisted Treatment of Adolescents With Opioid Use Disorders. *Pediatrics.* 2016;138(3).

116. Matson SC, Hobson G, Abdel-Rasoul M, Bonny AE. A retrospective study of retention of opioid-dependent adolescents and young adults in an outpatient buprenorphine/naloxone clinic. *J Addict Med.* 2014;8(3):176-182.

117. Schuman-Olivier Z, Weiss RD, Hoeppner BB, Borodovsky J, Albanese MJ. Emerging adult age status predicts poor buprenorphine treatment retention. *J Subst Abuse Treat.* 2014;47(3):202-212.

118. Kidorf M, Disney ER, King VL, Neufeld K, Beilenson PL, Brooner RK. Prevalence of psychiatric and substance use disorders in opioid abusers in a community syringe exchange program. *Drug Alcohol Depend.* 2004;74(2):115-122.

119. McLellan AT, Luborsky L, Woody GE, O'Brien CP, Druley KA. Predicting response to alcohol and drug abuse treatments. Role of psychiatric severity. *Arch Gen Psych.* 1983;40(6):620-625.

120. Rao SR, Broome KM, Simpson DD. Depression and hostility as predictors of long-term outcomes among opiate users. *Addiction.* 2004;99(5):579-589.

121. Saxon AJ, Wells EA, Fleming C, Jackson TR, Calsyn DA. Pre-treatment characteristics, program philosophy and level of ancillary services as predictors of methadone maintenance treatment outcome. *Addiction.* 1996;91(8):1197-1209.

122. Dreifuss JA, Griffin ML, Frost K, et al. Patient characteristics associated with buprenorphine/naloxone treatment outcome for prescription opioid dependence: Results from a multisite study. *Drug Alcohol Depend.* 2013;131(1-2):112-118.

123. Ross TR, Ng D, Brown JS, et al. The HMO Research Network Virtual Data Warehouse: A Public Data Model to Support Collaboration. *EGEMS (Wash DC).* 2014;2(1):1049.

124. Calcaterra S, Glanz J, Binswanger IA. National trends in pharmaceutical opioid related overdose deaths compared to other substance related overdose deaths: 1999-2009. *Drug Alcohol Depend.* 2013;131(3):263-270.

125. Zeng Z, Noonan M, Carson EA, Binswanger I, Blatchford P, Smiley-McDonald H. *Assessing Inmate Cause of Death: Deaths in Custody Reporting Program and National Death Index.* U.S. Department of Justice; April 2016.

126. Binswanger IA, Blatchford PJ, Forsyth SJ, Stern MF, Kinner SA. Epidemiology of Infectious Disease-Related Death After Release from Prison, Washington State, United States, and Queensland, Australia: A Cohort Study. *Public Health Rep.* 2016;131(4):574-582.

127. Binswanger IA, Carson EA, Krueger PM, Mueller SR, Steiner JF, Sabol WJ. Prison tobacco control policies and deaths from smoking in United States prisons: population based retrospective analysis. *BMJ.* 2014;349:g4542.

128. Binswanger IA, Blatchford PJ, Lindsay RG, Stern MF. Risk factors for all-cause, overdose and early deaths after release from prison in Washington state. *Drug Alcohol Depend.* 2011;117(1):1-6.

129. Calcaterra S, Binswanger IA. National trends in psychostimulant-related deaths: 1999-2009. *Subst Abus.* 2013;34(2):129-136.

130. Binswanger IA, Morenoff JD, Chilcote CA, Harding DJ. Ascertainment of Vital Status Among People With Criminal Justice Involvement Using Department of Corrections Records, the US National Death Index, and Social Security Master Death Files. *Am J Epidemiol.* 2017;185(10):982-985.

131. Binswanger IA, White MC, Perez-Stable EJ, Goldenson J, Tulsky JP. Cancer screening among jail inmates: frequency, knowledge, and willingness. *Am J Public Health.* 2005;95(10):1781-1787.

132. Binswanger IA, Wortzel HS. Treatment for individuals with HIV/AIDS following release from prison. *JAMA.* 2009;302(2):147; author reply 148.

133. Freedman LS, Midthune D, Carroll RJ, et al. Application of a New Statistical Model for Measurement Error to the Evaluation of Dietary Self-report Instruments. *Epidemiology.* 2015;26(6):925-933.

134. (ASAM) ASoAM. Providers Clinical Support System for Medication Assisted Treatment (PCSS-MAT) 2017; https://www.asam.org/education/resources/pcss-mat. Accessed July 2, 2017, 2017.

135. Gagne JJ, Glynn RJ, Avorn J, Levin R, Schneeweiss S. A combined comorbidity score predicted mortality in elderly patients better than existing scores. *J Clin Epidemiol.* 2011;64(7):749-759.

136. Charlson ME, Pompei P, Ales KL, MacKenzie CR. A new method of classifying prognostic comorbidity in longitudinal studies: development and validation. *J Chronic Dis.* 1987;40(5):373-383.

137. Elixhauser A, Steiner C, Harris DR, Coffey RM. Comorbidity measures for use with administrative data. *Med Care.* 1998;36(1):8-27.

138. Quan H, Sundararajan V, Halfon P, et al. Coding algorithms for defining comorbidities in ICD-9-CM and ICD-10 administrative data. *Med Care.* 2005;43(11):1130-1139.

139. Deyo RA, Cherkin DC, Ciol MA. Adapting a clinical comorbidity index for use with ICD-9-CM administrative databases. *J Clin Epidemiol.* 1992;45(6):613-619.

140. Freedland KE, Mohr DC, Davidson KW, Schwartz JE. Usual and unusual care: existing practice control groups in randomized controlled trials of behavioral interventions. *Psychosom Med.* 2011;73(4):323-335.

141. Stetler CB, Legro MW, Wallace CM, et al. The role of formative evaluation in implementation research and the QUERI experience. *J Gen Intern Med.* 2006;21 Suppl 2:S1-8.

142. Bradley K, Lozano P, (Multiple PIs). Sustained Implementation of Patient-Centered Care for Alcohol Misuse: The Alcohol Related Care (ARC) Project. Group Health Research Institute: Agency for Healthcare Research and Quality (AHRQ); 2014-2018.

143. Beebe J. *Rapid Assessment Process: An Introduction.* Altmira Press; 2001.

144. Williams E, Achtmeyer C, Young J, et al. Barriers and Facilitators to Alcohol Use Disorders Pharmacotherapy in Primary Care: A Qualitative Study in 5 VA Clinics. *J Gen Int Med.* 2017;Under revision.

145. Department of Health and Human Services. 42 CFR Part 2. Confidentiality of Substance Use Disorder Patient Records. In: Federal Register, ed. Vol 822017:6052-6127.

146. Curtis LH, Weiner MG, Boudreau DM, et al. Design considerations, architecture, and use of the Mini-Sentinel distributed data system. *Pharmacoepidemiol Drug Saf.* 2012;21 Suppl 1:23-31.

147. Maro JC, Platt R, Holmes JH, et al. Design of a national distributed health data network. *Ann Intern Med.* 2009;151(5):341-344.

CTN-0074

Primary Care Opioid Use Disorders Treatment (PROUD) Phase 2

Statistical Analysis Plan (SAP)

Version 2.0

DECEMBER 21, 2020

Table of Contents

LIST OF ABBREVIATIONS 1

OVERVIEW OF CHANGES FROM PRIOR VERSION OF SAP 2

1.0 SUMMARY OF STUDY DESIGN AND PROCESSES 3

1.1 Study Objectives 3
1.2 Study Design and Intervention 3
1.2.1 Study Design and Randomization 3
1.2.2 The PROUD Intervention 3
1.3 Sample and Sample Size 5

2.0 GENERAL PROCEDURES AND DEFINITIONS 6

2.1 Intent-to-treat (ITT) Analysis 6
2.2 Study Day 1 6
2.3 Pre-randomization Period 6
2.3.1 Baseline Period 6
2.4 Follow-up or “Post-randomization” Period 6
2.5 Study Period 6
2.6 Data Collection Period 6

3.0 STUDY POPULATION 8

3.1 Assignment of Patients to Clinics 8
3.2 Objective 1 Study Sample 8
3.3 Objective 2 Primary Study Sample (“Pre-randomization Sample”) 8
3.4 Objective 2 Secondary Study Sample 8
3.4.1 Objective 2 “Post-randomization Sample” 9

4.0 OUTCOME MEASURES 10

4.1 Definition of Primary Implementation Outcome Measure 10
4.1.1 Algorithm for Calculating the Primary Implementation Outcome Measure 10
4.2 Definition of Objective 2 Effectiveness Outcome Measure (Powered Secondary Outcome) 12
4.2.1 Addressing Data Anomalies and Outliers 12
4.3 Definitions of Secondary Measures 13

5.0 ANALYSES OF OUTCOME MEASURES 16

5.1 Primary Analytic Method for Primary Implementation Outcome Measure 16
5.2 Secondary Analyses of Primary Objective 1 Outcome Measure 16
5.2.1 Adjustment for Covariates 16
5.2.2 Modified Analyses Accounting for Success of Implementation 19
5.2.2.a. Analysis During the Period in Which the NCM was Seeing Patients 19
5.2.2.b. Analysis Limited to Clinics that Successfully Implemented the MA Model 19
5.2.3 Sensitivity Analysis Addressing the Algorithm for Assigning Patients to Clinics 19

5.2.4 Sensitivity Analyses Focused on Assumptions Made in Estimating Patient-Years of OUD Medication Treatment (Objective 1: Main Implementation Outcome) 20
5.2.4.a. Omitting Last Order in an Oral Buprenorphine Treatment Episode 20
5.2.4.b. Vary the Allowable 7-day Gap to Define Continuous Use Episodes 20
5.2.4.c. Incorporating Dispensings Data to Define Days of OUD Treatment 21
5.2.4.d. Incorporating Limited Data on Methadone Maintenance Therapy (MMT) 21
5.2.4.e. Combined Sensitivity Analysis Using All Optimal Data 21
5.2.5 Timing of OUD Diagnosis 21
5.2.6 Secondary Analyses Among Patients Treated for OUD 22
5.3 Primary Analytic Method for Objective 2 Primary Effectiveness Outcome Measure ... 22
5.4 Secondary Analyses of Objective 2 Primary Effectiveness Outcome Measure within the Primary Objective 2 Sample 23
5.4.1 Objective 2 Covariate-adjusted Model 23
5.4.2 Objective 2 Sensitivity Analysis Among “Active” Patients 24
5.4.3 Objective 2 Sensitivity Analysis Among Patients Enrolled in the HCS Health Plan 24
5.4.4 Objective 2 Sensitivity Analysis Addressing Clinic Assignment/crossover 25
5.4.5 Objective 2 Secondary Modified Analyses Accounting for Success of Implementation 25
5.4.6 Objective 2 Sensitivity Analysis Applying Stricter Eligibility Criteria 25
5.5 Secondary Analyses of Primary Objective 2 Outcome Measure within the Objective 2 Secondary Sample 25
5.5.1 Rationale for Conducting Analyses Within a Secondary Sample 26
5.5.2 Defining Covariates for the Objective 2 Secondary Sample That Includes Patients Not Seen in the Health System Pre-randomization 26
5.5.3 Primary Objective 2 Outcome Analysis within the Secondary Sample 27
5.5.4 Sensitivity Analyses of Primary Objective 2 Outcome Measure within the Objective 2 Secondary Sample 28
5.5.4.a. Restricting to Patients in the HCS Pre-randomization 28
5.5.4.b. Secondary Analyses Accounting for Implementation and Other Sensitivity Analyses 28
5.6 Analyses of Secondary Outcome Measures 28
5.7 Effect Modification and Subgroup Analyses 28
5.7.1 Objective 1 Primary Implementation Outcome 28
5.7.2 Objective 2 Primary Effectiveness Outcome 30
6.0 POWER CONSIDERATIONS 31
6.1 Power Simulations for Primary Objective 1 Outcome 31
6.1.1 Data-generation and Analysis Model 31
6.1.2 Parameters Used to Generate the Simulated Data 32
6.1.3 Results of Simulations 34
6.1.4 Potential Exclusion of One Health Care System 34
6.2 Power Simulations for Primary Objective 2 Outcome 35

6.2.1 Description of Power Simulation ... 35
6.2.2 Results of Power Simulations ... 37
7.0 DESCRIPTIVE ANALYSES ... 39
7.1 Analyses of Demographic and Baseline Data ... 39
7.2 Crossover ... 39
7.2.1 Main measures of Crossover of Patients with OUDs and Treated for OUDs ... 39
7.2.2 Other General Measures of Crossover ... 39
7.2.3 Crossover of Providers ... 39
7.3 Implementation ... 40
7.4 Characteristics of Treated Patients ... 40
7.5 Descriptive Analysis of Outcomes Among Treated Patients ... 40
7.6 Descriptive Information on Data Sources ... 41
7.7 Health Care System-specific Descriptive Analyses of Outcome Measures ... 41
8.0 OTHER CONSIDERATIONS ... 42
8.1 Missing Data ... 42
9.0 SAFETY AND INTERIM ANALYSES ... 44
10.0 Table Shells ... 45
10.1 Objective 1 Paper ... 45
10.2 Objective 2 Paper ... 55
11.0 REFERENCES ... 67

Appendix A. Algorithms for Days of OUDs Treatment ... 69
Appendix B. Initial Values for Power Simulations ... 85
Appendix C. Small-sample Degree of Freedom (DF) Correction Method for Objective 2 Primary Analysis ... 86

Listing of Tables

Table 1: Data Collection Timeline ... 7
Table 2: Primary, Secondary, and Other Outcomes Measured During Post-Randomization Period Based on EHR Data (*measures from clinicaltrials.gov) ... 13
Table 3: Number of Days Treated for OUDs (with buprenorphine or injectable naltrexone) per Patient Seen at 2 Phase 1 Clinics in Each of 6 Health Care Systems (HCS) ... 32
Table 4: Parameters Explored in the Power Simulation ... 34
Table 5: Power Results for a 0.05-level One-Tailed Test, Based on 10,000 Iterations Per Cell ... 34
Table 6: Power Results for a 0.05-level One-Tailed Test, Based on 10,000 Iterations Per Cell with *Five* Health Care Systems ... 35
Table 7: Missingness in Covariates among Primary Care Patients with a Visit to one of the 12 Randomized Clinics During the Pre-randomization Period (N = 291,136 patients), Based on Data Pull 4 ... 43
Table A1: Buprenorphine Formulations Included for OUD Treatment ... 69

Listing of Figures

Figure 1: Data Collection Period 7
Figure 2: Objective 2 Power Across Different Effect Sizes 37
Figure C1. Type 1 Error Rates from the Setting with Data-generating Model that Includes a Single Person-level Covariate...86
Figure C2. Type 1 Error Rates from the Setting with Data-generating Model that Includes Multiple Person-level Covariate..87

LIST OF ABBREVIATIONS

BMC	Boston Medical Center
BUP	Buprenorphine
CONSORT	Consolidated Standards of Reporting Trials
CTN	Clinical Trials Network
DEA	Drug Enforcement Agency
DD	Daily Dose
ED	Emergency Department
EHR	Electronic Health Record
EMR	Electronic Medical Record
FDA	Food and Drug Administration
FY	Fiscal Year
GLMM	Generalized Linear Mixed-effect Models
HCPCS	Healthcare Common Procedure Coding System Codes
HCS	Health Care System
IRB	Institutional Review Board
ITT	Intent-To-Treat
MA Model	Massachusetts Model
NCM	Clinic-based Nurse Care Manager
NDC	National Drug Code
NRUPC	Non-randomized usual primary care clinics
NTX	Naltrexone
OUD	Opioid Use Disorder
PC	Primary Care
PI	Principal Investigator
PPD	Pills Per Day
PROUD	Primary Care Opioid Use Disorders Treatment
QIT	Quality Improvement Team
SAP	Statistical Analysis Plan
TA	Technical Assistance
UPC	Usual Primary Care (Control Condition)
XR	Extended release

OVERVIEW OF CHANGES FROM PRIOR VERSION OF SAP

Version 1.0 of the SAP described the analysis related to the primary Objective 1 outcome of the clinic-level number of days of OUD treatment. The current version of the SAP updates the power calculations for Objective 1 addressing an error in the power calculations in alignment with the Protocol modification. The current version of the SAP also includes descriptions of the following:

- Descriptive analyses
- Augmentation of the intervention
- Additional pre-planned secondary analyses of the primary Objective 1 outcome
- Analytic plans for the main Objective 2 effectiveness outcome of acute care utilization
- Analytic plans for secondary outcome measures for Objectives 1 and 2
- Shell tables for the main Objective 1 and Objective 2 papers

1.0 SUMMARY OF STUDY DESIGN AND PROCESSES

1.1 Study Objectives

This implementation trial is a Hybrid type III, blending implementation and effectiveness objectives in a single trial, but with an emphasis on implementation objective.

The **implementation objective** of the PROUD trial (Objective 1; primary aim) is to evaluate whether the PROUD intervention designed to implement the Massachusetts (MA) Model[1] of collaborative care for management of opioid use disorders (OUDs) in primary care (PC; the “PROUD intervention”) increases OUD treatment with buprenorphine or extended release (XR) injectable naltrexone (XR-NTX), documented in the electronic health records (EHRs) of PC patients, over a 2-year follow-up, as compared to usual PC (UPC).

The **effectiveness objective** (Objective 2; powered secondary aim) is to test our hypothesis that PC patients with OUDs in the 3 years prior to randomization who receive care in PROUD intervention clinics, compared to those who receive care in UPC clinics, will have fewer days of acute care utilization (including urgent care, emergency department [ED] and hospital care) in the 2 years after randomization. This “effectiveness” objective assesses whether implementation of the MA Model improves patient outcomes.

Secondary objectives explore assumptions in choice of outcomes and analyses, as well as providing descriptive, explanatory, and exploratory secondary outcomes and analyses.

Observational study aims include comparing PROUD trial (randomized) clinics to non-randomized usual primary care (NRUPC) clinics and comparing outcomes of PROUD intervention clinics to non-randomized “exemplar” clinics selected by the health systems as providing quality care for patients with OUD. These observational analyses are described in separate statistical analysis plans outside of this main trial SAP.

1.2 Study Design and Intervention

1.2.1 Study Design and Randomization

The PROUD trial is a hybrid type III pragmatic, cluster-randomized, quality improvement trial. Hybrid type III trials are mixed effectiveness and implementation trials, with greater emphasis on implementation.[2] The trial is conducted in six health systems across the United States. Randomization is stratified by health system. Each health system has recruited 2 PC clinics (or a cluster of smaller clinics) willing to implement collaborative care for patients with OUDs using a model developed at the Boston Medical Center (BMC) in Massachusetts and spread across federally qualified health clinics in that state (the “MA Model” hereafter). One of the two recruited PC clinics in each health system is randomized to implement the MA Model, while the other continues with UPC.

All quantitative data for sample identification and outcome measures are derived solely from existing electronic health records (EHRs), which include but are not limited to electronic administrative data, patients’ electronic medical records (EMRs), and/or electronic data on health insurance claims.

1.2.2 The PROUD Intervention

Intervention: Implementation of the MA Model of Collaborative Care for OUDs. The PROUD trial provides financial support to cover the salary of a Nurse Care Manager (NCM) and technical assistance (TA) for the duration of the study, but the health system—not investigators—implement

the MA Model program as part of quality improvement, and the health system and its clinicians provide all clinical care. One PC clinic or cluster of smaller clinics ("*PROUD intervention clinics*" hereafter) is randomized to the PROUD intervention in each health system and implements the MA Model after randomization. Specifically, the PROUD intervention includes 3 strategies used to implement the Model in Massachusetts.

(1) Clinic leadership receives funding for a 1.0 full time equivalent NCM for 2 years after randomization and technical support for recruiting and hiring the NCM. Once hired for the study, the NCM will receive TA from experts in Massachusetts supported by PROUD, but NCMs will be employed and supervised by the health system.

(2) Experts at BMC who originally developed and disseminated the MA Model will: provide intervention clinics with a MA Model Manual; train PROUD NCMs at BMC for 1.5-2 days; and provide the ongoing TA for 2 years after randomization.

(3) At least three PC providers in the PROUD intervention clinic agreed to obtain DEA waivers to prescribe buprenorphine for OUDs (if not already waivered) and work closely with the NCM to offer high quality PC care for OUD (e.g., medication treatment with buprenorphine or injectable naltrexone with close follow-up to maximize retention in treatment), if randomized to the PROUD intervention.

Augmentation of the intervention to increase support to NCMs. PROUD included pre-planned, ongoing formative evaluation by an Implementation Monitoring Team to assess whether the 3 implementation strategies were adequate, or whether they needed to be adapted or augmented. As of early July 2019, none of the sites were consistently meeting the goal of seeing 1-2 new patients with OUD a week. A modification to the intervention was therefore approved by the IRB (7/31/2019) to add a new implementation strategy: recommending weekly interdisciplinary quality improvement meetings in the intervention clinics. Holding weekly quality improvement meetings is an evidence-based approach to quality improvement in primary care, and it was an affordable option within the PROUD budget. Health system lead investigators and project managers were asked to arrange quality improvement meetings with the intervention clinics with the goal of increasing the number of patients with OUD being treated by the clinic. The recommended elements of the quality improvement meetings were as follows. (1) Site lead investigator(s) and project managers work with clinic leaders to identify an interdisciplinary quality improvement team (QIT) consisting of champions from the intervention clinic (medical assistants, primary care waivered prescribers, front desk staff, etc.), the NCM, other clinical leaders, and themselves. (2) The QIT holds weekly meetings, ideally for an hour but at a minimum of 30 minutes, to review quality improvement activities from the past week and plan quality improvement activities for the next week using the plan-do-check-adjust (PDCA) cycle approach. (3) A note taker sends an email summarizing actions taken, results, and next steps/action items to the Boston TA Team after each meeting (cc'ing the PROUD study email box). The TA team may—as time allows at their weekly TA meetings with NCMs—ask the NCMs to share their experiences with the weekly QIT meetings, highlighting successes and lessons learned. All sites agreed to bring together an interdisciplinary group of stakeholders to help support the NCMs in regular quality improvement meetings, and some sites sent action items to the Boston TA team.

Comparison: Usual Primary Care (UPC). Clinics randomized to UPC do not receive any resources or support from the study but are free to improve OUD care in any way they choose, but they are asked not to use the OBAT manual from Boston Medical Center to replicate the PROUD intervention in the Usual PC clinic. UPC is the appropriate comparison to evaluate the

impact of implementation of the MA Model on access to and quality of OUD care because most PC clinics do not currently offer treatment for OUDs, but that could change over the course of the trial.

1.3 Sample and Sample Size

The sample for the trial consists of patients who have visited the PROUD trial PC clinics in the six participating health systems. Health systems were selected for the PROUD trial based on 1) leadership support for participating in the trial, 2) elements of clinic eligibility such as adequate size and having at least 3 PC providers willing to prescribe buprenorphine in each of the PC clinics, and 3) a demonstrated ability to obtain the secondary data necessary for the PROUD trial measures—specifically days of OUD treatment with buprenorphine and injectable naltrexone and days of acute care utilization. Smaller clinics were eligible if a group of clinics near each other included adequate numbers of patients (target ~10,000 unique patients with visits in a year), and were willing to participate as a single clinic for purposes of this trial, that is, if selected to implement the MA Model, a nurse care manager (NCM) would be shared between 2 intervention clinics.

PC patients 16-90 years old with at least 1 visit to the participating clinics from 3 years before to 2 years after randomization (the 5-year study period) will be included in the trial. The total sample of PC patients in the trial is anticipated to be over ~170,000 patients across the 12 clinics, since over 14,000 patients were seen, on average, in each clinic in 2016. The implementation objective is addressed in the total trial sample, while the secondary objective is evaluated in the subsample of trial patients who have EHR documentation of an OUD during the pre-randomization period.

2.0 GENERAL PROCEDURES AND DEFINITIONS

2.1 Intent-to-treat (ITT) Analysis

Unless otherwise specified, all analyses will follow an intent-to-treat principle whereby clinics (and patients therein) will be analyzed according to the treatment arm to which they were randomized regardless of the subsequent sequence of events.

2.2 Study Day 1

The randomization date is defined as study day 0 and study day 1 is defined as the day after randomization.

2.3 Pre-randomization Period

The pre-randomization period is defined as the period 3 years prior to randomization through study day 0 (randomization date), except at one health system that changed EHR systems where the pre-randomization period is limited to 2.8 years. For simplicity, throughout the SAP this pre-randomization period is referred to as 3 years. The pre-randomization period is used to define the Objective 2 primary sample. Including the randomization date in the pre-randomization period is appropriate because health systems were notified of their randomization status the day after the randomization date.

2.3.1 Baseline Period

Because all clinics have data 2 years prior to randomization through study day 0, many covariates for descriptive analyses and regression adjustment are defined over the 2 years prior to randomization through study day 0 (since all clinics have data from this period), referred to as "baseline" measures.

2.4 Follow-up or "Post-randomization" Period

Unless otherwise specified, the follow-up period is defined as the period from study day 1 to 2 years after the randomization date (or 1.5 years for one health system that randomized 6 months late). For simplicity, throughout the SAP this follow-up ("post-randomization") period is referred to as 2 years.

2.5 Study Period

The study period is defined as up to 3 years pre-randomization (3 years at 5 health systems and 2.8 years at 1 health system) through up to 2 years following randomization (2 years at 5 health systems and 1.5 years at 1 health system). The full study period of up to 5 years—defined as the start of the pre-randomization period to the end of the follow-up period for each health system—is used to define the Objective 1 sample.

2.6 Data Collection Period

For eligible patients (defined in Section "Study Population"), data are collected from 3 years prior to randomization through 2 years post randomization (Figure 1). Limited datasets are extracted from the EMR and insurance claims data during four interim time points ("Data Pulls" 1-4) during the post-randomization period for reports to the Data and Safety Monitoring Board and refinement of data specifications and measures (Table 1). Objective 1 and 2 analyses use data from Data Pull 5 collected after the end of follow-up.

Figure 1: Data Collection Period

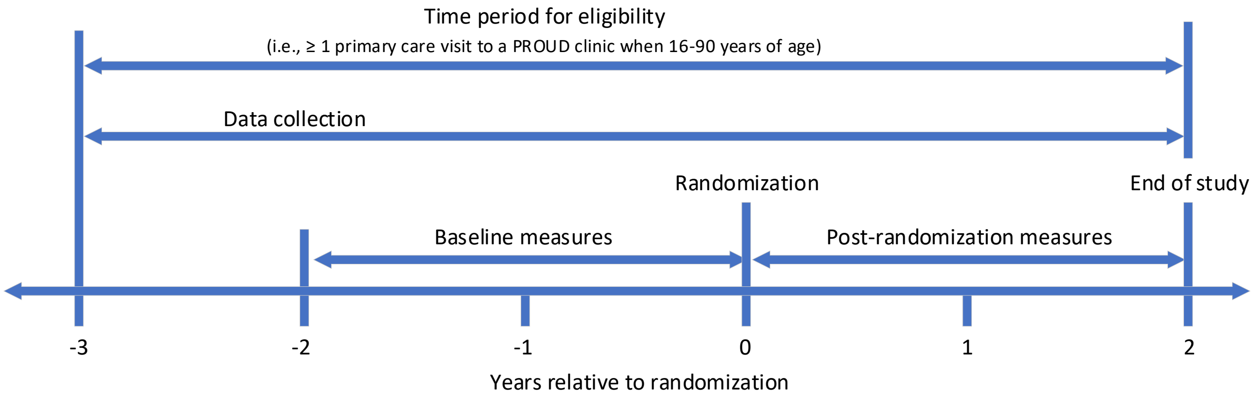

Table 1: Data Collection Timeline

Data Pull Number	Cohort Eligibility*	Data Collection*	When data are transferred to Lead Node (Post randomization)
1	T-3 to T0	T-3 to T0	6 mo
2	T-3 to T0.5	T-3 to T0.5	12 mo
3	T-3 to T1	T-3 to T1	18 mo
4	T-3 to T1.5	T-3 to T1.5	24 mo
5	T-3 to T2	T-3 to T2	33 mo

*T0 is randomization date.

3.0 STUDY POPULATION

Patients are eligible for inclusion in the trial if:

1) they had a PC visit at a PROUD trial PC clinic in the pre-randomization and/or follow-up period (defined above), and
2) age is 16 to 90 years at the time of a PC visit during the study period.

Eligible PC visits refer to outpatient clinic visits (in which the patient was age 16 to 90 on the day of the visit) to the departments of adolescent and pediatric medicine (including teen clinics), family practice, geriatric, general practice, or internal medicine that are provided by a physician, physician assistant, nurse practitioner, resident or fellow with one of these specialties.

3.1 Assignment of Patients to Clinics

Patients will be assigned to clinics based on the number of eligible PC visits. In the rare case that a patient had PC visits to both PROUD Intervention clinics and UPC clinics, they will be assigned the clinic which they visited the most pre-randomization (if they visited during that period), and if they are tied, the clinic visited nearest to and preceding the time of randomization will be considered the patient's PC clinic. If a patient only had PC visits to a trial clinic post-randomization but not pre-randomization, and they visit both PROUD and UPC clinics, they will be assigned to the clinic they visited the most, and if tied they will be assigned to the clinic they visited last.

3.2 Objective 1 Study Sample

The primary implementation (Objective 1) outcome, along with secondary implementation measures, will be analyzed among the full sample of eligible patients defined above.

3.3 Objective 2 Primary Study Sample ("Pre-randomization Sample")

The primary effectiveness (Objective 2) outcome, along with secondary effectiveness measures, will be analyzed in the subset of patients from the full sample of eligible patients who had (1) an eligible PC visit at a PROUD trial PC clinic during the pre-randomization period, (2) a documented OUD diagnosis during the pre-randomization period, and (3) who were age ≥16 as of the start of the baseline period (two years pre-randomization). This latter criterion ensures that the patient has at least 2 years pre-randomization in which to have an eligible PC visit (in which they are ≥16 years of age). Clinic assignment for the Objective 2 primary sample will be the same as above. Because study sample is defined using pre-randomization data, this sample is also referred to as the Objective 2 pre-randomization sample.

3.4 Objective 2 Secondary Study Sample

The Objective 2 primary sample as defined in the preceding section, which restricts analyses to patients seen in PROUD trial clinics with documented OUD pre-randomization, was selected for primary analyses to avoid the potential for *identification bias*[3] (type of selection bias that can occur when the analytic sample is defined using post-randomization data). However, an important limitation of analyses within the Objective 2 primary sample is that they miss any impact of the PROUD intervention among patients with a new OUD diagnosis post-randomization, including patients who initiate treatment in PROUD intervention clinics who are diagnosed with OUDs after randomization, and patients who are newly attracted to the clinic specifically because of the PROUD intervention. To address this limitation, secondary analyses are conducted in a broader sample that includes patients new to the PROUD trial clinics or with newly recognized OUDs post-randomization (see Figure Shell 1, Objective 2 paper, Section 10.2).

Specifically, the effectiveness (Objective 2) secondary study sample is defined as the subset of patients from the main trial sample who had a documented OUD diagnosis during either the pre-randomization or follow-up periods and who were age ≥16 as of the start of the baseline period. Clinic assignment for the Objective 2 secondary sample will be the same as above.

3.4.1 Objective 2 “Post-randomization Sample”

We note that the Objective 2 secondary study sample is a broader sample that includes the Objective 2 primary “pre-randomization” study sample as a subset (Figure Shell 1, Objective 2 paper, Section 10.2). We refer to patients who are included in the secondary study sample but who are not included in the primary pre-randomization sample as having “newly recognized OUD in trial clinics post-randomization,” and we refer to this sample of patients as the “post-randomization” Objective 2 sample. This post-randomization sample consists of patients who were new to the trial clinics or with newly documented OUD post-randomization (see Supplemental Figure Shell 1**,** Objective 2 paper, Section 10.2, which illustrates the different ways patients can enter the post-randomization sample).

4.0 OUTCOME MEASURES

4.1 Definition of Primary Implementation Outcome Measure

The primary outcome (clinic-level measure) is the number of patient days of OUD medication treatment documented in the EHR in each clinic post-randomization. To account for varying clinic sizes, the clinic-level outcome is divided by the number of patients seen in the clinic post-randomization and then multiplied by an appropriate scaling factor in order to report the results (e.g., multiplying by 10,000 to calculate the number of patient days of OUD treatment per 10,000 patients), and reported as patient-years of treatment provided by a clinic (calculated by simply dividing days of OUD treatment by 365).

“OUD medication treatment” includes medications for OUD that can be prescribed in PC and documented in EHRs—buprenorphine formulations indicated for OUD (oral, implants, sustained release injection with or without an OUD diagnosis) or injectable extended release (XR) naltrexone with a diagnosis of OUD. An OUD diagnosis is required for injectable XR naltrexone because it is often used for alcohol use disorders (AUD). We do not require an OUD diagnosis for buprenorphine because there are buprenorphine formulations specific for OUD and PROUD Phase 1 analyses revealed OUD diagnoses are often missing, consistent with the literature.[4] Further, an OUD diagnosis is likely to be documented by PROUD clinic providers based on the PROUD NCM manual, so that requiring an OUD diagnosis could bias findings toward favoring the intervention clinics.

4.1.1 Algorithm for Calculating the Primary Implementation Outcome Measure

Two FDA-approved treatments for OUDs that can be provided in medical settings and documented in EHRs are included as OUD treatment. Buprenorphine and injectable XR naltrexone use will be determined from medication orders (EHR data) and procedures (EHR for all health systems and claims data for 2 health systems). Text string searches on generic and brand medication name will be used to ascertain buprenorphine formulations indicated for OUD treatment (sublingual, tablet, film, subdermal implants, and subcutaneous) and injectable XR naltrexone use from medication orders. A clinical co-investigator then reviews all hits on the text string search as part of quality control. Procedure codes will also be used to identify injectable formulations, implants, and oral formulations given in the office setting. Pharmacy dispensings are the gold standard for outpatient non-injectable medication use but they are not comprehensively available for 5 of the 6 health systems (whereas medication orders are routinely available in all 6 health systems). These 4 health systems are not insurers and therefore do not receive claims from outside pharmacies. Site (health system) principal investigators state that patients frequently obtain medications from pharmacies outside of the health system.

A single medication order or procedure code for buprenorphine formulations indicated for OUD treatment or injectable XR naltrexone (the latter with an OUD diagnosis) will be considered OUD treatment, though sensitivity analyses will examine this assumption (Section 5.2.4)

The algorithm for calculating the primary outcome measure of days on OUD medication (buprenorphine or injectable XR naltrexone) is as follows with details in Appendix A.

Ascertained from the EHR and claims as specified above, all buprenorphine and naltrexone XR injections will be quality checked, combined, cleaned and values imputed (as necessary), and collapsed into an episode or episodes (if gaps in use) of OUD treatment.

Episodes of OUD treatment will then be summed to calculate the total patient days covered with OUD treatment in the time-period of interest. Details on estimating episodes of treatment and summing episodes are in Appendix A but briefly they will be estimated as follows from variables commonly available in the EHR – medication orders (i.e., medication name, date ordered, form, quantity, strength, strength unit, number of refills, and directions for use [SIG]) and procedures (medication name, form, strength, strength unit, and date administered).

Specifically, we will do the following:

1) Perform quality checks for outlier/implausible values for variables required to calculate the outcome and impute outliers/implausible values and missing values. See Appendix A for further details.
2) Estimate days' supply for orders of buprenorphine film and sublingual tablets.
    a. Translate directions for use (SIG) into pills per day (PPD). For example, "Take 1 pill twice a day" is a PPD=2.
    b. Divide the quantity field by PPD to estimate days' supply. Add any additional day's supply from refills on the original order to estimate the total days' supply of the order. For example, an order with quantity=60, PPD=2, and refills=2 would have a day's supply=90.
3) Assign naltrexone injection and buprenorphine subcutaneous Sublocade a day's supply=28.
4) Assign Probuphine (implant) a day's supply=180. Assign Brixadi a day's supply of 7 or 28 depending on the product.
5) Estimate runout dates (date when the medication supply provided runs out) as order/procedure date plus days' supply minus 1. An order (or dispensing in secondary measures for sensitivity analyses) on 1/1/2018 with a day's supply = 15 is estimated to runout on 1/15/2018.
6) Prior to dropping injectable XR naltrexone orders or procedures, we will describe patients with both OUD/OD and AUD diagnosis codes and adjudicate if needed
    a. For patients with 1+ injectable naltrexone orders or procedures and both 2+ OUD/OD and 1+ AUD diagnoses, provide a table of the numbers of OUD/OD and AUD diagnoses in 2 years pre-randomization and a similar table for post-randomization users of naltrexone.
    b. Adjudicate subjects with both 2+ OUD/OD and 1+ AUD diagnosis codes to decide whether to include or exclude as OUD treatment, using the number of times a code was used from 7a.
    c. Drop remaining injectable XR naltrexone if OUD diagnosis criteria are not met:
        i. Drop injectable XR naltrexone in the pre randomization period if there are not 2+ visits with an OUD/opioid overdose (OD) diagnosis (can be 1 OUD and 1 OD code) in the pre randomization period.
        ii. Drop naltrexone injections in the post randomization period if there are not 2+ visits with an OUD/OD diagnoses (can be 1 OUD and 1 OD code) in the pre or post randomization period.

7) In the pre and post-randomization periods, create continuous use episodes defined as OUD treatments with gaps ≤7 days between the runout of one order/procedure and the start date of the subsequent (other cut points to be evaluated in sensitivity analyses after summarizing the distribution of gaps). For example, two OUD treatment episodes with treatment episode 1 start 1/1/2018 and runout 1/15/208 and treatment episode 2 starting 1/17/2018 and runout 2/5/2018 are rolled into one episode of continuous treatment (start 1/1/2018 and end 2/5/2018). In general, this gap applies to same medication orders/procedures and different medications.
    a. One exception is that gaps of ≤14 days between the runout of a buprenorphine order and subsequent naltrexone XR injections are allowed in defining continuous use episodes to account for required washout periods prior to naltrexone XR injection.
8) Pre and post randomization episodes are not allowed to overlap. They will be left and right censored accordingly. For example, a treatment episode of 90 days with 15 days in the post randomization period (includes randomization date) will be split into two episodes with one episode contributing 75 days of treatment pre-randomization and a second episode contributing 15 days of treatment post randomization.
9) Add the number of days of each continuous use episode to arrive at total days of OUD treatment in the distinct periods of pre-randomization and post-randomization. The algorithm does not allow for double counting of any overlapping orders or procedures.

4.2 Definition of Objective 2 Effectiveness Outcome Measure (Powered Secondary Outcome)

The primary effectiveness (Objective 2) endpoint is a person-level count measure of the number of days of acute care utilization in the follow-up period, among patients with an OUD diagnosis pre-randomization. This measure includes visits to urgent care clinics or emergency departments (EDs), as well as days hospitalized. Acute care utilization will be determined from the EMR and insurance claims data when available.

For hospitalizations, the number of inpatient days will be the number of days from admission to discharge, inclusive. For urgent care or ED (referred to collectively as “emergency care”), each unique date with a visit to an urgent care or ED will be counted as 1 day (even if the patient stays overnight), except in the uncommon event that an ED visit spanned 3 or more days (< 0.5% of ED records collected in Data Pull 4, including both randomized and non-recruited UPC clinics), in which case the encounter will be classified as a hospitalization (as patients may be boarded in an ED until a hospital bed becomes available and some are subsequently discharged from the ED if no hospital bed ever became available). If a patient is admitted from urgent care or an ED, the ED or urgent care day is not (double) counted when counting the number of days of acute care.

4.2.1 Addressing Data Anomalies and Outliers

In examining the hospitalization data during interim Data Pulls, a few data anomalies were discovered (e.g., extremely long hospitalizations). Records with an anomaly will first be manually reviewed by health system study staff and corrected (where possible) or handled as follows:

- Discharge dates before the admission date: We assume the dates were incorrectly reversed and switch them back (< 50 records in Data Pull 4 out of over 300,000), unless

the resulting length of stay is an outlier (identified by comparing to the distribution in the full sample), in which case a basic imputation approach will be applied (defined below).

- Missing discharge date: most hospitalization records with a missing discharge date were embedded within another hospital record; after removing these and collapsing across any overlapping records, < 0.5% of the remaining ~275,000 hospitalization records (in Data Pull 4) had a missing discharge date. For these records length of stay will be imputed (details below).

In addition, hospital records with long inpatient stays (≥180 days) will be manually reviewed by the health system to determine whether there may have been a data error.

Imputation approach: For the rare scenarios with data anomalies, we will impute the median length of stay based on inpatient episodes among patients within the same health care system (HCS), stratified by whether the patient had any OUD diagnosis during the study period.

4.3 Definitions of Secondary Measures

Additional secondary measures will be analyzed to describe outcome measures reflecting processes of care, implementation, and effectiveness. The following table describes each of the measures that will be examined either descriptively, or through formal modeling, as described in the statistical analysis section below. Measures will be calculated broadly in the full study sample to facilitate analyses across different study samples. For example, we will calculate the number of days of OUD medication treatment for each patient, which will be summed across patients as part of the primary Objective 1 outcome and will also be analyzed at a patient-level among patients with an OUD diagnosis pre-randomization. Other implementation measures (e.g., number of patients treated) will be analyzed in both the full study sample of all patients with a visit, as well as in the subsample of these patients who had an OUD diagnosis pre-randomization.

Table 2: Primary, Secondary, and Other Outcomes Measured During Post-Randomization Period Based on EHR Data (*measures from clinicaltrials.gov)

Outcome Measures
***Objective 1. Patient-days of OUD medication treatment (primary outcome).** Clinic-level number of patient-days of OUD treatment with buprenorphine and XR-NTX documented in the EHR during the period from randomization until two years after, reported per 10,000 PC patients in the clinic in the two years post-randomization.
***Objective 2. Acute care utilization (secondary outcome).** Patient-level number of days of acute care utilization during the period from randomization until two years after, among patients with an OUD diagnosis documented in the EHR in the three years prior to randomization.
**Other Outcome Measures of Implementation**
***Newly diagnosed OUD (Implementation Reach).** Clinic-level number of patients with a new International Classification of Disease (ICD) code for OUD documented in the EHR during the period from randomization until two years after who did not have an OUD diagnosis documented in the EHR in the three years prior to randomization, reported per 10,000 patients in the PC clinic in the two years post-randomization.

This measure will also be estimated for any OUD diagnosis (new and previous diagnoses).
***Initiation[§] of OUD treatment (Implementation Reach).** Clinic-level number of patients who initiate: (1) buprenorphine or (2) XR-NTX with an indication of OUD as documented in the EHR during the period from randomization until two years after, reported per 10,000 PC patients in the clinic in the two years post-randomization.<br>This measure will also be estimated for any OUD treatment (initiation and on-going treatment).<br>[§]*Initiation of buprenorphine and* XR-NTX *in the context of PROUD trial outcome measures refers to the first order for OUD medication treatment post-randomization with no treatment with these medications in the prior 365 days (including pre-randomization)*. *See Section 4.1.1. for operationalization of OUD in this context.*
**Buprenorphine daily dose of 16 mg or more (Implementation Reach).** Clinic-level number of patients on ≥ 16 mg per day of buprenorphine at any time during the period from randomization until two years after randomization as documented in the EHR, reported per 10,000 PC patients in the clinic in the two years post-randomization.
**Retention measures of OUD treatment (Implementation Fidelity).**<br>***Retention in OUD treatment**. Clinic-level number of patients initiating[§] OUD treatment during the period from randomization until two years after randomization as documented in the EHR, who also receive OUD treatment on 80% of days available after initiation, reported per 10,000 PC patients in the clinic in the two years post-randomization [5].<br>**Retention in OUD treatment for ≥ 6-months**. Clinic-level number of patients initiating[§] OUD treatment during the period from randomization until two years after randomization as documented in the EHR, who remain on treatment for ≥ 6 months after initiation, reported per 10,000 PC patients in the clinic in the two years post-randomization.***<br>**Discontinuation of OUD treatment**. Clinic-level number of patients initiating[§] OUD treatment during the period from randomization until two years after randomization as documented in the EHR, who discontinue treatment (defined as a gap of 60+ days), reported per 10,000 PC patients in the clinic in the two years post-randomization.<br>[§]*See definition of initiation above;*<br>****Excludes patients whose only eligible PC visit occurred in the last 6 months of the post-randomization period or who began OUD treatment in the last 6-months of the post-randomization period. Measures of retention above will also be estimated for all subjects regardless of new or on-going treatment and for subjects with on-going treatment.*
**Number of buprenorphine prescribers (Implementation Fidelity).** Clinic-level number of buprenorphine prescribers[†] during the period from randomization until two years after randomization as documented in the EHR, who prescribe buprenorphine, reported per 10,000 PC patients in the clinic in the two years post-randomization.<br>Number of buprenorphine prescribers will also be reported per X total prescribers in the clinic in the two years post-randomization.<br>[†]*Prescribers determined from medication orders in the electronic health records. Providers assigned to clinics based on number of visits with patients in the clinic pre-randomization.*
***Naloxone prescribing (Implementation Fidelity).** Patient-level number of prescriptions of naloxone for overdose management in the period from randomization until two years after, among patients with an OUD diagnosis in the three years prior to randomization.

**OUD treatment duration (Implementation Fidelity).** Patient-level number of days of OUD treatment with buprenorphine and XR-NTX documented in the EHR during the period from randomization until two years after, among patients with an OUD diagnosis in the three years prior to randomization. This measure will be modeled as a categorical variable (0 days, 1-30 days, 31-90 days, 91-180 days, ≥180 days).
**Other Outcome Measures of Effectiveness**
***Urgent care or ED use**[**]**.** Patient-level number of visits to urgent care or EDs during the period from randomization until two years after, among patients with an OUD diagnosis documented in the EHR in the 3 years prior to randomization. Urgent care and ED are combined into a single outcome to represent “emergency care”.<br>This measure will secondarily be modeled as a categorical variable (with cut-points based on the empirical distribution).<br>[**] *ED or Urgent Care visits that lead to hospitalization are classified as inpatient.*
***Inpatient days hospitalized**[**]**.** Patient-level number of days hospitalized during the period from randomization until two years after, among patients with an OUD diagnosis documented in the EHR in the three years prior to randomization.
**Any acute care.** Patient-level binary indicator for whether the patient had any acute care utilization during the period from randomization until two years after, among patients with an OUD diagnosis documented in the EHR in the three years prior to randomization.
**Number of hospitalizations**[**]**.** Patient-level number of hospitalizations during the period from randomization until two years after, among patients with an OUD diagnosis documented in the EHR in the three years prior to randomization. This measure will be modeled as a categorical variable (with cut-points based on the empirical distribution).

5.0 ANALYSES OF OUTCOME MEASURES

5.1 Primary Analytic Method for Primary Implementation Outcome Measure

Since randomization and the intervention occur at the clinic-level, the unit of analysis will be clinic and not patient for the primary outcome. The mixed effects model[6] evaluating the effect of the PROUD intervention is:

$$y_{ij} = \alpha + \beta * trt_{ij} + \gamma * z_{ij} + \theta_j + \epsilon_{ij} \quad (1)$$

where

1) y_{ij} is the observed value of the primary outcome measure for clinic i at health care system (HCS) j
2) trt_{ij} is the treatment indicator (PROUD intervention) for clinic i at HCS j
3) z_{ij} is the observed value of the primary outcome measure for the two years prior to randomization (hereafter baseline) for clinic i at HCS j
4) θ_j is the random effect for HCS j and is distributed $N(0, \sigma_\theta^2)$
5) ϵ_{ij} is the error term for clinic i at HCS j and is distributed $N(0, \sigma_\in^2)$

Our primary hypothesis is that there will be a significant increase in the number of patient days of medication treatment for OUDs during the follow-up period in clinics randomized to the PROUD intervention as compared to clinics randomized to UPC. To evaluate this hypothesis, analyses test whether β (PROUD intervention effect) significantly exceeds zero using a one-sided hypothesis test at the 0.05 level. This is appropriate because our primary aim is to test superiority of implementation of the MA model relative to Usual PC in order to inform health systems' decisions as to whether to implement this model of OUD care.

Example SAS code for the analysis is as follows:

```
proc mixed data = primout;
  class arm;
  model scaletotdays = arm basevalue / solution;
  random intercept / subject = site;
  estimate "Treatment Effect" arm 1 -1;
run;
```

where "arm" is the treatment assignment (trt_{ij}), "scaletotdays" is the value of the primary outcome measure (y_{ij}), "basevalue" is the value of the primary outcome measure for the two years prior to randomization (z_{ij}) and "site" indexes the different HCSs (j).

This model allows for clinics to be correlated within a HCS, in addition to allowing for an association of the scaled days of OUD treatment prior to randomization with the post-randomization outcome.

5.2 Secondary Analyses of Primary Objective 1 Outcome Measure

5.2.1 Adjustment for Covariates

A secondary analysis of the primary Objective 1 outcome measure will adjust for additional covariates that are associated with the primary outcome. This secondary analysis will be the same as the primary analysis (i.e., it will still adjust for the baseline value of the outcome and include HCS-specific random intercepts), except that it will include additional covariates. The primary goal of this secondary analysis is as a sensitivity analysis, in which we will investigate the degree to

which the treatment effect estimate (β) from the primary analysis changes after covariate adjustment; thus, we plan focus on the magnitude of the change in the estimate rather than conduct inference on the treatment effect estimated under this secondary analysis.

The following approach will be utilized to identify the covariates for potential inclusion in the secondary analytic model of the primary outcome measure. First, we will consider a candidate set of clinic-level covariates (described below). Because of our small sample size (n=12) for the clinic-level primary outcome, we are limited in the number of covariates we can include in the outcome model to avoid overfitting, which can lead to unstable coefficient estimates. Consequently, we plan to conduct baseline analyses (detailed below) to identify a subset of these predictors to adjust for. The goal of these baseline analyses is to identify which covariates are associated with the clinic-level number of patient years of OUD treatment during a 1-year period pre-randomization, after adjusting for this measure in prior baseline years (to mimic the primary analysis but using only baseline data). The planned baseline analyses will proceed as follows.

1) Consider the pre-randomization data from the two years prior to randomization (Period 1 = two years prior to randomization; Period 2 = year prior to randomization). Denote B1 as the “baseline” value of the outcome measure in Period 1 and B2 as the “baseline” value in Period 2. Obtain values of each of the clinic-level covariates being considered during the earlier baseline period (Period 1), such as the proportion of patients seen in the clinic during Period 1 who were female.

2) Conduct a regularized (lasso) regression of the B2 values on the B1 values as well as all of the candidate clinic-level covariates for Period 1 (earlier baseline period). Lasso regression avoids overfitting by penalizing the magnitude of the coefficients such that some of the coefficients may be shrunk to exactly zero.[7,8] The B1 value of the outcome will be “forced” into the model (i.e., the corresponding coefficient will not be penalized). We will use cross-validation initially to select the best-fitting value of the tuning parameter λ (which controls the number of non-zero coefficients[7]). Specifically, we will perform a “leave-one-cluster-out” cross-validation as follows. For each candidate value of the tuning parameter λ, we predict the B2 values of each HCS using a regularized mixed-effect model fitted with the data from all other HCSs. We then compute the mean squared error (MSE) of the prediction, and take the average of these MSEs across all HCSs (“the cross-validated MSE”), as the measure of the performance of the candidate value of the tuning parameter λ. We select the candidate value of the tuning parameter λ with the smallest cross-validated MSE.

3) If the selected value of the tuning parameter λ is such that there are more than 2 non-zero coefficients, we plan to vary the value of λ such that no more than 2 covariates have non-zero coefficients. These 2 covariates will be the ones that we include in the covariate-adjusted analysis (except that the time window for the covariates included in the outcome model will be over the full 2-year baseline period, rather than just over Period 1).

With 12 clinics and 4 variables (intervention indicator, baseline value of the outcome, plus 2 covariates), this proposed covariate-adjusted analysis would have 3 observations (clinics) per variable. Although there is no firm rule on the maximal number of variables able to be included in the model, recent literature has suggested that having as few as two subjects per variable in a standard linear regression model did not adversely impact parameter estimation.[9]

Candidate set of covariates: We identified a broad set of covariates based on known and hypothesized predictors of initiation in and retention to medication treatment for OUDs from the literature and discussions with study co-investigators. Each covariate will be defined at a patient level, and then aggregated into Period-specific (i.e., Period 1 and Period 2) clinic-level measures (e.g., mean age of patients seen in a clinic in Period 1 or 2, proportion of patients with ≥1 depression diagnosis) to be considered for the covariate-adjusted analysis of the clinic-level outcome.

1) Age[1,10-15]
    a. Continuous: mean age
    b. Categorical: proportion in each group (<25, 25-44, 45-64, ≥65)
    c. Binary: proportion <45
2) Gender:[11,15] proportion female
3) Race/ethnicity:[1,11,13,15,16] proportion in each of the following groups:
    a. Hispanic
    b. Non-Hispanic Asian
    c. Non-Hispanic Black or African American,
    d. Non-Hispanic White,
    e. Non-Hispanic Other race (any of the following, as well as each of the following groups separately):
        i. American Indian / Alaska Native
        ii. Native Hawaiian or Pacific Islander
        iii. Multiple race
        iv. Other race
    f. Missing/Unknown
4) Number of days with an OUD diagnosis: proportion of patients with 0, ≥1, ≥2, ≥5, ≥10
5) Number of days with a diagnosis of each of the following conditions (using clinic-level proportions as for OUD above):[15]
    a. Depression
    b. Anxiety
    c. PTSD
    d. ADHD
    e. Other mental health conditions[17]
    f. Any mental health condition (a-e above)
    g. Alcohol use disorder
    h. Cannabis use disorder
    i. Stimulant use disorder
    j. Other (non-opioid) substance use disorder
    k. Any substance use disorder (g-j above)
        i. Also including OUD
        ii. Excluding OUD
    l. HCV
6) Number of days with an ICD code for housing instability / homelessness (using clinic-level proportions as for OUD above)
7) Neighborhood-level SES measures, deciles across patients of:

   a. median household income[18]
   b. percent below the federal poverty line
   c. percent unemployed[1,15,19]
8) Buprenorphine prescribers
   a. number in the clinic
   b. as a percentage of all prescribers in the PC clinic
9) Rurality of residence (rural urban commuting codes of zip code): proportion in each group (urban, large rural city/town, small and isolated small rural town)
10) Quadratic term of baseline outcome

Covariates will be centered and standardized to have mean 0 and standard deviation (SD) of 1 so that they have the same scale.[20] We note that many of the above measures are expected to be highly correlated; however, the proposed lasso approach is able to select from among correlated measures, based on the predictive ability.

5.2.2 Modified Analyses Accounting for Success of Implementation

Our Objective 1 analysis considers days of OUD treatment over the entire follow-up period since randomization. However, it took time for HCSs to hire the NCM, and for the NCM to start seeing patients once hired, and it is possible some may have never succeeded in implementing the model. We therefore plan to conduct two modified analyses, which will apply the same statistical model as the primary analysis but will either modify the definition of the follow-up period over which the Objective 1 outcome measure will be calculated or will restrict which HCSs are included.

5.2.2.a. Analysis During the Period in Which the NCM was Seeing Patients

First, we will modify the time period over which the main outcome is calculated to only include periods after which the NCM has engaged a patient in OUD treatment in the intervention clinic. For example, if the intervention clinic of a HCS hired the NCM 16 weeks after randomization and s/he was trained 8 weeks later and s/he engaged the first patient 1 week after training, for that HCS we would define the time period from 23 weeks post randomization until the end of the follow-up period, for both the clinic randomized to the PROUD intervention and the clinic randomized to UPC.

5.2.2.b. Analysis Limited to Clinics that Successfully Implemented the MA Model

Second, we will further restrict the set of clinics analyzed by excluding HCS from the analysis if the clinic randomized to the intervention in that HCS did not successfully implement the MA model. Successful implementation is defined as the NCM at the intervention clinic at that HCS seeing at least 30 patients. The choice of 30 patients was selected *a posteriori* as a level that several HCS had achieved and which was hypothesized as a tipping point for the clinic to be known as providing OUD treatment within the HCS. If more than 2 HCS are excluded, then we may not be able to fit the mixed-effect model; if that is the case then we will instead describe the outcome across intervention arms in these sets of clinics.

5.2.3 Sensitivity Analysis Addressing the Algorithm for Assigning Patients to Clinics

The primary algorithm for clinic assignment (see Section 3) prioritizes the clinic that the patient visited pre-randomization. If a patient visits the UPC clinic pre-randomization but then starts going to the PROUD clinic to receive care from the NCM, under this approach for clinic assignment any medication days of treatment will be “counted” toward the UPC clinic, which could bias the effect

of PROUD toward the null. To address this issue, we plan to describe the number of patients with OUD (diagnosed any time during the study period) treated in the intervention clinic during follow-up who were assigned to the UPC clinic (see Section 3 above). If we observe that *ever* occurs at a health system, we will apply an alternate algorithm as a sensitivity analysis, in which we will assign patients to clinics based on the number of visits to the clinic during the post-randomization period.

5.2.4 Sensitivity Analyses Focused on Assumptions Made in Estimating Patient-Years of OUD Medication Treatment (Objective 1: Main Implementation Outcome)

While medication dispensings are only a proxy for ingestion of medications, they are the gold standard for ascertaining medication use with EHR and claims data. When evaluating patient days of OUD treatment, only medication orders are available at most PROUD health systems. Pharmacy dispensings are complete at only one health system. Medication orders suffer from the same limitation of no assurance the medication was ingested plus medication orders could have been written and never dispensed. We expect that this is uncommon as preliminary data from Phase I at the health system with complete dispensings indicated 98% of patients with a buprenorphine order have a dispensing within 60 days of the order). However, medication dispensing data includes dispensed refills, but orders only include an indication that there can be a refill. Preliminary data from Phase I at this health system indicate approximately 14% of buprenorphine orders contain at least 1 refill on the order.

To address limitations of medication orders, we plan to conduct sensitivity analyses where we replicate the objective 1 main outcome analysis using different assumptions for calculating days of OUD medication treatment to see if there are changes in results and conclusion. Specifically, the sensitivity analyses below include more restrictive objective 1 outcome analyses with fewer assumptions about medication use and refills. In addition, we propose sensitivity analyses to address assumptions made in smoothing gaps between medication orders and/or procedures, a sensitivity analysis to add methadone maintenance therapy from the 1-2 health systems that have such data, and an analysis using dispensing data where available for at least some patients in both trial clinics within a health system.

5.2.4.a. Omitting Last Order in an Oral Buprenorphine Treatment Episode

To address the potential limitations of medication orders, we will conduct sensitivity analyses with the objective 1 main outcome restricted to episodes of treatment with at least two oral buprenorphine orders if the patient has only oral buprenorphine orders (majority of patients), with the last order in the episode omitted in case it was not picked up and taken by the patient. This sensitivity analysis also addresses any short-term buprenorphine therapy to assist with opioid tapers. This does not impact oral buprenorphine captured by procedure codes when given in the office or any injectable buprenorphine, buprenorphine implants, or injectable XR naltrexone. We will also describe the number of treatment days that are excluded in this restrictive analysis by intervention arm and health system. When the last order is omitted, all refills after the last order are also omitted.

5.2.4.b. Vary the Allowable 7-day Gap to Define Continuous Use Episodes

To address the limitation that we smooth small gaps of ≤ 7 day between medication orders and/or procedures to arrive at continuous episodes of OUD medication use, we will vary the allowable ≤ 7 day gap to smaller and larger values (e.g., 2 days, 5 days, 10 days based on descriptive data)

when estimating the objective 1 main outcome in this sensitivity analysis. For example, the main analysis treats a 10 day order for buprenorphine on 1/1/2019 and a 10 day order for buprenorphine on 1/15/2019 as one continuous episode lasting from 1/1/2019 to 1/24/2019 (24 days) because the gap between when the first order ran out and the second order began is < 7 days. Altering the allowable gap to only 2 days would result in two different episodes (1/1/2019 – 1/10/2019 and 1/15/2019-1/24/2019) that contribute 20 days of treatment.

5.2.4.c. Incorporating Dispensings Data to Define Days of OUD Treatment

For the health systems with outpatient pharmacies in both intervention and usual care clinics (including one health system that has complete dispensing data), we will estimate days of OUD treatment in this sensitivity analysis using a combination of medication orders and dispensing data. For each dispensing, if its dispense date (rxdate) is in "the range" of an order date or a refill date, then it is considered to be linked to that order or refill and dropped in calculations; otherwise, this dispensing is considered as not linked to any order and added to the calculation of days covered. The range is defined as dispensed within 30 days after the order/refill's start date (an order's start date is the order's date; a refill's start date is derived from the original order's date and days supply).

To characterize the potential for incomplete ascertainment of OUD medication treatment, we will describe 1) what proportion of patients have a buprenorphine order but not a dispensing for buprenorphine within 30 days of the order and 2) what proportion have a dispensing for buprenorphine but do not have a buprenorphine order 30 days prior to the dispensing. We will vary the 30-day window between order and dispensing (e.g., 60 days), and report by period (e.g., baseline and post-randomization) and by trial arm.

5.2.4.d. Incorporating Limited Data on Methadone Maintenance Therapy (MMT)

We will describe the availability of MMT by health system. For health systems with methadone maintenance outpatient treatment programs (OTPs) data available (one HCS and possibly a second), we will incorporate procedure codes for Methadone Maintenance OTPs when estimating the objective 1 main implementation outcome in this analysis. Based on consultation with addiction medicine specialists, we will require at least 2 procedure codes to be included as using Methadone Maintenance from an OTP and assume that treatment lasts for the time period between codes for a maximum of 365 days per code (e.g., 2 codes 365 days apart). The end date of MMT (i.e., last claim) will estimated at half the average days between previous MMT codes.

5.2.4.e. Combined Sensitivity Analysis Using All Optimal Data

We will conduct a sensitivity analysis of the objective 1 main implementation outcome using both MMT data (1-2 health systems) and dispensing data as they are detailed above. This will be done across the 1-2 health systems that have both MMT and dispensing data.

5.2.5 Timing of OUD Diagnosis

Our primary analysis includes all days of OUD treatment for buprenorphine and injectable naltrexone, including among patients with and without an OUD diagnosis prior to randomization, and among patients who were seen in the clinic previously as well as patients who were new to the clinic or to the health system entirely. If we find that the PROUD intervention increases the provision of OUD treatment based on our primary analysis, we will further explore which of the following mechanisms may have contributed to this increase: (1) by increasing the number of days treated among individuals in the clinic pre-randomization who also had a documented OUD

diagnosis pre-randomization, (2) by increasing the number of days treated among individuals in the clinic pre-randomization but who did not have a documented OUD diagnosis pre-randomization, or (3) by attracting patients post-randomization not previously seen in the clinic who have OUD (from within or outside the HCS; Supplemental Figure Shell 1, Objective 2, Section 10.2). To address each of these possibilities, we plan to conduct secondary analyses of the Objective 1 outcome among each of these 3 subsets overall by arm (as well as conducting descriptive analyses by health system). To facilitate comparison of days treated across these different study populations, we will apply the same scaling factor as in the definition of the primary analysis of the outcome (i.e., the number of patient-days of OUD treatment will be divided by the total number of patients seen in the clinic over the follow-up period).

5.2.6 Secondary Analyses Among Patients Treated for OUD

Our primary outcome is a scaled clinic-level measure of OUD treatment among all patients assigned to the clinic, scaled by the number of patients seen in the clinic, over the 2-year post-randomization period. In secondary analyses, we plan to repeat the primary analysis where we calculate the outcome measure among patients with documented OUD treatment during the 2 years post-randomization (and scale by the number of patients with OUD; see Table Shell 6, Objective 1, Section 10.1). However, we note that because the intervention could impact both the denominator (number of patients diagnosed with OUD) as well as the numerator (initiation and duration of treatment among treated patients), interpretation of these secondary analyses should be interpreted cautiously.

5.3 Primary Analytic Method for Objective 2 Primary Effectiveness Outcome Measure

Our primary outcome for the main Objective 2 (effectiveness) outcome is a patient-level measure of the number of days of acute care utilization over the follow-up period, which is a 2-year period for 5 of the 6 HCSs, and a 1.5-year period for the 6th HCS.

The primary analysis will be among individuals with an eligible PC visit pre-randomization who had an OUD diagnosis during the pre-randomization period (see definition of “Objective 2 primary study sample” in Section 3). The patient-level analysis will follow ITT principles, with patients analyzed according to the randomization group of the clinic to which they were assigned pre-randomization, regardless of the degree to which the clinic actually implemented the intervention, and regardless of whether the patient was actually treated by the NCM or seen post randomization.

We hypothesize that, among patients who had an eligible PC visit and were identified as having an OUD diagnosis pre-randomization (documented in their EHRs up to 3 years prior to randomization), individuals from a PROUD intervention clinic will have decreased acute care utilization after randomization as compared to individuals from a UPC clinic. We plan to fit a mixed-effect Poisson regression model (with log link) at the patient level to the number of days of acute care utilization. The model will account for clustering of patients within a clinic by including clinic-specific random intercepts. Specifically, the regression model will be of the following form:

$$log\left[E\left(y_{ijk}\right)\right] = \log\left(\mathrm{days_j}\right) + \alpha + \beta * trt_{ij} + \gamma * z_{ijk} + \theta_{ij}$$

where

- y_{ijk} is the observed number of days of acute care utilization of patient k in clinic i of HCS j over the follow-up period

- trt_{ij} is the treatment indicator (PROUD intervention) for clinic i in HCS j
- z_{ijk} is the baseline value of the outcome during the two years prior to randomization for patient k in clinic i of HCS j (γ is the corresponding coefficient)
- θ_{ij} is the random effect for clinic i in HCS j

Because one of the HCSs was randomized at a later date than the others, not all HCSs have the same amount of follow-up time. To account for this difference, we will include in the model an offset term for the number of days of potential follow-up time (e.g., 2 years for 5 of the HCSs and 1.5 years for the 6th HCS); in the above model, $\log(\mathrm{days_j})$ denotes the offset term where $\mathrm{days_j}$ denotes the days of follow up in HCS j.

We will evaluate our primary Objective 2 hypothesis by testing the null hypothesis $H_0\colon \beta = 0$ versus the two-sided alternative hypothesis that β is non-zero with a type 1 error rate of 0.05. In cluster-randomized trials with a small number of clusters, a small-sample correction is often necessary to obtain correct type 1 error rates.[21,22] Although small-sample degree of freedom (DF) correction methods have been evaluated for continuous and binary outcomes under generalized linear mixed-effect models (GLMMs), there has not been prior guidance of which approach to use in our setting with a count outcome and covariate adjustment. Given this lack of knowledge, members of the PROUD statistical team are conducting simulations to evaluate alternate small-sample DF methods in this setting. Our results so far suggest that the optimal choice of testing procedure varies depending on the data-generating scenario in terms of the intraclass correlation coefficient (ICC), number of clusters, and sample size within a cluster; however, using the likelihood ratio test with the Between-Within (BW) DF correction method (also referred to as "inner-outer" approach) appears to perform generally well in the scenario that aligned most closely with PROUD Phase 1 data used in the power simulation (see Appendix C).

Inclusion of a HCS-specific random effect. The above model accounts for clustering of patients within clinics but does not account for the possibility of additional correlation of outcomes from patients within the same HCS beyond any within-clinic correlation. The reason for this is because analyses of Phase 1 data suggested that within-clinic correlation was considerably larger than within-HCS correlation (the random-effect variance was 0.00016 for HCS versus 0.055 for clinic from a model including random effects for both). Although including a random-effect term that is not truly needed should not impact parameter estimates, including a HCS-level random effect leads to inferential challenges. In particular, as above there has not been guidance on applying small-sample DF correction methods when there are random-effects at two levels (here 12 clinics nested within 6 HCSs). We therefore chose the clustering level that explained most of the correlation for primary Objective 2 analyses. As a sensitivity analysis, we plan to examine the impact of additionally including a HCS-level random effect on the treatment effect parameter and standard error estimate.

5.4 Secondary Analyses of Objective 2 Primary Effectiveness Outcome Measure within the Primary Objective 2 Sample

5.4.1 Objective 2 Covariate-adjusted Model

We will apply a sensitivity analysis that includes additional covariate adjustment beyond the number of days of acute care at baseline. We plan to additionally adjust for the following pre-specified covariates, identified based on known and hypothesized predictors of acute care utilization among patients with OUDs identified from the literature and from discussions with study co-investigators:

1) age (at randomization, including linear and quadratic terms)
2) gender (F/M)
3) race/ethnicity (Hispanic, Asian, Black or African American, White, Other, Unknown)
4) Neighborhood-level measures capturing socioeconomic status (obtained from census data linked via zip code [using most recent zip code available pre-randomization]):
    a. median Neighborhood household income
    b. percent Neighborhood below the federal poverty line
    c. percent Neighborhood unemployed
5) Insurance status (binary indicators for the patient's type of insurance [Medicaid, Medicare, other insurance, uninsured], using the most recent available known value pre-randomization)[23]
6) Days of OUD medication treatment (in the two years pre-randomization)[24]
7) Number of days with OUD documented
8) Comorbidity (yes/no, in the two years pre-randomization)
    a. Alcohol use disorders[25]
    b. Other (non-opioid) substance use disorder
    c. Schizophrenia and other psychotic disorders
    d. Weighted summary score of other comorbidities (Elixhauser index[26], pulling out a-c above).[25]
9. Housing instability, including homelessness (indicator for having a V or Z code in the two years pre-randomization)[23]

Note that some of these covariates (e.g., race/ethnicity; insurance status) have some missing values; the approach to handling missing data in adjustment variables is described below in Section 8.1.

5.4.2 Objective 2 Sensitivity Analysis Among "Active" Patients

Because we have a visit-based sample, we do not know when a patient is no longer observable (e.g., switched clinics, moved, went elsewhere for medical or OUD treatment, or died), except among enrolled patients in HCSs with insurance claims (discussed in the following section). In particular, we cannot distinguish between a patient who has no acute care utilization over the follow-up period and a patient who has acute care utilization outside of the health system (that is not captured across all HCSs in our data). To address this, we plan to repeat the primary analysis among the subset of patients who are "active in the HCS", defined as having any evidence that they are still observable (e.g., any visit (PC and non-PC), or diagnosis anywhere in the health system during follow-up). In addition, we plan to conduct a second sensitivity analysis among patients "active in PC", defined as having a PC visit to their "assigned" trial clinic during the follow-up period.

5.4.3 Objective 2 Sensitivity Analysis Among Patients Enrolled in the HCS Health Plan

Two of the HCSs are integrated health systems that insure a subset of the patients who receive clinical care in the health system. We will examine the proportion of patients with an OUD diagnosis at baseline who are in the insured sample and who were enrolled in the health plan at the time of randomization. Descriptively, we will compare the estimated outcome rate separately within each of these two HCS in a model that accounts for enrollment as compared to the main analysis approach that does not account for enrollment (e.g., by comparing the mean number of

days of acute care utilization per year enrolled vs. the mean number of days of acute care utilization per year of the 2-year HCS follow-up period).

In addition, depending on the size of the subsample, we will consider repeating the primary Obj. 2 analysis among this subsample. The offset term will be slightly modified from the primary analysis: rather than use the amount of possible follow-up from the randomization date, we will use more precise information on the number of days the patient was continuously enrolled in the health plan post-randomization during the available follow-up period (allowing gaps of up to 90 days). Although this proposed analysis is only available on a subset of the Objective 2 primary sample in the 2 HCSs, by restricting to enrolled patients and accounting for follow-up time in which the patient was enrolled (such that their outcome data is known to be observable), analyses within this sample could mitigate issues whereby outcomes of patients included in the primary Objective 2 visit-based sample may not be observable (e.g., if they left the health system or accessed acute care outside the system). Understanding differences in this analysis and HCS-level estimates for these sites will aid in interpretation of findings.

5.4.4 Objective 2 Sensitivity Analysis Addressing Clinic Assignment/crossover

We will follow a similar approach as the above Objective 1 sensitivity analysis (see Section 5.2.3 above). Specifically, if we observe that any patients assigned to a UPC clinic is ever treated in the intervention clinic at that HCS during the follow-up period, we will apply an alternate algorithm in which patients are assigned to the clinic where they have the most PC visits post-randomization.

5.4.5 Objective 2 Secondary Modified Analyses Accounting for Success of Implementation

We will conduct secondary modified analyses accounting for success of implementation following a similar approach as for Objective 1 (see Section 5.2.2 “Modified analyses accounting for success of implementation”), including (1) analyses restricted to the follow-up period in which the NCM was seeing patients (for which we will modify the offset term), and (2) analyses limited to HCS in which the clinic randomly assigned to the PROUD intervention successfully implemented the MA model. We also plan to repeat analysis (1) restricted to patients with an active PC visit post-randomization (Section 5.4.2).

5.4.6 Objective 2 Sensitivity Analysis Applying Stricter Eligibility Criteria

Our primary analysis requires just a single OUD diagnosis pre-randomization for a patient to be included in the sample, as well as just a PC visit at any time pre-randomization (up to 3 years). To account for the possibility that having only one diagnosis could reflect an error in identifying OUD and that patients with a PC visit early in the pre-randomization period may have left the clinic, we will conduct a sensitivity analysis among the sub-sample of our primary sample that restricts to patients with both ≥2 days with an OUD diagnosis during the pre-randomization period and a PC visit in the year prior to randomization.

5.5 Secondary Analyses of Primary Objective 2 Outcome Measure within the Objective 2 Secondary Sample

Here we describe secondary analyses of the primary Objective 2 outcome measure within the larger secondary sample (see Section 3.3) that includes patients in the primary Objective 2 sample (i.e., the pre-randomization sample with a PC visit and OUD diagnosis pre-

randomization), as well as the post-randomization sample of patients with newly recognized OUD in the trial clinics post-randomization.

5.5.1 Rationale for Conducting Analyses Within a Secondary Sample

It is expected that some of the patients who initiate treatment in PROUD intervention clinics will have been diagnosed with OUDs after randomization, potentially due to the PROUD intervention. It is also possible that patients may be newly attracted to the clinic (or to the health system entirely) potentially because of the PROUD intervention (based on the fact that at least 77% of patients treated in MA were new to the clinic after implementation).[27] The primary analysis of Objective 2 described above would miss any impact of the PROUD intervention on both of these subsamples of patients, because they were not identified pre-randomization (because they were not previously diagnosed with OUDs or because they did not visit the clinic in the pre-randomization period). These secondary analyses within the secondary Objective 2 study sample, which includes patients seen in the trial clinics with an OUD diagnosis post-randomization only, are designed to capture these additional patients who may be affected by the PROUD intervention.

On the other hand, these secondary analyses must account for the fact that patients with newly recognized OUD in the PROUD intervention clinics post-randomization are likely to differ markedly from patients with newly recognized OUD in the UPC clinics post-randomization. Further, it is likely that these patients could differ in ways that may be associated with acute care utilization (e.g., patients could be referred for ongoing buprenorphine treatment from an ED or hospital that started treatment). To address this, analyses will adjust for covariates known or hypothesized to be associated with acute care utilization (based on prior literature), as well as secondarily for any additional covariate observed to differ across patients with newly recognized OUD post-randomization in the UPC versus PROUD intervention clinics.

5.5.2 Defining Covariates for the Objective 2 Secondary Sample That Includes Patients Not Seen in the Health System Pre-randomization

Defining covariates using pre-randomization data is preferred for randomized studies, because this ensures that the intervention does not affect the covariate values. However, this is not possible for patients who were not seen in the HCS pre-randomization. For these patients we therefore plan to use the post-randomization value of time-varying covariates that is available closest to randomization. We note that using this approach means that analyses including patients new to the HCS post-randomization cannot adjust for "baseline" acute care utilization (since patients new to the HCS post-randomization do not have baseline data observed). We considered applying a multiple imputation approach to address missing baseline data among these patients; however, we did not think the missing at random (MAR) assumption was reasonable (i.e., we did not think using data from patients in the HCS pre-randomization was sufficient to impute data from patients new to the HCS). Therefore, sensitivity analyses will be applied to examine the subpopulation excluding these patients (see Section 5.5.4).

Decisions regarding the set of covariates to be included in analyses within this secondary sample (listed below) were made in conjunction with the Investigator team to determine whether individual covariates measured post-randomization are likely to be impacted by the intervention. We wanted to avoid including covariates that are likely to be impacted by the intervention (as they may be on the causal pathway). Although documentation of certain comorbidities were thought to be most likely to be impacted by the intervention, it was hypothesized that documentation of any comorbidities could be increased via the intervention due to increased care in general; insurance

status was also plausibly thought to be related to intervention status (as poor OUD-related outcomes could affect insurance status).

Covariate adjustment: We plan to adjust for the following subset of covariates as in the secondary analysis within the primary Objective 2 sample (described above in Section 5.4.1):

1) age (at randomization, including linear and quadratic terms)
2) gender (F/M)
3) race/ethnicity (Hispanic, Asian, Black or African American, White, Other, Unknown)
4) Neighborhood-level measures capturing socioeconomic status (obtained from census data linked via zip code [using zip code available closest to randomization, prioritizing pre-randomization if available]):
    a. median Neighborhood household income
    b. percent Neighborhood below the federal poverty line
    c. percent Neighborhood unemployed

In a sensitivity analysis, we will further adjust for additional covariates (including insurance status, comorbidities, and housing instability measures from Section 5.4.1) found to differ between individuals with newly recognized OUD post-randomization in the PROUD intervention clinics as compared to individuals with newly recognized OUD post-randomization in the UPC clinics (e.g., with a standardized mean difference of <0.10). However, because differences in these covariates (e.g., comorbidities) could be affected by the intervention, results from this sensitivity analysis will be interpreted with caution.

5.5.3 Primary Objective 2 Outcome Analysis within the Secondary Sample

We plan to fit a similar mixed-effect Poisson regression model as in the primary Objective 2 analysis but that (1) includes additional covariates and (2) that allows the treatment effect comparing the PROUD intervention clinics to UPC clinics to differ among patients with newly recognized OUD in the clinics post-randomization vs. those with visits to the trial clinics and OUD documented pre-randomization. Specifically, the model will be of the following form:

$$log\left[E\left(y_{ijk}\right)\right] = \log\left(\mathrm{days_j}\right) + \alpha_0 + \alpha_1 \mathrm{period}_{ijk} + \left(\beta_0 + \beta_1 * \mathrm{period}_{ijk}\right) * trt_{ij} + \gamma * z_{ijk} + \theta_{ij}$$

where *period* is an indicator for the period when the patient was first recognized with OUD in the trial clinics (i.e., whether the patient is in the post-randomization sample or pre-randomization sample; see Section 3.3), and the other terms are defined as in the primary analysis, except for the covariate vector (*z*), which includes the set of covariates listed above in Section 5.5.2.

We will evaluate our secondary Objective 2 hypothesis by testing the composite null hypothesis $H_0: \beta_0 = \beta_1 = 0$ versus the alternative hypothesis that at least one of β_0 or β_1 is non-zero by conducting a likelihood ratio test. Additionally, we will estimate the treatment effect separately among patients identified in the pre-randomization period (β_0) and patients identified in the post-randomization period ($\beta_0 + \beta_1$). The coefficient β_1 is the difference in the treatment effect comparing patients identified post-randomization to those identified in the pre-randomization period. This could either reflect a true difference in the treatment effect, or, more likely, it could reflect unmeasured confounders (not included in *z*) that differ between patients newly recognized with OUD post-randomization in the intervention versus UPC trial clinics. We do not have a specific hypothesis regarding β_1 because new patients may be attracted to the clinic to receive the PROUD intervention, as seen in Labelle,[1,27] and these patients may be sicker (or healthier) than patients identified pre-randomization, or more motivated for treatment, which could increase

(or decrease) acute care utilization during the period after randomization. We are not powered to test for the difference in intervention effects between these two groups (β_1); rather, the analyses proposed here are exploratory analyses that will generate hypotheses for testing in future studies.

5.5.4 Sensitivity Analyses of Primary Objective 2 Outcome Measure within the Objective 2 Secondary Sample

5.5.4.a. Restricting to Patients in the HCS Pre-randomization

As discussed in Section 5.5.2, analyses in the full secondary sample that includes patients new to the HCS post-randomization are challenging due to the inability to adjust for baseline characteristics (including the baseline value of the outcome). To address this limitation, we plan to repeat the Objective 2 analysis in the secondary sample restricted to patients in the HCS pre-randomization (see Supplemental Figure Shell 1, Objective 2, Section 10.2) and using the full set of covariates as in the secondary analysis within the Objective 2 primary sample (described above in Section 5.4.1). Because differences in estimates in this sample could be due either to using a fuller set of covariate adjustment or using a different sample, we will additionally conduct this analysis using the same set of covariates as in Section 5.5.2 (age, gender, race/ethnicity, and neighborhood-level SES).

5.5.4.b. Secondary Analyses Accounting for Implementation and Other Sensitivity Analyses

We also plan to apply the same set of sensitivity analyses in the Objective 2 secondary sample as in the Objective 2 primary sample, including among “active” patients (in the HCS, in PC; Section 5.4.2), among enrolled patients (in 2 HCSs; Section 5.4.3), under the alternate clinic assignment algorithm (Section 5.4.4), under modified analyses accounting for success of implementation (Section 5.4.5), and among patients with ≥2 OUD diagnoses (Section 5.4.6) over the full study period.

5.6 Analyses of Secondary Outcome Measures

Analyses of pre-specified secondary clinic-level outcomes (Table 2, Section 4.3) will use the same analytic approach as for the primary Objective 1 outcome, and analyses of pre-specified secondary patient-level outcomes will use the same analytic approach as for the primary Objective 2 outcome, using the appropriate link function (e.g. logit for binary measures). In addition, patient-level versions of the clinic-level implementation outcomes (e.g., number of days of OUD medication treatment) will be analyzed at a patient level among the Objective 2 sample (Objective 2 sample, see Shell Table 2; Section 10.2).

Note that unless otherwise specified, analyses will be adjusted for the baseline measure of the respective outcome being considered; exceptions are that binary or categorical outcomes derived from continuous measures will adjust for the continuous version of the measure (e.g., the binary variable of “Any acute care”) and that the patient-level binary versions of the initiation and retention measures described in in Table 2 (Section 4.3) will adjust for the baseline number of days of OUD treatment.

5.7 Effect Modification and Subgroup Analyses

5.7.1 Objective 1 Primary Implementation Outcome

Given the NIH requirement to perform subgroup analyses of the primary (Objective 1) outcome on the basis of sex, race and ethnicity, and the importance of understanding how the MA Model performs in individuals < 26 years, we plan to conduct analyses of subgroups based on: age (<

26 vs older), where age is calculated at the eligible visit closest to randomization, prioritizing any pre-randomization visits; sex; and race/ethnicity (Hispanic, Non-Hispanic White, Non-Hispanic Black, Non-Hispanic Asian, Other). We note that race and ethnicity are combined into a single variable, because in some HCS race is not documented if the patient reports ethnicity as Hispanic. An interaction term between demographic subgroup and treatment assignment can be used to evaluate whether the demographic factor moderates the implementation effect. Any such comparisons will likely be underpowered and must be interpreted with caution. The original Massachusetts studies observed that patients who were male or Black or Hispanic were less likely to engage in PC treatment of OUD with the MA Model, compared to female and white patients, respectively,[1,15] but no differences were observed across age groups. As a result, PROUD investigators hypothesize that the intervention will result in smaller increases in OUD medication treatment in patients who are male or non-Hispanic Black or Hispanic but hypothesize no differences across age groups.

For each subgroup of interest, we will define the Objective 1 outcome measure among individuals within that subgroup as the number of patient-days of OUD treatment among individuals in that subgroup who were seen in the clinic during the 2 years after randomization, scaled by the total number of patients seen in the clinic (within that subgroup) during that time period. We denote the outcome for clinic i at HCS j among subgroup g as y_{ijg}.

As a concrete example, here we write down the model for the effect modification analysis for age group. Let g be an indicator variable that takes the value 1 if a person is < 26 and takes the value 0 if a person is 26 years or older. We plan to adapt the model for the primary analysis as follows:

$$y_{ijg} = \alpha_0 + \alpha_1 g + \beta_0 trt_{ij} + \beta_1 trt_{ij} g + \gamma * z_{ijg} + \theta_j + \epsilon_{ijg}$$

where

- y_{ijg} is the age-group specific Objective 1 outcome measure for clinic i at HCS j (defined above),
- trt_{ij} is the treatment indicator (PROUD intervention) for clinic i at HCS j,
- z_{ijg} is the age-group specific Objective 1 outcome measure for the two years prior to randomization (hereafter baseline) for clinic i at HCS j,
- θ_j is the random effect for HCS j and is distributed $N(0, \sigma_\theta^2)$, and
- ϵ_{ijg} is the error term for clinic i at HCS j and is distributed $N(0, \sigma_\epsilon^2)$.

We will test whether the effect of the PROUD intervention differs across age groups by testing whether the term β_1 differs from zero using an F test. We will also estimate age-group specific intervention effects (e.g., β_0 for individuals of age 26 or older and $\beta_0 + \beta_1$ for individuals < 26). Subgroup-specific intervention effects will be tested by using a one-tailed test, consistent with the primary (i.e., un-stratified) analysis of the Objective 1 outcome. The same modeling approach will be applied for analyses of gender. If race/ethnicity is a categorical variable with more than 2 categories, we will evaluate whether the intervention effect differs across any of the racial/ethnic groups (i.e., if effect modification is present) with an omnibus (overall) test. In addition, per our hypotheses above, we will test for a difference in outcomes between non-Hispanic Black patients and Hispanic patients, each as compared to white patients. Again, as discussed above such comparisons will likely be underpowered and should be interpreted with caution.

5.7.2 Objective 2 Primary Effectiveness Outcome

We plan to conduct analyses of subgroups based on: age (< 26 vs older) at randomization; sex; race and ethnicity (Hispanic, Non-Hispanic White, Non-Hispanic Black, Non-Hispanic Asian, Other), using similar subgroups as Objective 1. If one of the subgroups is small (fewer than 5% of patients in the sample), we will consider excluding that category or combining with the "Other" category. To examine whether the treatment effect may differ across groups, we plan to apply the same analytic approach as for the primary analysis of the Objective 2 outcome, in which we will additionally include an interaction term between the categorical subgroup variable of interest and the treatment effect term. As above, such comparisons are expected to be underpowered and will be interpreted cautiously. To aid in the interpretation of potential subgroup-specific effects, we plan to additionally conduct analyses of these subgroups for the patient-level treatment outcome of number of days of OUD medication treatment.

6.0 POWER CONSIDERATIONS

6.1 Power Simulations for Primary Objective 1 Outcome

The power calculations focus on the six HCS determined to be eligible during Phase 1 of CTN-0074, with each HCS contributing two clinics. One clinic from each HCS will be randomized to implement the PROUD intervention, while the other will continue with usual primary care (UPC). Simulations were conducted to calculate the power associated with various values of the treatment effect, which is parameterized as the mean of the primary outcome measure in the intervention clinics divided by the mean in the UPC clinics. Of ultimate interest in the calculations presented here is whether, with the health systems selected in Phase 1, there will be sufficient power (>80%) to detect at least a 5-fold increase in the number of patient days of OUD treatment (per 10,000 participants) associated with implementation of the PROUD intervention as compared to UPC. To accomplish this, we considered various values of the treatment effect and calculated the corresponding statistical power via simulation.

6.1.1 Data-generation and Analysis Model

In our power simulation, there were 6 HCSs and 2 clinics within each HCS (one clinic assigned to the intervention and one to UPC), making 12 clinics in all.

The outcome variable for each clinic is *Treated Days per Patient Seen,* which with actual data, we would calculate by dividing the total number of treated days at the clinic during 2 years of follow-up by the total number of unique patients seen by the clinic during that same time period. To evaluate power under an analysis approach that adjusts for the baseline value of the outcome, we used Phase 1 data to calculate an approximation of the magnitude of the association between the number of OUD-treated days per patient seen pre- and post-randomization. Using the analytic model specified in Equation (1) would require four years of data for this estimation, however only three years of data are available from Phase 1 (FYs 2014, 2015 and 2016). Thus, data were simulated in a recursive fashion using an autoregressive lag-1 approach.

Step 1: Estimate via regression the relationship between the number of OUD-treated days per patient seen in the last two years of Phase 1 data (2015-2016) and the same measure for the first two years of the Phase 1 data (2014-2015). Letting *j* index HCS and *i* index clinics within an HCS, this corresponds to regression of the outcome for 2015-2016 on 2014-2015 (see Section 6.1.2). That is, model

$$y_{ij\langle 2015-2016\rangle} \sim \mu + \rho\, y_{ij\langle 2014-2015\rangle}. \qquad (2)$$

For this model, a random effect capturing the correlation of clinics from the same health care system was *not* included (see Section 6.1.2). The parameter ρ captures the association between the number of treated days per patient seen across consecutive years, and the parameter μ captures the mean number of treated days per patient seen if no individuals were treated for OUD in the previous year. Let $\hat{\mu}$ and $\hat{\rho}$ denote the estimates from the above model.

Step 2: Using this estimated regression model ($\hat{\mu}, \hat{\rho}$), simulate the outcome measure for the next two-year period (2016-2017) for the UPC clinics (i.e., i=1) from

$$\hat{y}_{1j\langle 2016-2017\rangle} = \varphi + \omega\, \tilde{y}_{1j\langle 2015-2016\rangle} + \varepsilon_{1j}$$

where ε_{1j} are normally distributed with mean zero and variance σ_{ε}^2. The parameters $\varphi$and ω are analogous to μ and ρ from Equation (2). Note that the $\tilde{y}_{1j\langle 2015-2016\rangle}$ are generated from a normal distribution with

$$E(\tilde{y}_{1j\langle 2015-2016\rangle}) = {}^{\varphi}\!/_{(1-\omega)}$$

$$Var(\tilde{y}_{1j\langle 2015-2016\rangle}) = {}^{\sigma_\epsilon^2}\!/_{(1-\omega^2)}$$

See Appendix B for the justification of these initial values.

Step 3: Repeat the simulation for the UPC clinics in the next overlapping two-year period (i.e., 2017-2018) from

$$\hat{y}_{1j\langle 2017-2018\rangle} = \varphi + \omega\, \tilde{y}_{1j\langle 2016-2017\rangle} + \varepsilon_{1j}.$$

Step 4: Then repeat the simulation for the UPC clinics a final time for the post-randomization period corresponding roughly to 2018-2019 from

$$\hat{y}_{1j\langle 2018-2019\rangle} = \varphi + \omega\, \tilde{y}_{1j\langle 2017-2018\rangle} + \varepsilon_{1j}.$$

Then simulate the 2018-2019 outcome data for the MA Model clinics (i.e., *i*=2) from

$$\tilde{y}_{2j\langle 2018-2019\rangle} = \tau + \tilde{y}_{1j\langle 2018-2019\rangle} + \pi\, \vartheta_j$$

where j are normally distributed with mean zero and variance σ_{ϑ}^2. Note that $\vartheta_j$induces a correlation between the two clinics from HCS *j*.

6.1.2 Parameters Used to Generate the Simulated Data

Table 3 provides the number of OUD-treated days during a two-year period per patient seen in the clinic. These values were used to estimate the parameters for Steps 1 and 2 in the algorithm summarized in Section 6.1.1.

Table 3: Number of Days Treated for OUDs (with buprenorphine or injectable naltrexone) per Patient Seen at 2 Phase 1 Clinics in Each of 6 Health Care Systems (HCS)

HCS	Fiscal Years 2014-2015		Fiscal Years 2015-2016	
	Clinic 1	Clinic 2	Clinic 1	Clinic 2
1	0.031	0.008	0.085	0.019
2	0.339	0.215	0.354	0.173
3	0.001	0.022	0.001	0.076
4	0.015	0.089	0.021	0.431
5	0.007	0.002	0.004	0.002
6	1.385	0.715	1.361	0.703

From Table 3 we fit a random effects model with a fixed intercept for the number of OUD-treated days per patient seen in FYs 2015-2016 as a function of FYs 2014-2015 where the random effect captures the correlation between clinics from the same HCS. The estimated intercept was 0.05, the coefficient for FYs 2014-2015 was 0.94 and the variance of the random effect was not significantly different from zero (in fact, the actual estimated variance was zero). Thus, the

predictive model used to generate the simulated data did not include a random effect for HCS (see Section 6.1.1).

The parameters used for this set of simulations are summarized in Table 4 below.

Table 4: Parameters Explored in the Power Simulation

Parameter	Value
σ_ϵ	0.10
σ_ϑ	0
ω	0.94
φ	0.05

6.1.3 Results of Simulations

Table 5 presents power results (detectable effect size) for the 0.05-level one-tailed test, based on 10,000 iterations per table cell for two models: one without adjustment for baseline, and one with baseline included as a covariate. The column "Inclusion of Baseline as a Covariate" adjusts for the pre-randomization/baseline value of the primary outcome measure.

Table 5: Power Results for a 0.05-level One-Tailed Test, Based on 10,000 Iterations Per Cell

	Model	
k-fold Increase in Primary Outcome (Treatment Effect)	No Adjustment for Baseline	Inclusion of Baseline as a Covariate
1.00	5%	5%
1.06	9%	13%
1.12	15%	29%
1.18	21%	49%
1.24	30%	68%
1.30	39%	84%
1.36	50%	93%
1.42	60%	98%

Based on Table 5, there is at least 80% power to detect a 30% increase in the number of OUD-treated days per patient seen. Thus, with two clinics in each of six HCSs, the study is sufficiently powered to detect the targeted 5-fold increase in the primary outcome measure. As anticipated, there is a substantial gain in power when the baseline value is included as a covariate in the primary outcome model.

6.1.4 Potential Exclusion of One Health Care System

At the time power calculations were originally conducted, it was thought possible that one of the HCSs might not be able to participate due to issues with ceding to the single Institutional Review Board (IRB), though this did not end up occurring (instead, this site randomized 6 months late; see Section 2.5). To address this possibility, the power calculations were repeated but using information from only the five other HCSs and the data simulated arise from only five HCSs and ten clinics.

As with the original power calculations, we fit a random effects model to the remaining health systems' data in Table 3 with a fixed intercept for the number of OUD-treated days per patient seen in FYs 2015-2016 as a function FYs 2014-2015 where the random effect captures the correlation between clinics arising from the same HCS. The parameter estimates from this model did not change substantially. The estimated intercept was 0.05, the coefficient for FYs 2014-2015 was 0.94 and the variance of the random effect was not significantly different from zero. Thus,

the predictive model used to generate the simulated data did not include a random effect for HCS. Table 6 summarizes the results of these additional simulations.

Table 6: Power Results for a 0.05-level One-Tailed Test, Based on 10,000 Iterations Per Cell with *Five* Health Care Systems

	Model	
k-fold Increase in Primary Outcome (Treatment Effect)	No Adjustment for Baseline	Inclusion of Baseline as a Covariate
1.00	5%	5%
1.03	8%	12%
1.06	12%	25%
1.09	16%	40%
1.12	22%	59%
1.18	35%	87%
1.24	49%	98%

With only five HCSs, the study will still be powered sufficiently to detect at least 80% power to detect an 18% increase in the number of OUD-treated days per patient seen. Specifically, the power to detect a 1.18-fold increase in the per patient primary outcome measure is 87%. Thus, with two clinics in each of five HCSs, the study is sufficiently powered to detect the targeted 5-fold increase in the primary outcome measure.

6.2 Power Simulations for Primary Objective 2 Outcome

6.2.1 Description of Power Simulation

We investigated the power of the primary Objective 2 analysis via Monte Carlo simulation. We assumed the following sample sizes for the number of patients with a prior OUD diagnosis over a 3-year period from the Phase 1 data, reflecting the 3-year baseline period of PROUD during which patients with an OUD diagnosis will be identified:

site_id	clin_num	Clin	nOUD
A	1	A1	9
A	2	A2	12
C	1	C1	63
C	2	C2	39
E	1	E1	58
E	2	E2	200
I	1	I1	100
I	2	I2	49
J	1	J1	27
J	2	J2	10
K	1	K1	388
K	2	K2	290

We generated individual-level outcome data within each of the 12 clinics as follows. First, we randomly assigned one of the two clinics within a HCS to receive the PROUD intervention and the other to the Usual Primary Care (UPC) group. We then generated outcome data from a

Poisson distribution with mean number of acute care days over a two-year period (time-frame of PROUD outcome ascertainment) using the following mean model,

$$\log E(y_{ijk}) = \alpha + \beta * trt_{ij} + \theta_{ij}$$

where

1) y_{ijk} is the number of days of acute care utilization of patient k in clinic i of HCS j
2) trt_{ij} is the treatment indicator (PROUD intervention versus UPC) for clinic i in HCS j
3) $\theta_{ij} \sim N(0, \tau^2)$ is the random effect for clinic i in HCS j (τ is the standard deviation of the clinic-level random effect)

For the parameter α we assumed that the baseline rate of acute care utilization over a two-year period for patients assigned to a UPC clinic was equal to the average number of acute care visits among patients with a prior OUD diagnosis obtained from Phase 1 data (=4.0 visits) multiplied by the average number of days per acute care visit. The average number of days per acute care visit was based on data from one health system on the average length of stay among all patients (since length of stay data was not available from all health systems at Phase 1), which was 2.04 days per visit. That is, we assumed $\alpha = \log(4 * 2.04) = 2.1$. We considered a range of values for the intent-to-treat relative risk parameter $RR_{ITT} = \exp(\beta)$ governing the association between being assigned to the PROUD intervention and acute care utilization over the follow-up period. Finally, we considered three different values for the standard deviation τ of the clinic-level random effect θ_{ij}. Specifically, we estimated a value for τ using Phase 1 data of $\tau = 0.23$, and also considered two values as a sensitivity analysis: one that was 50% smaller ($\tau = 0.12$) as well as one that was 50% larger ($\tau = 0.35$). We estimated power and type 1 error based on the standard Wald test, as well as the Wald F test that used a denominator degree of freedom based on the Between-Within (BW) small sample degree of freedom correction. For testing the coefficient β from the above model, the BW method uses as denominator degree of freedom (10 = 12 clinics - 2 fixed effect parameters being estimated). Results are based off of 1,000 simulation repetitions.

In addition to presenting power across values of RR_{ITT}, we also provide additional context in light of the fact that not all individuals in the intervention clinic with an EHR documented OUD diagnosis pre-randomization will visit the PROUD NCM and receive sustained treatment with buprenorphine or injectable naltrexone (hereafter "treated for OUDs"), which is hypothesized to meaningfully reduce acute care utilization.[17] Specifically, for different values of RR_{ITT}, we report the proportion of patients who would need to be treated for OUDs (denoted by p_{treat}), if the relative risk of acute care utilization comparing patients who are treated for OUDs versus patients who are not treated for OUDs ($RR_{treated}$) is 0.1 or 0.2. A value of 0.2 corresponds approximately to the observed RR of acute care visits comparing those without OUD to those with OUD; a value of 0.1 corresponds to the assumption that those who are treated for OUDs will have a 50% decrease in the average visit length compared to those with OUD who are not treated. These calculations assume that patients with OUD in a PROUD intervention clinic who are not treated for OUDs have the same rate of acute care utilization as patients with OUD in UPC clinics.

6.2.2 Results of Power Simulations

The following table shows the type 1 error rates for each of the 3 values of clinic-level random effect SD (τ) using the naïve Wald test ("Wald" below), as well as the Wald F test based on the BW degree of freedom correction (BW below):

	τ	**Wald**	**BW**
Sensitivity	0.12	0.106	0.075
**Primary**	0.23	0.104	0.070
Sensitivity	0.35	0.111	0.067

Although the type 1 error rates using the BW method (0.067-0.075) are still slightly elevated over the nominal 0.05 level, they are much closer to the correct level as compared to the standard Wald test (all > 0.1). We will continue to explore whether this can be improved further as the SAP continues to be developed.

The power (or type 1 error rates for $RR_{ITT} = 1$) across different effect sizes (parameterized by RR_{ITT}) and different values of the random-effect SD τ (0.12, 0.23, and 0.35) is given in Figure 2:

Figure 2: Objective 2 Power Across Different Effect Sizes

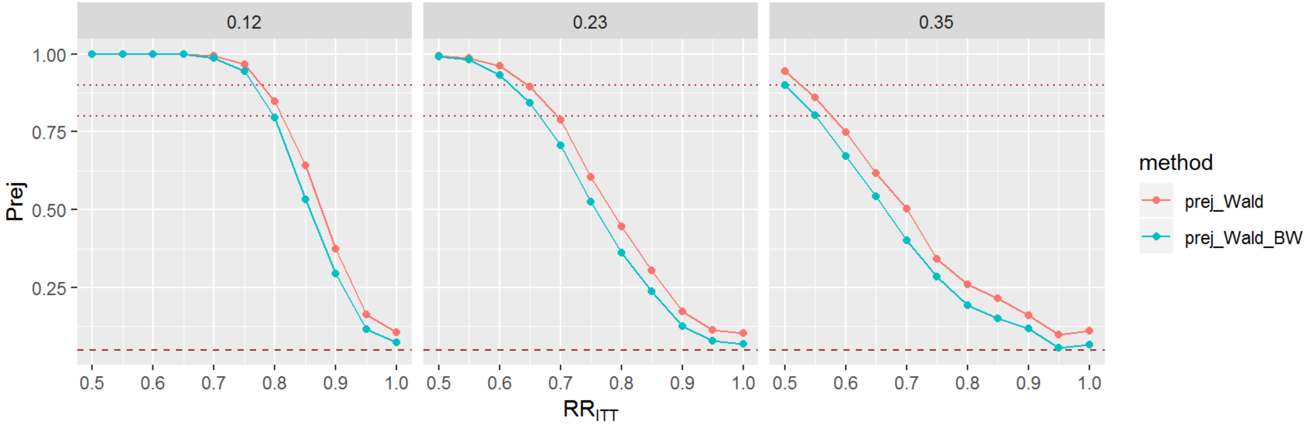

Here 'Prej' denotes the proportion of Monte Carlo iterations for which the null hypothesis was rejected; each panel corresponds to a different value of the random-effect SD (τ). Based on using the BW degree of freedom correction approach, we estimated that we have >80% power to detect values of RR_{ITT} < 0.65 when $\tau = 0.23$, corresponding to a 35% reduction in the acute care utilization rate among patients in a PROUD versus UPC clinic. Similarly, we have >80% power to detect values of RR_{ITT} < 0.80 when $\tau = 0.12$ and of RR_{ITT} < 0.55 when $\tau = 0.35$.

We next provide additional context on the corresponding proportion of patients in PROUD intervention clinics who would need to be treated for OUDs (p_{treat}) in order to detect the above values of RR_{ITT} when the underlying relative risk of acute care utilization comparing patients treated for OUDs versus those who are not treated ($RR_{treated}$) is 0.1 or 0.2.

	$\boldsymbol{\tau}$	$\boldsymbol{RR_{ITT}}$	**Proportion of patients needing to be treated for OUD ($\boldsymbol{p_{treat}}$)**	
			RR among treated ($RR_{treated}$)=	
			0.1	0.2
Sensitivity	0.12	< 0.80	>22%	>25%
**Primary**	0.23	< 0.65	>39%	>44%
Sensitivity	0.35	< 0.55	>50%	>56%

Thus, under our primary assumption for the random-effect SD (τ) if at least 39-44% of patients with OUD in the PROUD intervention arm at baseline are treated for OUD by the NCM, then we will have over 80% power to detect at least a 35% decrease in acute care utilization (RR_{ITT} < 0.65) comparing patients with OUD in the PROUD intervention arm versus UPC, when the true RR comparing treated to untreated patients with OUD ($RR_{treated}$) is 0.1-0.2. If fewer patients are treated, then our power would be less than 80% under these same assumptions.

7.0 DESCRIPTIVE ANALYSES

7.1 Analyses of Demographic and Baseline Data

The demographic variables for this study include age, sex, race/ethnicity, zip code-based characteristics (e.g., census variables), and type of insurance. The baseline clinical characteristics include diagnoses of medical conditions, mental health disorders, substance use disorders, and co-morbidity indices.

Descriptive statistics for baseline and demographic variables will be presented for the randomized clinics and for participants assigned to the clinics, overall and separately for each of the treatment arms (as well as secondary analyses within each HCS). Descriptive statistics will include N, mean, standard deviation, median, 25th and 75th percentiles, minimum and maximum for continuous variables and proportions and percentages for categorical variables. Since randomization is expected to produce balance at baseline between the two arms of the trial, statistical comparisons of treatment groups with respect to baseline characteristics will not be conducted. The updated CONSORT statement for parallel group-randomized trials no longer recommends formal testing of statistical significance of differences between baseline characteristics.[28]

7.2 Crossover

For all measures described below, patients will be assigned to a primary care clinic based on the algorithm stipulated above in the SAP (Section 3.0).

7.2.1 Main measures of Crossover of Patients with OUDs and Treated for OUDs

We will describe cross-over in terms of the following key measure:

**Cross-over between clinic arms***	The number of patients with OUDs assigned to each clinic (PROUD and UPC) in the pre-randomization period who are seen in the other clinic post-randomization.

For this measure, we will evaluate crossover in both directions, each separately (from PROUD to UPC and vice versa). However, the main analysis of interest will be the proportion of UPC patients with OUD diagnosed prior to randomization who are treated for OUDs in a PROUD clinic post-randomization.

7.2.2 Other General Measures of Crossover

In addition to evaluating crossover among patients *with an OUD diagnosis* in the 3 years prior to randomization, we will also describe crossover more generally, among *all patients seen* in the primary care clinic. Specifically, crossover will be defined as occurring if a patient assigned to a clinic in one arm of the trial (PROUD or UPC) during the 3 years prior to randomization is seen in a clinic in the other arm post-randomization. As above, we will evaluate crossover in both directions (separately), with the main analysis of interest the proportion of UPC patients who are treated for OUDs in a PROUD clinic post-randomization.

7.2.3 Crossover of Providers

If we observe that patients assigned to the UPC clinic (with or without an OUD diagnosis) are treated for OUDs post-randomization in the PROUD intervention clinic or vice versa, we will examine crossover of PC providers who prescribe buprenorphine. Specifically, we will examine whether PC providers move between randomized PROUD intervention and UPC clinics.

7.3 Implementation

We will describe, in each of the PROUD intervention clinics, the time from randomization until the NCM was (1) hired, (2) trained in Boston, (3) engaged the first patient in care in the PROUD intervention clinic, (4) left the study; (5) second NCM hired; and (6) engaged the first 10 patients in care in the PROUD intervention clinic. We will also summarize other relevant implementation milestones, such as when the Boston Training and Technical Assistance team conducted a clinic visit as well as those captured by the weekly NCM reports:

For each week

- # of New starts
- # of Re-engagements
- # of Transfers (patients new to NCM who were already on treatment)
- # of Discharges

Cumulative

- Total Ever Treated on Meds
- Total Currently Treated on Meds
- # of Starts for injectable BUP, Oral BUP, Oral NTX, Injectable NTX
- # of Re-engagements
- # of Transfers (patients new to NCM who were already on treatment)

7.4 Characteristics of Treated Patients

Key components of the PROUD intervention are to increase documentation and treatment of OUD among patients already in the clinic, and to attract new patients to receive care in the clinic who have not been seen previously in the clinic or who may be new to the HCS entirely. We therefore plan to describe, overall and by intervention arm, the proportion of patients with medication treatment for OUDs after randomization who were

1) In the clinic pre-randomization and
    a. had an EHR-documented OUD diagnosis (“documented OUD”) pre-randomization
    b. did not have documented OUD pre-randomization
2) Not in the clinic pre-randomization and
    a. were seen pre-randomization in the clinic of the HCS that was randomized to the other arm of the trial
        i. With documented OUD pre-randomization
        ii. Without documented OUD pre-randomization
    b. were seen pre-randomization in a different clinic of the HCS that was not randomized
        i. With documented OUD pre-randomization
        ii. Without documented OUD pre-randomization
    c. were not seen pre-randomization in the HCS

We will also describe these proportions among patients who engaged with the NCM.

7.5 Descriptive Analysis of Outcomes Among Treated Patients

Objective 2 effectiveness analyses (described above) provide an estimate of the intervention effect among patients with an OUD diagnosis who were in an intervention versus control clinic, regardless of whether the patient was actually treated by the NCM. For example, among patients with an OUD diagnosis at baseline, some may have left the HCS, some may not be interested in

receiving medication treatment for OUD, and some may be seeking treatment externally (e.g., via methadone maintenance). We therefore plan to additionally describe patient-level outcomes (e.g., acute care utilization, days of OUD medication treatment) among patients with an OUD diagnosis pre-randomization across levels of engagement with the NCM (e.g. seen by the NCM vs not).

We also plan to conduct descriptive analyses to characterize whether OUD treatment is being provided elsewhere within HCS before the patient sees the NCM, and is transferred to the PROUD intervention clinic, by describing the number of days of OUD treatment (pre- and/or post-randomization) a patient received prior to their first visit with the NCM using the most inclusive secondary measure (e.g., including dispensed buprenorphine; see Section 5.2.4).

7.6 Descriptive Information on Data Sources

We will describe the proportion of patients within each HCS with claims data available, which is expected to include most of the sample for one of the HCS and 10-30% enrolled patients from a second HCS. We will look at this overall and by clinic (intervention vs. control).

7.7 Health Care System-specific Descriptive Analyses of Outcome Measures

Because some HCS may have had different degrees of success in implementation, we plan to describe primary and secondary study outcome measures by study arm within each health care systems.

8.0 OTHER CONSIDERATIONS

8.1 Missing Data

Given that the primary and secondary outcomes rely on the EHR data or insurance claims data, if there is no evidence of a particular event, such as provision of buprenorphine or a visit to the ED, we will assume that the event did not occur. These same assumptions apply to the only covariate in the primary analyses of the primary Objective 1 and Objective 2 measures (baseline value of the respective measure in the 2 years prior to randomization).

Our primary Objective 1 outcome uses medication orders to capture days of OUD medication treatment as orders were available for all health systems and dispensings are considered incomplete at 5 of the 6 health systems. However, because dispensing data and not medication orders are considered the gold standard, we will conduct sensitivity analyses in which we estimate the primary outcome at each health system using only medication dispensing data in the 3 health systems that have some dispensing data and pharmacies in both the usual care and intervention clinics (Section 5.2.4).

For Objective 2, given our visit-based sample we are not able to identify when a patient is no longer observable. To address this, we will conduct a sensitivity analysis for our Objective 2 outcome among the two health systems that are health insurance plans in which we use enrolled samples (Section 5.4.3). We will also conduct sensitivity analyses that restrict to patients with evidence of being active in the health system post-randomization (as described above in Section 5.4.2).

Secondary analyses of the primary Objective 1 outcome and Objective 2 outcomes adjust for additional covariates as described above. Based on explorations of covariate distributions in prior data pulls (e.g., data pull 4), we anticipate that fewer than 6% of patients will have a missing value for any one of the individual covariates being considered (See Table 7). For Objective 1, in defining clinic-level covariates (e.g., proportion of patients with commercial insurance), we plan to exclude patients with missing covariate values when calculating clinic-level measures. For example, we will estimate the proportion of a clinic's patients who are commercially insured based on those with non-missing insurance status. Similarly, we will calculate the average neighborhood-level SES measures for a clinic based on patients with non-missing values (i.e., who have a zip code that was able to be linked to the census data). For Objective 2, for patients with missing covariate values, we plan to apply either mean imputation (for continuous covariates) or to use the indicator method (for categorical variables;[29] i.e., by including a "missing" category).

Table 7: Missingness in Covariates among Primary Care Patients with a Visit to one of the 12 Randomized Clinics During the Pre-randomization Period (N = 291,136 patients), Based on Data Pull 4

Covariate*	N (%) of patients with missing/unknown
Gender	4 (0.00%)
Race/ethnicity (combined version**)	16,825 (5.78%)
Insurance status (no insurance record, or recorded value listed as “unknown”)	11,082 (3.81%)
Neighborhood-level SES	
No zip code available to link to census data	4,422 (1.52%)
No zip code or zip code does not link to valid value	5,906 (2.03%)

* Only covariates with any missingness are shown; diagnosis-based measures (e.g., comorbidity flags) are assumed to not be present if a diagnosis is not documented

** HCSs ask about race/ethnicity in different ways: in some health systems, race is not documented if the patient reports ethnicity as Hispanic; thus, the combined race/ethnicity is used for covariate adjustment

9.0 SAFETY AND INTERIM ANALYSES

Due to the nature of this study—testing an implementation intervention, with all care provided by the health systems and using only secondary data—there are no formal interim analyses of safety performed. Further, all clinical care—and therefore responsibility for the quality of care—in this cluster-randomized pragmatic quality improvement trial is provided by the health systems. Therefore, this study monitored diverse measures of interest for the DSMB, including deaths and overdoses, but was not be able to intervene on the basis of any data and had no formal interim analyses linked to stopping rules. Since all care is provided by the health system, not the study, it would not be appropriate to intervene at the patient or provider level for any safety issue.

10.0 Table Shells

10.1 Objective 1 Paper

Table 1a. Characteristics of clinics' patient population in the pre-randomization period.

	**6 intervention clinics (No. patients =)[a]**	**6 usual care clinics (No. patients =)[a]**
	**Clinic Mean (SD)**	
**Staffing and Size of Clinics**		
Number of providers (MD, PA, ARNP) in clinic[b]		
Number of buprenorphine prescribers in clinic[c]		
Number of patients seen in clinic		
**Proportion of Clinics' Patient Population**		
Age, years		
16-17		
18-24		
25–44		
45–64		
65-74		
75+		
Female		
Race/ethnicity		
Hispanic ethnicity		
Non-Hispanic		
Asian		
Black or African American		
American Indian / Alaska Native		
Native Hawaiian or Pacific Islander		
White		
Multiple race		
Other race		
Missing race and ethnicity data		
Insurance status closest to randomization		
Medicare		
Medicaid		
Otherwise insured (e.g., commercial, private)		
Uninsured		
Unknown		
Patients' neighborhood[e]		
Median household income		
% unemployed		
% below federal poverty level		

**Table 1a**. Characteristics of clinics' patient population in the pre-randomization period.		
	**6 intervention clinics (No. patients =)[a]**	**6 usual care clinics (No. patients =)[a]**
	**Clinic Mean (SD)**	
Rurality-urbanicity[f]		
Urban		
Large rural city/town		
Small rural town		
Isolated small rural town		
Housing instability[g]		
Any mental health diagnoses [g]		
Depression		
Anxiety		
ADHD		
PTSD		
Schizophrenia/psychoses		
Other mental health conditions		
Any non-opioid SUD diagnoses[g]		
Tobacco		
Alcohol		
Cannabis		
Stimulant		
Other		
Other diagnoses[g]		
HCV infection		
HIV infection		
Non-cancer pain		
Elixhauser Comorbidity Index [h]		
0		
1		
2+		

[a] All patients with an eligible PC visit to one of the PROUD trial clinics during baseline (prior to randomization)
[b] Number and type of providers determined from encounter data in the electronic health record
[c] Prescribers determined from medication orders in the electronic health records. Providers assigned to clinics based on number of visits with patients in the clinic pre-randomization.
[d] At eligible visit closest to and prior to randomization
[e] Using zip code closest to randomization date
[f] based on rural urban commuting codes (RUCA) https://depts.washington.edu/uwruca/ruca-uses.php
g based on International Classification of Disease codes 2 years prior to randomization
[h] It is standard to calculate the Elixhauser using 1 year of data and thus was calculated using data in the year prior to randomization
Abbreviations: ADHD – attention deficit hyperactivity disorder; OUD – opioid use disorder; PTSD – post-traumatic stress disorder; SD – standard deviation; SUD – substance use disorder

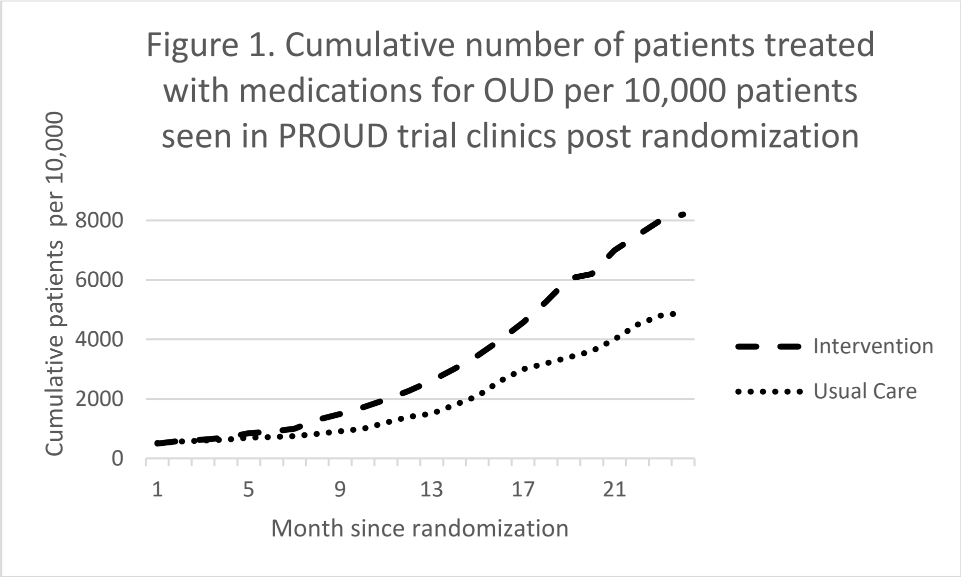

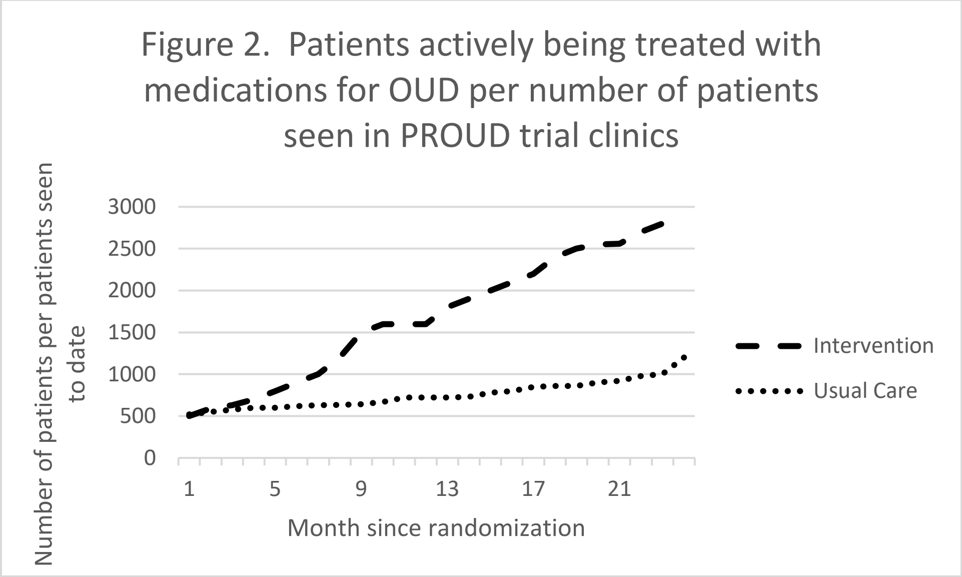

*Active defined as having 1+ days possession of medications to treat OUD during that month. Month defined as 30 days.

Table 1b. Opioid use disorder (OUD) and OUD treatment related characteristics of clinics in the PROUD trial in the 2 years prior to randomization, N=12

	**6 intervention clinics (No. patients =)[a]**	**6 usual care clinics (No. patients =)[a]**
	**Mean (SD) across clinics per 10,000 patients seen in the clinic pre-randomization**	
Patient years of OUD treatment with buprenorphine or XR-NTX, pre-randomization [a]		
Proportion of clinics' patient population pre-randomization with:		
OUD diagnosis		
Opioid overdose		
Other drug overdoses		
OUD treatment [a]		
Buprenorphine for OUD		
XR-NTX for OUD		
80% of days covered by OUD treatment[b]		
≥ 6-months retention in OUD treatment[c]		
Discontinuation of OUD treatment[d]		
Buprenorphine daily dose ≥16 mg[e]		
Naloxone prescribed[e]		

[a] Defined as having a medication order or procedure code for buprenorphine formulations that are indicated for OUD or having a medication order or procedure code for XR-NTX and 2+ visits with diagnosis code for OUD or opioid overdose during the pre-randomization period.
[b] Does not smooth over gaps between runout of one prescriptions and start of the next prescription.
[c] Restricted to subjects who entered the sample (i.e., had a PC visit) in the 6 months-2.0 years prior to randomization to allow for at least 6 months of follow-up
[d] Defined as a gap in OUD treatment of 60 days
[e] at any time during pre-randomization treatment
Abbreviations: OUD – opioid use disorder; SD – standard deviation; XR-NTX – extended release injectable naltrexone

Table 2. Clinic level primary and secondary implementation outcomes of the Primary Care Opioid Use Disorders Treatment (PROUD) trial, during 2 years post randomization.

Implementation measures	6 intervention clinics	6 usual care clinics	P value[a]
	Mean (SD) across clinics per 10,000 patients seen in clinic post randomization		
Patient years of OUD treatment (primary outcome[b])			
Proportion of clinics' patient population post-randomization with:			
Any OUD diagnosis post-randomization			
Any OUD treatment[b]			
80% of days covered by OUD treatment[c]			
≥ 6-months retention in OUD treatment[b,d]			
Discontinuation of OUD treatment[b,e]			
Buprenorphine daily dose ≥16 mg[f]			
Naloxone prescribed[f]			
Number of buprenorphine prescribers[g]			

[a] Random effects model adjusted for the outcome measure at baseline (two years prior to randomization)
[b] Treatment defined as having a medication order or procedure code for buprenorphine formulations that are indicated for OUD or having a medication order or procedure code for XR-NTX and 2+ visits with diagnosis code for OUD or opioid overdose (diagnosis codes can be during the 2-years pre or 2-years post randomization).
[c] Does not smooth over gaps between runout of one prescriptions and start of the next prescription
[d] Restricted to subjects who entered the sample (i.e., had a PC visit) in the 1 day to 1.5 years post randomization (1 day to 12 months for the site randomizing 6-months late) to enable at least 6 months of follow-up
[e] Defined as a gap in OUD treatment of 60 days
[f] At any time during OUD treatment post randomization
[g] Prescribers determined from medication orders in the electronic health records. Providers assigned to clinics based on number of visits with patients in the clinic post-randomization.
Abbreviations: NCM – nurse care manager; ITT- intent to treat; OUD: opioid use disorder; SD – standard deviation; XR-NTX – extended release injectable naltrexone

**Supplemental Table 1a** is the main Table 1a above by health system
**Supplemental Table 1b** is the main Table 1b above by health system
**Supplemental Table 2** is the main Table 1a above but using data for the post randomization period and among patients with an eligible visit post randomization (closest to study end as anchor for variables)
**Supplemental Table 3** is the main Table 2 above by health system

Table 4. Clinic level primary and secondary implementation outcomes of the Primary Care Opioid Use Disorders Treatment (PROUD) trial stratified by newly initiated treatment and new OUD diagnoses, during 2 years post randomization.

**Implementation measures**	**6 intervention clinics**	**6 usual care clinics**	**P value**[a]
	**Mean (SD) across clinics per 10,000 patients seen in clinic post randomization**		
Patient years of OUD treatment (primary outcome)[b]			
Newly initiated treatment[c]			
On-going treatment[d]			
Proportion of clinics' patient population post-randomization with:			
Any OUD diagnosis post-randomization			
New diagnosis[e]			
Prevalent diagnosis[f]			
Any OUD treatment[b]			
Newly initiated treatment[c]			
On-going treatment[d]			
80% of days covered by OUD treatment[g]			
Newly initiated treatment[c]			
On-going treatment[d]			
≥ 6-months retention in OUD treatment[b,h]			
Newly initiated treatment[c]			
On-going treatment[d]			
Discontinuation of OUD treatment[b,i]			
Newly initiated treatment[c]			
On-going treatment[d]			
Buprenorphine daily dose ≥16 mg[j]			
Newly initiated treatment[c]			
On-going treatment[d]			

[a] Random effects model adjusted for the unstratified outcome measure at baseline (two years prior to randomization)

Table 4. Clinic level primary and secondary implementation outcomes of the Primary Care Opioid Use Disorders Treatment (PROUD) trial stratified by newly initiated treatment and new OUD diagnoses, during 2 years post randomization.

Implementation measures	6 intervention clinics	6 usual care clinics	P value[a]
	Mean (SD) across clinics per 10,000 patients seen in clinic post randomization		

[b] Treatment defined as having a medication order or procedure code for buprenorphine formulations that are indicated for OUD or having a medication order or procedure code for XR-NTX and 2+ visits with diagnosis code for OUD or opioid overdose (diagnosis codes can be during the 2-years pre or 2-years post randomization).
[c] OUD treatment in the 2 years post randomization and no OUD treatment in the prior 365 days
[d] OUD treatment post-randomization that does not meet criteria for initiated above
[e] 1+ OUD diagnosis anywhere in the health system in the 2 years post randomization and no OUD diagnosis in the 2 years pre randomization.
[f] 1+ OUD diagnoses in both the 2 years pre randomization and 2-years post randomization
[g] Does not smooth over gaps between runout of one prescriptions and start of the next prescription
[h] Restricted to subjects who entered the sample (i.e., had a PC visit) in the 1 day to 1.5 years post randomization (1 day to 12 months for the site randomizing 6-months late) to enable at least 6 months of follow-up
[i] Discontinuation defined as a gap in OUD treatment of 60 days
[j] At any time during OUD treatment post randomization
Abbreviations: NCM – nurse care manager; ITT- intent to treat; OUD: opioid use disorder; SD – standard deviation; XR-NTX – extended release injectable naltrexone

Supplementary Table 5. Sensitivity analyses focused on assumptions made in estimating patient-years of OUD medication treatment, clinic-level results in the 2 years post randomization

	**Patient years treatment /10,000 patients seen in clinic post-randomization[a]**		
	**6 intervention clinics**	**6 usual care clinics**	**Mean difference (95% CI)[b]**
Primary outcome			---
Limited to the time period in which the NCM started seeing patients[d]			
Limited to health systems that successfully implement the MA model [e]			
Covariate adjusted[c]			
Using a combination of medication orders and pharmacy dispensings			
Adding Methadone Maintenance Therapy data from 1-2 sites, per 10,000 patients seen in the 1-2 clinics post randomization			
Adding pharmacy dispensings and MMT data at the 1-2 sites with these sources of data			
Omitting the last buprenorphine order			
Altering allowable gap to define continuous episodes of OUD treatment (main analysis allowable gap=7 days)			
2-day allowable gap			
5-day allowable gap			
10-day allowable gap			
Seen in the clinic pre-randomization			
OUD pre-randomization			
No OUD pre-randomization			
Patients new to the clinic post randomization			
Altering assignment of clinic to be the clinic with the most visits post randomization			

[a] Defined as having a medication order or procedure code for buprenorphine formulations that are indicated for OUD or having a medication order or procedure code for injectable naltrexone and 2+ visits with an OUD or OD diagnosis code (diagnosis codes can be during the 2-years pre or 2-years post randomization)
[b] Mean difference in the primary outcome comparing clinics randomized to the PROUD intervention vs. clinics randomized to usual care, estimated from a random effects model adjusted for the primary outcome measure at baseline (two years prior to randomization)

[c] Adjusted for {*2 covariates determined from lasso regression*} and baseline OUD treatment in random effects model

Supplementary Table 6. Implementation of OUD medication treatment at the clinic-level in the 2 years post randomization stratified by patient characteristics, Primary Care Opioid Use Disorders Treatment (PROUD) trial

	**6 intervention clinics**	**6 usual care clinics**	
	**Mean (SD) patient years of treatment across clinics per 10,000 patients in the subgroup**		**P value[b]**
Age at eligible visit closest to randomization[a]			
<26			
≥ 26			
Sex			
Female			
Male			
Race/Ethnicity[c]			
Hispanic			
Non-Hispanic			
White			
Black			
Asian			
Other			

[a] Eligible primary care visits closest to and prior to randomization. If no eligible visits prior to randomization, post-randomization visit closest to randomization date was chosen.
[b] P values are presented for omnibus tests evaluating whether there is any difference in the intervention effect across subgroups, as well as for differences in subgroup-specific intervention effects, obtained from a linear mixed model with interaction terms between the intervention group and the subgroup
[c] X individuals dropped due to missing race and/or ethnicity.

Table 7. Clinic level primary and secondary implementation outcomes of the Primary Care Opioid Use Disorders Treatment (PROUD) trial among patients receiving OUD treatment during the 2 years post randomization.

Scaled implementation measure among treated patients	6 intervention clinics	6 usual care clinics	P value[a]
	Mean (SD) across clinics per 100 patients treated in the clinic post randomization		
Patient years of OUD treatment[b]			
Primary outcome (ITT analysis)[c]			
Limited to the time period after the NCM started seeing patients[d]			
Limited to health systems that successfully implement the MA model [e]			
Number of patients with:			
Newly initiated treatment [a, f]			
On-going treatment[a,g]			
80% of days covered by OUD treatment			
Newly initiated treatment [a, f]			
On-going treatment[a,g]			
≥ 6-months retention in OUD treatment [h]			
Newly initiated treatment[a,f]			
On-going treatment[a,g]			
Discontinuation of OUD treatment[i]			
Newly initiated treatment[a,f]			
On-going treatment[a,g]			
Buprenorphine average daily dose in mg			
Buprenorphine daily dose ≥16 mg[j]			
Naloxone prescription			

[a] Random effects model adjusted for the primary outcome measure at baseline (two years prior to randomization);
[b] Defined as having a medication order or procedure code for buprenorphine formulations that are indicated for OUD or having a medication order or procedure code for XR-NTX and 2+ visits with diagnosis code for OUD or opioid overdose (diagnosis codes can be during the 2-years pre or 2-years post randomization)
[c] Includes all clinics and all follow-up time post randomization
[d] Time period during which NCM seeing patients: X days for clinic A, X days for clinic B, X days for clinic C, X days for clinic D, X days for clinic E, and X days for clinic F
[e] Successful implementation defined as the nurse care manager seeing at least 30 patients. Analysis includes X of 6 health systems.
[f] OUD treatment in the 2 years post randomization and no OUD treatment in the prior 365 days
[g] OUD treatment in post-randomization, but not meeting criteria for initiated
[h] Restricted to subjects who entered the sample (i.e., had a PC visit) in the 1 day to 1.5 years post randomization (1 day to 12 months for the site randomizing 6-months late) to enable at least 6 months of follow-up
[i] defined as a gap in OUD treatment of 60 days
[j] 16 mg or more of buprenorphine/day at any time post randomization

Table 7. Clinic level primary and secondary implementation outcomes of the Primary Care Opioid Use Disorders Treatment (PROUD) trial among patients receiving OUD treatment during the 2 years post randomization.

Scaled implementation measure among treated patients	6 intervention clinics	6 usual care clinics	P value[a]
	Mean (SD) across clinics per 100 patients treated in the clinic post randomization		

Abbreviations: NCM – nurse care manager; ITT- intent to treat; OUD: opioid use disorder; SD – standard deviation; XR-NTX – extended release injectable naltrexone

10.2 Objective 2 Paper

Figure 1. Cohort identification of patients for the primary and larger secondary sample of the PROUD Trial effectiveness analyses

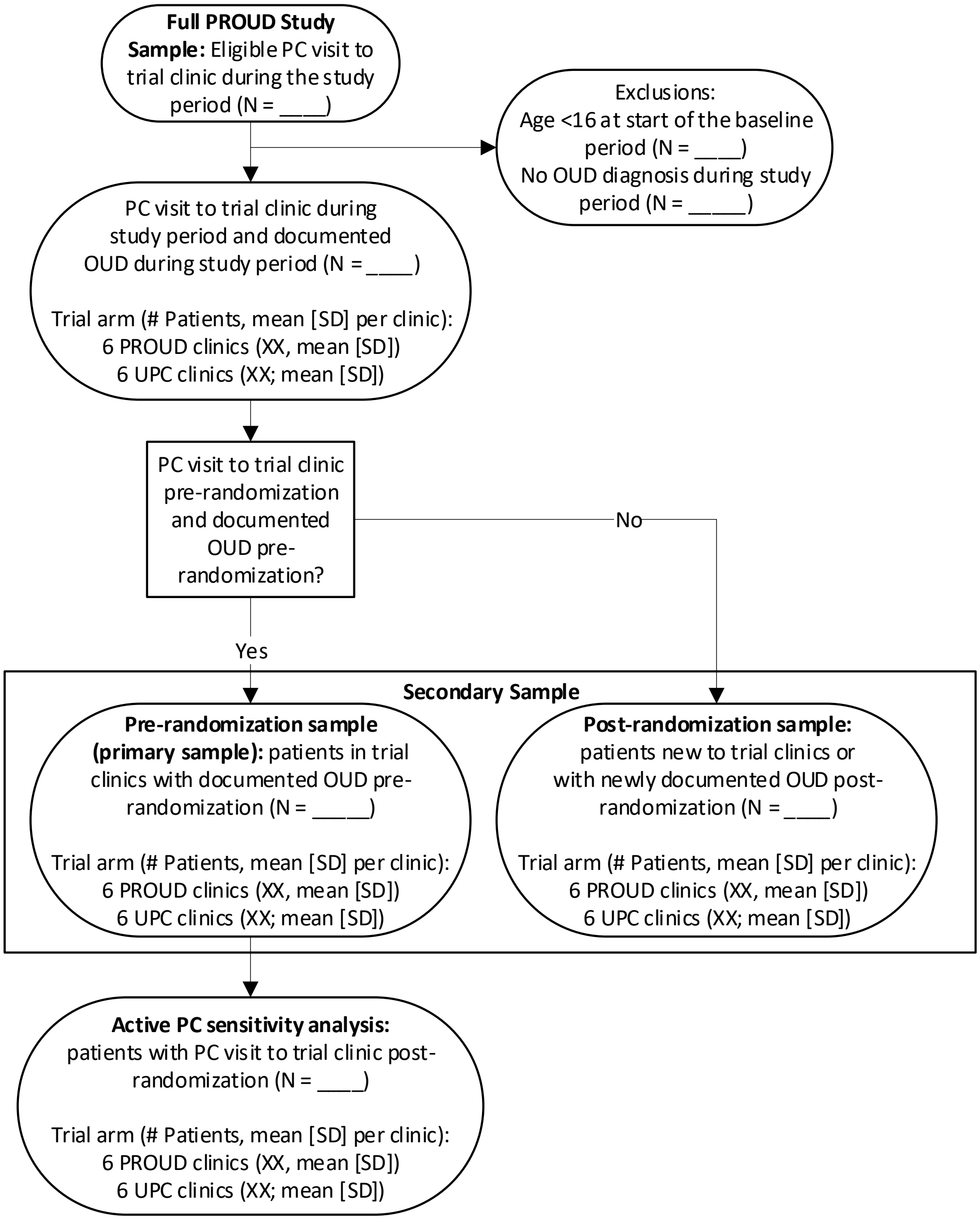

Supplemental Figure 1. Samples of patients in the primary and secondary Objective 2 analysis

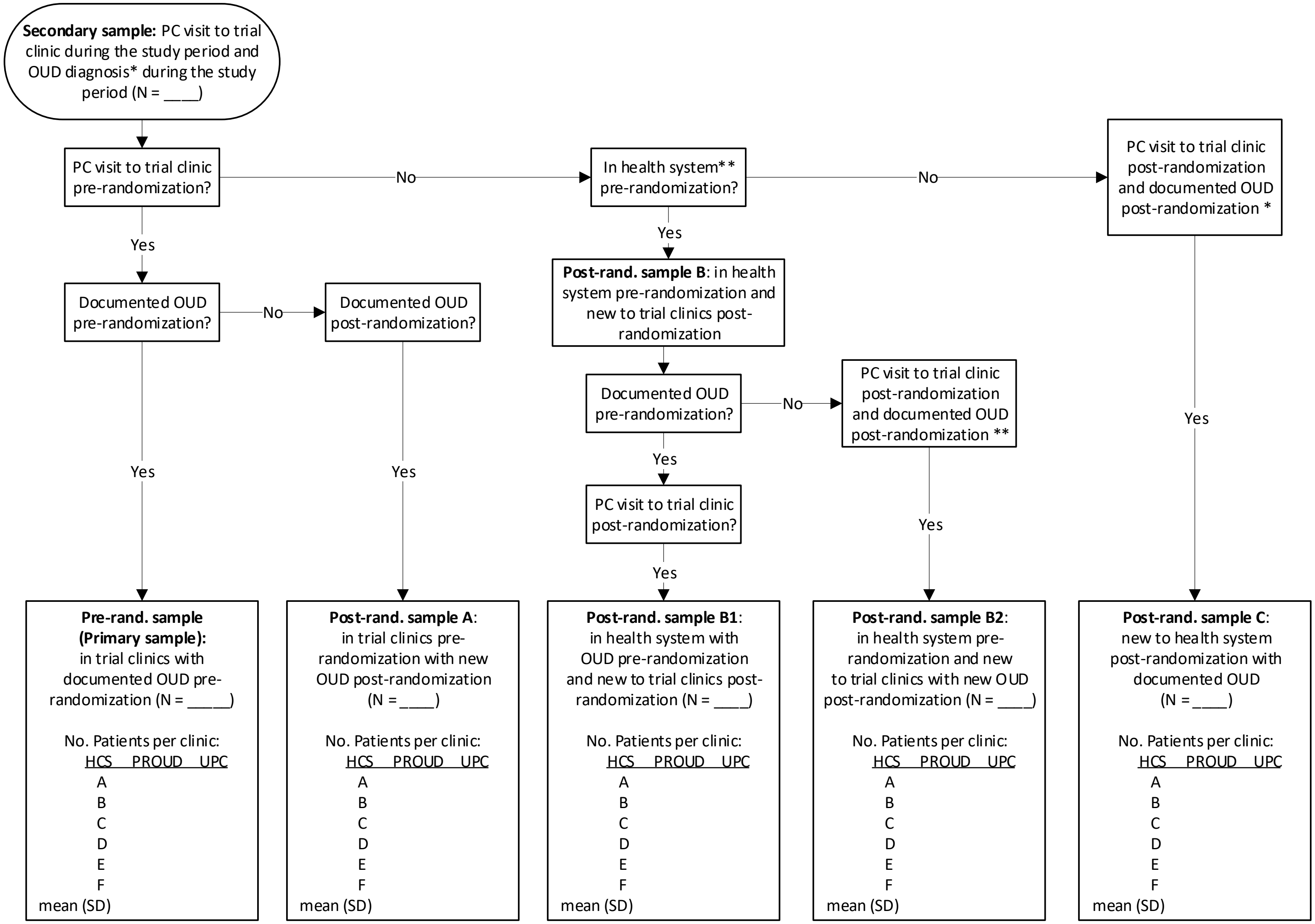

HCS = health care system
rand. = randomization
* OUD diagnosis anywhere in the health system
** Defined by having any visit, diagnosis, or procedure

Table 1. Characteristics of primary care patients with documented OUD who visited PROUD trial clinics (Effectiveness Objective Samples)

	**Pre-randomization Sample:** Patients in Trial Clinics with Documented OUD Pre-randomization		**Post-randomization Sample** [a, b]**:** Patients New to Trial Clinics or with Newly Documented OUD Post-randomization	
**Characteristic**	**Intervention** (N =)	**Usual Care** (N =)	**Intervention** (N =)	**Usual Care** (N =)
	%			
Age at randomization, years				
16-17				
18–24				
25–44				
45–64				
65–74				
75+				
Female				
Race/ethnicity				
Hispanic ethnicity				
Non-Hispanic				
Asian				
Black or African American				
American Indian / Alaska Native				
Native Hawaiian or Pacific Islander				
White				
Multiple race				
Other race				
Missing race and ethnicity data				

Table 1. Characteristics of primary care patients with documented OUD who visited PROUD trial clinics (Effectiveness Objective Samples)

	**Pre-randomization Sample:** Patients in Trial Clinics with Documented OUD Pre-randomization		**Post-randomization Sample** [a, b]**:** Patients New to Trial Clinics or with Newly Documented OUD Post-randomization	
**Characteristic**	**Intervention** (N =)	**Usual Care** (N =)	**Intervention** (N =)	**Usual Care** (N =)
Insurance status closest to randomization [b]				
Medicare				
Medicaid				
Otherwise insured (e.g., commercial, private)				
Uninsured				
Unknown				
Unstable housing including homelessness [b]				
Socioeconomic status variables using zip code closest to randomization, median (IQR)				
Median household income				
% unemployed				
% below federal poverty level				
Comorbidity in the two years pre-randomization [b]				
Alcohol use disorder				
Other (non-opioid) substance use disorder				
Schizophrenia and other psychotic disorders				

Table 1. Characteristics of primary care patients with documented OUD who visited PROUD trial clinics (Effectiveness Objective Samples)

	**Pre-randomization Sample:** Patients in Trial Clinics with Documented OUD Pre-randomization		**Post-randomization Sample** [a, b]**:** Patients New to Trial Clinics or with Newly Documented OUD Post-randomization	
**Characteristic**	**Intervention** (N =)	**Usual Care** (N =)	**Intervention** (N =)	**Usual Care** (N =)
**Baseline OUD treatment** [b]				
Days of treatment per year, mean (IQR)				
OUD treatment duration				
0 days				
1-30 days				
31-90 days				
91-180 days				
180+ days (sustained OUD treatment)				
**Baseline acute care utilization** [b]				
Days of acute care utilization per year, mean (IQR)				
Days hospitalized [c]				
Days of emergency care [d]				

Table 1. Characteristics of primary care patients with documented OUD who visited PROUD trial clinics (Effectiveness Objective Samples)

	**Pre-randomization Sample:** Patients in Trial Clinics with Documented OUD Pre-randomization		**Post-randomization Sample** [a, b]**:** Patients New to Trial Clinics or with Newly Documented OUD Post-randomization	
**Characteristic**	**Intervention** (N =)	**Usual Care** (N =)	**Intervention** (N =)	**Usual Care** (N =)
Proportion of patients with:				
Any acute care				
Hospitalization [c]				
Never				
Once				
2-3 times				
4+ times				
Emergency care visit [d]				
Never				
Once				
2-3 times				
4+ times				

HCS = health care system

[a] Includes patients in the trial clinics without an OUD diagnosis pre-randomization who had an OUD diagnosis post-randomization, patients in the HCS (but not in the trial clinics) with an OUD diagnosis pre-randomization or post-randomization, and patients who were new to the HCS post-randomization (see Supplemental Figure)

[b] Patients in the HCS pre-randomization use baseline values of time-varying covariates; patients new to HCS post-randomization do not have baseline values of time-varying covariates (insurance status and zip code use post-baseline

measure available closest to randomization date; clinical characteristics use two years post-randomization; baseline treatment and outcomes not available)

[c] Days hospitalized also includes emergency department or urgent care visits that resulted in a hospitalization. [Report % of hospitalizations that started in (or immediately preceded) an emergency visit.]

[d] Emergency care includes visits to an emergency department or urgent care facility that did not result in hospitalization

Table 3. Effect of PROUD Intervention on acute care utilization among primary care patients with documented OUD pre-randomization

**Measure of Acute Care Utilization in 2 years post-randomization**	**Intervention** (n=xxx)	**Usual Care** (n=xxx)	Treatment Effect [a]	95% CI	P value
	mean (IQR) days per year [b]		RR		
**Primary outcome:** days of acute care utilization (intention to treat)					
Sub-samples and/or follow-up periods restricted to:					
11.0 Clinics that successfully implemented MA model [c]					
12.0 Time period in which the NCM was seeing patients					
13.0 Patients with PC visit to assigned clinic post-randomization					
14.0 Both b and c					
Days of hospitalization [d] (intention to treat)					
Sub-samples and/or follow-up periods restricted to:					
a. Clinics that successfully implemented MA model [c]					
b. Time period in which the NCM was seeing patients					
c. Patients with PC visit to assigned clinic post-randomization					
d. Both b and c					
Days of emergency care [e] (intention to treat)					
Sub-samples and/or follow-up periods restricted to:					
a. Clinics that successfully implemented MA model [c]					
b. Time period in which the NCM was seeing patients					
c. Patients with PC visit to assigned clinic post-randomization					
d. Both b and c					

Table 3. Effect of PROUD Intervention on acute care utilization among primary care patients with documented OUD pre-randomization

Measure of Acute Care Utilization in 2 years post-randomization	Intervention (n=xxx)	Usual Care (n=xxx)	Treatment Effect [a]	95% CI	P value
	mean (IQR) days per year [b]		RR		
Proportion of patients with:	N (%)[b]		OR		
Any acute care					
Hospitalization [d]					
Never					
Once					
2-3 times					
4+ times					
Emergency care visit [e]					
Never					
Once					
2-3 times					
4+ times					

[a] Estimated from a mixed-effect model with clinic-specific random intercept, adjusted for the baseline value of the outcome (binary/categorical measures adjust for the continuous value of the measure)

[b] Unadjusted estimates. [We note that if we observe a statistically significant difference from the adjusted model, but the unadjusted estimates appear similar then we plan to also calculate the adjusted estimates.]

Table 3. Effect of PROUD Intervention on acute care utilization among primary care patients with documented OUD pre-randomization

Measure of Acute Care Utilization in 2 years post-randomization	Intervention (n=xxx)	Usual Care (n=xxx)	Treatment Effect [a]	95% CI	P value
	mean (IQR) days per year [b]		RR		

[c] Successful implementation is defined as the NCM at the intervention clinic at that HCS seeing at least 30 patients. [We note that estimates from the secondary analyses of the main treatment measure may instead be separated into a different paper or included in a supplemental table.]

[d] Days hospitalized also includes emergency department or urgent care visits that resulted in a hospitalization. [Report % of hospitalizations that started in (or immediately preceded) an emergency visit.] Specific cut-points for categorization to be determined based on empirical distribution at baseline

[e] Emergency care includes visits to an emergency department or urgent care facility that did not result in hospitalization; specific cut points for categorization to be determined based on empirical distribution at baseline

Table 4. Observational analysis of effect of PROUD Intervention on OUD treatment in the larger secondary sample that includes patients with newly recognized OUD in trial clinics post-randomization

	**Pre-randomization Sample:** Patients in Trial Clinics with Documented OUD Pre-randomization [a]		**Post-randomization Sample:** Patients New to Trial Clinics or with Newly Documented OUD Post-randomization				**Secondary Sample** (Overall)
**Measure**	Treatment effect [b]	95% CI	**Intervention**	**Usual Care**	Treatment effect [b]	95% CI	Omnibus P value [c]
Same measures as Table 2							

[a] Estimating the same thing as Table 2, but adjusted for demographics and not adjusting for baseline (which is not measured among patients new to the health system post-randomization); likely will be omitted from main paper (include in supplement instead)

[b] Estimated from a mixed-effect model with clinic-specific random intercept and an interaction term between the intervention effect and the timing of when the patient was first seen in the trial clinic with diagnosed OUD (pre- or post-randomization) and adjusted for demographics
[c] Testing for whether there is a difference between intervention and usual care clinics for either subset of patients (primary sample or secondary sub-sample added to the primary sample)

Table 5. Observational analysis of intervention effect on acute care utilization in the larger secondary sample including patients with newly recognized OUD in trial clinics post-randomization

	**Pre-randomization Sample:** Patients in Trial Clinics with Documented OUD Pre-randomization [a]		**Post-randomization Sample:** Patients New to Trial Clinics or with Newly Documented OUD Post-randomization				**Secondary Sample** (Overall)
**Measure**	RR [b]	95% CI	**Intervention**	**Usual Care**	Treatment Effect [b]	95% CI	Omnibus P value [c]
Same measures as Table 3							

[a] Estimating the same thing as Table 3, but adjusted for demographics and not adjusting for baseline (which is not measured among patients new to the health system post-randomization); likely will be omitted from main paper
[b] Estimated from a mixed-effect model with clinic-specific random intercept and an interaction term between the intervention effect and the timing of when the patient was first seen in the trial clinic with diagnosed OUD (pre- or post-randomization) and adjusted for demographics
[c] Testing for whether there is a difference between intervention and usual care clinics for either subset of patients (primary sample or secondary sub-sample added to the primary sample)

11.0 REFERENCES

1. Alford DP, LaBelle CT, Kretsch N, Bergeron A, Winter M, Botticelli M, Samet JH. Collaborative care of opioid-addicted patients in primary care using buprenorphine: five-year experience. Arch Intern Med 2011;171:425-31.
2. Curran GM, Bauer M, Mittman B, Pyne JM, Stetler C. Effectiveness-implementation hybrid designs: combining elements of clinical effectiveness and implementation research to enhance public health impact. Med Care 2012;50:217-26.
3. Bobb JF, Qiu H, Matthews AG, McCormack J, Bradley KA. Addressing identification bias in the design and analysis of cluster-randomized pragmatic trials: a case study. Trials 2020;21:289.
4. Gordon AJ, Lo-Ciganic WH, Cochran G, Gellad WF, Cathers T, Kelley D, Donohue JM. Patterns and Quality of Buprenorphine Opioid Agonist Treatment in a Large Medicaid Program. J Addict Med 2015;9:470-7.
5. Bryson CL, Au DH, Sun H, Williams EC, Kivlahan DR, Bradley KA. Alcohol screening scores and medication nonadherence. Ann Intern Med 2008;149:795-804.
6. Hedeker D, Gibbons RD, Flay BR. Random-effects regression models for clustered data with an example from smoking prevention research. J Consult Clin Psychol 1994;62:757-65.
7. Tibshirani R. Regression shrinkage and selection via the lasso. Journal of the Royal Statistical Society. Series B (Methodological). 1996 Jan 1:267-88.
8. Friedman J, Hastie T, Tibshirani R. Regularization Paths for Generalized Linear Models via Coordinate Descent. J Stat Softw 2010;33:1-22.
9. Austin PC, Steyerberg EW. The number of subjects per variable required in linear regression analyses. J Clin Epidemiol 2015;68:627-36.
10. Bhatraju EP, Grossman E, Tofighi B, McNeely J, DiRocco D, Flannery M, Garment A, Goldfeld K, Gourevitch MN, Lee JD. Public sector low threshold office-based buprenorphine treatment: outcomes at year 7. Addict Sci Clin Pract 2017;12:7.
11. Burns L, Gisev N, Larney S, Dobbins T, Gibson A, Kimber J, Larance B, Mattick RP, Butler T, Degenhardt L. A longitudinal comparison of retention in buprenorphine and methadone treatment for opioid dependence in New South Wales, Australia. Addiction 2015;110:646-55.
12. Gryczynski J, Mitchell SG, Jaffe JH, O'Grady KE, Olsen YK, Schwartz RP. Leaving buprenorphine treatment: patients' reasons for cessation of care. J Subst Abuse Treat 2014;46:356-61.
13. Hser YI, Saxon AJ, Huang D, Hasson A, Thomas C, Hillhouse M, Jacobs P, Teruya C, McLaughlin P, Wiest K, Cohen A, Ling W. Treatment retention among patients randomized to buprenorphine/naloxone compared to methadone in a multi-site trial. Addiction 2014;109:79-87.
14. Marcovitz DE, McHugh RK, Volpe J, Votaw V, Connery HS. Predictors of early dropout in outpatient buprenorphine/naloxone treatment. Am J Addict 2016;25:472-7.
15. Weinstein ZM, Kim HW, Cheng DM, Quinn E, Hui D, Labelle CT, Drainoni ML, Bachman SS, Samet JH. Long-term retention in Office Based Opioid Treatment with buprenorphine. J Subst Abuse Treat 2017;74:65-70.
16. Lagisetty PA, Ross R, Bohnert A, Clay M, Maust DT. Buprenorphine Treatment Divide by Race/Ethnicity and Payment. JAMA Psychiatry 2019;76:979-81.
17. Lo-Ciganic WH, Gellad WF, Gordon AJ, Cochran G, Zemaitis MA, Cathers T, Kelley D, Donohue JM. Association between trajectories of buprenorphine treatment and emergency department and in-patient utilization. Addiction 2016;111:892-902.
18. Hansen H, Siegel C, Wanderling J, DiRocco D. Buprenorphine and methadone treatment for opioid dependence by income, ethnicity and race of neighborhoods in New York City. Drug Alcohol Depend 2016;164:14-21.

19. Stein MD, Cioe P, Friedmann PD. Buprenorphine retention in primary care. J Gen Intern Med 2005;20:1038-41.
20. Tibshirani R. The lasso method for variable selection in the Cox model. Stat Med 1997;16:385-95.
21. Kahan BC, Forbes G, Ali Y, Jairath V, Bremner S, Harhay MO, Hooper R, Wright N, Eldridge SM, Leyrat C. Increased risk of type I errors in cluster randomised trials with small or medium numbers of clusters: a review, reanalysis, and simulation study. Trials 2016;17:438.
22. Leyrat C, Morgan KE, Leurent B, Kahan BC. Cluster randomized trials with a small number of clusters: which analyses should be used? Int J Epidemiol 2018;47:321-31.
23. Bell J, Turbow S, George M, Ali MK. Factors associated with high-utilization in a safety net setting. BMC Health Serv Res 2017;17:273.
24. Mohlman MK, Tanzman B, Finison K, Pinette M, Jones C. Impact of medication-assisted treatment for opioid addiction on Medicaid expenditures and health services utilization rates in Vermont. J Subst Abuse Treat 2016;67:9-14.
25. Robinson RL, Grabner M, Palli SR, Faries D, Stephenson JJ. Covariates of depression and high utilizers of healthcare: Impact on resource use and costs. J Psychosom Res 2016;85:35-43.
26. Gagne JJ, Glynn RJ, Avorn J, Levin R, Schneeweiss S. A combined comorbidity score predicted mortality in elderly patients better than existing scores. J Clin Epidemiol 2011;64:749-59.
27. LaBelle CT, Han SC, Bergeron A, Samet JH. Office-Based Opioid Treatment with Buprenorphine (OBOT-B): Statewide Implementation of the Massachusetts Collaborative Care Model in Community Health Centers. J Subst Abuse Treat 2016;60:6-13.
28. Schulz KF, Altman DG, Moher D; CONSORT Group. CONSORT 2010 Statement: updated guidelines for reporting parallel group randomised trials. BMC Med. 2010 Mar 24;8:18.
29. White IR, Thompson SG. Adjusting for partially missing baseline measurements in randomized trials. Stat Med 2005;24:993-1007.

Appendix A. Algorithms for Days of OUDs Treatment

A1. Operationalization to Estimate Number of Patient-days of Treatment for Opioid Use Disorders from Electronic Health Records and Claims Data

12.0 The data used to estimate patient-days of treatment for OUD can include medication orders from the EHR, and procedure codes from the EHR. Medication dispensings from pharmacy claims and the EHR will be considered in sensitivity analyses only. Medication orders (and dispensings) commonly include variables for the drug name (generic and/or brand); date dispensed/ordered; quantity dispensed or ordered; intended days' supply (dispensings only); directions for use (used to estimate days' supply for orders); strength per unit; and prescriber. Procedure data includes the drug name, strength, date administered, and provider. Using the data noted above, we estimate runout dates (date when the OUD treatment days provided by that particular dispensing/order/procedure ends) for each order/procedure (and dispensings in sensitivity analyses) of OUD treatment (buprenorphine [Table A1] and naltrexone) by adding the intended or estimated days' supply to the start date (date of dispensing, order, or procedure). Sublocade and naltrexone injections are assumed to provide 28 days of OUD treatment (i.e., days' supply=28). Probuphine is assumed to provide 6 months of treatment. Each unique order, and procedure (and dispensings when included), are then combined into an episode or episodes (if gaps) of OUD treatment. Episodes are then summed to calculate the total patient days covered with OUD treatment in a given time period. See Section A3 for details on how episodes are estimated from the unique orders and procedures (and dispensings when included).

13.0 Often data obtained from clinical and/or administrative systems confronts us with missing data fields (i.e., quantity dispensed/ordered or intended days' supply) or situations that would be very unlikely or impossible, such as an unrealistic daily dose of buprenorphine or a day's supply greater than 6 months for a controlled substance. Therefore, as part of Phase 1 of CTN-0074, we developed data cleaning rules to address these situations. Section A2 outlines some of the key situational assumptions.

Table A1: Buprenorphine Formulations Included for OUD Treatment

Medication Name(s)	Route of Administration (form)	Strength in Milligrams (mg)
Buprenorphine (Brands: Subutex®)	Sublingual tablet and film	2 mg, 8 mg
Buprenorphine/naloxone (Brands: Suboxone®, Cassipa®)	Sublingual tablet and film	2 mg/0.5 mg, 4mg/1mg, 8 mg/2 mg, 12mg/3mg, 16mg/4mg
Bunavail® (buprenorphine/naloxone)	Buccal film	2.1 mg/0.3 mg, 4.2 mg/0.7 mg, 6.3 mg/1 mg
Buprenorphine/naloxone (Brand: Zubsolv®	Sublingual tablet	1.4 mg/0.36 mg, 2.9 mg/0.71 mg, 5.7 mg/1.4 mg, 8.6 mg/2.1 mg, 11.4 mg/2.9 mg
Buprenorphine (Brand: Probuphine®)	Subdermal implants	4 single rods each 74.2 mg
Buprenorphine (Brand: Sublocade)	Subcutaneous injection	100 , 300 mg

A2. Data Cleaning and Quality Checks for Individual Oral Buprenorphine Orders (and dispensings for sensitivity analyses)

Variables	Definition
Sid	Unique study id
rxname	Drug name (e.g., buprenorphine, naltrexone, buprenorphine and naloxone)
rxamt	Quantity ordered (e.g., 30 tablets)
rxsup	Supply of the order or how long the order should last (e.g., 30 days)<br>This is determined for orders by 1) converting SIG (directions for use) into pills per day (PPD). For example, “take 1 tablet twice a day” is 2 PPD; 2) dividing the rxamt by ppd. For example, an order for rxamt=30 that has PPD=2 would have a rxsup=30/2 or 15 days.<br>Days supply is then rounded to nearest whole number.
strength	Mg in one dose (e.g., 8 mg)
daily dose (DD)	=PPD * strength, for example, 2 PPD of 8 mg bup is 16 mg/day.
date	Date medication was ordered or dispensed

1) Summarize duplicates for orders. Duplicates defined as orders for the same **patient (sid)** with same rxname, date, and strength.
   - Number of total orders =
   - Number of unique subjects =
   - Number of subjects with at least 1 duplicate pair:
   - Percent of subjects with at least 1 duplicate pair:
   - Number of total orders that are considered a potential duplicate =
     - Example: 6 orders for the same patient where orders 1&2 have the same rxname, order date, and strength, and orders 3-5 also have the same rxname, date, and strength. This is a total of 5 duplicates. The percent of duplicate pairs across all this patient’s orders is 5 divided by 6 (total number of orders).
   - Percent of duplicate pairs across total orders:
2) Provide potential duplicates to health systems and ask that they chart review and de-dupe where needed (data anomaly) or provide revised order “date” if they are not duplicates
3) **De-dupe remaining duplicates**. If rxsup is different or 1+ is missing, keep the one with the largest rxsup. If rxsup is the same but quantity differs, keep the one with the largest quantity. If rxsup and quantity are the same across dupes, randomly keep one order.
   - Number of total orders =
   - Number of unique subjects (should not change):
4) Derive a pills per day (PPD) variable =Rxamt / Rxsup for each bup rx where rxamt and rxsup are known and reasonable (not missing, not zero, not <1).
5) Calculate daily dose (DD) variable = PPD x strength (where strength is known). Round DD to an integer for each bup rx.
6) Set missing refills to = 0

7) Provide frequencies in table 1 and table 2 below for buprenorphine. This is post de-dupe. (The days supply in health system C are calculated as quantity/ppd before calculating the frequencies. They are originally missing.)

**Table 1a.** Proportion of orders and unique individuals with data anomalies		
	**No. bup orders=**	**No. subjects=**
	**n (%)**	
**Missing**		
Strength		
DD		
Rxsup		
Rxamt		
Both rxsup & rxamt		
Missing strength, rxsup, & rxamt		
**Zero value**		
Rxsup		
Rxamt		
Both Rxsup & rxamt		
**<0 as a value**		
Rxsup		
Rxamt		
Both Rxsup & rxamt		
**Specific cut points**		
DD>40		
DD>40 in 3+ consecutive Rx		
DD>40 in < 3 consecutive Rx		
Rxsup<1		
Rxsup>30		
Rxsup >60		
Rxsup>90		
Rxsup>180		
Rxsup=1 & DD>8 mg		
Refills ≥1		
Refills >6		
Rxamt<1		
Rxamt=1		

**Table 1a.** Proportion of orders and unique individuals with data anomalies		
	**No. bup orders=**	**No. subjects=**
	**n (%)**	
Rxamt >30		
Rxamt >60		
Rxamt >90		

**Table 2a**. Distribution of values for raw variables of interest						
	**Min**	**25th**	**50th**	**75th**	**Max**	**% above 3 times 75th percentile**
DD						
Strength						
Rxamt						
Rxsup						
Refills						

8) Output all bup orders for subjects with outliers (defined as above 3 x the 75th percentile) for rxsup and/or rxamt.
9) Output all bup orders for subjects with rxup and/or rxamt less than 1.
10) Co-I reviews all bup orders identified in steps 7 and 8 to flag which outliers are likely legit and should not be imputed. Co-I to determine if and what to impute for rxsup or rxamt <1.
11) For rxsup and rxamt outliers that are to be imputed and for rxsup and rxamt values <1 that are to be imputed, set to missing.
12) Impute all missing rxsup and rxamt as follows below. We do not need to impute refills, strength, ppd, or DD.
    - Use the most common value from the same patient's bup rx w/in +/- 100 days of missing field Take the value closest to missing if there is a tie in the # of occurrences. If tie in both, use the smallest value.
    - If the patient has no other bup rx w/in +/- 100 days of the missing, impute the median value from all bup rx at that health system. If all bup rx are missing the field at that health system, impute the median value from all bup rx at all health systems.
13) Populate Table 1b and 2b below (post imputation of bup)

**Table 1b.** Proportion of buprenorphine orders and unique individuals with data anomalies		
	**No. bup orders=**	**No. subjects=**
	n (%)	

**Specific cut points**		
DD>40		
DD>40 in 3+ consecutive Rx		
DD>40 in < 3 consecutive Rx		
Rxsup<1		
Rxsup>30		
Rxsup >60		
Rxsup>90		
Rxsup>180		
Rxsup=1 & DD>8 mg		
Refills ≥1		
Refills >6		
Rxamt<1		
Rxamt=1		
Rxamt >30		
Rxamt >60		
Rxamt >90		

Table 2b. Distribution of values for raw variables of interest that were imputed

	**Min**	**25th**	**50th**	**75th**	**Max**
Rxamt					
Rxsup					

14) Pull in buprenorphine (J0570, G2070, G2072, Q9991, Q9992, G2068, G2079, G2069, J0571, J0572, J0573, J0574, J057), injectable naltrexone (J2315, G2073, HZ84ZZZ, HZ94ZZZ), and methadone maintenance therapy (HZ91ZZZ, HZ81ZZZ, H0020, G2067, G2078) procedures from procedures data tables and injectable naltrexone (defined as brand= Vivitrol or route=intramuscular) from medication order tables.

**Procedure data**	**Number of occurrences**	**Unique number of patients**
Implant probuphine - J0570, G2070, G2072		
Sublocade - Q9991, Q9992, or G2069		

Oral bup - J0571, J0572, J0573, J0574, or J0575, G2068, G2079		
Oral bup procedure codes after adding up ones billed on same day (see Step 14)		
XR injectable naltrexone procedures (J2315, G2073, HZ84ZZZ, HZ94ZZZ)		
XR injectable naltrexone from medication orders		
Methadone maintenance therapy (HZ91ZZZ, HZ81ZZZ, H0020, G2067, G2078)		

15) Create a start and end date for each bup rx and procedure
- Start date=rxdate (orders) or adate (procedures)
- For bup orders: End date= (rxdate + rxsup) -1
  - If refills, multiply rxsup by number of refills +1 to calculate the end date. For example, an order with one refill and rxsup=30 would be 30*2 = 60 days. An order with 2 refills and rxsup=30 would be 30*3=90 days.
- For oral bup procedures (J0571, J0572, J0573, J0574, J0575), set rxsup=1 and enddate=(adate + rxsup)-1. Essentially, each procedure code for oral med covers 1 day. If duplicates (same day for any of these 5 codes), add them together and then keep 1. For example, a subject with 3 oral bup codes (any not distinct codes) on 1/1/2020 would have a start date of 1/1/2020 and rxsup=3. Keep just one of these records with rxsup=3 and enddate=(1/1/2020 + 3) -1 or 1/3/2020
- For oral bup procedures (G2068 or G2079), set rxsup=7 and enddate=adate + rxsup -1
- For procedures J0570, G2070, G2072: (buprenorphine rods; brand is probuphine): enddate=adate+179 days
- For procedure Q9991, Q9992, or G2069 (Sub Q buprenorphine; brand is sublocade): enddate=adate + 27 days
- For injectable naltrexone (from procedure codes J2315, G2073 or naltrexone XR injectable orders (brand=Vivitrol or route=intramuscular): enddate=adate (procedure) or rxdate (order) + 27 days

- Define the list of OUD ICD-9 and ICD-10 diagnosis codes

Opioid abuse	F11.1
Opioid abuse, uncomplicated	F11.10
Opioid abuse, in remission	F11.11
Opioid abuse with intoxication	F11.12
Opioid abuse with intoxication, uncomplicated	F11.120
Opioid abuse with intoxication delirium	F11.121
Opioid abuse with intoxication with perceptual disturbance	F11.122

Opioid abuse with intoxication, unspecified	F11.129
Opioid abuse with opioid-induced mood disorder	F11.14
Opioid abuse with opioid-induced psychotic disorder	F11.15
Opioid abuse with opioid-induced psychotic disorder with delusions	F11.150
Opioid abuse with opioid-induced psychotic disorder with hallucinations	F11.151
Opioid abuse with opioid-induced psychotic disorder, unspecified	F11.159
Opioid abuse with opioid-induced disorder	F11.18
Opioid abuse with opioid-induced sexual dysfunction	F11.181
Opioid abuse with opioid-induced sleep disorder	F11.182
Opioid abuse with other opioid-induced disorder	F11.188
Opioid abuse with unspecified opioid-induced disorder	F11.19
Opioid dependence, uncomplicated	F11.20
Opioid dependence, in remission	F11.21
Opioid dependence with intoxication, uncomplicated	F11.220
Opioid dependence with intoxication delirium	F11.221
Opioid dependence with intoxication with perceptual disturbance	F11.222
Opioid dependence with intoxication, unspecified	F11.229
Opioid dependence with withdrawal	F11.23
Opioid dependence with opioid-induced mood disorder	F11.24
Opioid dependence with opioid-induced psychotic disorder with delusions	F11.250
Opioid dependence with opioid-induced psychotic disorder with hallucination	F11.251
Opioid dependence with opioid-induced psychotic disorder, unspecified	F11.259
Opioid dependence with other opioid-induced disorder	F11.28
Opioid dependence with opioid-induced sexual dysfunction	F11.281
Opioid dependence with opioid-induced sleep disorder	F11.282
Opioid dependence with other opioid-induced disorder	F11.288
Opioid dependence with unspecified opioid-induced disorder	F11.29
Opioid type dependence, unspecified	304.00
Opioid type dependence, continuous	304.01
Opioid type dependence, episodic	304.02
Opioid type dependence, in remission	304.03
Combinations of opioid type drug with any other drug dependence, unspecified	304.70
Combinations of opioid type drug with any other drug dependence, continuous	304.71
Combinations of opioid type drug with any other drug dependence, episodic	304.72
Combinations of opioid type drug with any other drug dependence, in remission	304.73
Opioid abuse, unspecified	305.50
Opioid abuse, continuous	305.51
Opioid abuse, episodic	305.52

Opioid abuse, in remission	305.53

- Define the list of opioid overdose ICD-9 and ICD-10 diagnosis codes

Poisoning by, adverse effect of and underdosing of opium	T40.0
Poisoning by, adverse effect of and underdosing of opium	T40.0X
Poisoning by opium, accidental (unintentional)	T40.0X1
Poisoning by opium, accidental (unintentional), initial encounter	T40.0X1A
Poisoning by opium, accidental (unintentional), subsequent encounter	T40.0X1D
Poisoning by opium, accidental (unintentional), sequela	T40.0X1S
Poisoning by opium, intentional self-harm	T40.0X2
Poisoning by opium, intentional self-harm, initial encounter	T40.0X2A
Poisoning by opium, intentional self-harm, subsequent encounter	T40.0X2D
Poisoning by opium, intentional self-harm, sequela	T40.0X2S
Poisoning by opium, assault	T40.0X3
Poisoning by opium, assault, initial encounter	T40.0X3A
Poisoning by opium, assault, subsequent encounter	T40.0X3D
Poisoning by opium, assault, sequela	T40.0X3S
Poisoning by opium, undetermined	T40.0X4
Poisoning by opium, undetermined, initial encounter	T40.0X4A
Poisoning by opium, undetermined, subsequent encounter	T40.0X4D
Poisoning by opium, undetermined, sequela	T40.0X4S
Poisoning by and adverse effect of heroin	T40.1
Poisoning by and adverse effect of heroin	T40.1X
Poisoning by heroin, accidental (unintentional	T40.1X1
Poisoning by heroin, accidental (unintentional), initial encounter	T40.1X1A
Poisoning by heroin, accidental (unintentional), subsequent encounter	T40.1X1D
Poisoning by heroin, accidental (unintentional), sequela	T40.1X1S
Poisoning by heroin, intentional self-harm,	T40.1X2
Poisoning by heroin, intentional self-harm, initial encounter	T40.1X2A
Poisoning by heroin, intentional self-harm, subsequent encounter	T40.1X2D
Poisoning by heroin, intentional self-harm, sequela	T40.1X2S
Poisoning by heroin, assault	T40.1X3
Poisoning by heroin, assault, initial encounter	T40.1X3A
Poisoning by heroin, assault, subsequent encounter	T40.1X3D
Poisoning by heroin, assault, sequela	T40.1X3S
Poisoning by heroin, undetermined	T40.1X4
Poisoning by heroin, undetermined, initial encounter	T40.1X4A
Poisoning by heroin, undetermined, subsequent encounter	T40.1X4D
Poisoning by heroin, undetermined, sequela	T40.1X4S
Poisoning by, adverse effect of and underdosing of other opioids	T40.2
Poisoning by, adverse effect of and underdosing of other opioids	T40.2X

Poisoning by other opioids, accidental (unintentional)	T40.2X1
Poisoning by other opioids, accidental (unintentional), initial encounter	T40.2X1A
Poisoning by other opioids, accidental (unintentional), subsequent encounter	T40.2X1D
Poisoning by other opioids, accidental (unintentional), sequela	T40.2X1S
Poisoning by other opioids, intentional self-harm,	T40.2X2
Poisoning by other opioids, intentional self-harm, initial encounter	T40.2X2A
Poisoning by other opioids, intentional self-harm, subsequent encounter	T40.2X2D
Poisoning by other opioids, intentional self-harm, sequela	T40.2X2S
Poisoning by other opioids, assault	T40.2X3
Poisoning by other opioids, assault, initial encounter	T40.2X3A
Poisoning by other opioids, assault, subsequent encounter	T40.2X3D
Poisoning by other opioids, assault, sequela	T40.2X3S
Poisoning by other opioids, undetermined	T40.2X4
Poisoning by other opioids, undetermined, initial encounter	T40.2X4A
Poisoning by other opioids, undetermined, subsequent encounter	T40.2X4D
Poisoning by other opioids, undetermined, sequela	T40.2X4S
Poisoning by methadone, accidental (unintentional)	T40.3X1
Poisoning by methadone, accidental (unintentional), initial encounter	T40.3X1A
Poisoning by methadone, accidental (unintentional), subsequent encounter	T40.3X1D
Poisoning by methadone, accidental (unintentional), sequela	T40.3X1S
Poisoning by methadone, intentional self-harm, initial encounter	T40.3X2
Poisoning by methadone, intentional self-harm, initial encounter	T40.3X2A
Poisoning by methadone, intentional self-harm, subsequent encounter	T40.3X2D
Poisoning by methadone, intentional self-harm, sequela	T40.3X2S
Poisoning by methadone, assault	T40.3X3
Poisoning by methadone, assault, initial encounter	T40.3X3A
Poisoning by methadone, assault, subsequent encounter	T40.3X3D
Poisoning by methadone, assault, sequela	T40.3X3S
Poisoning by methadone, undetermined	T40.3X4
Poisoning by methadone, undetermined, initial encounter	T40.3X4A
Poisoning by methadone, undetermined, subsequent encounter	T40.3X4D
Poisoning by methadone, undetermined, sequela	T40.3X4S
Poisoning by other synthetic narcotics, accidental (unintentional)	T40.4X1
Poisoning by other synthetic narcotics, accidental (unintentional), initial encounter	T40.4X1A
Poisoning by other synthetic narcotics, accidental (unintentional), subsequent encounter	T40.4X1D
Poisoning by other synthetic narcotics, accidental (unintentional), sequela	T40.4X1S
Poisoning by other synthetic narcotics, intentional self-harm	T40.4X2
Poisoning by other synthetic narcotics, intentional self-harm, initial encounter	T40.4X2A

Poisoning by other synthetic narcotics, intentional self-harm, subsequent encounter	T40.4X2D
Poisoning by other synthetic narcotics, intentional self-harm, sequela	T40.4X2S
Poisoning by other synthetic narcotics, assault	T40.4X3
Poisoning by other synthetic narcotics, assault, initial encounter	T40.4X3A
Poisoning by other synthetic narcotics, assault, subsequent encounter	T40.4X3D
Poisoning by other synthetic narcotics, assault, sequela	T40.4X3S
Poisoning by other synthetic narcotics, undetermined	T40.4X4
Poisoning by other synthetic narcotics, undetermined, initial encounter	T40.4X4A
Poisoning by other synthetic narcotics, undetermined, subsequent encounter	T40.4X4D
Poisoning by other synthetic narcotics, undetermined, sequela	T40.4X4S
Poisoning by opioids	965.0
Poisoning by opium (alkaloids), unspecified	965.00
Poisoning by heroin	965.01
Poisoning by methadone	965.02
Poisoning by other opiates and related narcotics	965.09
Accidental poisoning by heroin	E850.0
Accidental poisoning by methadone	E850.1
Accidental poisoning by other opiates and related narcotics	E850.2

- Define the list of AUD codes

Alcohol abuse, uncomplicated	F10.10
Alcohol abuse, remission	F10.11
Alcohol abuse with intoxication, uncomplicated	F10.120
Alcohol abuse with intoxication delirium	F10.121
Alcohol abuse with intoxication, unspecified	F10.129
Alcohol abuse with alcohol-induced mood disorder	F10.14
Alcohol abuse with alcohol-induced psychotic disorder with delusions	F10.150
Alcohol abuse with alcohol-induced psychotic disorder with hallucinations	F10.151
Alcohol abuse with alcohol-induced psychotic disorder, unspecified	F10.159
Alcohol abuse with alcohol-induced anxiety disorder	F10.180
Alcohol abuse with alcohol-induced sexual dysfunction	F10.181
Alcohol abuse with alcohol-induced sleep disorder	F10.182
Alcohol abuse with other alcohol-induced disorder	F10.188
Alcohol abuse with unspecified alcohol-induced disorder	F10.19
Alcohol dependence, uncomplicated	F10.20
Alcohol dependence, in remission	F10.21
Alcohol dependence with intoxication, uncomplicated	F10.220
Alcohol dependence with intoxication delirium	F10.221
Alcohol dependence with intoxication, unspecified	F10.229
Alcohol dependence with withdrawal, uncomplicated	F10.230
Alcohol dependence with withdrawal delirium	F10.231
Alcohol dependence with withdrawal with perceptual disturbance	F10.232

Alcohol dependence with withdrawal, unspecified	F10.239
Alcohol dependence with alcohol-induced mood disorder	F10.24
Alcohol dependence with alcohol-induced psychotic disorder with delusions	F10.250
Alcohol dependence with alcohol-induced psychotic disorder with hallucinations	F10.251
Alcohol dependence with alcohol-induced psychotic disorder, unspecified	F10.259
Alcohol dependence with alcohol-induced persisting amnestic disorder	F10.26
Alcohol dependence with alcohol-induced persisting dementia	F10.27
Alcohol dependence with alcohol-induced anxiety disorder	F10.280
Alcohol dependence with alcohol-induced sexual dysfunction	F10.281
Alcohol dependence with alcohol-induced sleep disorder	F10.282
Alcohol dependence with other alcohol-induced disorder	F10.288
Alcohol dependence with unspecified alcohol-induced disorder	F10.29
Alcohol withdrawal delirium	291.0
Alcohol withdrawal	291.81
Acute alcoholic intoxication in alcoholism, unspecified	303.00
Acute alcoholic intoxication in alcoholism, continuous	303.01
Acute alcoholic intoxication in alcoholism, episodic	303.02
Acute alcoholic intoxication in alcoholism, in remission	303.03
Other and unspecified alcohol dependence	303.9
Other and unspecified alcohol dependence, unspecified	303.90
Other and unspecified alcohol dependence, continuous	303.91
Other and unspecified alcohol dependence, episodic	303.92
Other and unspecified alcohol dependence, in remission	303.93
Alcohol abuse	305.0
Alcohol abuse, unspecified	305.00
Alcohol abuse, continuous	305.01
Alcohol abuse, episodic	305.02
Alcohol abuse, in remission	305.03

- Drop naltrexone injections in the pre randomization period if there are not 2+ visits with an OUD/OD diagnosis (can be 1 OUD and 1 OD code) in the pre randomization period.
- Drop naltrexone injections in the post randomization period if there are not 2+ visits with an OUD/OD diagnoses (can be 1 OUD and 1 OD code) in the pre or post randomization period.
- Adjudicate subjects with both 2+ OUD/OD and 1+ AUD diagnosis codes to decide whether to include or exclude as OUD treatment.

16) Using the start and end dates of **each bup (including probuphine and sublocade) and naltrexone order or procedure** (and adding dispensed buprenorphine and/or methadone in a sensitivity analysis), create continuous episodes of use. This will result in a start and end date for each continuous OUD treatment. Continuous is defined as <=7 days gap between end date and subsequent start date or <= 14 days gap if bup end date and XR injectable naltrexone start date (allow for washout period prior to starting XR injectable naltrexone). There may be multiple continuous episodes for any given subject because there will be gaps (see below) or breaks in continuous treatment.

17) **Determine treatment day**

a. Order all of a patient's orders and procedures by start and end dates. This should include rx and procedures 180 days before day 1 of the time period of interest because these may run out into the time period of interest.
b. For example: Within a period of interest starting on 1/1/20, a bup order occurring on 12/25/20 for 30 days (end date 1/24/20) would contribute 20 treatment days to January.
c. During time periods of interest, consider it a day "covered" by buprenorphine or injectable naltrexone during the period of interest if there is bup or naltrexone that covers that day based on rx start and end date. Do not double count if >1 bup or naltrexone on the same day.

Estimate begin and stop dates for each continuous episode (continuous defined as a gap ≤ 7 days between end of one bup rx and start of the next bup rx; <=14 days between bup end and naltrexone start, and <=7 days between naltrexone end and bup or naltrexone start) for all the bup and naltrexone in the time period of interest. In other words, the <=14 rule may only be applied when the last day before the gap is covered by bup only and the first day after the bup gap is covered by naltrexone only.

a. Treatment days = sum of all days covered by bup or naltrexone during the period of interest. No overlap or double counting of treatment days is allowed. In other words, a patient can have a max of 365 days covered by treatment in a year.
    i. Left and right truncate when time period begins and ends
        1. For example: Bup order that covers 12/15/2019 to 1/14/220 would stop contributing treatment days on 12/31/2019 if the period of interest ends on 12/31/2019.

Sensitivity analyses (SA): The above steps will be replicated for

1) A SA that only includes episodes with at least 2 buprenorphine orders omitting last order (and any refills on the order) in an oral buprenorphine treatment episode
2) A SA that varies the allowable 7-day gap of continuous use to a lower threshold (based on distribution of the gaps; example: 2 days).
3) A SA that uses a combination of orders and dispensings at health systems with some or all (one health system) dispensings data.

For each dispensing, if its rxdate is in "the range" of an order date or a refill date, then it is considered to be linked to that order or refill and dropped in calculations; otherwise, this dispensing is considered as not linked to any order and added to the calculation of days covered. The range is defined as dispensed within 30 days <u>after</u> the order/refill's start date (an order's start date is the order's date; a refill's start date is derived from the original order's date and days supply).

This calculation will be done after all data cleaning and imputation.

1) A SA that adds in procedure codes for methadone maintenance therapy at sites where this data is available
2) A SA that combines SA # 3 and #4 at sites with both dispensing and methadone maintenance therapy data.

A3. Combine OUD Treatments of Buprenorphine and Naltrexone

1) Order all OUD treatments by their respective start and runout dates. See Examples below.
2) During time periods of interest, consider it a day "covered" by OUD treatment during the period of interest if there is buprenorphine or injectable naltrexone that covers that day based on start and runout dates. Do not double count covered days.
3) Estimate start and end dates for each continuous episode (continuous defined as a gap ≤ 7 days between runout date of one OUD treatment and start date of the next treatment OR ≤14 days between end date of one buprenorphine prescription and start date of naltrexone) based on days covered for all the buprenorphine and naltrexone in the time period of interest.
4) Any gaps not meeting these criteria above result in the end of a continuous episode and these gaps between episodes are NOT part of any episode and do not contribute to treatment days. See examples below.
5) Pre and post randomization episodes are not allowed to overlap. They will be left and right censored accordingly. For example, a treatment episode of 90 days with 15 days in the post randomization period (includes randomization date) will be split into two episodes with one episode contributing 75 days of treatment pre-randomization and a second episode contributing 15 days of treatment post randomization.
6) OUD treatment days = sum of all episodes [(episode end date – episode start date) + 1)] in the pre and post randomization periods.

14.0 Examples 1-8 below are each a single patient's prescriptions. These examples are shortened to a 20-day period of interest as an example only. Our perspective here is the prescribers' "intent to treat" (i.e., we do not know how or if patients actually took the oral medications but the assumptions we are making are typical of studies that use electronic health data for studying medication treatment and adherence). (Bup=buprenorphine; NTX=naltrexone)

**Example 1: Bup**	**TIME PERIOD OF INTEREST**																				
	**-1**	**1**	**2**	**3**	**4**	**5**	**6**	**7**	**8**	**9**	**10**	**11**	**12**	**13**	**14**	**15**	**16**	**17**	**18**	**19**	**20**
Bup1																					
Bup2																					
Bup3																					
Bup4																					
Bup5																					
Bup 1 covers day 1 (from a rx occurring during -90 days of period of interest as depicted by coverage on -1 day) and ends day 8; Bup 2 starts day 8 and ends day 11; Bup 3 starts day 12 and ends day 15; Bup 4 starts day 15 and ends day 16; Bup 5 starts day 19 and covers day 19 and 20 – may end on day 20 or after period of interest (truncated).<br>One continuous episode: Day 1 to Day 20. The gap (day 17 and day 18) is 2 days (≤7 days) so smooth it to be continuous.<br>20 of the 20 day time period of interest is covered by treatment.																					

**Example 2: Bup**	**TIME PERIOD OF INTEREST**

	**1**	**2**	**3**	**4**	**5**	**6**	**7**	**8**	**9**	**10**	**11**	**12**	**13**	**14**	**15**	**16**	**17**	**18**	**19**	**20**
Bup1																				
Bup2																				
Bup3																				
Bup4																				
Bup5																				
Bup 1 covers day 1 and ends day 6; Bup 2 starts day 1 and ends day 11; Bup 3 starts day 6 and ends day 8; Bup 4 starts day 15 and ends day 16; Bup 5 starts day 16 and ends day 19.<br>One continuous episode: Day 1 to Day 20. The gap is 4 days (<=7 days) so smooth it to be continuous.																				

**Example 3: Bup & NTX**	**TIME PERIOD OF INTEREST**																			
	1	2	3	4	5	6	7	8	9	10	11	12	13	14	15	16	17	18	19	20
Bup1																				
NTX1																				
Bup 1 covers day 1 and ends day 4; NTX1 starts day 10 and covers through day 20 (right truncated since end date would be past time period of interest of 20 days).<br>One continuous episode: Day 1 to Day 20. The gaps in treatment (day 5-9) are ≤14 days.<br>20 of the 20 day time period of interest are covered by treatment.																				

**Example 4: Bup & NTX**	**TIME PERIOD OF INTEREST**																			
	1	2	3	4	5	6	7	8	9	10	11	12	13	14	15	16	17	18	19	20
NTX1																				
Bup1																				
NTX1 1 covers day 1 and ends day 9 (left truncated because it must have begun prior to day 1 given inj ntx lasts 28 days); Bup1 starts day 18 and covers though day 20 (likely right truncated) in the period of interest.<br>Two continuous episodes: 1st episode is Day 1 to Day 9. Then there is a gap >7 days so a new episode begins. 2nd episode starts day 18 and ends day 20.<br>12 of the 20 day time period of interest are covered by treatment.																				

**Example 5: Bup**	**TIME PERIOD OF INTEREST**																			
	1	2	3	4	5	6	7	8	9	10	11	12	13	14	15	16	17	18	19	20
Bup1																				
Bup2																				
BP1 1 covers day 1 and ends day 9 (possibly left truncated if it began prior to day 1; Bup2 starts day 7 and covers though day 17.<br>One continuous episode: Day 1 to Day 17. No double counting of overlap days (7-9).<br>17 of the 20 day time period of interest are covered by treatment.																				

**Example 6: Bup & NTX**	**TIME PERIOD OF INTEREST**																			
	1	2	3	4	5	6	7	8	9	10	11	12	13	14	15	16	17	18	19	20
Bup1																				
NTX1																				
NTX2																				
BP1 1 covers day 1 and ends day 4 (possibly left truncated if it began prior to day 1; NTX1 also covers day 1 and ends day 4 (left truncated); NTX2 starts day 10 and ends day 20 (right truncated)<br>One continuous episode: Day 1 to Day 20. The gap is 5 days (<=7) so smooth it to be continuous.																				

**Example 7: Bup & NTX**	**TIME PERIOD OF INTEREST**																			
	1	2	3	4	5	6	7	8	9	10	11	12	13	14	15	16	17	18	19	20
Bup1																				
NTX1																				
NTX2																				
BP1 1 covers day 1 and ends day 4 (possibly left truncated if it began prior to day 1; NTX1 covers day 1 and ends day 3 (left truncated); NTX2 starts day 10 and ends day 20 (right truncated).<br>One episode: Day 1 to Day 20. No double counting of overlap in days between bup1 and ntx1. There is <=14 day gap between when bup1 ends (last drug before NTX2) and when NTX2 starts so it is continuous.<br>20 of the 20 day time period of interest are covered by treatment.																				

**Example 8: Bup & NTX**	**TIME PERIOD OF INTEREST**																			
	1	2	3	4	5	6	7	8	9	10	11	12	13	14	15	16	17	18	19	20
Bup1																				
NTX1																				
NTX2																				
BP1 1 covers day 1 and ends day 4 (possibly left truncated if it began prior to day 1; NTX1 covers day 1 and ends day 5 (left truncated); NTX2 starts day 10 and ends day 20 (right truncated).<br>One continuous episode: Day 1 to Day 20. The gap is 4 days (<=7) so smooth it to be continuous.																				

Appendix B. Initial Values for Power Simulations

The following is a justification of the initial values used in Step 2 of the data generation model for the Primary Objective 1 Outcome power simulations.

Consider a lag-1 autoregressive time series given by

$$y_{t+1} = \xi + \psi\, y_t + e$$

where ξ and ψ are constants, *e* is normally distributed with mean zero and variance σ_e^2 and *t* indexes time interval Letting η denote the mean of any particular y_t, and ς the variance, then we have

$$E(y_{t+1}) = \xi + \psi\, E(y_t)$$
$$\Rightarrow \eta = \xi + \psi\, \eta$$
$$\Rightarrow \eta = \frac{\xi}{1-\psi}$$

and

$$Var(y_{t+1}) = \psi^2\, Var(y_t) + Var(e)$$
$$\Rightarrow \varsigma = \psi^2\, \varsigma + \sigma_e^2$$
$$\Rightarrow \varsigma = \frac{\sigma_e^2}{1-\psi^2}.$$

Appendix C. Small-sample Degree of Freedom (DF) Correction Method for Objective 2 Primary Analysis

Recent literature has highlighted the need to incorporate small-sample correction methods when analyzing data from cluster-randomized trials (CRTs) when there are few (e.g., <30) clusters (Kahan et al. 2016; Leyrat et al. 2017). In particular, it has been documented that when there are few clusters the usual approaches to analyzing correlated data in CRTs, including generalized estimating equations (GEE) and generalized linear mixed models (GLMM; the proposed analysis approach), can yield inflated type 1 error rates (Kahan et al. 2016). For example, applying a traditional test based on these models that has a nominal type 1 error rate of 5%, may lead to actual type 1 error rates that are much larger (e.g., 10-20%) such that we would reject the null hypothesis that the intervention is effective, even if it is truly not effective, above the pre-specified acceptable level.

Although corrections for tests of treatment effects under GLMM with few clusters have been studied, there remain gaps in knowledge of which test to use in the context of PROUD Objective 2 analyses. In particular, although it is common to adjust for covariates (such as the baseline value of the outcome as in PROUD analyses), covariate-adjusted analyses have not received attention in existing literature. Moreover, despite extensive research on corrections when the outcome is continuous or binary, to our knowledge the performance of the methods for count outcomes (such as the number of days of acute care utilization) has not been studied.

To address these gaps in the statistical literature (and to gain insight on how to account for the small number of clusters for PROUD Objective 2 analyses), we investigated the performance of various testing procedures for GLMM via simulation. The tests we considered were all eight combinations of two forms of test and four methods to compute the denominator degree of freedom (DDF). The forms of test we considered were the Wald t-test and the likelihood ratio F-test (LRT). The methods to compute DDF we considered were residual, containment, between-within 1 (BW1), and between-with 2 (BW2) (also referred to as “inner-outer”). BW1 and BW2 are two generalizations of the BW method to covariate-adjusted models, and BW has been shown to perform well via various scenarios in simulation (Li & Redden 2015).

We conducted simulations for count outcomes (i.e., Poisson GLMM) with various numbers of clusters, mean and coefficient of variation (CV) of clusters sizes, and intraclass correlation coefficients (ICC) characterizing the correlation of patients within a cluster. We also vary the number and the level (individual- vs. cluster-level) of (i) prognostic variables in the data-generating model, and (ii) extra (i.e., non-prognostic) covariates in the correctly specified fitted model. We consider including extra covariates to account for the potential that non-prognostic covariates may be included in secondary analyses. Additional details on the simulation study setup are described in a manuscript in preparation and are available upon request.

Selected Results

Here we present selected simulation study results that correspond to the settings of the primary Objective 2 analysis that adjusts for a single covariate (baseline value of the outcome), as well as for the secondary analysis that adjusts for additional person-level covariates. For these settings, the GLMM fitted to the data was the correctly specified model (i.e., the model did not include redundant covariates).

Figure C1. Type 1 Error Rates from the Setting with Data-generating Model that Includes a Single Person-level Covariate (setting for primary Objective 2 analysis that adjusts solely for the baseline value of the outcome)

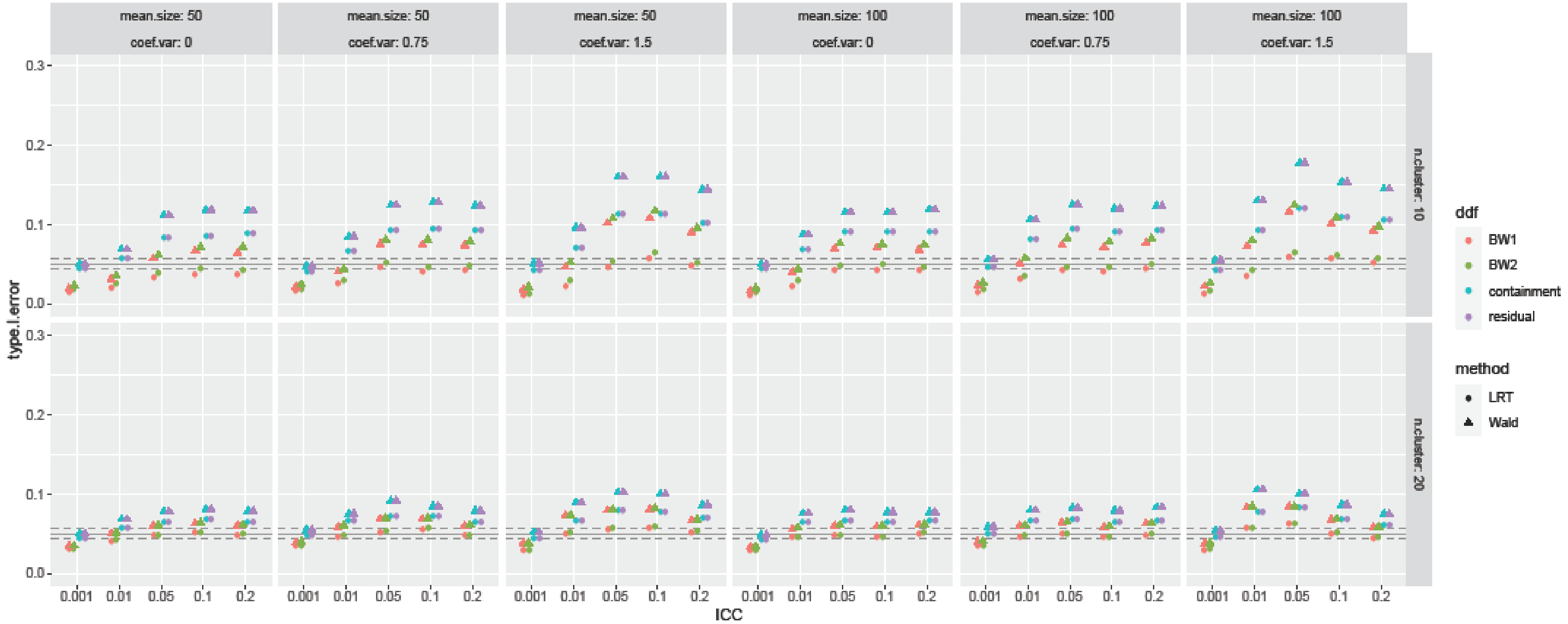

Figure C2. Type 1 Error Rates from the Setting with Data-generating Model that Includes Multiple Person-level Covariate (setting for sensitivity Objective 2 analysis that adjusts for multiple covariates in addition to the baseline value of the outcome)

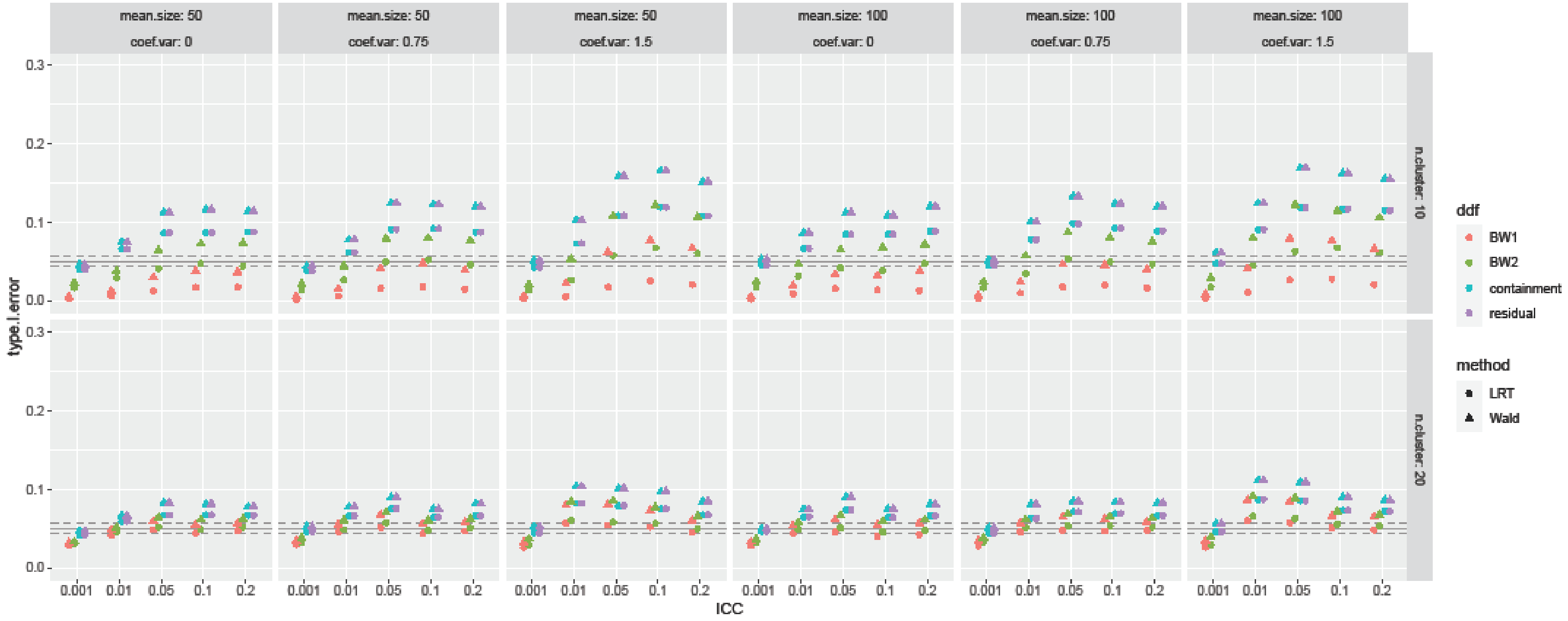

Implications for PROUD Objective 2 Analyses

None of the tests considered performed uniformly well across all data generating scenarios considered, and the optimally performing test (i.e., with type 1 error rate closest to the nominal 0.05 level), varied highly depending on the scenario.

With the Objective 2 analysis clustered at the clinic level (see Section 5.3), the scenario that most closely aligns with our setting, based on PROUD Phase 1 data, is given in the top-right plot of the two Figures above. (For Phase 1, the ICC under a linear mixed model was approximately 0.02, average sample size within a cluster was approximately 100, and CV of cluster sizes was 1.2.)

For the results that align with the covariate adjustment setting under the primary analysis approach (Figure C1), the LRT with either BW1 or BW2 have reasonable type 1 error rates. For the results that align with the covariate adjustment setting under the secondary analysis approach (Figure C2), LRT with BW2 performs reasonably well (whereas with BW1 performance was overly conservative). We therefore selected LRT with BW2 (also referred to as “inner-outer”) for the testing approach that incorporates small sample methods

Year 3 Supplement to Statistical Analytic Plan

Health care system-specific intervention effects for the primary outcome

Here we provide details on how health care system-specific intervention effects were calculated for the primary outcome. Specifically, we compared the primary outcome in the PROUD clinic with the Usual Care clinic within each health system, by calculating the difference, in the change from baseline to follow-up, between the PROUD intervention and usual care clinic (denoted by Δ). To obtain 95% confidence intervals, we used a non-parametric bootstrap approach to resample patient-level data from each clinic with replacement (500 resampling iterations). Resampled datasets used the number of eligible patients assigned to PROUD intervention or usual care clinics equal to the number in the original data. For each resampled data set, we calculated the t-test statistic for Δ that was centered at the Δ from the original data and divided by the pooled variance of the changes in both arms. Calculations assumed that changes in each arm were independent and that the number of eligible patients within each clinic remained constant over time. Using the 2.5% and 97.5% quantiles of the 500 t-statistics and the pooled variance of the changes in both arms based on the original data, we calculated the 95% confidence intervals.

This above method was applied to secondary restricted outcomes. However, ongoing OUD treatment cases were rare (<0.01%), so the bootstrap resampling does not create a proper empirical variation. Thus, we used z-test statistic rather than bootstrap t-statistics by using the normal approximation.

Post-hoc permutation test of the primary outcome

We conducted permutation tests using a clinic-level linear model adjusting for the patient years of OUD treatment at baseline to confirm robustness of the primary mixed model analysis. There are 64 possible permutation tests by flipping the randomization of two clinics within each health system. Among the adjusted treatment effects from the 64 permutations, only 2 resulted in an absolute mean difference equal to or greater than the primary outcome (mean different of 19.7 patient-years of OUD treatment per 10,000 patients). Therefore, the exact p-value of the permutation test is 0.031 (=2/64) for a two-sided test, which is the smallest p-value possibly obtained from the permutation test. The permutation result supports the robustness of the significant treatment effect in the primary outcome.
