# Supplementary material for: Nurse Care Management of Opioid Use Disorder Treatment After 3 Years: A Secondary Analysis of the PROUD Cluster Randomized Clinical Trial
Source: JAMA Netw Open. 2024 Nov 22;7(11):e2447447. doi: 10.1001/jamanetworkopen.2024.47447 (PMC11584924; doi:10.1001/jamanetworkopen.2024.47447)
Supplement: Supplement 2. — eFigure 1. PROUD Study Flow Diagram eFigure 2. PROUD Study Timeline and Sample, With an Additional Year for the Nurse Care Manager and Outcome Measurement eFigure 3. Calendar Dates of When the Nurse Was Hired and When the Nurse Was Seeing Patients in Each Health System After Randomization eFigure 4. Number of Patients Treated for OUD in Intervention and Usual Care Clinics per 10,000 Primary Care Patients per Month: Cumulative Numbers of Patients Treated for OUD Since Randomization, and Numbers in Active OUD Treatment Each Month From Baseline Through Follow-Up (Unadjusted) eFigure 5. Cumulative Numbers of Patients Treated for OUD per 10,000 Patients Seen by Trial Arm for Each Health System During 3 Years of Follow-Up in Intervention and Usual Care Clinics, Unadjusted for Baseline [file jamanetwopen-e2447447-s002.pdf]

Supplemental Online Content

Lapham GT, Hyun N, Bobb JF, et al. Implementation of nurse care management of opioid use disorder treatment after 3 years: a secondary analysis of the PROUD cluster-randomized clinical trial. *JAMA Netw. Open*. 2024;7(11):e2447447. doi:10.1001/jamanetworkopen.2024.47447

eFigure 1. PROUD Study Flow Diagram

eFigure 2. PROUD Study Timeline and Sample, With an Additional Year for the Nurse Care Manager and Outcome Measurement

eFigure 3. Calendar Dates of When the Nurse Was Hired and When the Nurse Was Seeing Patients in Each Health System After Randomization

eFigure 4. Number of Patients Treated for OUD in Intervention and Usual Care Clinics per 10,000 Primary Care Patients per Month: Cumulative Numbers of Patients Treated for OUD Since Randomization, and Numbers in Active OUD Treatment Each Month From Baseline Through Follow-Up (Unadjusted)

eFigure 5. Cumulative Numbers of Patients Treated for OUD per 10,000 Patients Seen by Trial Arm for Each Health System During 3 Years of Follow-Up in Intervention and Usual Care Clinics, Unadjusted for Baseline

This supplemental material has been provided by the authors to give readers additional information about their work.

© 2024 Lapham GT et al. *JAMA Network Open.*

eFigure 1. PROUD Study Flow Diagram

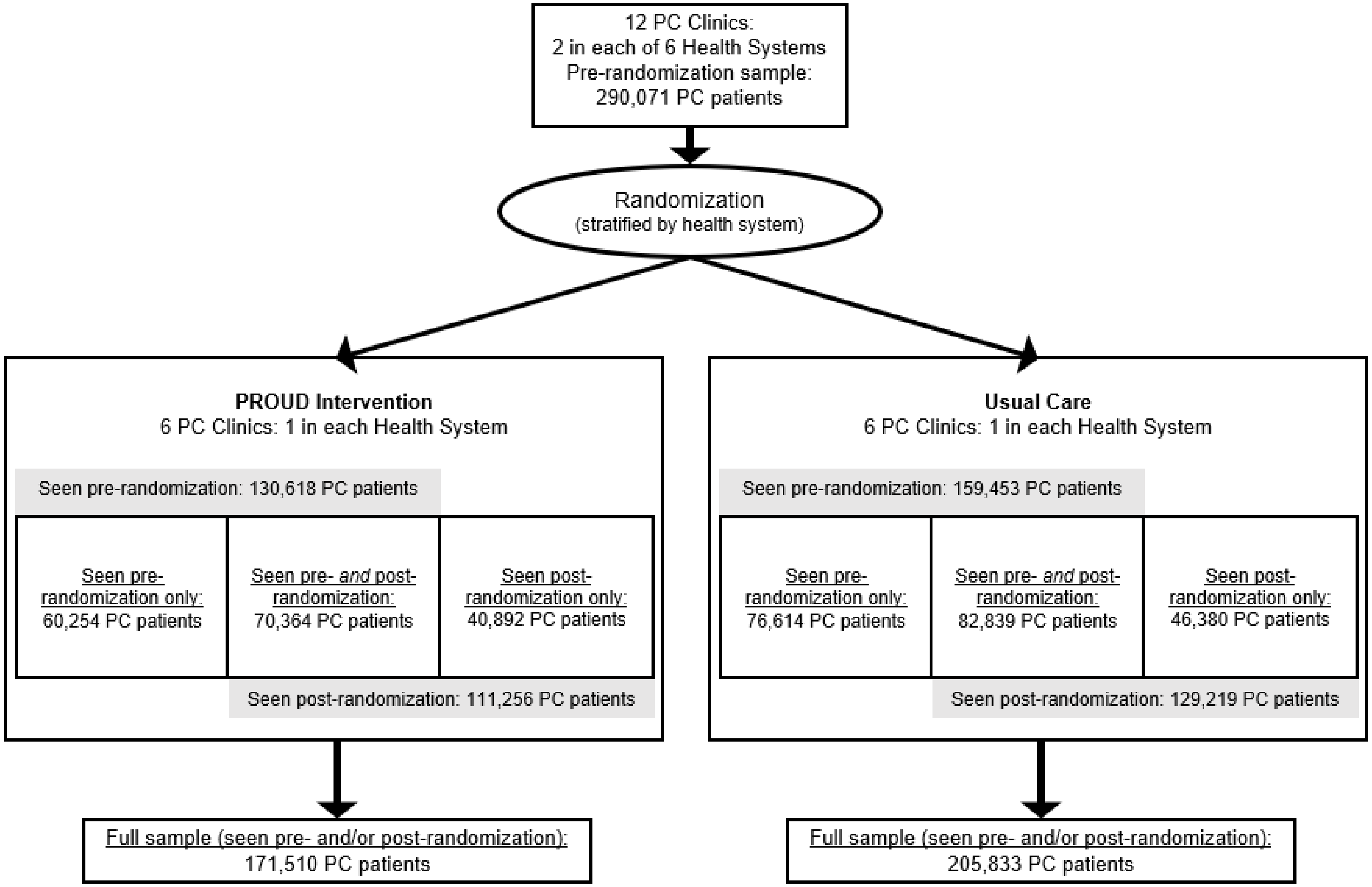

Note, study data was based on a final data abstraction (May 2021), which was intended to ensure data completeness and occurred subsequent to analysis of main trial results. In the 9 months between data pulls (August 2020 to May 2021), some claims records were amended or corrected, resulting in some patients being reassigned clinics or removed, resulting in minor differences between this consort diagram and the original.[1,2]
PC=primary care; SD=standard deviation

1. Schneeweiss S, Rassen JA, Brown JS, et al. Graphical depiction of longitudinal study designs in health care databases. *Annals of internal medicine*. 2019;170(6):398-406.
2. Wartko PD, Bobb JF, Boudreau DM, et al. Nurse Care Management for Opioid Use Disorder Treatment: The PROUD Cluster Randomized Clinical Trial. *JAMA Internal Medicine*. 2023

© 2024 Lapham GT et al. *JAMA Network Open.*

eFigure 2. PROUD Study Timeline and Sample, With an Additional Year for the Nurse Care Manager and Outcome Measurement

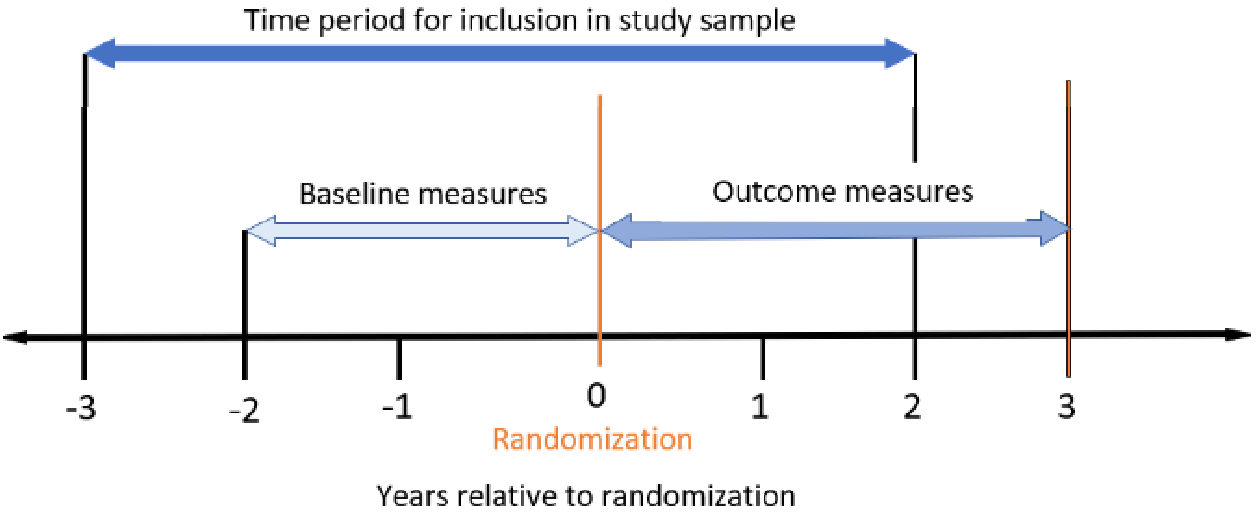

© 2024 Lapham GT et al. *JAMA Network Open.*

eFigure 3. Calendar Dates of When the Nurse Was Hired and When the Nurse Was Seeing Patients in Each Health System After Randomization[a]

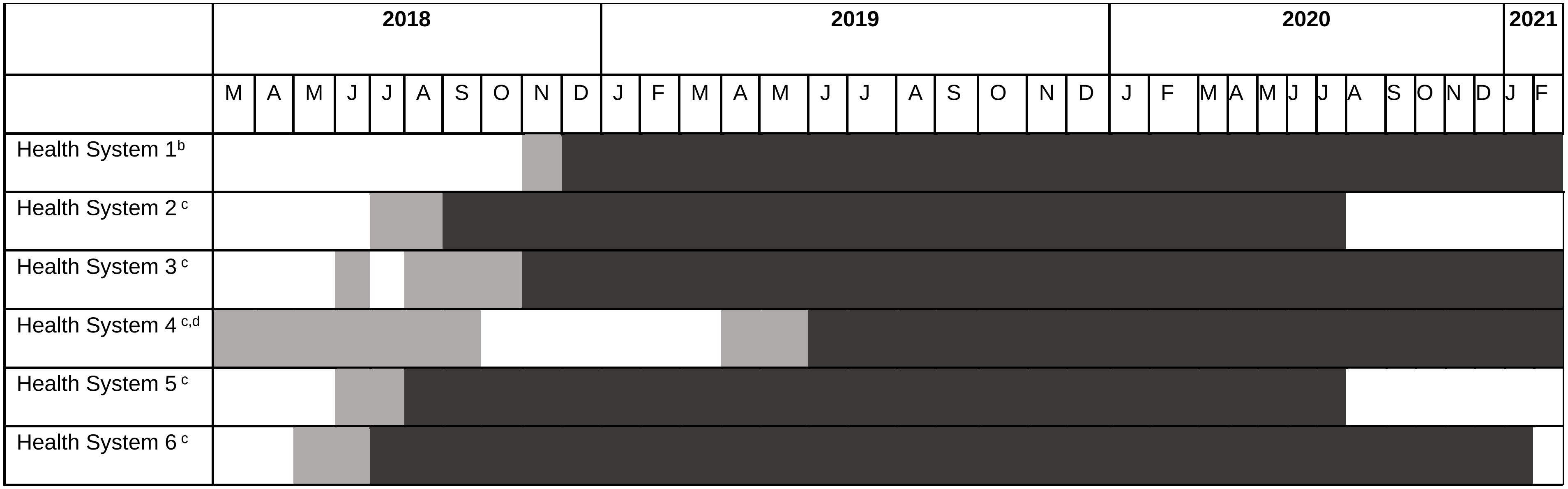

[a] Health systems learned which clinic was randomized to the PROUD intervention, and therefore would need to hire a nurse, on the day of randomization. Then, all but one health system had to search for, hire, and onboard a new nurse before they could be trained at Boston Medical Center. While we had estimated 6 months on average for this process, there was considerable variability.

[b] Health system 1 was notified of the clinics' random assignment on 8/31/2018 (6 months later than other health systems due to a delay in execution of the data use agreement and therefore delayed randomization).

[c] Health systems 2–6 were notified of the clinics' random assignment on 2/28/2018.

[d] Health system 4 had a nurse on staff at randomization but that nurse left before ever seeing patients with OUD in the intervention clinic, leading to a gap without a nurse before another was hired.

No nurse
Nurse hired but not seeing patients
Nurse seeing patients

© 2024 Lapham GT et al. *JAMA Network Open.*

eFigure 4. Number of Patients Treated for OUD in Intervention and Usual Care Clinics per 10,000 Primary Care Patients per Month: Cumulative Numbers of Patients Treated for OUD Since Randomization, and Numbers in Active OUD Treatment Each Month From Baseline Through Follow-Up (Unadjusted)

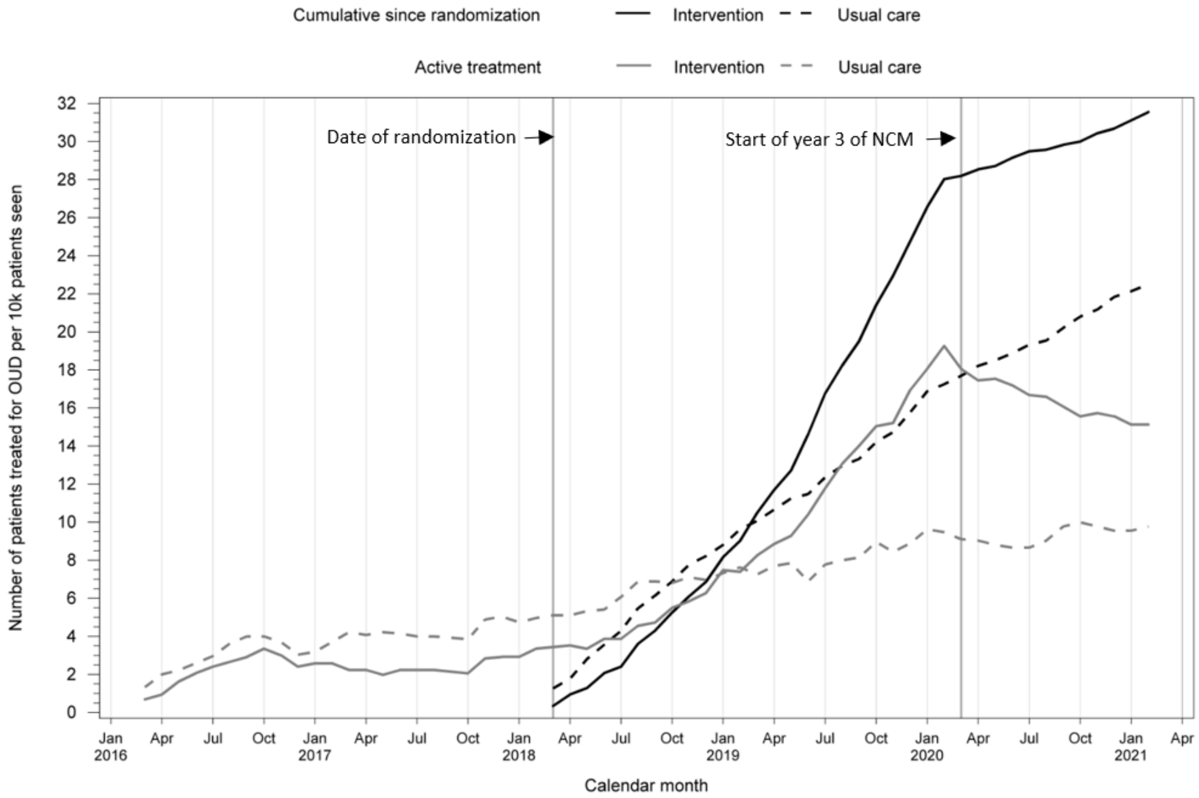

© 2024 Lapham GT et al. *JAMA Network Open.*

eFigure 5. Cumulative Numbers of Patients Treated for OUD per 10,000 Patients Seen by Trial Arm for Each Health System During 3 Years of Follow-Up in Intervention and Usual Care Clinics, Unadjusted for Baseline

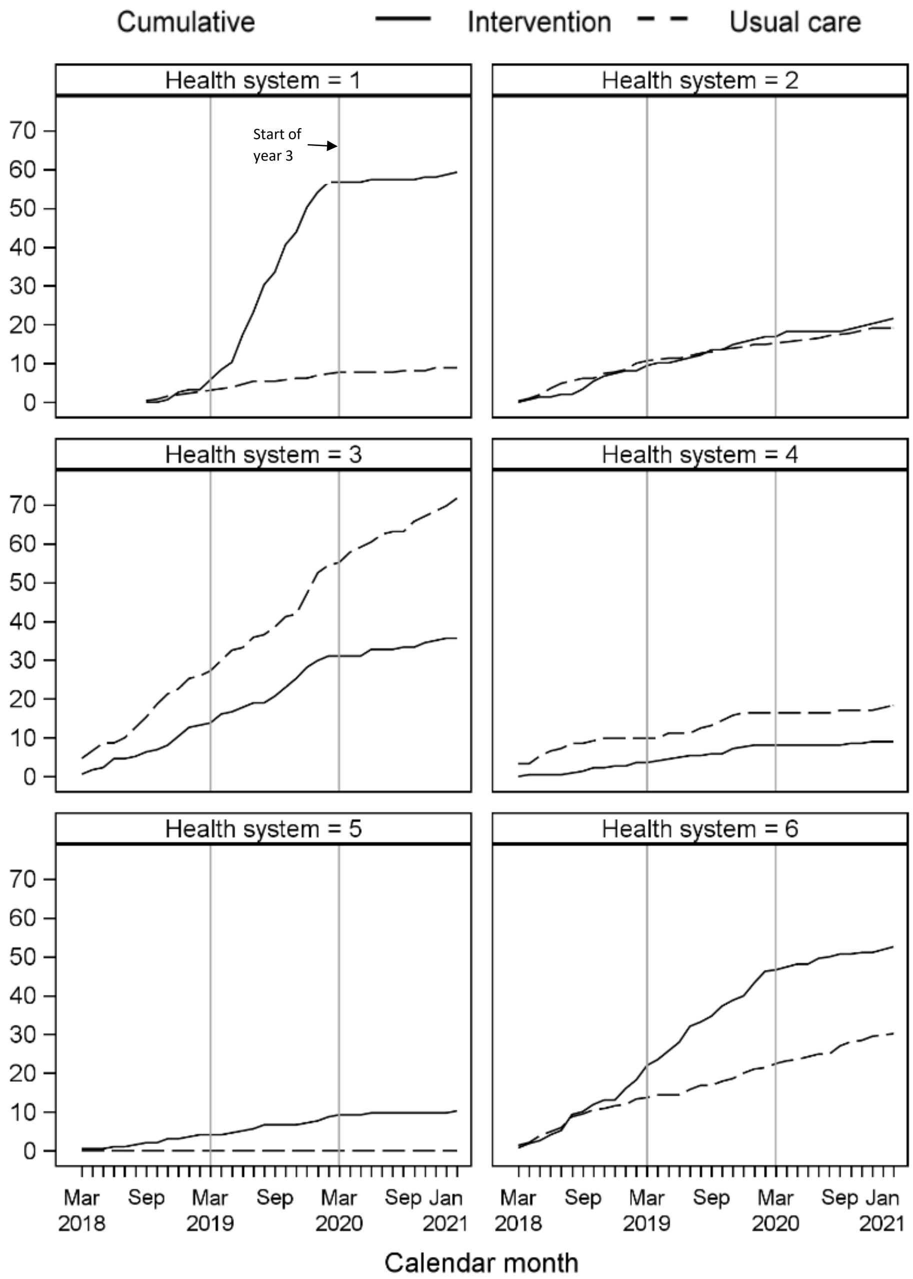

© 2024 Lapham GT et al. *JAMA Network Open.*
