# Supplementary material for: Nurse Care Management of Opioid Use Disorder Treatment After 3 Years: A Secondary Analysis of the PROUD Cluster Randomized Clinical Trial
Source: JAMA Netw Open. 2024 Nov 22;7(11):e2447447. doi: 10.1001/jamanetworkopen.2024.47447 (PMC11584924; doi:10.1001/jamanetworkopen.2024.47447)
Supplement: Supplement 3. — Data Sharing Statement [file jamanetwopen-e2447447-s003.pdf]

Data Sharing Statement

Lapham. Effect of Nurse Care Management on Office-Based Opioid Use Disorder Treatment. *JAMA Netw Open*. Published November 22, 2024. doi:10.1001/jamanetworkopen.2024.47447

Data
Additional Information: ClinicalTrials.gov Identifier: NCT03407638
https://clinicaltrials.gov/study/NCT03407638?titles=PRimary%20care%20Opioid%20Use%20Disorders%20treatment%20(PROUD)%20trial%20&rank=1
Data available: Yes
Data types: Deidentified participant data
How to access data: Investigators wishing to obtain the main analytic dataset or analytic datasets for secondary analyses should contact the Lead Investigator and project manager. Requests will be reviewed on a case-by-case basis and may require funding to support 1) programming to create the necessary de-identified analytic dataset(s) and 2) establish a data transfer agreement and Institutional Review Board approval. Any data shared will honor the original data use agreements with study sites. The contact information for this study's Lead Investigator (Dr. Katharine Bradley) is katharine.a.bradley@kp.org ; the project manager is Megan Addis, whose email is Megan.J.Addis@kp.org.When available: With publication
When available: With publication

Supporting Documents
Document types: None

Additional Information
Who can access the data: Researchers whose proposed use of the data has been approved by lead investigator and project manager
Types of analyses: Deidentified participant data for secondary Analyses
Mechanisms of data availability: Requests will be reviewed on a case-by-case basis and may require funding to support 1) programming to create the necessary de-identified analytic dataset(s) and 2) establish a data transfer agreement and Institutional Review Board approval. Any data shared will honor the original data use agreements with study sites.
